# Concise Synthesis of Cyctetryptomycin A and B Enabled by Zr‐Catalyzed Dimerization

**DOI:** 10.1002/anie.202414295

**Published:** 2024-11-06

**Authors:** Longhui Yu, Hiroshige Ogawa, Shangzhao Li, Tsoh Lam Cheung, Wenchao Liu, Dexiu Yan, Yudai Matsuda, Yusuke Kobayashi, Zhihong Guo, Kotaro Ikeda, Trevor A. Hamlin, Ken Yamazaki, Pei‐Yuan Qian, Hugh Nakamura

**Affiliations:** ^1^ The Hong Kong University of Science and Technology (HKUST) Clear Water Bay, Kowloon Hong Kong SAR China; ^2^ Southern Marine Science and Engineering Guangdong Laboratory (Guangzhou) Nansha Guangzhou China; ^3^ City University of Hong Kong Tat Chee Avenue Kowloon Hong Kong SAR China; ^4^ Kyoto Pharmaceutical University 5 Nakauchi-cho, Misasagi Yamashina-ku Kyoto 607-8414 Japan; ^5^ Vrije Universiteit Amsterdam De Boelelaan 1108 1081 HZ Amsterdam The Netherlands.; ^6^ Division of Applied Chemistry, Okayama University Tsushimanaka Okayama 700-8530 Japan

**Keywords:** natural product synthesis, Zr catalysis, dimerization, chemoenzymatic synthesis

## Abstract

A concise synthetic strategy utilizing a Zr catalyst for the construction of cyctetryptomycin A and B is reported. Cyctetryptomycin A and B are recently isolated, complex tetrameric natural products for which total synthesis has not been previously reported. This study presents a practical approach for the construction of two consecutive quaternary carbon centers with a Zr catalyst. Furthermore, the first total synthesis of cyctetryptomycin A and B was achieved by this Zr‐catalyzed radical coupling. The radical dimerization reaction mediated by the Zr catalyst required 1,2‐bis(diphenylphosphino)ethane (dppe) as an indispensable additive. Through both experimental and theoretical investigations into the mechanism of this Zr‐catalyzed reaction, the specific role of dppe was elucidated. In addition, the synthetic approach was extended to enable the practical synthesis of other dimeric natural products, including tetratryptomycin A, dibrevianamide F, and ditryptophenaline. Finally, the synthetic mechanism of cyctetryptomycin A and B, through the oxidative macrocyclization of tetratryptomycin A by CttpC, was newly elucidated by both experimental and docking simulations.

## Graphical abstract

A concise synthesis of cyctetryptomycin A and B employing a zirconium‐catalyzed dimerization and CttpC‐catalyzed oxidative macrocyclization is presented, including in‐depth mechanistic analysis of the zirconium‐catalyzed C−C bond formation and its various applications. Additionally, the previously unknown detailed reaction mechanism of the CttpC‐catalyzed oxidative macrocyclization of tetratryptomycin A has been elucidated.

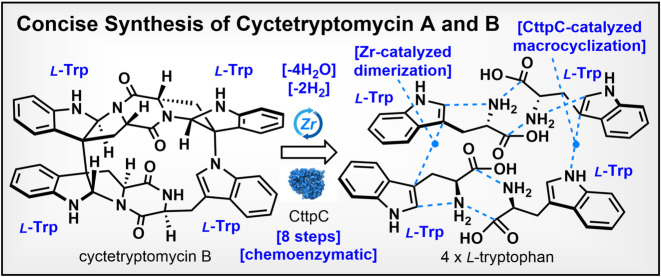

## Introduction

**Figure 1:**
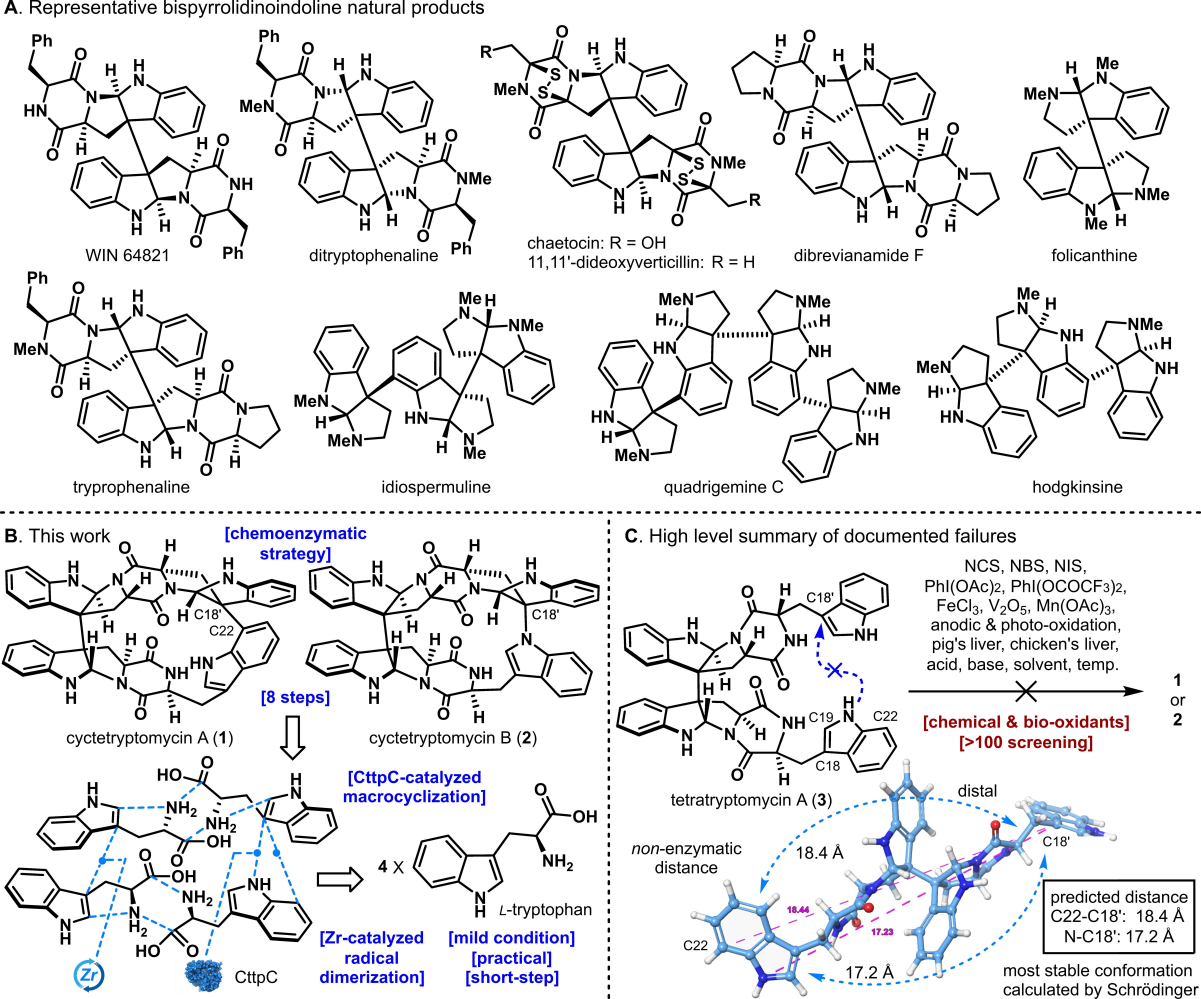
A) Representative bispyrrolidinoindoline natural products. B) This work. C) High level summary of documented failures.

Cyctetryptomycin A and B (**1**–**2**) (Figure 1) are unprecedented tetrameric tryptophan natural products with a highly strained linkage, isolated in 2021.^[1]^ Cyctetryptomycin A and B (**1**–**2**) possess potent neuroprotective activity, making them promising candidates for novel therapeutics targeting neurodegenerative diseases such as ALS, Alzheimer's disease, and Parkinson's disease.^[1]^ However, the total synthesis of cyctetryptomycin A and B (**1**–**2**) has not yet been reported. In this study, a practical and mild method using a novel Zr catalyst was developed for the dimerization of tryptophan derivatives at the C3−C3 position. Following this, synthesis of cyctetryptomycin A and B (**1**–**2**) was achieved in 8 steps via subsequent CttpC‐catalyzed oxidative coupling.

Tryptophan‐derived natural products have garnered significant interest in the field of synthetic chemistry, not only due to their diverse and potent biological activities but also because of their intricate structures.^[3]^ Numerous C3‐linked tryptophan derivative dimers have been isolated and reported thus far (Figure 1A).[^6^, ^13^] Common methods for C3−C3 dimerization of tryptophan derivatives include reactions using Co,^[21]^ Ni,^[26]^ Cu,^[29]^ Fe,^[30]^ V_2_O_5_,^[32]^ or hypervalent iodine reagents,^[34]^ which are generally recognized for this transformation (see Supporting Information). While these methods are effective, they often necessitate over one equivalent of metal reagents, strong acidic conditions, or expensive catalysts and ligands. Additionally, issues related to stereoselectivity and regioselectivity frequently arise in the C3−C3 dimerization reactions of tryptophan derivatives, leading to mixtures of stereoisomers and regioisomers. Although some direct dimerization conditions from tryptophan have been documented, these methods are plagued by incomplete stereoselectivity and regioselectivity. Therefore, a user‐friendly and practical novel C3−C3 dimerization reaction of tryptophan derivatives is reported here.

The retrosynthetic analysis of cyctetryptomycin A and B (**1**–**2**) is outlined in Figure 1B. These compounds exhibit a highly complex three‐dimensional structure arising from the intricate linkage of four tryptophan units. Hence, developing a mild synthetic route that circumvents harsh conditions is crucial. To this end, the dimerization of tryptophan derivatives was planned using a mild, cost‐effective Zr catalyst under radical conditions. The formation of the C22−C18′ and N−C18′ linkages was intended to be achieved via oxidative macrocyclization. Tetratryptomycin A (**3**) is considered a pivotal intermediate in the biosynthetic pathway of cyctetryptomycin A and B (**1**–**2**). Consequently, an initial attempt was made to synthesize cyctetryptomycin A and B (**1**–**2**) via the oxidative macrocyclization of tetratryptomycin A (**3**) (Figure 1C).^[36]^ Despite screening over 100 different oxidation conditions on tetratryptomycin A (**3**), cyctetryptomycin A and B (**1**–**2**) were not obtained. Subsequent computational analysis of the lowest energy conformation of tetratryptomycin A (**3**) revealed that the oxidative macrocyclization was hindered by significant distances between the C22−C18′ and N−C18′ positions. Computational data indicated that the most stable conformation of tetratryptomycin A (**3**) showed a C22−C18′ distance of 18.4 Å and an N−C18′ distance of 17.2 Å. These findings suggest that the spatial separation of the tryptophan units in tetratryptomycin A (**3**) hindered the intended oxidative macrocyclization. Major side products observed during the oxidative macrocyclization of tetratryptomycin A (**3**) included compounds where the indole moiety was converted to oxindole or covalently halogenated. Thus, this study focused on the efficient synthesis of tetratryptomycin A (**3**) via a C3−C3 dimerization reaction of tryptophan derivatives, followed by its oxidative macrocyclization to construct cyctetryptomycin A and B (**1**–**2**).^[38]^

## Results and Discussion

**Figure 2:**
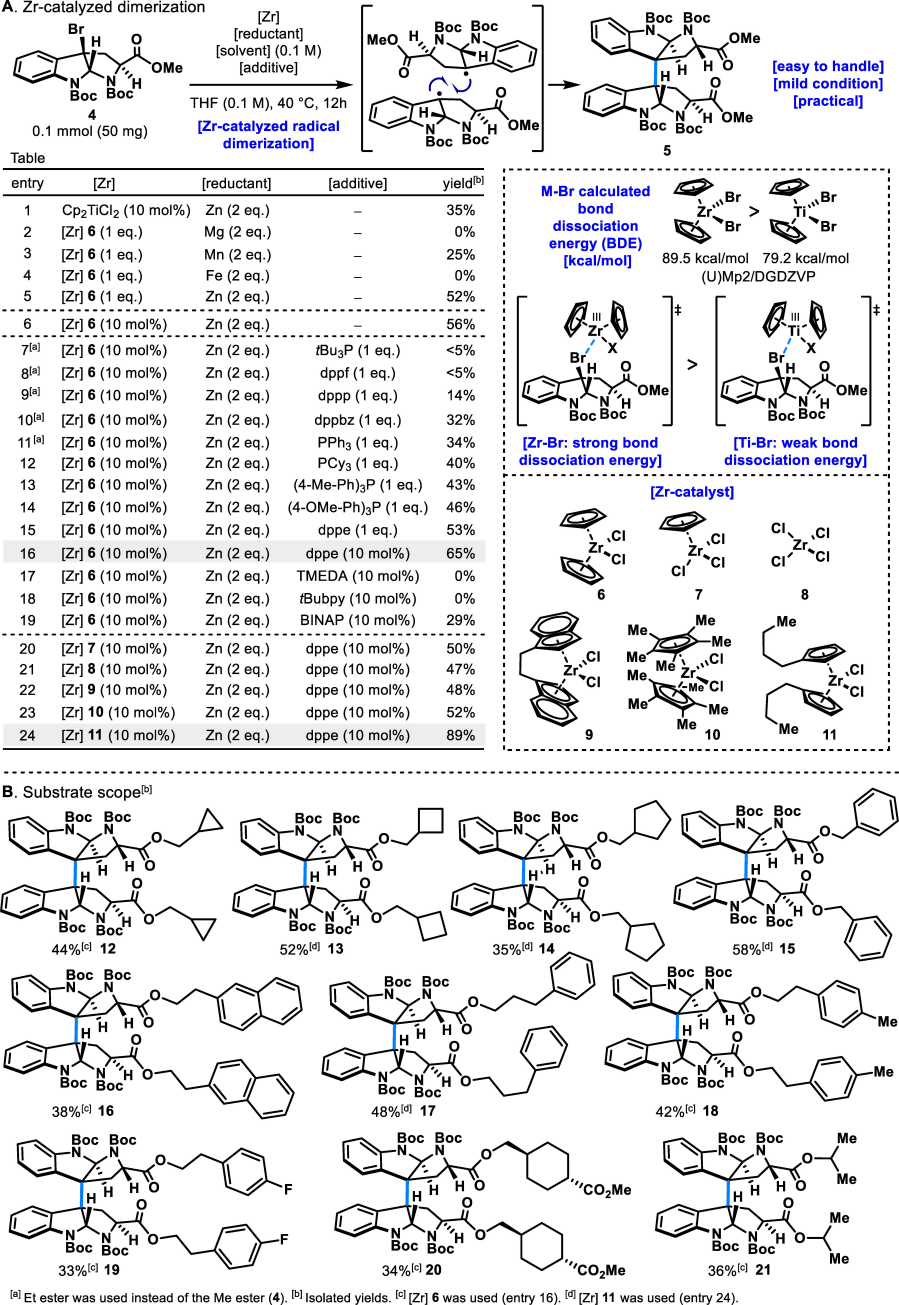
A) Zirconium‐catalyzed dimerization. B) Substrate scope.

Initially, the development of a mild and practical C3−C3 dimerization reaction for tryptophan derivative **4** was attempted (Figure 2A). As previously mentioned, several methods for C3−C3 dimerization of tryptophan derivatives have been reported.[^21^, ^22^, ^23^, ^24^, ^25^, ^26^, ^27^, ^28^, ^29^, ^30^, ^31^, ^32^, ^33^, ^34^] However, these methods often require over one equivalent of metal reagents, involve metal reagents that quickly deactivate in the presence of atmospheric oxygen, necessitate strong acidic conditions, or demand expensive catalysts and ligands. Considering these limitations, we explored a simpler and more economically viable practical dimerization reaction (table in Figure 2A). Titanium catalysts, being affordable and geopolitically stable, were an attractive option. Accordingly, screening various reaction conditions using titanium catalysts revealed that Cp_2_TiCl_2_ (10 mol %) afforded the desired dimeric product **5** in a 35 % isolated yield (entry 1).

In recent years, Zr‐catalyzed radical reactions have been actively studied due to their mild reaction conditions and low toxicity.^[43]^ Zirconium also possesses physical properties similar to titanium. Using DFT calculations, the bond dissociation energies (BDE) of Ti−Br in Cp_2_TiBr_2_ and Zr−Br in Cp_2_ZrBr_2_ were predicted. It was found that the Ti−Br bond in Cp_2_TiBr_2_ is 79.2 kcal mol^−1^, whereas the Zr−Br bond in Cp_2_ZrBr_2_ is 89.5 kcal mol^−1^. These results suggested that Cp_2_ZrCl_2_ could more readily generate radicals from **4** compared to Cp_2_TiCl_2_. Therefore, to improve yields, we investigated the dimerization of tryptophan derivative **4** using inexpensive Cp_2_ZrCl_2_.

Initially, various reductants were examined for this reaction with Cp_2_ZrCl_2_ (1.0 eq.). The reaction did not proceed at all with Mg (entry 2). Using Mn as the reductant, the target dimer **5** was obtained, but the yield was only 25 % (entry 3). Fe was found to be unsuitable (entry 4). Further exploration revealed Zn to be the optimal reductant, yielding dimer **5** in a 52 % yield on a 0.1 mmol (50 mg) scale (entry 5). The reaction also proceeded well with 10 mol % of Cp_2_ZrCl_2_, affording a 56 % yield of the desired dimer **5** (entry 6). Solvent screening revealed that THF provided the highest yield (see Supporting Information).

Despite these advancements, inconsistent yields during scale‐up with Cp_2_ZrCl_2_ catalysis prompted an investigation of various additives to stabilize reaction reproducibility. Ligands such as bipyridine and phenanthroline were ineffective. Phosphine ligands including *t*Bu_3_P, dppf, dppp, dppbz, PPh_3_, PCy_3_, (4‐Me−Ph)_3_P, and (4‐OMe−Ph)_3_P were also largely ineffective (entries 7–14). However, employing dppe (1.0 eq.) produced the dimer **5** with a 53 % yield and good reproducibility (entry 15). Further optimization using 10 mol % dppe improved the isolated yield to 65 % with consistent results (entry 16). Other bidentate ligands, such as TMEDA, *t*Bubpy, and BINAP, were ineffective (entries 17–19).

Subsequent screening of various Zr catalysts revealed that multiple Zr catalysts could facilitate the dimerization reaction, although no significant yield improvements were observed (entries 20–23). Notably, the use of Zr catalyst (**11**) (10 mol %), Zn (2 eq.), and dppe (10 mol %) dramatically improved the reaction, yielding dimer **5** in 89 % isolated yield (entry 24). This demonstrated the superior efficacy of Zr catalyst (**11**) in this dimerization reaction.

The optimized conditions were applicable to various tryptophan derivatives (Figure 2B). Substrates with cyclopropane, cyclobutane, and cyclopentane alkyl esters were readily converted to the corresponding dimers (**12**–**14**). Application of this reaction to benzyl ester gave the desired dimer (**15**) in 58 % yield. The reaction was also successful with substrates containing naphthalene and aromatic rings linked by alkyl chains (**16**, **17**). A substrate with a methyl group on the aromatic ring yielded dimer (**18**) in 42 %. Conversely, a substrate with an electron‐deficient F on the aromatic ring gave a slightly lower yield (**19**). Chiral and secondary alcohol‐derived esters also participated well in the reaction, producing the corresponding dimers (**20**, **21**). During the investigation of the substrate scope, yields tended to decrease when substrates with bulky ester substituents, such as **14**, **20**, and **21**, were used. These results suggest that the reaction may be sensitive to the steric hindrance of ester substituents, which can be a limitation.

**Figure 3:**
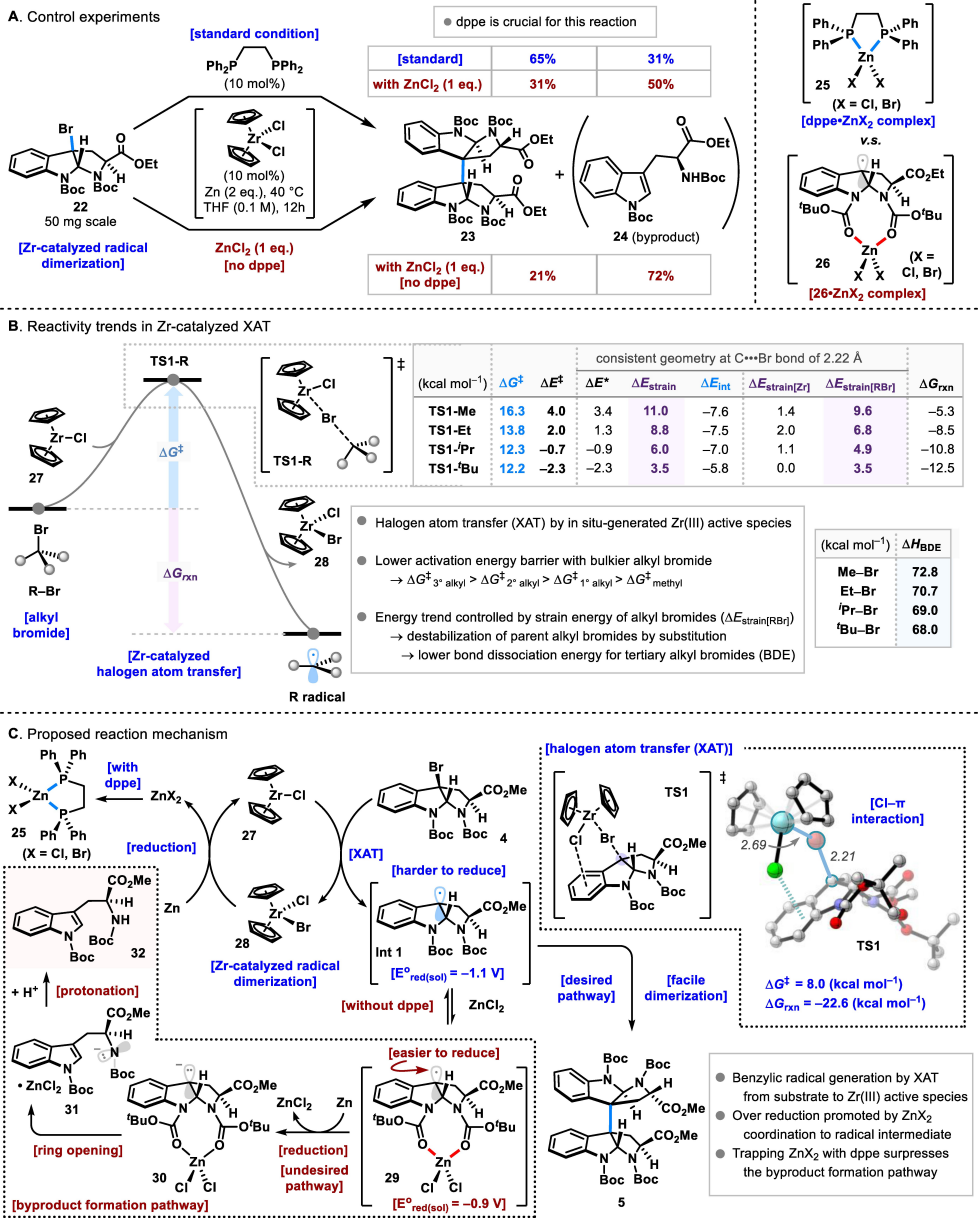
A) Control experiments. B) Reactivity trends in Zr‐catalyzed XAT. C) Proposed reaction mechanism.

To investigate the role of dppe, control experiments were conducted (Figure 3A). Under standard conditions (Figure 2A, entry 16), 31 % yield of the over‐reduced product **24** and 65 % yield of the dimerized product **23** were obtained. When 1.0 equivalent of ZnCl_2_ was added under the same conditions, the yield of the dimerized product **23** decreased to 31 %, and the yield of the over‐reduced product increased to 50 %. Furthermore, when the 1.0 equivalent of ZnCl_2_ was added to the standard conditions without dppe, the over‐reduced product was obtained as a major product, in 72 % yield. These results suggest that dppe plays a crucial role in sequestering ZnCl_2_ and inhibiting the formation of the over‐reduced product. It is hypothesized that dppe chelates to ZnCl_2_ and forms complex (**25**), thereby preventing the formation of ZnCl_2_/substrate complex (**26**). Consequently, the byproduct formation pathway is inhibited, and desired dimerization is promoted.

In order to elucidate the reaction mechanism of the Zr‐catalyzed halogen atom transfer (XAT) process, a density functional theory (DFT) study was performed (Figure 3B). The reaction is initiated by an *in situ* generation of the Zr(III) active species from Cp_2_ZrCl_2_ in the presence of zinc, and this can promote the abstraction of halogen atoms from various alkyl bromides.^[43]^ We found that the Zr‐catalyzed XAT process is generally exergonic with a relatively low energy barrier (Scheme 3B). The reaction with methyl bromide goes with the highest reaction barrier (Δ*G* ^*≠*^=16.3 kcal mol^−1^), and the energy decreases as the number of substituents of the radical increases (Δ*G* ^*≠*^=13.8 [EtBr], 12.3 [*i*PrBr], 12.2 [*t*BuBr] kcal mol^−1^). To gain deeper insight into the origin of reactivity differences observed for the various alkyl bromides in XAT process, we turned to the activation strain model (ASM). The ASM involves the decomposition of the electronic energy Δ*E* into the strain energy Δ*E_strain_* associated with the structural deformation of the Zr(III) active species and the alkyl bromide from their equilibrium geometry and the interaction energy *ΔE*_int_ between these deformed reactants [Eq. 1].^[45]^

(1)

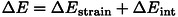




We performed our activation strain analysis (ASA) at a consistent geometry on the reaction coordinate near all of the XAT transition states with a C(alkyl)−Br stretch of 2.22 Å. Performing this analysis at a consistent geometry instead of at the transition state ensures that the results are not erroneously skewed by the fact that some transition states are earlier than others.^[51]^ Importantly, the trend in Gibbs free activation barriers is the same as for the electronic activation barriers (ΔE*) computed at the consistent geometry. The differences in reactivity can be traced back to differences in the strain energy, while the trend in interaction energy follows the opposite trend. The higher activation barrier for the methyl bromide originates from a more destabilizing strain energy of the substrate (Δ*E*_strain[RBr]_=9.6 kcal mol^−1^ [R=Me]), whereas the lower barrier for the tertiary alkyl bromide benefits from a less destabilized strain energy (Δ*E*_strain[RBr]_=3.5 kcal mol^−1^ [R=*t*Bu]). This trend in strain energy can be traced back to the strength of the C−Br bond that is broken during the XAT step, where the stronger primary alkyl bromide bond requires more energy to break compared to the tertiary alkyl bromide bond (see Figure 3B for the computed BDEs).^[52]^

Furthermore, we investigated the catalytic cycle with the tryptophane derivative **22** (Figure 3C). The XAT process is even more facile than for the tertiary alkyl bromide and goes with a lower energy barrier through the transition state **TS** **1** (Δ*G* ^*≠*^=8.0 kcal mol^−1^) to furnish a stable benzylic radical (Δ*G*_rxn_=−22.6 kcal mol^−1^). The optimized reaction conditions require the use of a catalytic amount of dppe, and this can be attributed to the role of phosphine that can prevent the over‐reduction of the benzylic radical **Int** **1** by zinc. In the absence of dppe, Zn(II) species are generated in the reaction mixture as the reaction proceeds, and this can act as a Lewis acid that promotes further single electron reduction of **Int** **1** to generate a benzylic anion. The anion easily undergoes the ring‐opening process, and upon the protonation the side product **24** is formed. The addition of dppe is crucial in this reaction, especially on a large scale, because this byproduct formation pathway is inhibited by the chelation of phosphine into Zn(II) species to maintain the appropriate concentration of the in situ generated benzylic radical. On the other hand, as shown in Figure 2A, using more than 10 mol % of dppe did not enhance the yield. This suggests that excessive dppe can also inhibit the desired reaction pathway and result in deteriorated yields.

**Figure 4:**
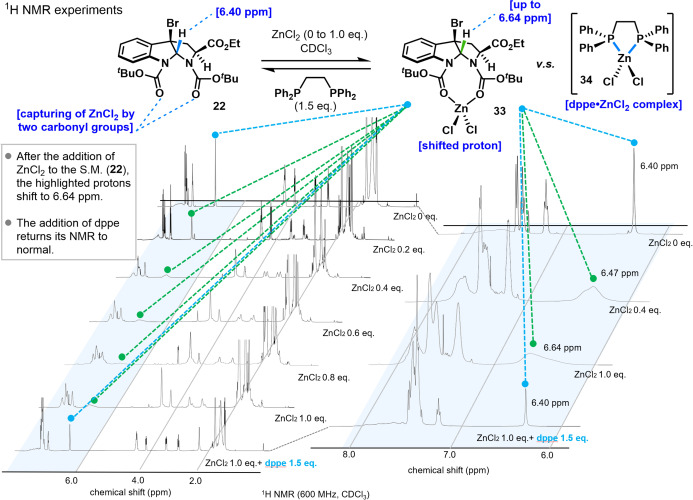
^1^H NMR experiments.

To gain further insights into the effect of dppe, ^1^H NMR titration experiments were conducted (Figure 4). Upon the addition of 0.2 equivalents of ZnCl_2_ to substrate **22**, the highlighted protons exhibited a downfield shift of 0.07 ppm compared to substrate **22**. Incremental addition of ZnCl_2_ resulted in a further downfield shift of these protons. When 1.0 equivalent was added, the shift was 0.24 ppm compared to substrate **22**. Notably, when 1.5 equivalents of dppe were added simultaneously with 1.0 equivalent of ZnCl_2_, the shifts observed without dppe were negated, aligning with the ^1^H NMR spectrum of substrate **22**. This indicates that the addition of dppe disrupts the ZnCl_2_/complex (**33**), forming the dppe/ZnCl_2_ complex (**34**).

Similarly, ^13^C NMR titration experiments were conducted. The addition of 0.2–1.0 equivalents of ZnCl_2_ to substrate **22** and tracking the ^13^C NMR changes revealed downfield shifts in the carbons near the coordination site. A 1.0 equivalent addition of ZnCl_2_ caused a 3.1 ppm downfield shift in the carbon adjacent to bromine. The carbonyl carbon of the Boc group (highlighted in blue) experienced a 1.0 ppm downfield shift upon the addition of 1.0 equivalent of ZnCl_2_. Similar to the ^1^H NMR results, no significant changes were observed in the ethyl ester chemist shifts. (see Supporting Information).

The chemical shifts observed in both ^1^H and ^13^C NMR upon the addition of ZnCl_2_ suggest the existence of the ZnCl_2_/complex (**33**). Additionally, comparing the ethyl ester chemical shifts of compounds **22** and **33**, the absence of changes suggests that the ethyl ester in compound **22** does not participate in the ZnCl_2_ interaction. These NMR trace experiments imply that dppe captures ZnCl_2_ formed during the dimerization reaction, suppressing the formation of byproducts **24** or **32** and thus improving reaction yields. The beneficial effect of dppe was particularly pronounced on gram scales.

Conversely, regarding compound **22**, attempts were made to utilize protective groups other than the Boc group to prevent the over‐reduced byproducts of this dimerization reaction. However, when using the *p*Ts (4‐toluenesulfonyl) group, the over‐reduced byproduct predominated as the main product, and the desired dimerization compound was scarcely obtained. These results elucidated that the Boc group is optimal for this dimerization reaction (see Supporting Information).

**Scheme 1:**
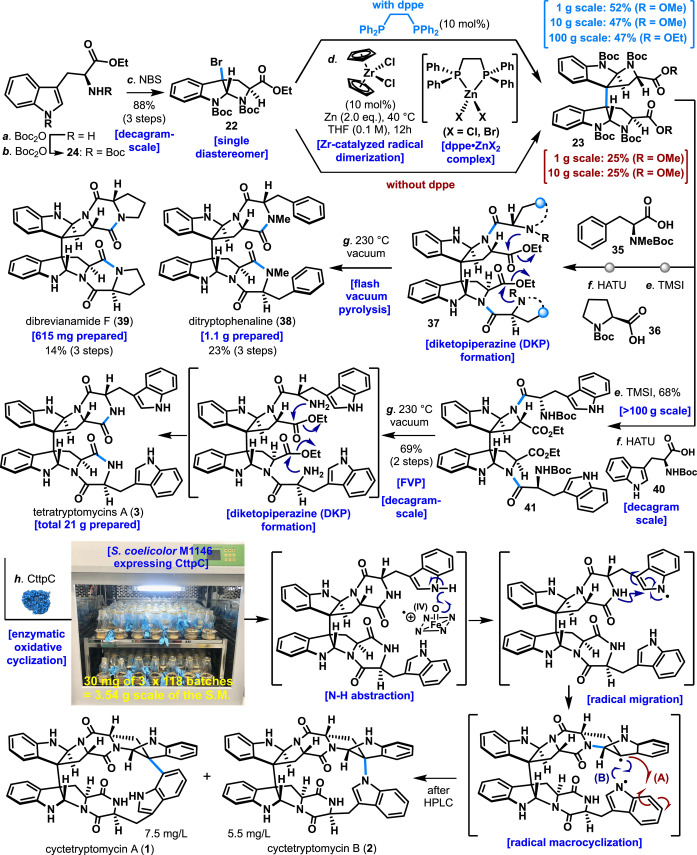
Concise synthesis of cyctetryptomycin A (**1**) and B (**2**) enabled by Zr‐catalyzed dimerization.^[a]^

Next, the total synthesis of cyctetryptomycin A and B (**1**–**2**) using the developed Zr‐catalyzed dimerization reaction was attempted (Scheme 1). The synthesis of **1** and **2** began with *L*‐tryptophan ethyl ester, protecting the nitrogen on the indole and primary amine with Boc groups to yield compound **24**. Bromination of **24** with NBS yielded the single diastereomer bromo cyclized compound **22**. The slow addition of NBS was crucial to isolate the single compound **22**, as rapid NBS addition produced an inseparable mixture of diastereomers. Dimerization of synthesized **22** using a Zr catalyst was then attempted. While the bulky Zr catalyst (**11**) was most effective, cost considerations led to the use of Cp_2_ZrCl_2_ for decagram‐scale reactions. Following the optimized conditions from Figure 2A, bromo cyclized compound **22** was treated with Cp_2_ZrCl_2_ catalyst (10 mol %), dppe (10 mol %), and Zn (2.0 eq.) in THF at 40 °C, yielding dimer **23** with 52 % isolated yield on a 1 g scale and 47 % on 10 g and 100 g scales. Notably, the reaction was scalable to 100 g. When dppe was omitted, the yield dramatically dropped to 25 % on 1 g and 10 g scales, underscoring the effectiveness of dppe (10 mol %) in this reaction, especially on larger scales. The additive dppe likely captures ZnCl_2_ formed during the reaction, preventing over‐reduction of the radical intermediate from **22** and thereby enhancing dimerization yields.

The obtained dimer **23** was deprotected of its four Boc groups using TMSI and coupled with *N*‐Boc‐*N*‐Me‐*L*‐phenylalanine (**35**) and Boc *L*‐proline (**36**) using HATU to yield **37**. Flash vacuum pyrolysis (FVP) then facilitated Boc deprotection and cyclization, forming diketopiperazine, leading to the synthesis of ditryptophenaline (**38**) and dibrevianamide F (**39**) in yields of 1.1 g and 615 mg respectively. Similarly, compound **41** was synthesized from dimer **23** and subjected to FVP, constructing two diketopiperazines and yielding 21 g of tetratryptomycin A (**3**).[^13^, ^32^]

The synthesis of cyctetryptomycin A and B (**1**–**2**) through oxidative macrocyclization of tetratryptomycin A (**3**) was then explored. We reported in 2021 that the enzyme CttpC catalyzes the oxidative macrocyclization of tetratryptomycin A (**3**) to cyctetryptomycin A and B (**1**–**2**).^[1]^ Computational chemistry previously indicated that the most stable conformation of tetratryptomycin A (**3**) has tryptophan units too distant for macrocyclization. Over 100 oxidative macrocyclizations were screened using chemical and biological oxidants (e.g., chicken and pig liver), but only oxindole or halogenated byproducts were obtained, with no macrocyclization. Therefore, conditions for mass culturing *S. coelicolor M1146* expressing CttpC, facilitating the oxidative macrocyclization of tetratryptomycin A (**3**) to cyctetryptomycin A and B (**1**–**2**), were investigated (see Supporting Information). Adequate oxygen supply was crucial, avoiding overly large Erlenmeyer flasks. Optimal conditions involved smaller flasks (125 ml and 50 ml), resulting in successful culture of numerous *S. coelicolor M1146* colonies (see Supporting Information). Under optimal conditions, 6 liters of *S. coelicolor M1146* culture containing CttpC were added to 3.54 grams of tetratryptomycin A (**3**) divided into 118 flasks (each 30 mg), stirred at 28–30 °C. This facilitated the oxidative macrocyclization of tetratryptomycin A (**3**), successfully synthesizing cyctetryptomycin A and B (**1**–**2**).

The mechanism of CttpC‐catalyzed oxidative macrocyclization of tetratryptomycin A (**3**) involves the formation of a high‐valent iron complex that abstracts an N−H proton, generating a radical on the indole nitrogen. Radical migration creates a C3 radical on the tryptophan unit, which then undergoes nucleophilic attack by the diketopiperazine‘s nitrogen, forming a pyrrolidinoindoline. The C3 radical of the tryptophan moiety then completes the macrocyclization by coupling with an intramolecular tryptophan nitrogen radical, yielding cyctetryptomycin B (**2**). Alternatively, the C3 radical can add to the C7 position of another tryptophan, generating cyctetryptomycin A (**1**).

**Figure 5:**
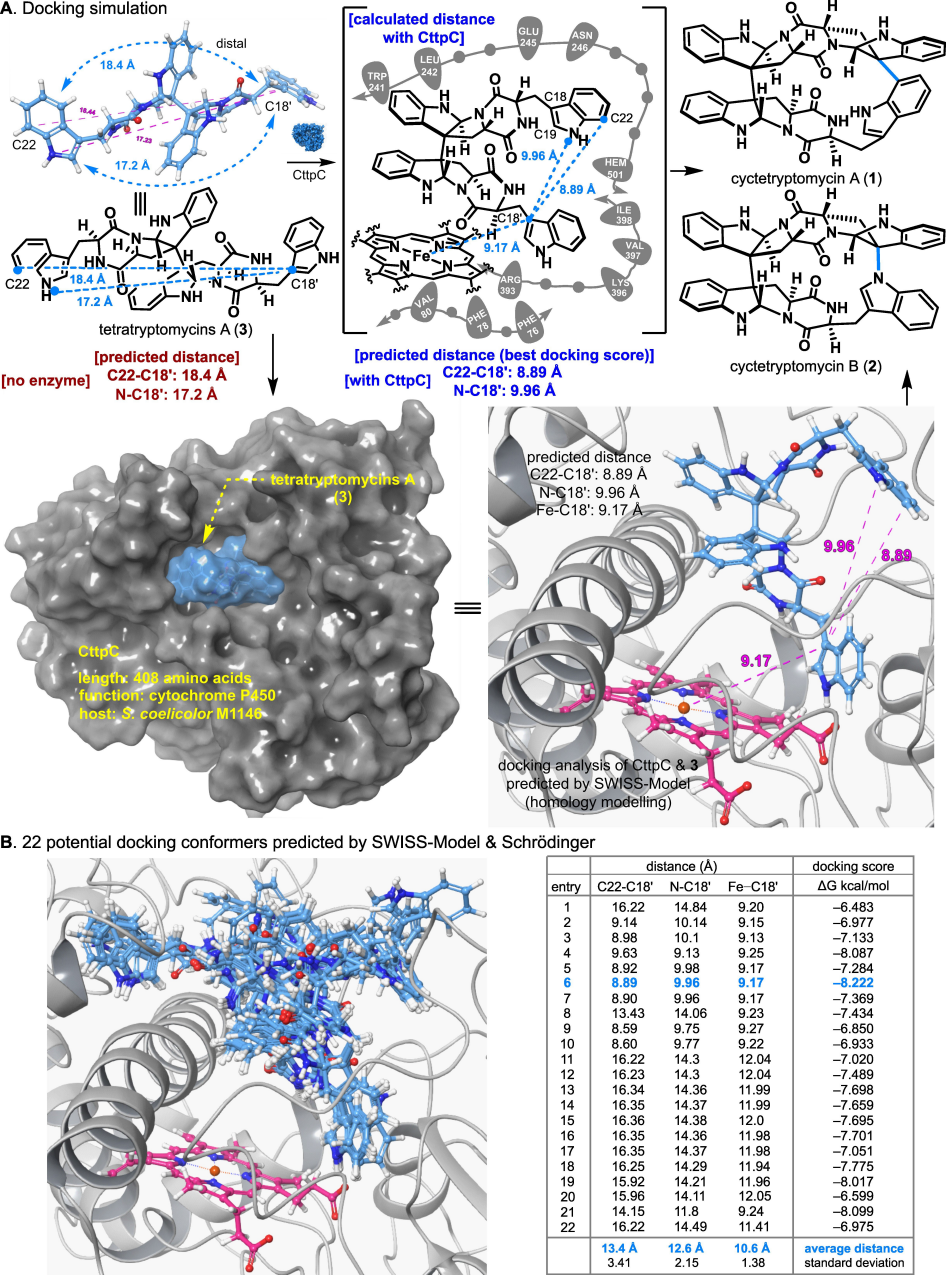
A) Docking simulation. B) 22 potential docking conformers predicted by SWISS‐Model & Schrödinger.

The exact mechanism of CttpC‐mediated oxidative macrocyclization of tetratryptomycin A (**3**) to cyctetryptomycin A and B (**1**–**2**) was previously unclear. Docking simulations between tetratryptomycin A (**3**) and CttpC were therefore conducted to elucidate the detailed mechanism (Figure 5A). Docking simulations using SWISS‐Model showed significant conformational changes in tetratryptomycin A (**3**) in the presence of CttpC. The highest docking score conformation predicted an 8.89 Å distance between C 22 and C 18′ in the presence of CttpC, compared to 18.4 Å without CttpC, indicating dramatic conformational changes induced by CttpC. This docking result suggests that CttpC reduces the C22−C18′ distance by half. Similarly, the N−C18′ distance shortened from 17.2 Å without CttpC to 9.96 Å with it. Interestingly, tetratryptomycin A (**3**) and CttpC′s heme group were closely aligned within the pocket (Fe−C18′ : 9.17 Å).

To rule out random predictions and enhance accuracy, further detailed docking analyses were conducted (Figure 5B). Averaging intermolecular distances across **22** potential docking conformations from the docking simulation of tetratryptomycin A (**3**) with CttpC, the mean distances were C22−C18′ : 13.4 Å, N−C18′ : 12.6 Å, Fe−C18′ : 10.6 Å, with respective standard deviations of 3.41, 2.15, and 1.38. This confirmed that CttpC significantly shortens the average intermolecular distances in tetratryptomycin A (**3**) compared to when it is absent, with relatively low variation.

## Conclusions

This study enabled a practical and reliable synthetic route of tetratryptomycin A (**3**), ditryptophenaline (**38**), and dibrevianamide F (**39**) through the utilization of a newly developed Zr‐catalyzed dimerization method for tryptophan derivatives. Additionally, the detailed mechanism of the oxidative macrocyclization of tetratryptomycin A (**3**) using CttpC was elucidated, facilitating the 8‐step synthesis of cyctetryptomycin A and B (**1**–**2**). The highlights of this research are as follows: (1) This study reported a novel, mild, and safe Zr‐catalyzed dimerization method that is user‐friendly and highly reproducible. The superior role of dppe as an additive was discovered and its mechanism was elucidated. Moreover, this method was shown to be applicable to natural products such as cyctetryptomycin A and B (**1**–**2**), tetratryptomycin A (**3**), ditryptophenaline (**38**), and dibrevianamide F (**39**). (2) For the oxidative macrocyclization of tetratryptomycin A (**3**) using CttpC, optimal large‐scale cultivation conditions for *S. coelicolor M1146* were established. Docking simulations revealed that CttpC induces dramatic conformational changes in tetratryptomycin A (**3**), thus elucidating the mechanism of this intricate oxidative macrocyclization. With the recent advancements in genetic engineering technologies, further optimized artificial proteins related to CttpC are anticipated in the future.

## Conflict of Interests

The authors declare no conflict of interest.

1

## Supporting information

As a service to our authors and readers, this journal provides supporting information supplied by the authors. Such materials are peer reviewed and may be re‐organized for online delivery, but are not copy‐edited or typeset. Technical support issues arising from supporting information (other than missing files) should be addressed to the authors.

Supporting Information

## Data Availability

The data that support the findings of this study are available in the supplementary material of this article.
