# Supplementary material for: Concise Synthesis of Cyctetryptomycin A and B Enabled by Zr‐Catalyzed Dimerization
Source: Angew Chem Int Ed Engl. 2024 Nov 6;64(2):e202414295. doi: 10.1002/anie.202414295 (PMC11720396; doi:10.1002/anie.202414295)
Supplement: Supplementary file 1 — Supporting Information [file ANIE-64-e202414295-s001.pdf]

Angewandte Chemie
Eine Zeitschrift der Gesellschaft Deutscher Chemiker

Supporting Information

Concise Synthesis of Cyctetryptomycin A and B Enabled by Zr-Catalyzed Dimerization

L. Yu, H. Ogawa, S. Li, T. Lam Cheung, W. Liu, D. Yan, Y. Matsuda, Y. Kobayashi, Z. Guo, K. Ikeda, T. A. Hamlin, K. Yamazaki, P.-Y. Qian*, H. Nakamura**

SUPPLEMENTARY INFORMATION

Concise Synthesis of Cyctetryptomycin A and B Enabled by Zr-catalyzed Dimerization

Longhui Yu, †,[a] Hiroshige Ogawa, †,[a] Shangzhao Li, †,[a] Tsoh Lam Cheung, †,[a] Wenchao Liu, [a,b] Dexiu Yan, [c] Yudai Matsuda, [c] Yusuke Kobayashi, [d] Zhihong Guo, [a] Kotaro Ikeda, [a] Trevor A. Hamlin, [e] Ken Yamazaki, *[f] Pei-Yuan Qian, *[a,b] Hugh Nakamura*[a]

[a] Dr. L. Yu, H. Ogawa S. Li, T.-L. Cheung, W. Liu, Prof. Dr. Z. Guo, K. Ikeda, Prof. Dr. P.-Y. Qian, and Prof. Dr. H. Nakamura
The Hong Kong University of Science and Technology (HKUST), Clear Water Bay, Kowloon, Hong Kong SAR, China

[b] W. Liu and Prof. Dr. P.-Y. Qian.
Department Southern Marine Science and Engineering Guangdong Lab (Guangzhou), Nansha, Guangzhou, China

[c] D. Yan and Prof. Dr. Y. Matsuda.
City University of Hong Kong, Tat Chee Avenue, Kowloon, Hong Kong SAR, China

[d] Prof. Dr. Y. Kobayashi
Kyoto Pharmaceutical University, 5 Nakauchi-cho, Misasagi, Yamashina-ku, Kyoto 607-8414, Japan

[e] Prof. Dr. T. A. Hamlin
Vrije Universiteit Amsterdam, De Boelelaan 1108, 1081 HZ Amsterdam, The Netherlands.

[f] Prof. Dr. K. Yamazaki
Okayama University, Tsushimanaka, Okayama 700-8530, Japan

[†] These authors contributed equally to this work.

General Experimental

Reagents were purchased from commercial sources (Bide, Energy, TCI, and Sigma-Aldrich) and used without further purification, unless otherwise stated. Yields refer to chromatographically unless otherwise stated. Reactions were monitored by LC/MS, and thin layer chromatography (TLC). TLC was performed using 0.2-0.25 mm silica plates, and using short-wave UV light as the visualizing agent, and phosphomolybdic acid and $Ce(SO_4)_2$, or $KMnO_4$ and heat as developing agents. NMR Spectrum were recorded on Bruker AVII 400, and JEOL 600 instruments and are calibrated using residual undeuterated solvent ($CHCl_3$ at 7.26 ppm ^{1}H NMR, 77.16 ppm ^{13}C NMR). The following abbreviations were used to explain multiplicities: s = singlet, d = doublet, t = triplet, q = quartet, m = multiplet, br = broad. Column chromatography was performed using 230-400 mesh silica gel, and PTLC was performed using 0.2-0.25 mm silica plates. High-resolution mass Spectrum (HRMS) were recorded on an Agilent LC/MSD TOF mass spectrometer by electrospray ionization time of flight reflectron experiments.

Comparisons of bispyrrolidinoindoline dioxopiperazine in total synthesis enable by C3-C3 coupling.

Synthesis	Steps	Overall Yield	Scale of final product	Highlights	Skeletal Disconnection
Movassaghi (2008)[a]	6	9.7%<br>7.3%	90.2 mg<br>26.3 mg	-Divergent synthesis<br>-Stoichiometric CoCl(PPh)$_3$ for dimerization<br>-Low overall yield	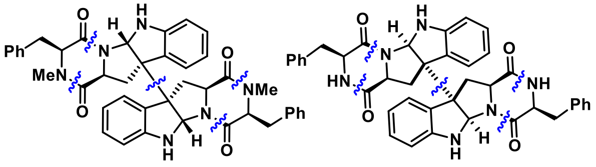<br>(-)-Ditryptophenaline (+)-WIN 64821
Ishikawa (2013)[b]	3	9.2%,<br>20%	100 mg<br>65.2 mg	-Divergent synthesis<br>-Inexpensive reagent<br>-Short-step synthesis<br>-Stoichiometric V_2O_5 for dimerization	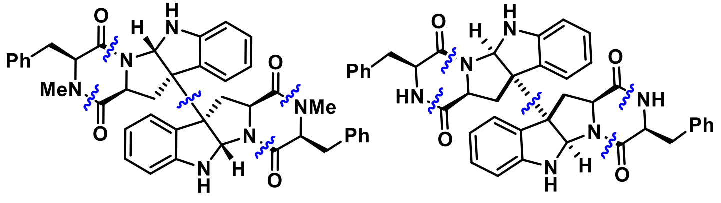<br>(-)-Ditryptophenaline (+)-WIN 64821
Oguri (2014)[c]	4	7.3%	33.2 mg	-Catalytic NiI_2•$6H_2O$ for dimerization<br>-Short-step synthesis	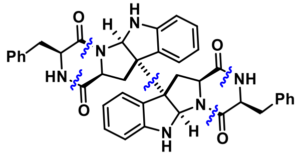<br>(+)-WIN 64821
Xia (2015)[d]	4	26%<br>21%	~50 mg	-Divergent synthesis<br>-Stoichiometric $CuCl_2$ for dimerization, low selectivity<br>-Short synthetic steps	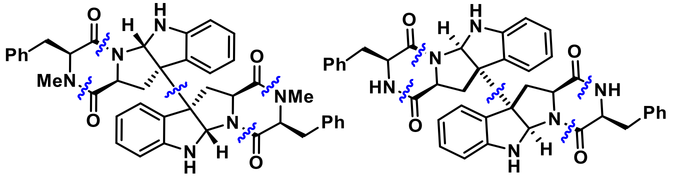<br>(-)-Ditryptophenaline (+)-WIN 64821
Álvarez & de Lera (2021)[e]	6	15%<br>23%<br>26%	11.3 mg<br>5.2 mg<br>17.5 mg	-Divergent synthesis<br>-Stoichiometric CoCl(PPh)$_3$ for dimerization of tertiary bromide<br>-High overall yield	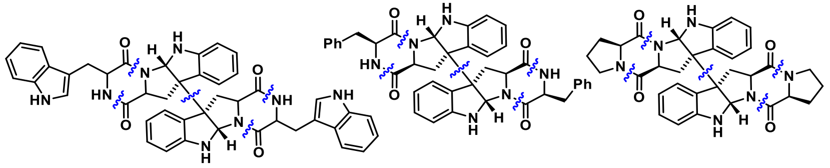<br>(-)-Tetratryptomycin A (-)-SF-5280-451 (-)-Dibrevianamide F

Comparison of representative C3-C3 dimerization methods for tryptophan and tryptamine derivatives.

Reporter	Yield	Scale of product	Highlights	Detail Disconnection
Movassaghi (2008)[a]	~50%	12.3 mg	-Pre-activation of indole scaffold is needed<br>-Stoichiometric reagent of $CoCl(PPh)_3$<br>-High selectivity	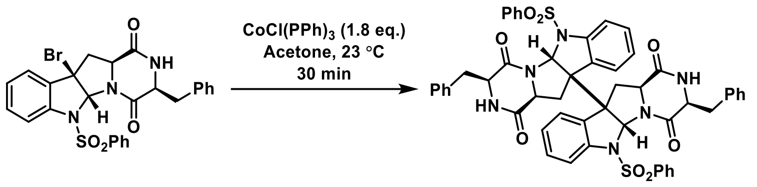
Ishikawa (2013)[b]	28%	13.0 mg	-direct dimerization<br>-no activation is required<br>-short-step<br>-nearly stoichiometric reagent of V_2O_5	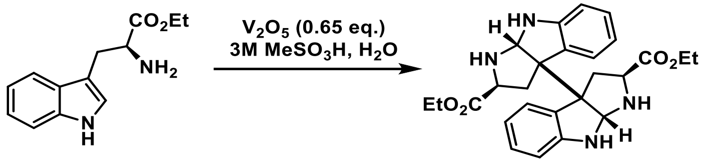
Oguri (2014)[c]	70%	259 mg	-Pre-activation of indole scaffold is needed<br>-Catalytic NiI_2•$6H_2O$ coupling reaction<br>-High selectivity	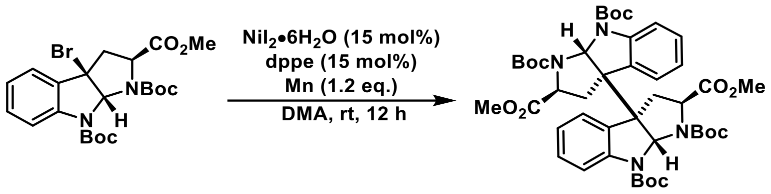
Xia (2015)[d]	35%	709 mg	-Direct activation of indole scaffold<br>-Stoichiometric reagent of $CuCl_2$<br>-Low selectivity	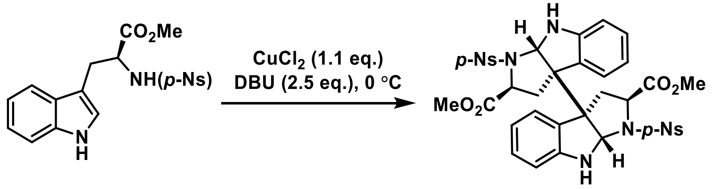
de Lera (2009)[f]	~25%	40 mg	-Pre-activation of indole scaffold is needed<br>-Stoichiometric reagent Bu_6Sn_2<br>-High selectivity	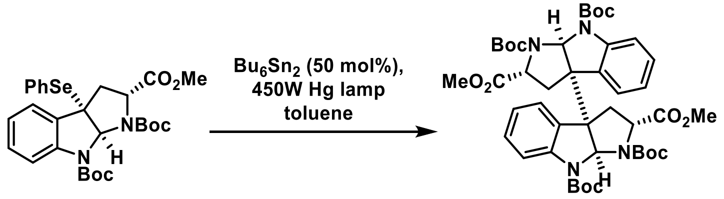

Hall (1964)[g]	20%	No data	-Direct activation of indole scaffold<br>-Stoichiometric reagent of $FeCl_3$<br>-Strong reagent is needed (MeMgI)	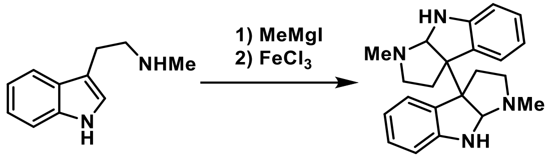
Takayama (2002)[h]	13%	No data	-Direct activation of indole scaffold<br>-Stoichiometric and Strong reagent of PIFA	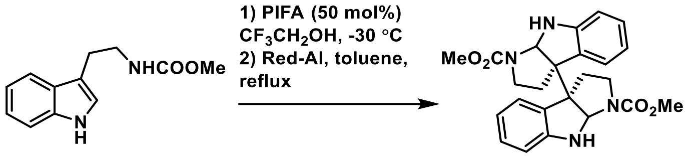
Ueda & Tokuyama (2023)[i]	56% (C3-C3 coupling)	27.7 mg	-direct dimerization<br>-no activation is required<br>-short-step<br>-non-toxic metal	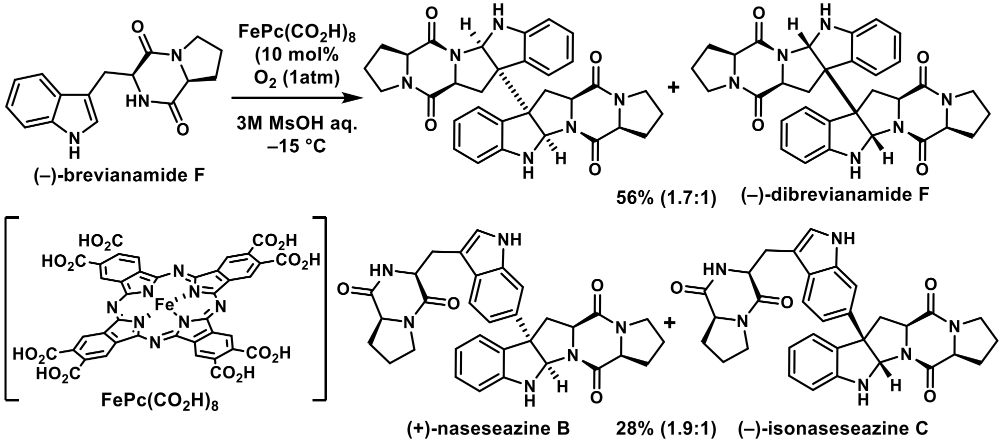

(a) M. Movassaghi, M. A. Schmidt, J. A. Ashenhurst, *Angew. Chem. Int. Ed.* **2008**, *47*, 1485–1487. (b) S. Tadano, Y. Mukaeda, H. Ishikawa, *Angew. Chem. Int. Ed.* **2013**, *52*, 7990-7994. (c) M. Wada, T. Murata, H. Oikawa, H. Oguri, *Org. Biomol. Chem*., **2014**, *12*, 298-306. (d) K.-J. Liang, X. Deng, X.-G. Tong, D.-S. Li, M. Ding, A.-K. Zhou, C.-F. Xia, *Org. Lett.* **2015**, *17*, 206-209. (e) Areal, M. Domínguez, P. Vendrig, S. Alvarez, R. Álvarez, Á. R. de Lera, *J. Nat. Prod.* **2021**, *84*, 1725-1737. (f) C. Peŕez-Balado, P. Rodríguez-Graña, Á. R. de Lera, *Chem. Eur. J.* **2009**, *15*, 9928-9937. (g) A. I. Scott, F. Mccapra, E. S. Hall, *J. Am. Chem. Soc.* **1964**, *86*, 302-303. (h) H.; Ishikawa, H. Takayama, N. Aimi, *Tetrahedron Lett.* **2002**, *43*, 5637-5639. (i) H. Ueda, S. Sato, K. Noda, H. Hakamata, E. Kwon, N. Kobayashi, H. Tokuyama, *Angew. Chem. Int. Ed.* **2023,** *62*, e202302404.

Reported total Synthesis of the bispyrrolidinoindoline dioxopiperazines natural products

Movassaghi’s total synthesis of (+)-WIN 64821 and (−)-Ditryptophenaline. *Angew. Chem. Int. Ed.* **2008**, *47*, 1485.

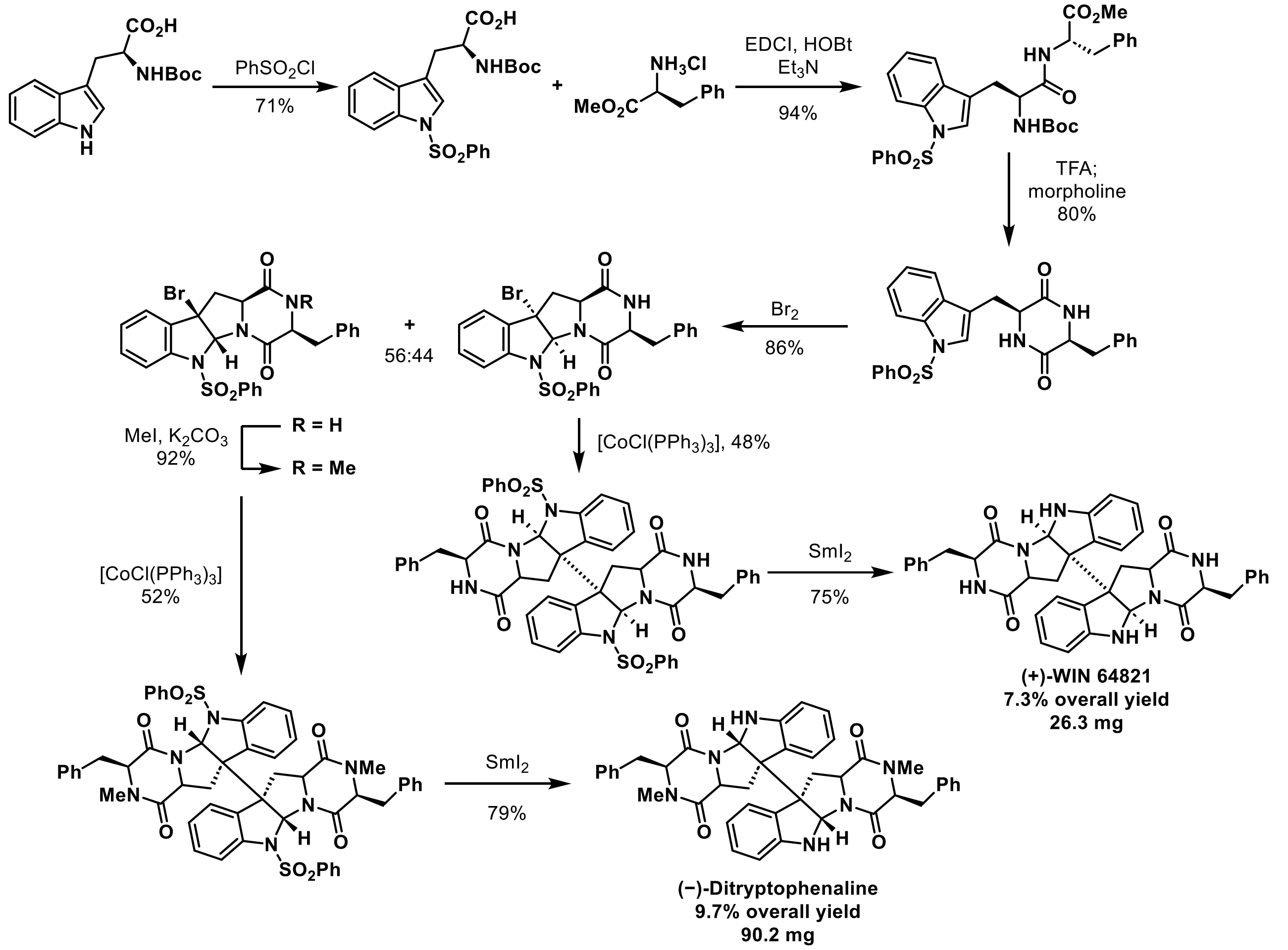

Ishikawa's total syntheses of (+)-WIN 64821 and (−)-Ditryptophenaline. *Angew. Chem. Int. Ed.* **2013**, *52*, 7990.

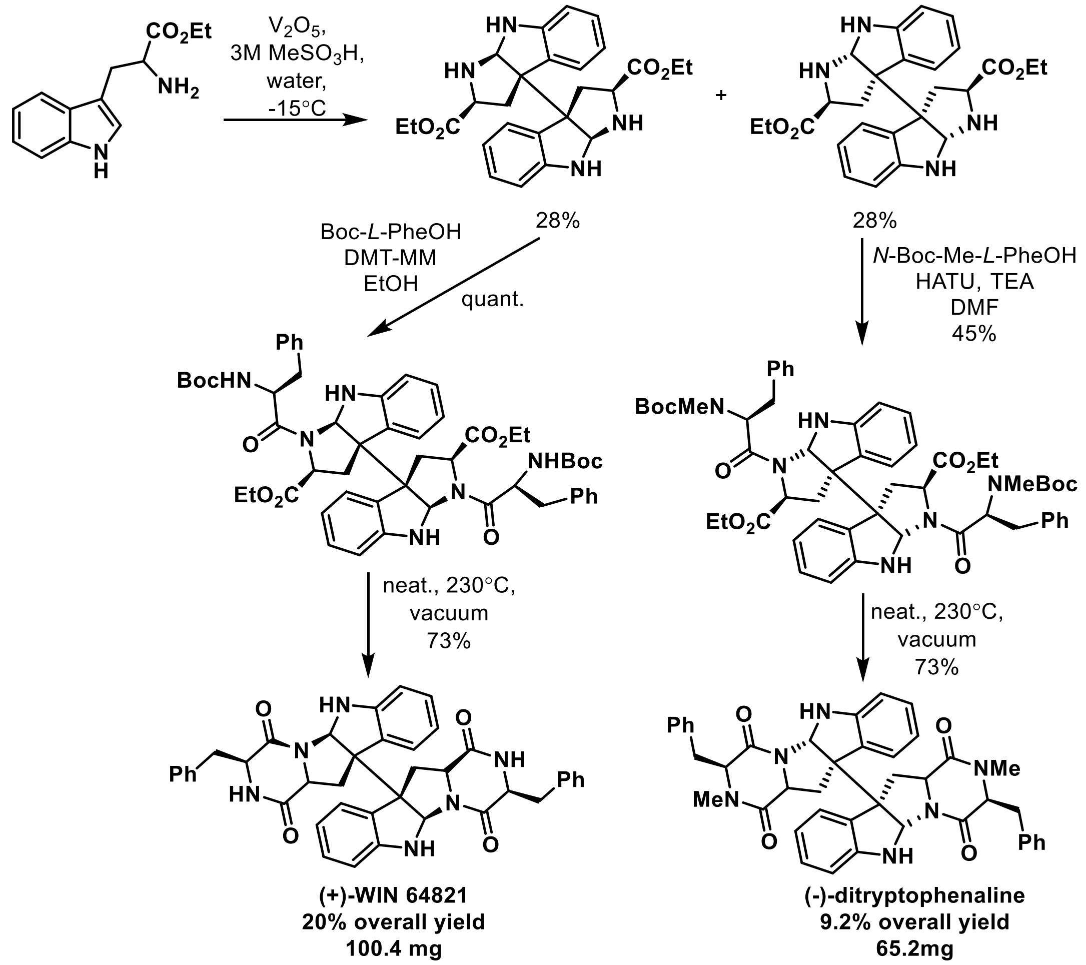

Oguri's total synthesis of (+)-WIN 64821. *Org. Biomol. Chem.* **2014**, *12*, 298.

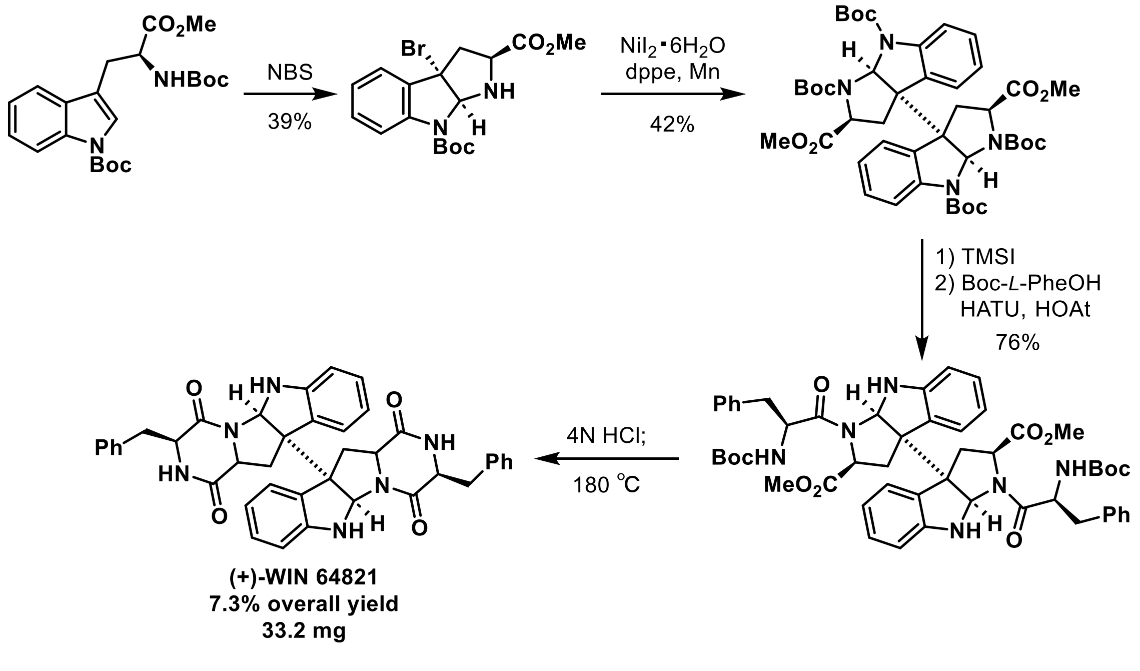

Xia's total synthesis of (+)-WIN 64821 and (−)-Ditryptophenaline. *Org. Lett.* **2015**, *17*, 206.

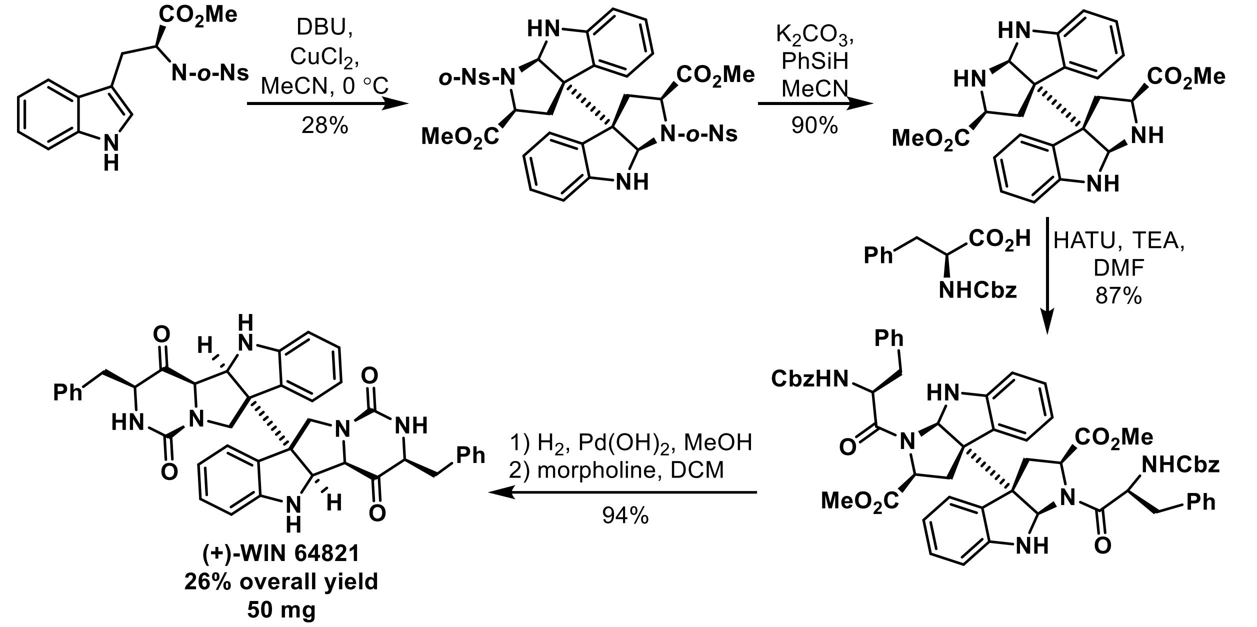

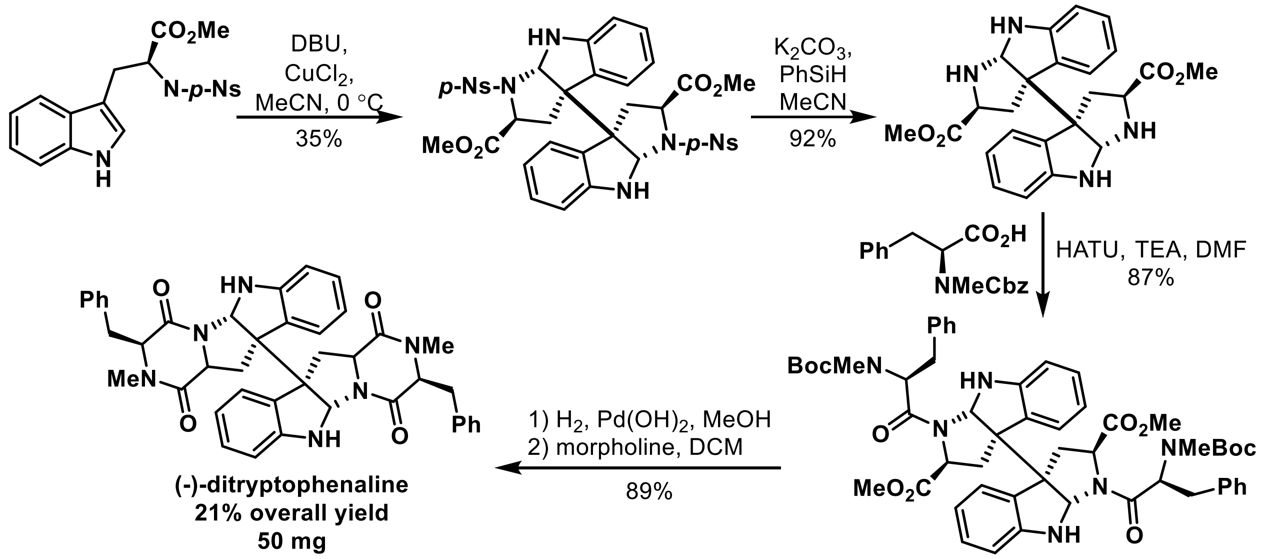

de Lera's total synthesis of Bispyrrolidinoindoline Dioxopiperazine. *J. Nat. Prod.* **2021**, *84*, 1725.

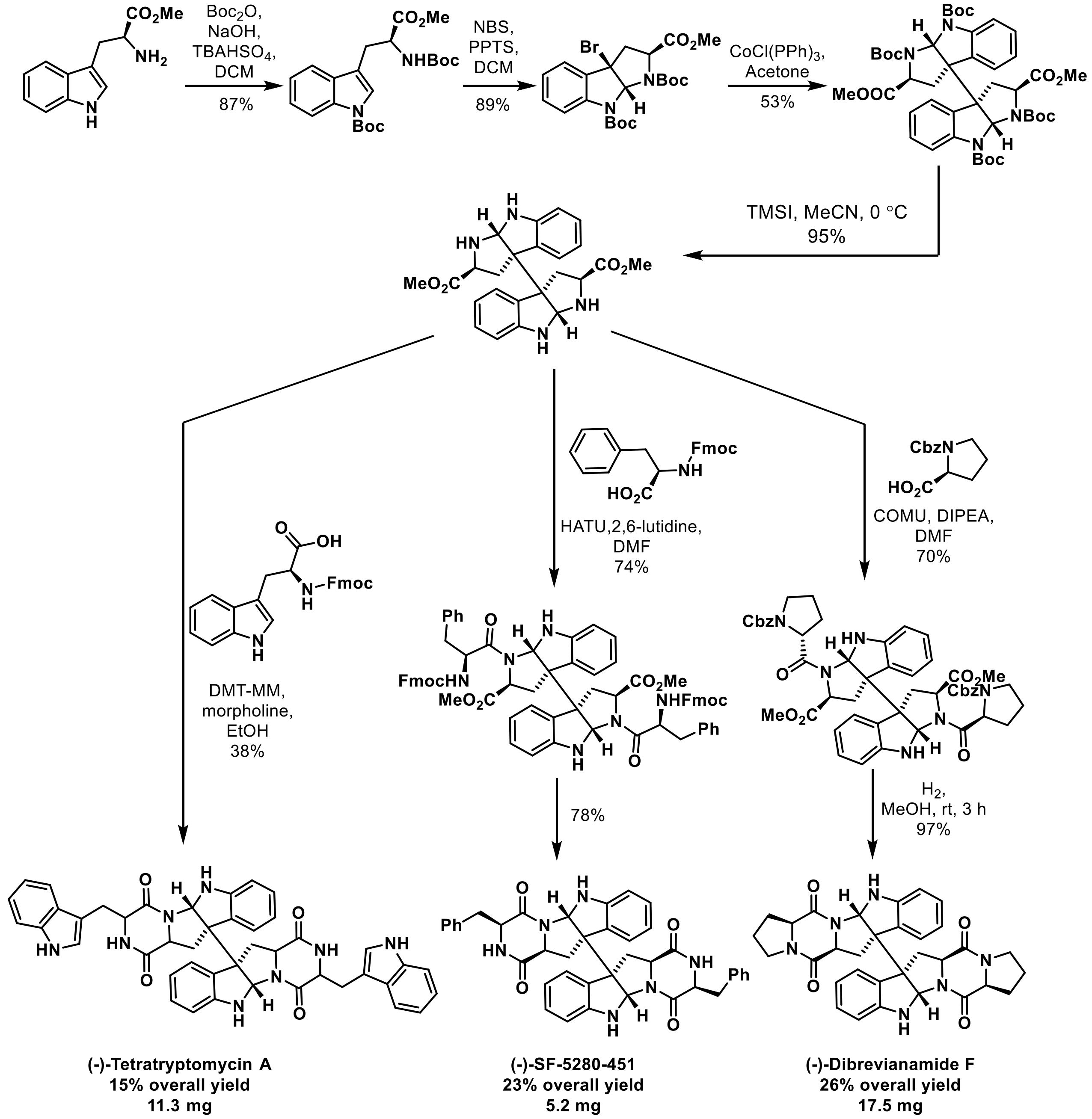

Ueda and Tokuyama's semisynthesis of (–)-dibrevianamide F, (+)-naseseazine B, and (–)-isonaseseazine C. *Angew. Chem. Int. Ed.* **2023**, *62*, e202302404.

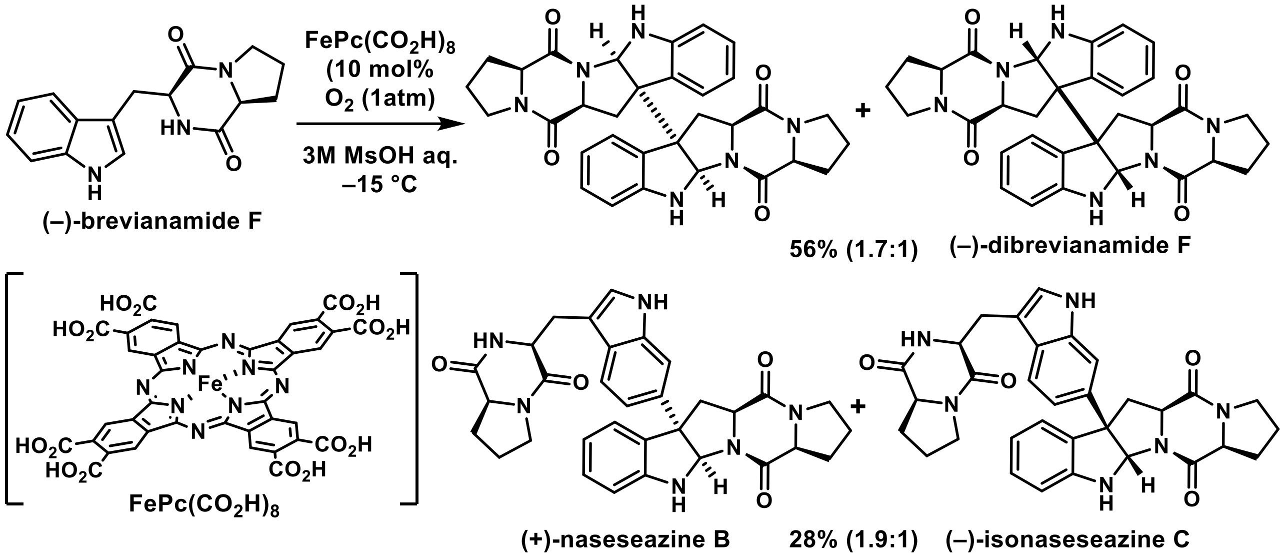

Synthetic Route

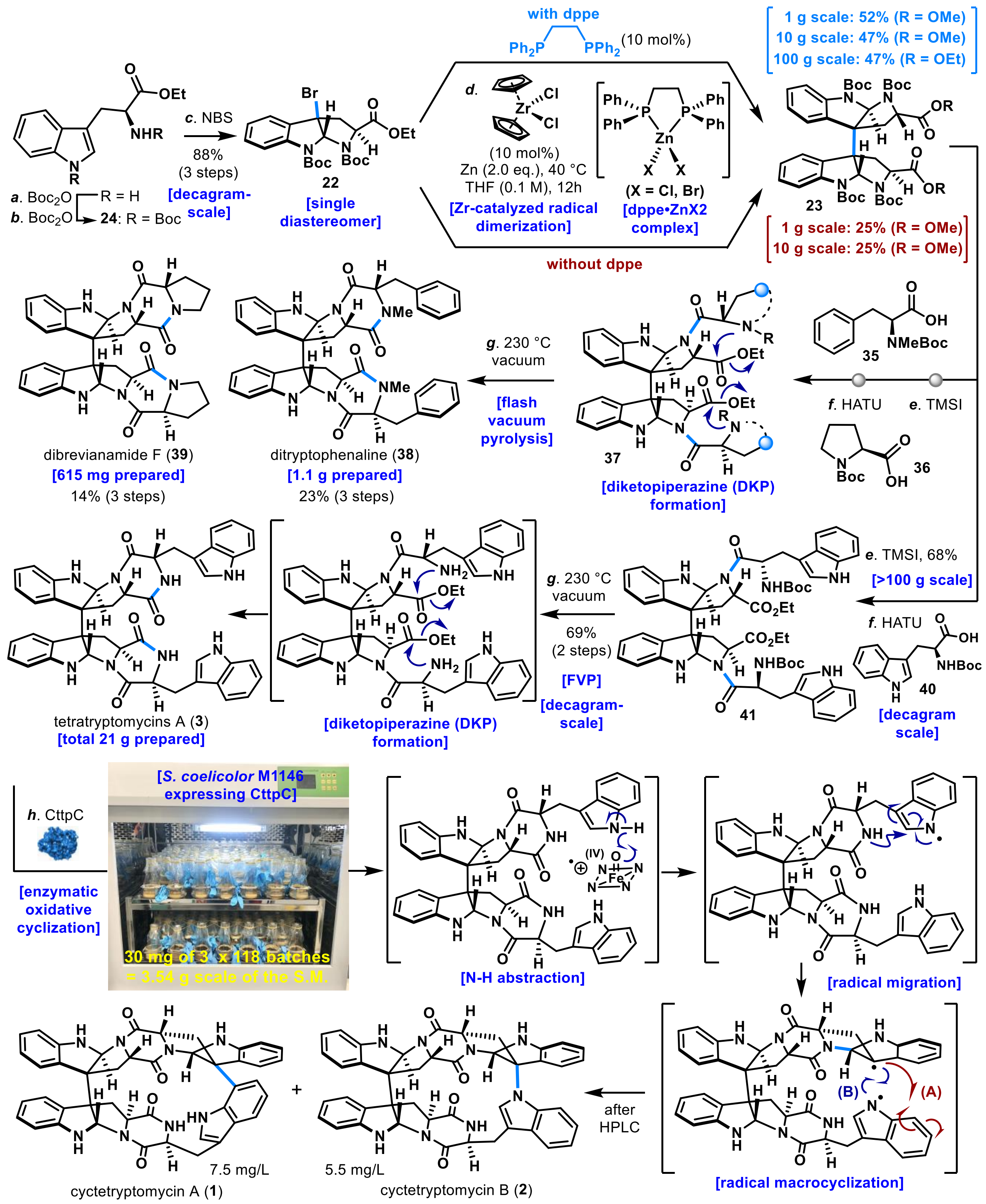

Optimization of Zr-catalyzed dimerization

Table S1 Representative dimerization method

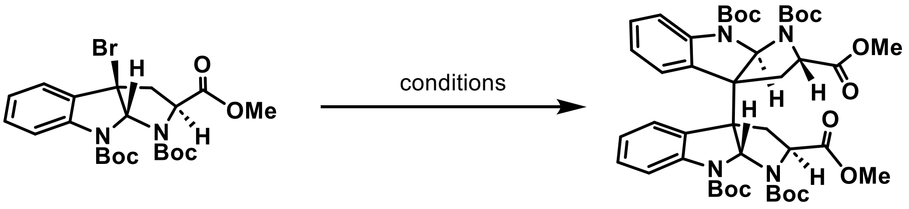

entry[a]	solvent	yields[b]
1	NiI_2 (cat.), phen (cat.)<br>Zn, DMA, 25 °C	38%
2	Cp_2TiCl_2 (cat.)<br>Zn, THF, 40 °C	35%
3	$(PPh_3)_3CoCl$ (1.8 eq.)<br>acetone, 25 °C	24%
4	$NiI_2 \cdot 6H_2O$ (cat.), dppe (cat.)<br>Mn, DMA, 25 °C	<5%

a 50 mg scale, b isolated yields

Table S2 Optimization of solvents

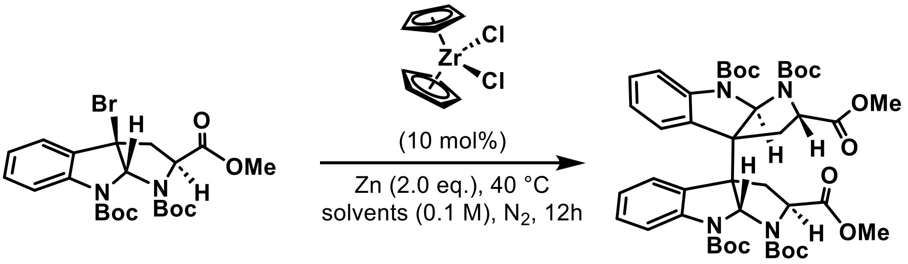

entry[a]	solvent	yields[b]
1	THF	65%
2	DME	37%
3	MeCN	19%
4	2-Me-THF	<5%
5	TBME	<5%
6	NMP	<5%
7	hexane	<5%
8	EtOAc	<5%
9	DMA	<5%
10	DMF	<5%
11	1,4-dioxane	<5%

a 50 mg scale, b isolated yields

Table S3 Control experiments and the others

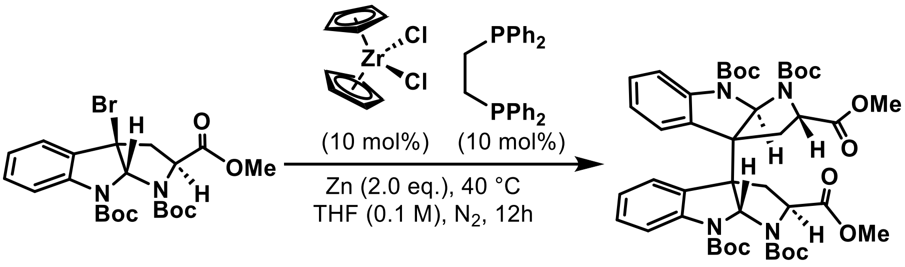

entry[a]	deviations	yields[b]
1	50 mg scale	65%
2	1 g scale	52%
3	10 g scale	47%
4	1 g scale, w/o dppe	25%
5	10 g scale, w/o dppe	25%
6	without [Zr]	19%
7	without Zn^0	0%
8	without dppe	23%
9	exposed to air	trace
10	2.0 eq. H_2O added	54%
11	0.2 M in THF	54%
12	60 °C instead of 40 °C	41%
13	rt instead of 40 °C	52%

a 50 mg scale, b isolated yields

Mechanistic Study

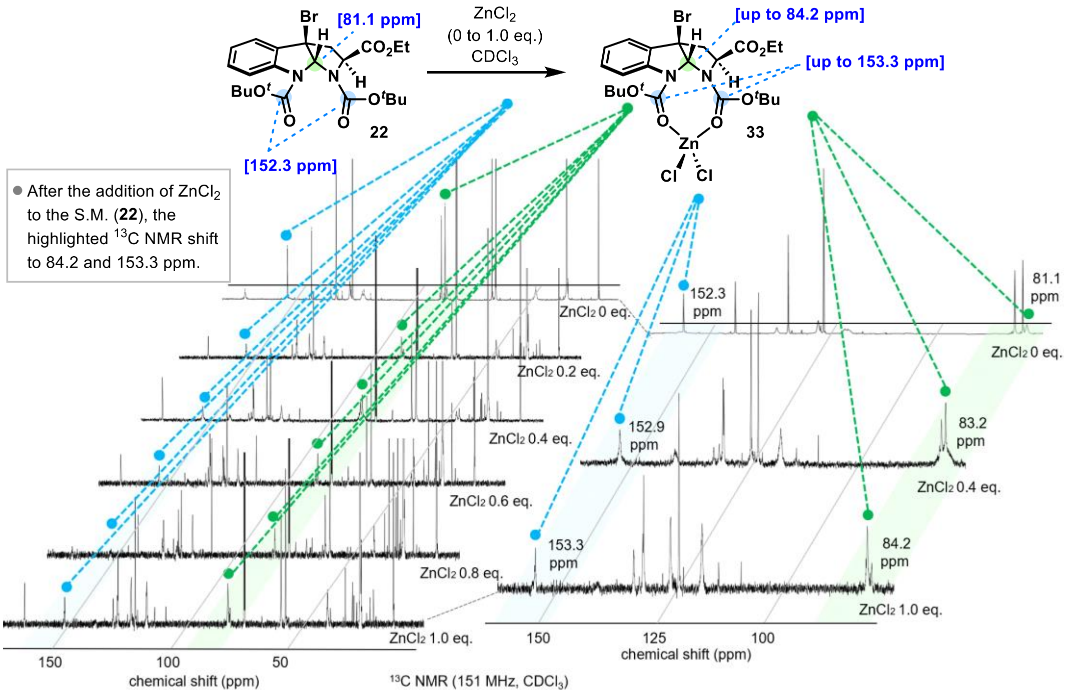

Figure S1 ^{13}C NMR titration experiment

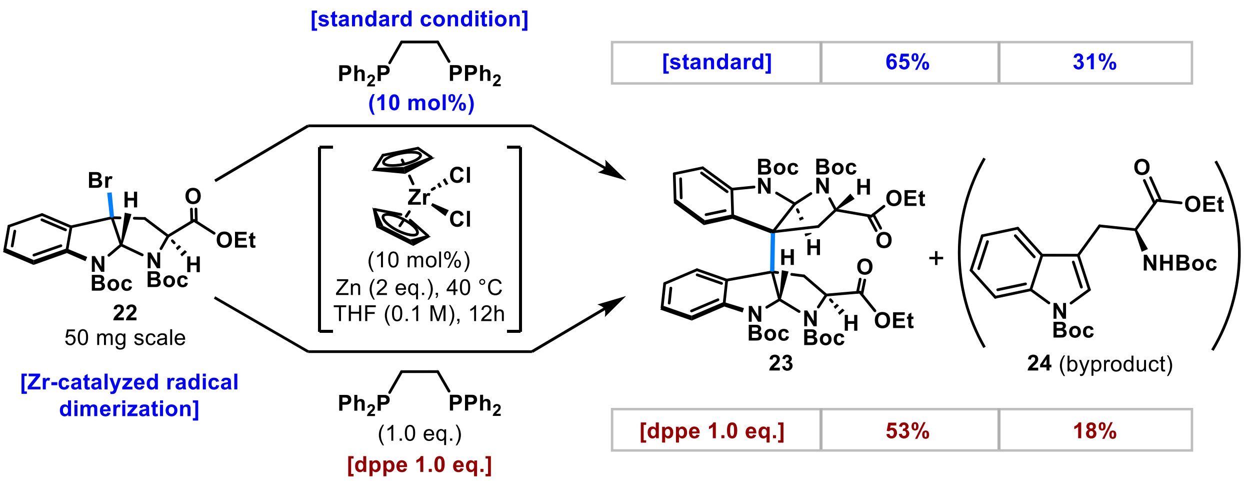

Figure S2 Effect of excessive dppe

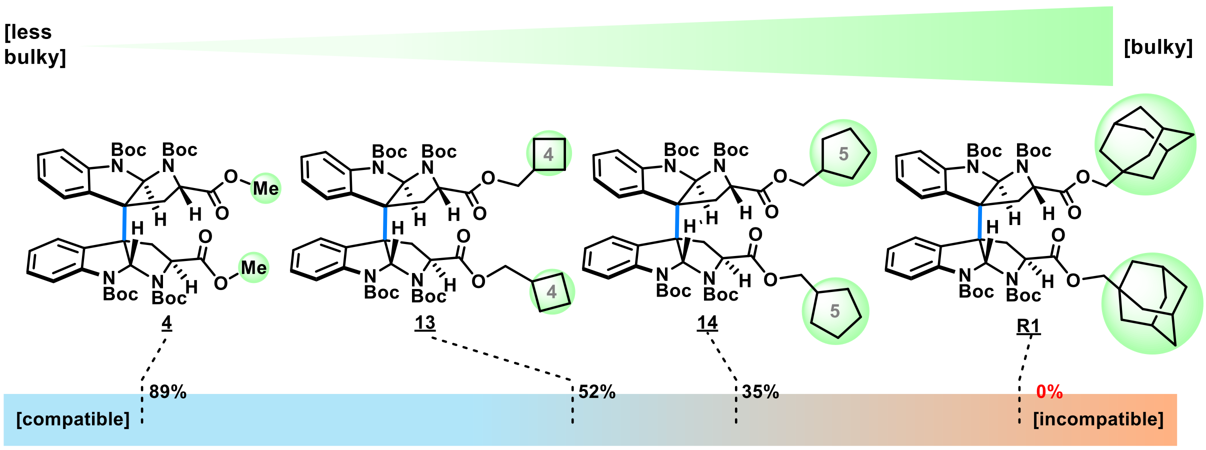

Figure S3 Effect of the steric hindrance

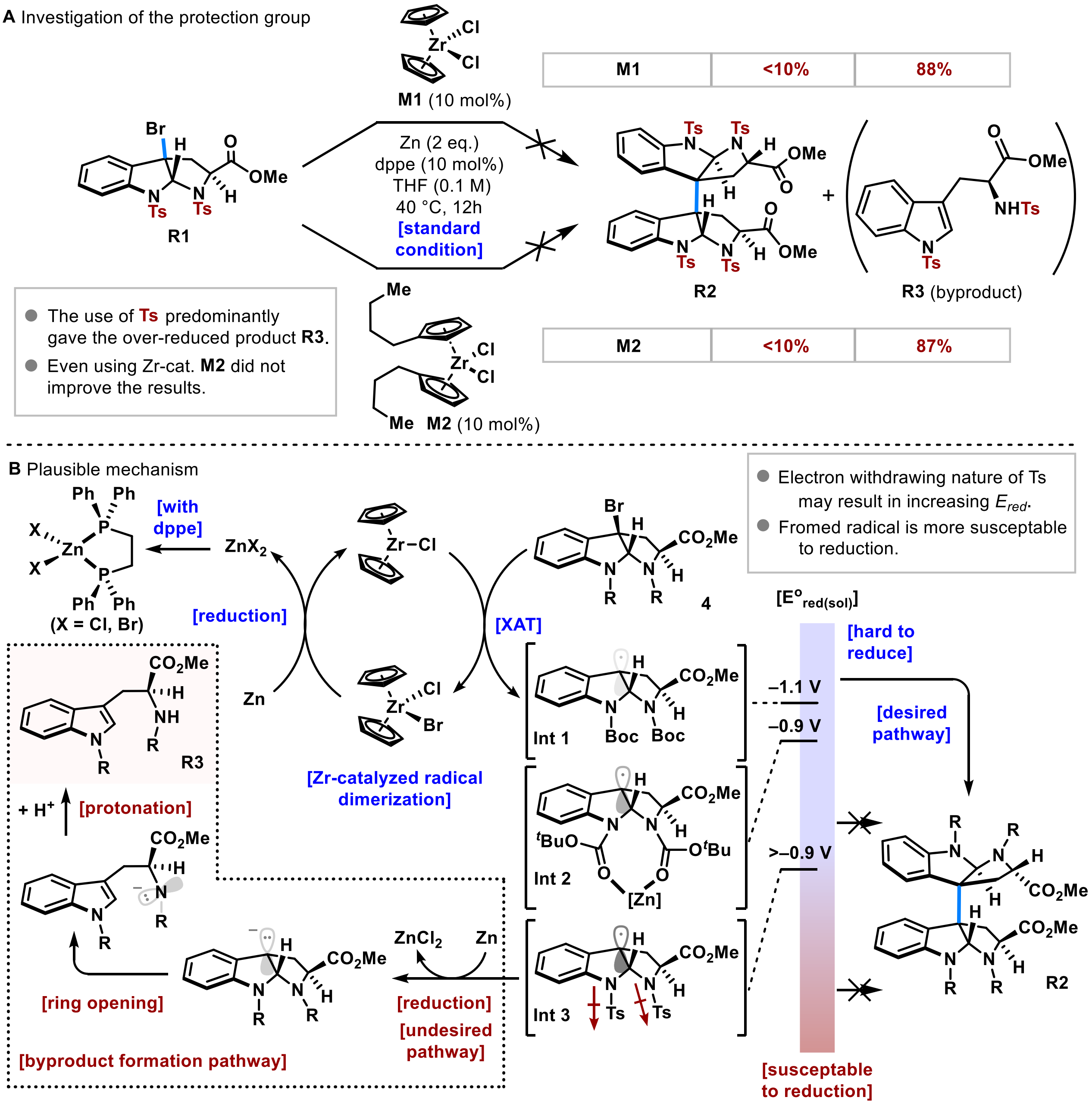

Figure S4 Optimization of the protecting group

Graphical guide for the Zr-catalyzed dimerization.

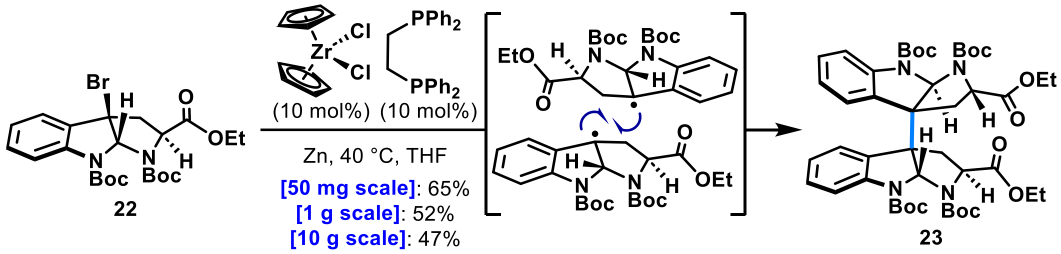

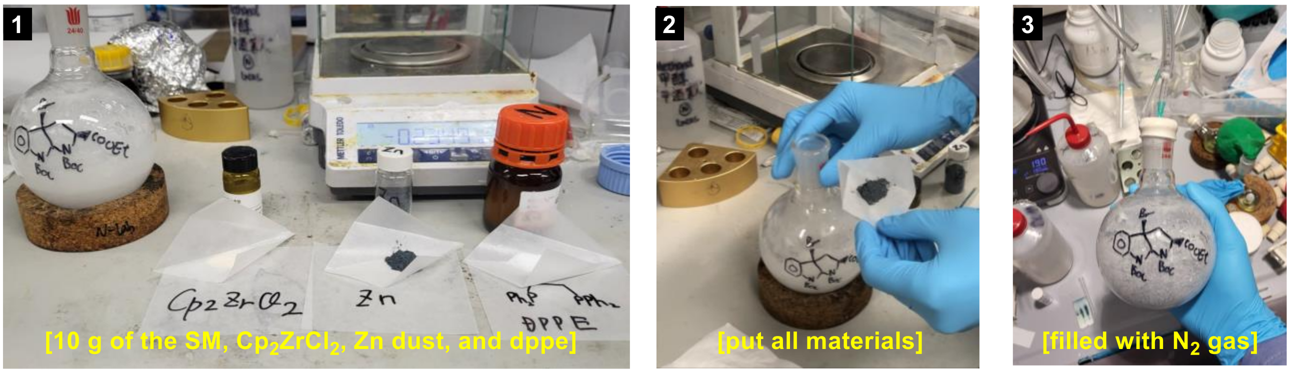

1: Weight all the material (10 g of **22**, Cp_2ZrCl_2, Zn dust, and dppe).

2: Put all material to the round-bottom flask.

3: Seal the flask with plastic septum and parafilm, then remove air and fill with N_2 gas (x3).

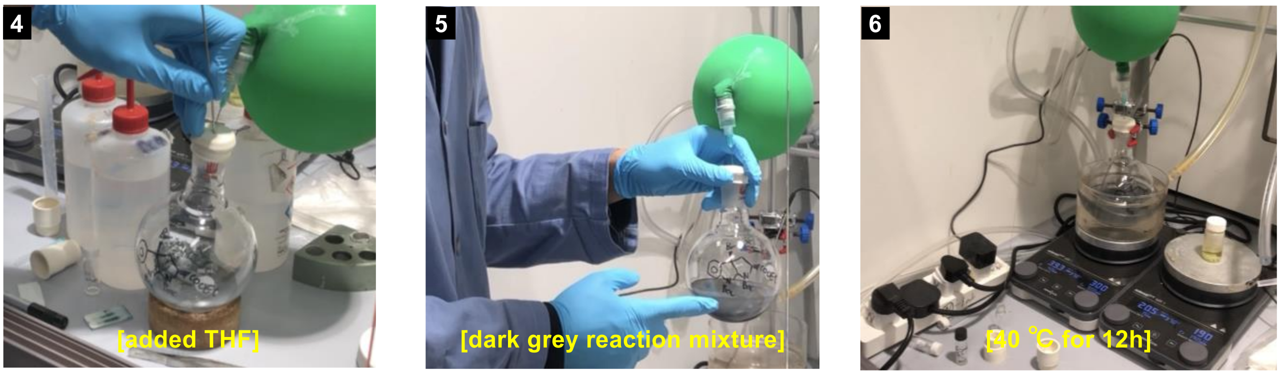

4: Inject THF with needle.

5: Dark grey reaction mixture obtained.

6: Put the flask to 40 °C oil-bath for 12 h reaction.

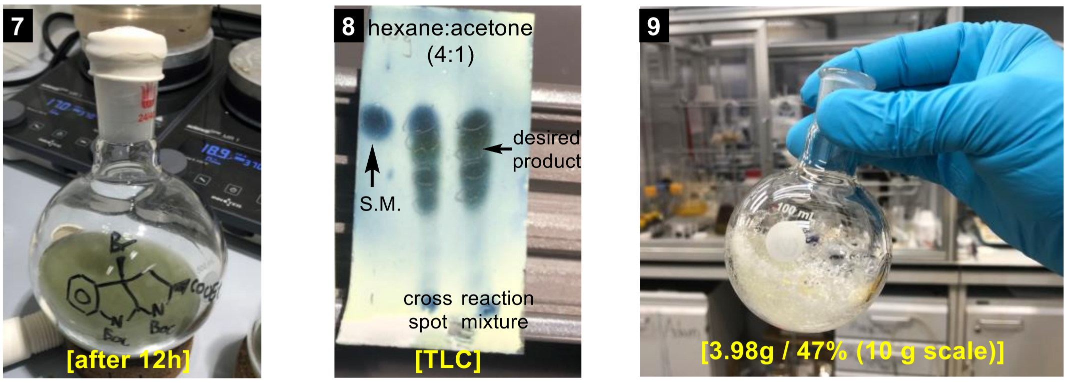

7: Green reaction mixture obtained after 12 h.

8: TLC of the reaction using hexane:acetone (4:1) as eluent.

9: After the purification, 3.98 g/47% (10 g scale) of the desired product **23** was obtained.

Graphical guide for culture preparation of *S. coelicolor* M1146 containing the plasmids *cttpC* (pIZ11) in AM6 medium.

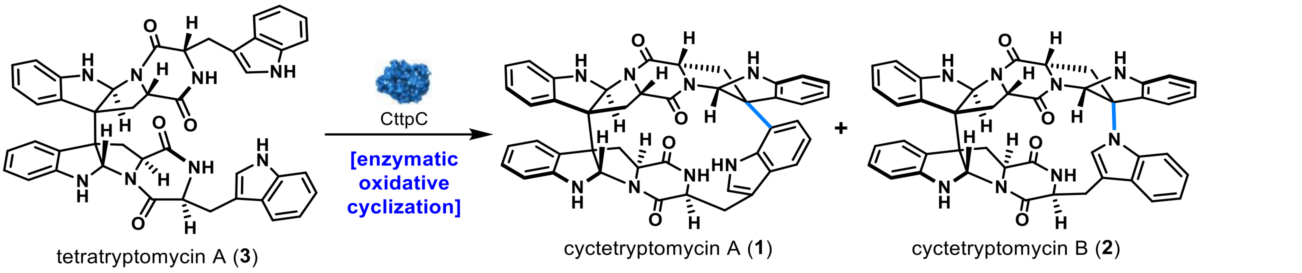

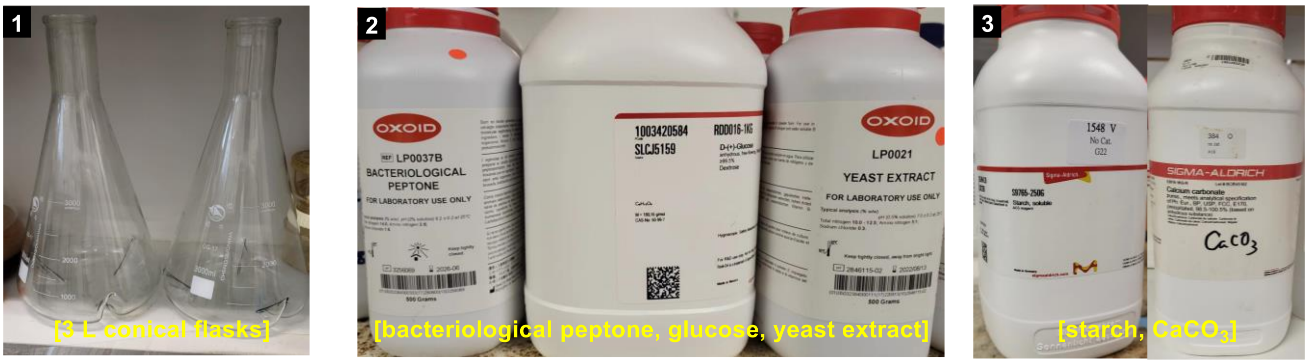

1: A 3 L conical flask was used for the preparation of AM6 medium.

2: The ingredients of medium, glucose (10 g/L), bacteriological peptone (5 g/L), yeast extract (5 g/L).

3: Soluble starch (20 g/L), calcium carbonate (5 g/L).

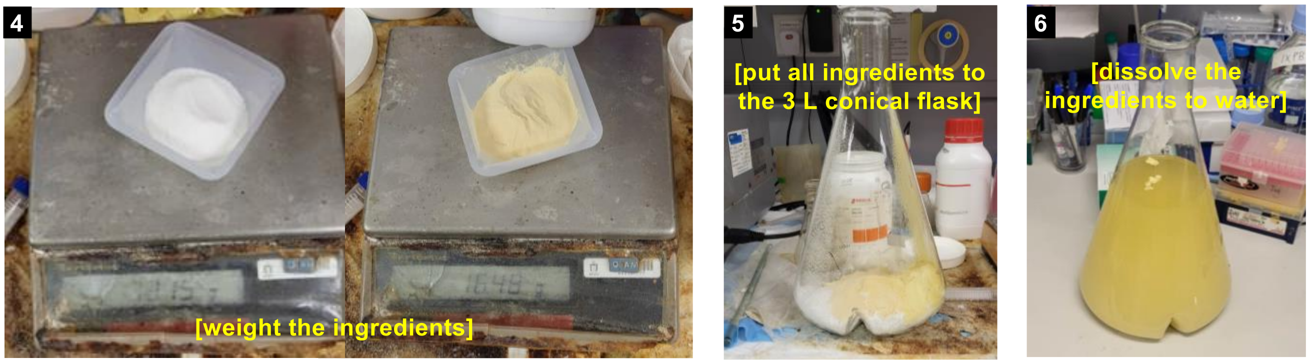

4: Weight the ingredients.

5: Put all ingredients to the conical flask.

6: Dissolve the ingredients to water.

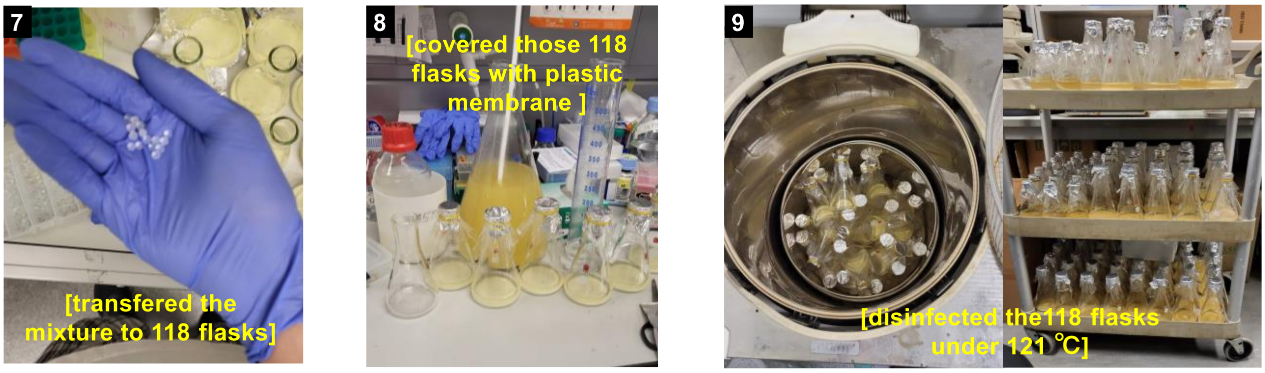

7: Transfer and distribute the mixture to 118 smaller flasks, then put glass beans to those flasks.

8: Cover those 118 flasks with plastic membrane and aluminum foil, then wrap with rubber band.

9: Sterilize all 118 flasks under 121 °C.

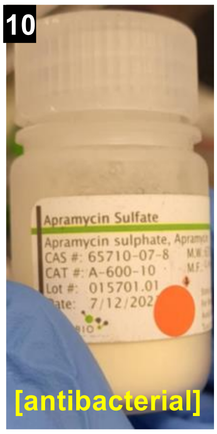

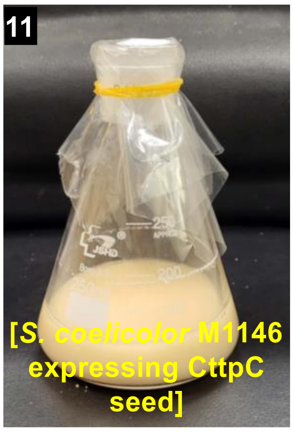

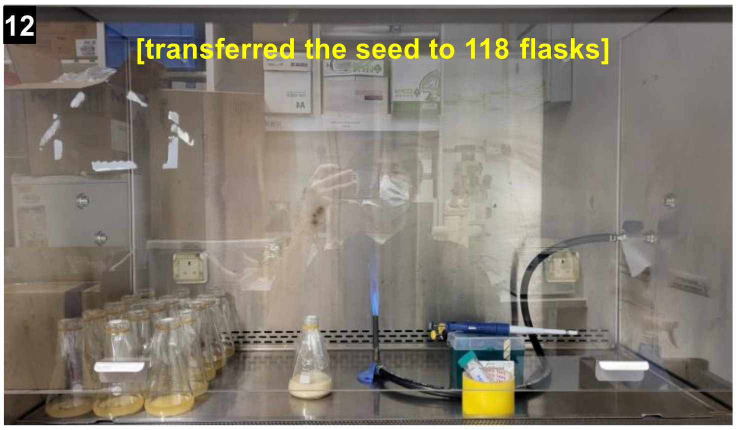

10: Apramycin sulfate was used as an antibacterial.

11: [*S. coelicolor* M1146 expressing CttpC] seed solution.

12: Transferred the bacterial seed (3 v/v%) and the antibacterial to all the flasks (118 flasks).

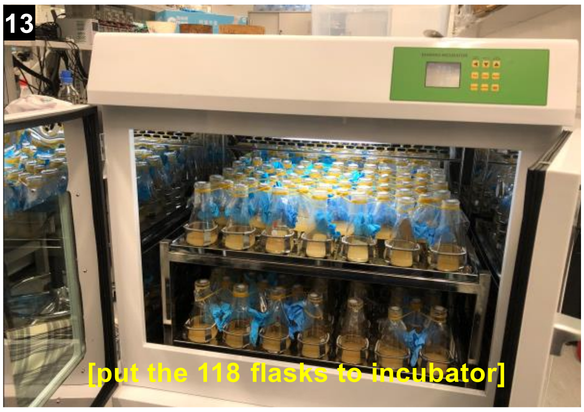

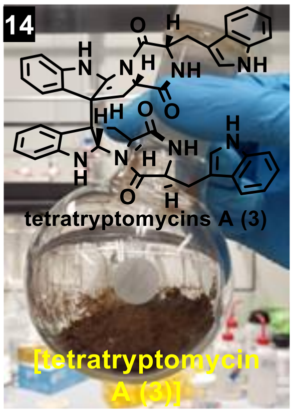

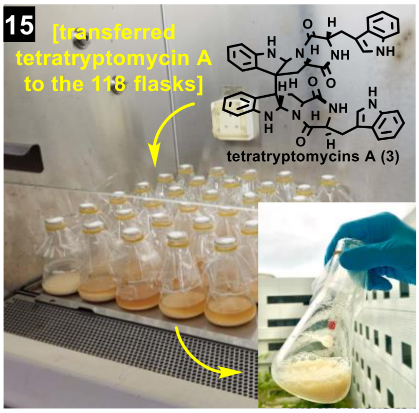

13: Put the 118 flasks to incubator with 28-30 °C, 220 rpm for 3 days to grow up *S. coelicolor* M1146.

14: Prepared 3.54 g of tetratryptomycin A (**3**).

15: Transferred tetratryptomycin A (**3**) to the 118 flasks and incubate 2-3 weeks.

Scale: 3.54 g of tetratryptomycin A (**3**) (= 30 mg×118 batches). Volume: 50 ml x 118 flasks = 5.9 L

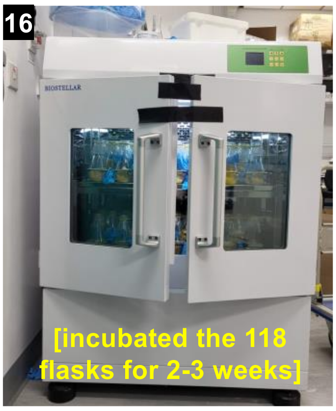

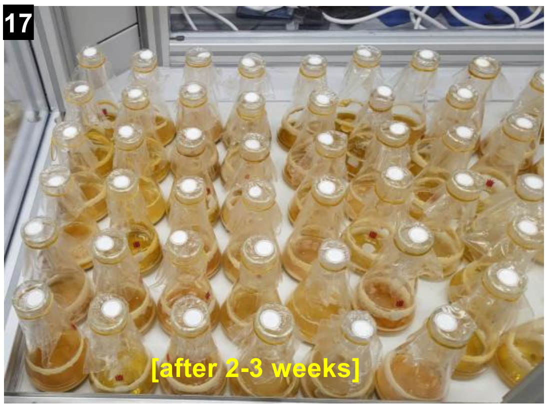

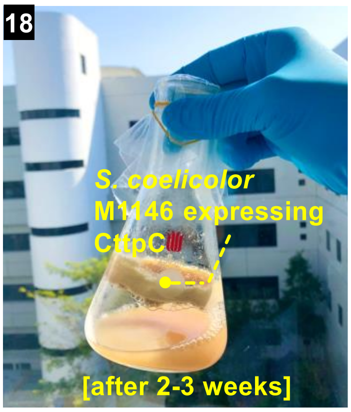

16: Incubated the 118 flasks of *S. coelicolor* M1146 expressing CttpC with tetratryptomycin A (**3**).

17: Took out all flaks after 2-3 weeks.

18: Observed cell density of *S. coelicolor* M1146 expressing CttpC (solids agglomerated on the flask surface).

19

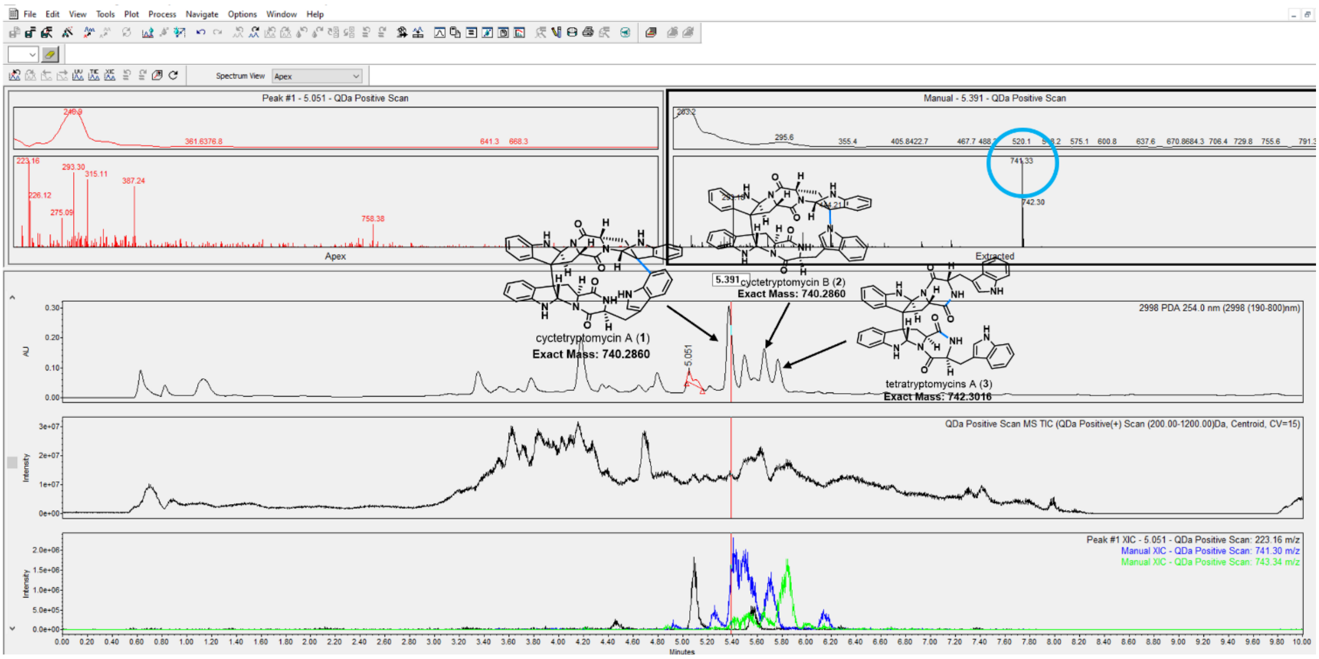

19: LC-MS monitoring chromatogram of the crude mixture after 2-3 weeks. Cyctetryptomycin A (**1**) (exact Mass: 740.2860) and cyctetryptomycin B (**2**) (exact Mass: 740.2860), and the S.M. tetratryptomycins A (**3**) (exact Mass: 742.3016) were observed.

20

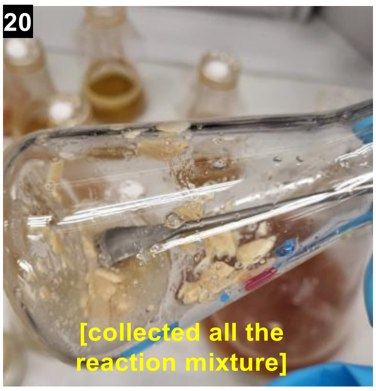

21

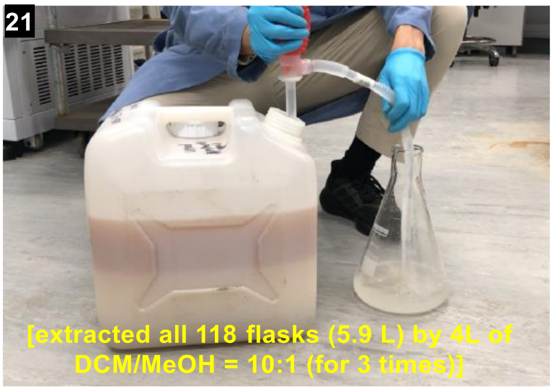

22

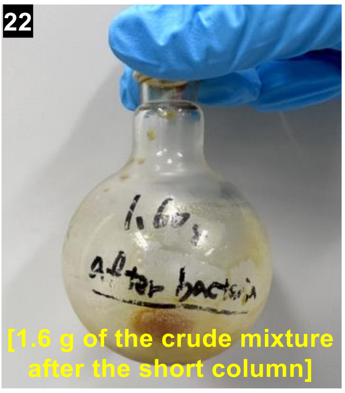

20: Collected all the reaction mixture and filter the solid (washed by DCM:MeOH = 10:1).
21: Extracted the aqueous liquid with DCM:MeOH (10:1) using 20 L plastic container.
22: Concentrated the organic layer, then purified by short column chromatography. 1.6 g of crude mixture was obtained after the short column chromatography. Then, purified the crude mixture of cyctetryptomin A (**1**), cyctetryptomin B (**2**), and the S.M. tetratryptomycins A (**3**) by prep HPLC for a couple of times.

Initial attempt for the macrocyclization of tetratryptomycin A (3).

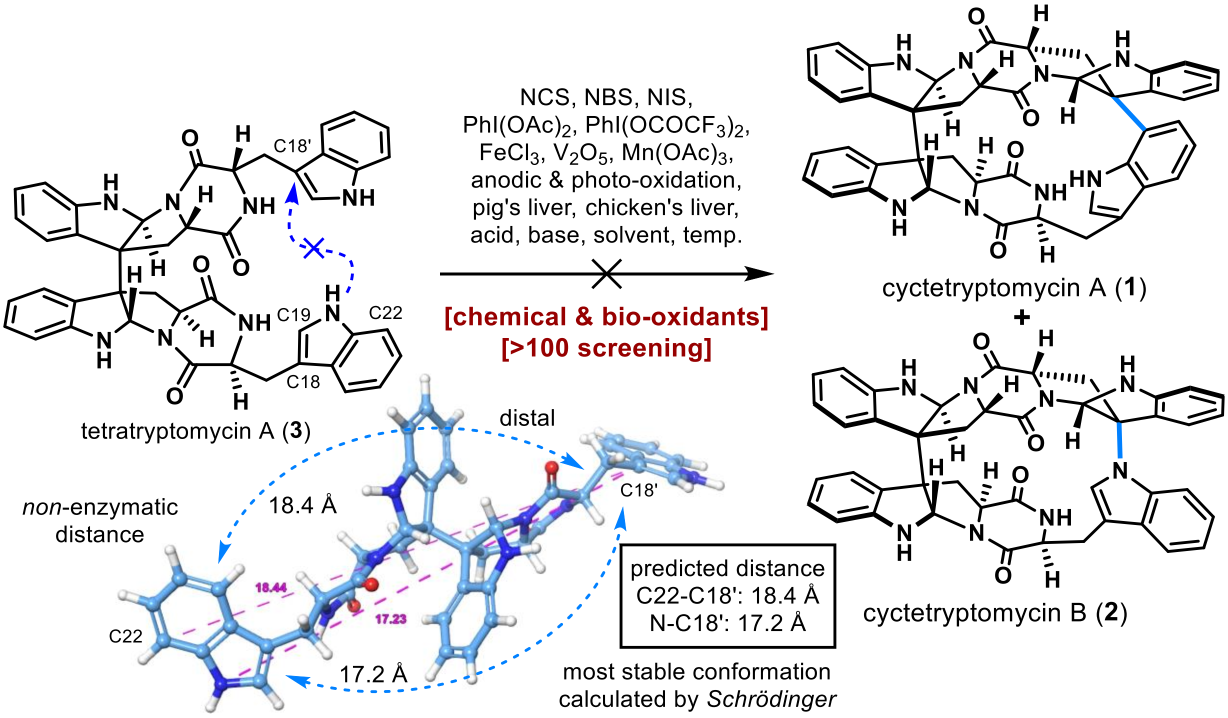

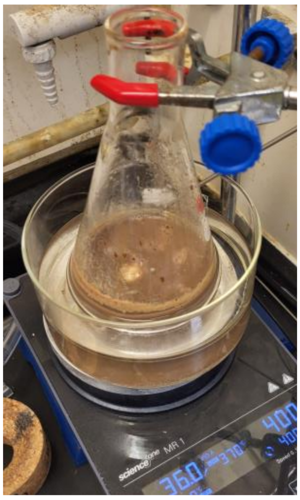

[fresh pig's liver]

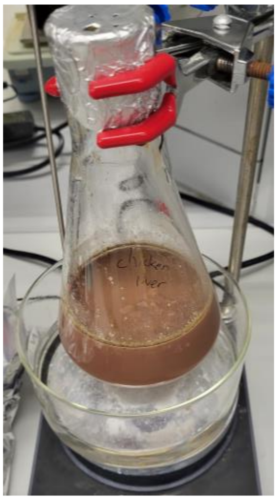

[fresh chicken's liver]

purchased from local supermarket in Hong Kong

Optimization of the macrocyclization of tetratryptomycin A (3).

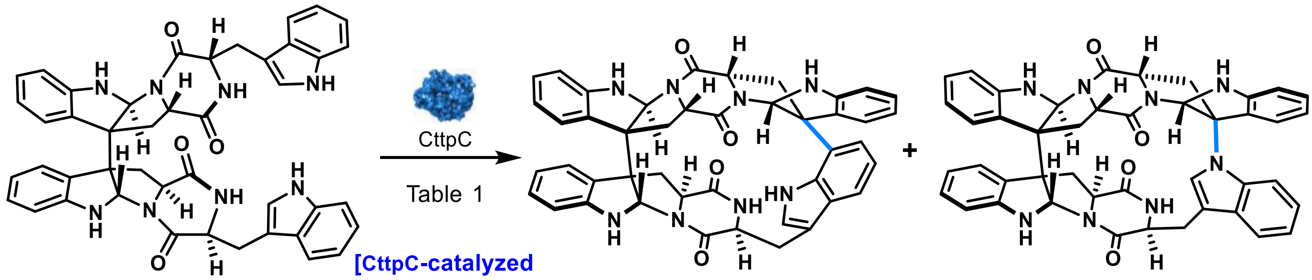

tetratryptomycin A (**3**) [CttpC-catalyzed macrocyclization] cyctetryptomycin A (**1**) + cyctetryptomycin B (**2**)

Table 1

Entry	Conditions	Results
1	20 mg of **3**, 1L bacteria solution in 3L flask	no reaction, (14 days)
2	100 mg of **3**, 1L bacteria solution in 3L flask	no reaction, (14 days)
3	500 mg of **3**, 1L bacteria solution in 3L flask	no reaction, (14 days)
4	1 g of **3**, 1L bacteria solution in 3L flask	no reaction, (14 days)
5	2 g of **3**, 1L bacteria solution in 3L flask	no reaction, (14 days)
6	2.5 g of **3**, 1L bacteria solution in 3L flask	no reaction, (14 days)
7	3 g of **3**, 1L bacteria solution in 3L flask	no reaction, (14 days)
8	5 mg of **3**, 50 ml bacteria solution in 250 ml flask	ca. 60% conversion (20 days)
9	10 mg of **3**, 50 ml bacteria solution in 125 ml flask	ca. 80-90% conversion (17 days)
10	15 mg of **3**, 50 ml bacteria solution in 125 ml flask	ca. 80-90% conversion (17 days)
11	20 mg of **3**, 50 ml bacteria solution in 125 ml flask	ca. >600% conversion (17 days)
**12**	**30 mg of 3, 50 ml bacteria solution in 125 ml flask**	**ca. 60% conversion (17 days)**
13	40 mg of **3**, 50 ml bacteria solution in 125 ml flask	ca. 20-30% conversion (17 days)
14	50 mg of **3**, 50 ml bacteria solution in 125 ml flask	ca. 20-30% conversion (17 days)
15	40 mg of **3**, 100 ml bacteria solution in 250 ml flask	didn't work well (17 days)
16	60 mg of **3**, 100 ml bacteria solution in 250 ml flask	didn't work well (17 days)
17	100 mg of **3**, 100 ml bacteria solution in 250 ml flask	didn't work well (17 days)
18	200 mg of **3**, 100 ml bacteria solution in 250 ml flask	didn't work well (17 days)

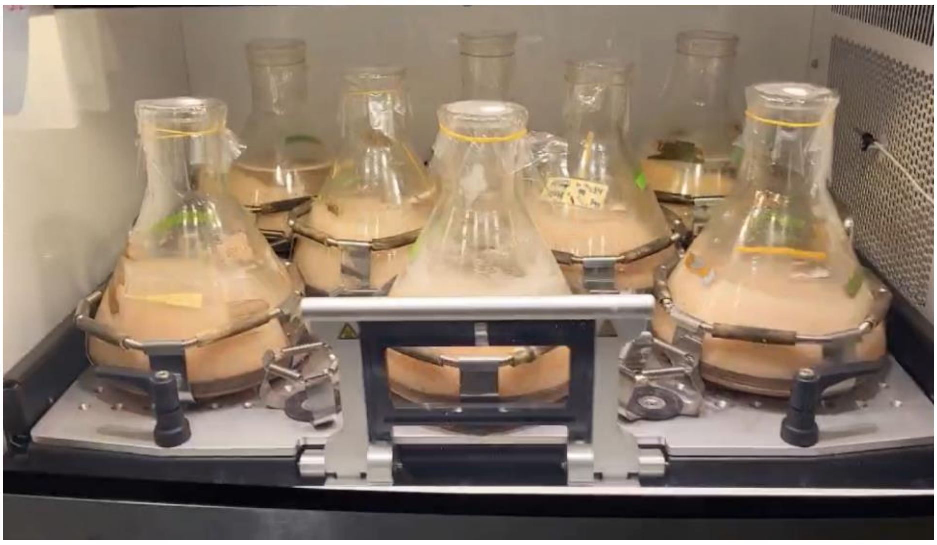

[Failed *S. coelicolor* M1146 expressing CttpC]
1L reaction mixture in 3L flask

Synthetic Procedures and Characterization Data

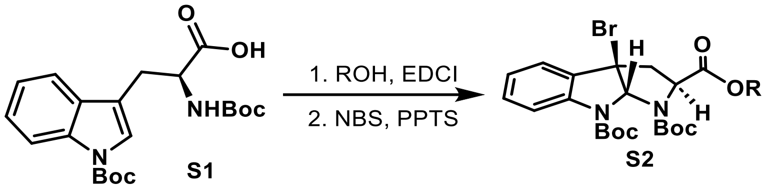

[General Procedure A]

Step 1: *N*,1-Bis[(1,1-dimethylethoxy)carbonyl]-*L*-tryptophan (1.0 eq.), alcohol (1.1 eq.), DMAP (0.1 eq.) were dissolved in DCM (0.2 M). To this solution, 1-Ethyl-3-(3-dimethylaminopropyl) carbodiimide (1.1 eq.) was then added, and the reaction was allowed to stir until the acid was consumed. Upon completion of the reaction, the reaction was quenched by saturated *aq.* NH_4Cl, extracted with EtOAc three times. The organic layers were combined and washed with saturated aq. $NaHCO_3$. The combined organic layers were dried over Na_2SO_4 and concentrated under reduced pressure to give the crude ester. The crude ester was used directly in the next step without further purification.

Step 2: The crude and pyridinium *p*-toluenesulfonate (1.0 eq.) were dissolved in DCM (0.25 M) in dark. *N*-Bromosuccinimide (1.0 eq.) was added into the reaction mixture portionwise (20 portions, 10 min/portion) and stirred at room temperature. After the addition of all portions, and all starting material consumed according to the TLC, saturated $Na_2S_2O_3$ solution was added into the reaction mixture, and extracted with DCM (500 mL x3). The combined organic layer was dried over Na_2SO_4 and concentrated under reduced pressure. The crude residue was purified by column chromatography to afford bromo cyclized compound **7**.

*Note: To obtain a single diastereomer **7**, the portionwise of NBS is essential.*

Compound S3

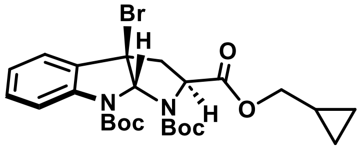

S3

On 3.75 mmol scale, **General Procedure A** was followed with cyclopropanemethanol. Purification by standard procedure afforded the title compound **S3** (1.35 g, 67%).

Physical State: white solid

^{1}H NMR (600 MHz, $CDCl_3$): δ 8.11 – 7.41 (m, 1H), 7.38 (d, *J* = 7.6 Hz, 1H), 7.32 (t, *J* = 7.8 Hz, 1H), 7.13 (t, *J* = 7.5 Hz, 1H), 6.41 (s, 1H), 4.02 – 3.93 (m, 2H), 3.91 (dd, *J* = 10.2, 6.4 Hz, 1H), 3.25 (dd, *J* = 12.7, 6.4 Hz, 1H), 2.83 (dd, *J* = 12.7, 10.2 Hz, 1H), 1.59 (s, 9H), 1.42 (s, 9H), 1.18 – 1.08 (m, 1H), 0.58 (t, *J* = 7.9, 4.1 Hz, 2H), 0.29 (dt, *J* = 5.1 Hz, 2H).

^{13}C NMR (151 MHz, $CDCl_3$): δ 171.2, 152.3, 141.6, 133.0, 130.7, 124.5, 123.4, 119.1, 83.9, 82.3, 81.5, 70.4, 59.9, 59.7, 42.1, 28.3, 9.7, 3.4, 3.3.

HRMS (ESI-TOF): calculated for $C_{12}H_{33}BrN_2NaO_6$ $[M+Na]^+$: 559.1420, found: 559.1421.

TLC: R_f = 0.7 (4:1 hexane:acetone, $Ce_2(SO_4)_3$ in phosphomolybdic acid).

[α]$^{20}_{D}$: –92.0 (c = 26.1, $CHCl_3$)

Compound S4

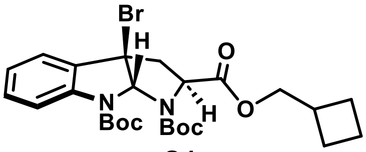

S4

On a 1.25 mmol scale, **General Procedure A** was followed with cyclobutylmethanol. Purification by standard procedure afforded the title compound **S4** (227 mg, 33%).

Physical State: white solid

^{1}H NMR (600 MHz, $CDCl_3$): δ 7.73 – 7.43 (m, 1H), 7.37 (d, *J* = 7.6 Hz, 1H), 7.31 (t, *J* = 7.8 Hz, 1H), 7.12 (t, *J* = 7.5 Hz, 1H), 6.40 (s, 1H), 4.11 (dddd, *J* = 7.0, 7.0, 7.0, 6.9 Hz, 2H), 3.90 (dd, *J* = 10.2, 6.4 Hz, 1H), 3.23 (dd, *J* = 12.7, 6.4 Hz, 1H), 2.81 (dd, *J* = 12.7, 10.1 Hz, 2H), 2.62 (hept, *J* = 7.5 Hz, 1H), 2.06 (dtd, *J* = 12.4, 8.2, 4.0 Hz, 2H), 1.97 – 1.85 (m, 2H), 1.77 (dq, *J* = 11.5, 8.5 Hz, 2H), 1.59 (s, 9H), 1.41 (s, 9H).

^{13}C NMR (151 MHz, $CDCl_3$): δ 171.3, 153.1, 152.3, 141.6, 133.1, 130.7, 124.5, 123.4, 119.2, 84.0, 82.3, 81.5, 69.3, 59.9, 59.7, 42.0, 34.0, 28.3, 24.80, 24.7, 18.5.

HRMS (ESI-TOF): calculated for $C_{26}H_{35}BrN_2NaO_6$ $[M+Na]^+$: 573.1576, found: 573.1578.

TLC: R_f = 0.7 (4:1 hexane:acetone, $Ce_2(SO_4)_3$ in phosphomolybdic acid).

[α]$^{20}_{D}$: –24.5 (c = 1.95, $CHCl_3$)

Compound S5

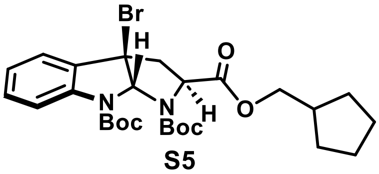

S5

On a 3.75 mmol scale, **General Procedure A** was followed with cyclopentylmethanol. Purification by standard procedure afforded the title compound **S5** (1.67 g, 79%).

Physical State: white solid

^{1}H NMR (600 MHz, $CDCl_3$): δ 7.55 (br, 1H), 7.38 (d, *J* = 7.6 Hz, 1H), 7.32 (td, *J* = 8.0, 1.2 Hz, 1H), 7.13 (t, *J* = 7.5 Hz, 1H), 6.40 (s, 1H), 4.08 – 3.98 (m, 2H), 3.90 (dd, *J* = 10.1, 6.5 Hz, 1H), 3.23 (dd, *J* = 12.7, 6.5 Hz, 1H), 2.81 (dd, *J* = 12.7, 10.1 Hz, 1H), 2.21 (hept, *J* = 7.6 Hz, 1H), 1.80 – 1.71 (m, 2H), 1.59 (s, 9H), 1.63 – 1.51 (m, 4H), 1.41 (s, 9H), 1.28 – 1.21 (m, 2H).

^{13}C NMR (151 MHz, $CDCl_3$): δ 171.2, 152.3, 141.6, 133.1, 130.7, 124.5, 123.4, 119.3, 84.0, 82.3, 81.5, 69.5, 59.9, 59.7, 38.5, 29.4, 29.4, 28.3, 25.3, 25.3.

HRMS (ESI-TOF): calculated for $C_{27}H_{37}BrN_2NaO_6$ $[M+Na]^+$: 587.1733, found: 587.1736.

TLC: R_f = 0.7 (4:1 hexane:acetone, $Ce_2(SO_4)_3$ in phosphomolybdic acid).

[α]^{20}D: –93.0 (c = 14.5, $CHCl_3$)

Compound S6

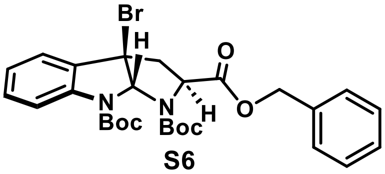

S6

On a 2.50 mmol scale, **General Procedure A** was followed, followed with benzyl alcohol. Purification by standard procedure afforded the title compound **S6** (1.00 g, 70%).

Physical State: white solid

^{1}H NMR (600 MHz, $CDCl_3$): δ 7.47 (s, 1H), 7.38 – 7.27 (m, 7H), 7.10 (t, *J* = 7.6 Hz, 1H), 6.41 (s, 1H), 5.21 (d, *J* = 12.3 Hz, 1H), 5.13 (d, *J* = 12.3 Hz, 1H), 3.94 (dd, *J* = 10.2, 6.4 Hz, 1H), 3.21 (dd, *J* = 12.7, 6.4 Hz, 1H), 2.80 (dd, *J* = 12.7, 10.3 Hz, 1H), 1.59 (s, 9H), 1.39 (s, 9H).

^{13}C NMR (151 MHz, $CDCl_3$): δ 170.9, 152.3, 152.2, 141.5, 135.2, 132.9, 130.6, 128.6, 128.5, 128.4, 124.5, 123.3, 119.2, 83.9, 82.3, 81.5, 67.2, 59.8, 59.6, 41.9, 28.3, 28.2.

HRMS (ESI-TOF): calculated for $C_{28}H_{33}BrN_2NaO_6$ $[M+Na]^+$: 595.1420, found: 595.1422.

TLC: R_f = 0.7 (4:1 hexane:acetone, $Ce_2(SO_4)_3$ in phosphomolybdic acid).

[α]^{20}D: –97.5 (c = 16.7, $CHCl_3$)

Compound S7

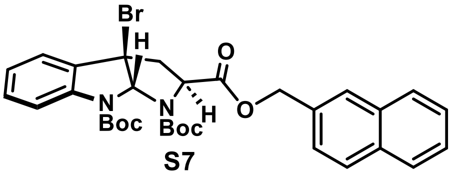

S7

On a 5.2 mmol scale, **General Procedure A** was followed, followed by 2-(naphthalene-2-yl)ethan-1-ol. Purification by standard procedure afforded the title compound **S7** (2.88 g, 87%).

Physical State: white solid

^{1}H NMR (600 MHz, $CDCl_3$): δ 7.81 (dd, *J* = 8.1, 4.5 Hz, 3H), 7.66 (s, 1H), 7.45 (dddd, *J* = 17.8, 8.0, 6.8, 1.4 Hz, 3H), 7.35 (dd, *J* = 8.4, 1.7 Hz, 1H), 7.32 – 7.28 (m, 2H), 7.10 (t, *J* = 7.5 Hz, 1H), 6.39 (s, 1H), 4.50 – 4.39 (m, 2H), 3.86 (dd, *J* = 10.2, 6.4 Hz, 1H), 3.12 (t, *J* = 7.1 Hz, 2H), 3.06 (dd, *J* = 12.7, 6.4 Hz, 1H), 2.65 (dd, *J* = 12.7, 10.3 Hz, 1H), 1.59 (s, 9H), 1.42 (brs, 9H).

^{13}C NMR (151 MHz, $CDCl_3$): δ 170.8, 152.3, 152.3, 141.6, 134.9, 133.6, 133.0, 132.4, 130.7, 128.3, 127.8, 127.6, 127.5, 127.3, 126.2, 125.7, 124.6, 123.3, 118.9, 83.9, 82.4, 81.7, 65.7, 59.8, 59.6, 42.5, 35.2, 28.4, 28.3.

HRMS (ESI-TOF): calculated for $C_{33}H_{37}BrN_2NaO_6$ $[M+Na]^+$: 659.1733, found: 659.1735.

TLC: R_f = 0.7 (4:1 hexane:acetone, $Ce_2(SO_4)_3$ in phosphomolybdic acid).

[α]^{21}D: –47.9 (c = 24.0, $CHCl_3$)

Compound S8

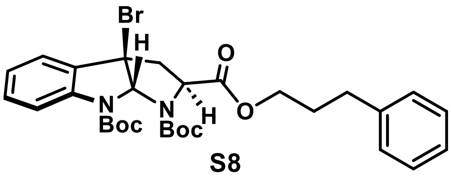

S8

On a 2.50 mmol scale, **General Procedure A** was followed, followed with 3-phenylpropan-1-ol. Purification by standard procedure afforded the title compound **S8** (1.07 g, 71%).

Physical State: white solid
^{1}H NMR (600 MHz, $CDCl_3$): δ 7.48 (s, 1H), 7.37 (d, *J* = 7.7 Hz, 1H), 7.32 (td, *J* = 7.8, 1.3 Hz, 1H), 7.28 (t, *J* = 7.6 Hz, 2H), 7.20 (dd, *J* = 8.4, 1.5 Hz, 1H), 7.18 (d, *J* = 8.0 Hz, 2H), 7.13 (t, *J* = 7.6 Hz, 1H), 6.41 (s, 1H), 4.16 (dd, *J* = 7.5, 5.9 Hz, 2H), 3.90 (dd, *J* = 10.2, 6.4 Hz, 1H), 3.20 (dd, *J* = 12.7, 6.4 Hz, 1H), 2.80 (dd, *J* = 12.7, 10.2 Hz, 1H), 2.69 (dd, *J* = 8.5, 6.8 Hz, 2H), 1.98 (m, 2H), 1.59 (s, 9H), 1.41 (s, 9H).
^{13}C NMR (151 MHz, $CDCl_3$): δ 171.1, 152.3, 152.3, 141.6, 141.0, 133.1, 130.7, 128.5, 128.5, 126.2, 124.6, 123.4, 119.2, 83.9, 82.4, 81.6, 64.9, 59.9, 59.6, 41.9, 32.1, 30.1, 28.4, 28.3.
HRMS (ESI-TOF): calculated for $C_{30}H_{33}BrN_2NaO_6$ $[M+Na]^+$: 623.1733, found: 623.1734.
TLC: R_f = 0.7 (4:1 hexane:acetone, $Ce_2(SO_4)_3$ in phosphomolybdic acid).
$[\alpha]^{21}_D$: –87.7 (c = 12.7, $CHCl_3$)

Compound S9

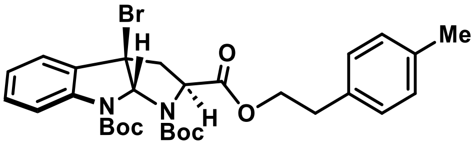

S9

On a 1.25 mmol scale, **General Procedure A** was followed, followed by methyl 2-(4-metylphenyl)ethan-1-ol. Purification by standard procedure afforded the title compound **S9** (1.25 g, 42%).

Physical State: white solid
^{1}H NMR (600 MHz, $CDCl_3$): δ 7.54 (brs, 1H), 7.36 – 7.26 (m, 2H), 7.16 – 7.04 (m, 5H), 6.39 (s, 1H), 4.41 – 4.25 (m, 2H), 3.86 (ddd, *J* = 9.5, 6.4, 2.7 Hz, 1H), 3.12 (dd, *J* = 12.7, 6.4 Hz, 249H), 2.91 (t, *J* = 7.1 Hz, 2H), 2.66 (dd, *J* = 12.7, 10.2 Hz, 1H), 2.31 (s, 9H), 1.59 (s, 9H), 1.39 (brs, 9H).
^{13}C NMR (151 MHz, $CDCl_3$): δ 170.9, 152.2, 141.5, 136.2, 134.2, 132.9, 130.6, 129.3, 128.8, 124.4, 123.3, 118.7, 83.8, 82.2, 81.4, 65.8, 59.8, 59.6, 41.8, 34.5, 28.8, 21.1.
HRMS (ESI-TOF): calculated for $C_{30}H_{37}BrN_2NaO_6$ $[M+Na]^+$: 623,1727, found: 623.1733.
TLC: R_f = 0.7 (4:1 hexane:acetone, $Ce_2(SO_4)_3$ in phosphomolybdic acid).
$[\alpha]^{21}_D$: –32.8 (c = 8.5, $CHCl_3$)

Compound S10

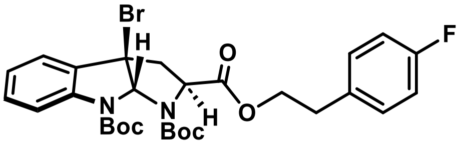

S10

On a 1.25 mmol scale, **General Procedure A** was followed, followed with methyl 2-(4-fluorophenyl)ethan-1-ol. Purification by standard procedure afforded the title compound **S10** (249 mg, 33%).

Physical State: white solid
^{1}H NMR (600 MHz, CDCl$_3$): δ 7.48 (brs, 1H), 7.35 (d, *J* = 7.6 Hz, 1H), 7.32 (t, *J* = 7.8 Hz, 1H), 7.18 (dd, *J* = 8.4, 5.5 Hz, 2H), 7.12 (t, *J* = 7.5 Hz, 1H), 7.00 (t, *J* = 8.6 Hz, 2H), 6.38 (s, 1H), 4.32 (t, *J* = 7.0 Hz, 2H), 3.87 (dd, *J* = 10.2, 6.4 Hz, 1H), 3.14 (dd, *J* = 12.7, 6.4 Hz, 1H), 2.93 (t, *J* = 7.0 Hz, 2H), 2.69 (dd, *J* = 12.7, 10.2 Hz, 1H), 1.59 (s, 9H), 1.41 (brs, 9H).
^{13}C NMR (151 MHz, CDCl$_3$): δ 162.6, 161.0, 152.2, 141.5, 133.1, 130.7, 130.5, 130.4, 124.5, 123.3, 115.5, 115.4, 83.9, 82.4, 65.7, 59.6, 34.2, 28.3, 28.3.
^{19}F NMR (565 MHz, CDCl$_3$): δ −116.3.
HRMS (ESI-TOF): calculated for $C_{29}H_{34}BrFN_2NaO_6$ $[M+Na]^+$: 627.1482, found: 627.1486.
TLC: R_f = 0.7 (4:1 hexane:acetone, $Ce_2(SO_4)_3$ in phosphomolybdic acid).
$[\alpha]^{21}_D$: –59.0 (c = 8.3, $CHCl_3$)

Compound S11

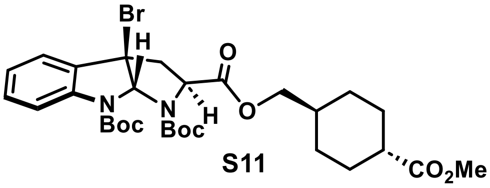

S11

On a 2.50 mmol scale, **General Procedure A** was followed, followed with methyl (1*R*,4*R*)-4-(hydroxymethyl)cyclohexane-1-carboxylate. Purification by standard procedure afforded the title compound **S11** (1.22 g, 77%).

Physical State: white solid
^{1}H NMR (600 MHz, CDCl$_3$): δ 7.50 (brs, 1H), 7.38 (dd, *J* = 7.6, 2.6 Hz, 1H), 7.32 (td, *J* = 8.1, 3.0 Hz, 1H), 7.14 (dt, *J* = 10.7, 5.5 Hz, 1H), 6.39 (s, 1H), 3.96 (ddt, *J* = 17.1, 13.2, 8.9 Hz, 2H), 3.90 (ddd, *J* = 10.0, 6.5, 2.5 Hz, 1H), 3.67 (s, 3H), 3.22 (dd, *J* = 12.7, 6.4 Hz, 1H), 2.80 (t, *J* = 11.4 Hz, 1H), 2.26 (td, *J* = 12.3, 3.4 Hz, 1H), 2.02 (d, *J* = 12.3 Hz, 2H), 1.85 (d, *J* = 9.2 Hz, 2H), 1.59 (s, 9H), 1.52 – 1.33 (m, 2H), 1.43 (s, 9H), 1.02 (q, *J* = 12.8 Hz, 2H).
^{13}C NMR (151 MHz, CDCl$_3$): δ 176.2, 171.1, 152.3, 152.3, 141.6, 133.1, 130.7, 124.6, 123.4, 119.2, 83.9, 82.4, 81.6, 70.1, 59.9, 59.7, 51.6, 43.1, 36.4, 28.7, 28.6, 28.3, 28.3, 28.2.

HRMS (ESI-TOF): calculated for $C_{30}H_{41}BrN_2NaO_8$ $[M+Na]^+$: 659.1944, found: 659.1949.
TLC: R_f = 0.6 (4:1 hexane:acetone, $Ce_2(SO_4)_3$ in phosphomolybdic acid).
$[\alpha]^{20}_D$: −50.8 (c = 12.3, $CHCl_3$)

Compound S12

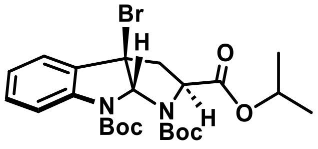

S12

On a 3.75 mmol scale, **General Procedure A** was followed, followed with methyl *i*PrOH. Purification by standard procedure afforded the title compound **S12** (1.23 g, 65%).

Physical State: white solid
^{1}H NMR (600 MHz, $CDCl_3$): δ 7.42 (brs, 1H), 7.33 (d, *J* = 7.6 Hz, 1H), 7.27 (t, *J* = 7.8 Hz, 1H), 7.08 (t, *J* = 7.6 Hz, 1H), 6.36 (s, 1H), 5.01 (p, *J* = 6.4 Hz, 1H), 3.82 (dd, *J* = 10.1, 6.4 Hz, 1H), 3.18 (dd, *J* = 12.7, 6.5 Hz, 1H), 2.74 (dd, *J* = 12.7, 10.1 Hz, 1H), 1.55 (s, 9H), 1.38 (s, 9H), 1.23 (d, *J* = 6.5 Hz, 3H), 1.21 (d, *J* = 6.3 Hz, 3H).
^{13}C NMR (151 MHz, $CDCl_3$): δ 170.3, 152.3, 152.3, 141.5, 133.2, 130.6, 124.6, 123.4, 119.2, 83.9, 82.3, 81.5, 69.1, 59.9, 59.8, 41.8, 28.3, 28.3, 21.7.
HRMS (ESI-TOF): calculated for $C_{24}H_{33}BrN_2NaO_6$ $[M+Na]^+$: 5547.1420, found: 5547.1423.
TLC: R_f = 0.7 (4:1 hexane:acetone, $Ce_2(SO_4)_3$ in phosphomolybdic acid).
$[\alpha]^{21}_D$: −109.6 (c = 5.6, $CHCl_3$)

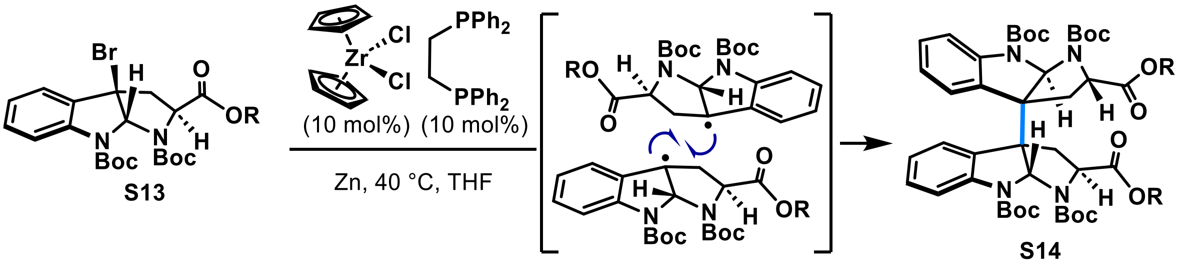

[General Procedure B]

A 25 mL round-bottom flask was added with bromo cyclized product **S13** (1.0 eq.), Cp_2ZrCl_2 (0.1 eq.), 1,2-Bis(diphenylphosphino)ethane (0.1 eq.), Zinc dust (2.0 eq.), purged with N_2 for three times. Then, THF (0.1 M) was added slowly via the syringe. The reaction mixture was stirred at 40℃ for 12 h, then the reaction mixture turned green and was filtered and concentrated under reduced pressure. The crude residue was purified by preparative thin-layer chromatography (silica gel, 10:1 hexane:Acetone) to afford the dimeric compound **S14**.

Compound 12

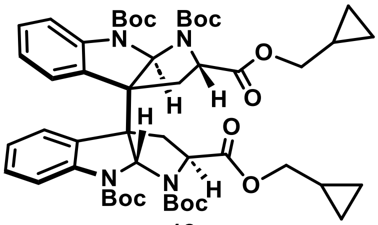

12

On a 1.25 mmol scale, **General Procedure B** was followed with bromo-cyclized compound **S3** (107 mg, 0.2 mmol, 1.0 eq.). Purification by standard procedure afforded the title compound **12** (40.4 mg, 44%).

Physical State: white solid

^{1}H NMR (400 MHz, CD_3OD, 60 °C): δ 8.75 (d, *J* = 7.8 Hz, 2H), 8.59 (d, *J* = 7.5 Hz, 2H), 8.54 (t, *J* = 7.8 Hz, 2H), 8.32 (t, *J* = 7.4 Hz, 2H), 7.74 (s, 2H), 5.45 – 5.31 (m, 4H), 5.21 (t, *J* = 8.5 Hz, 2H), 4.10 (s, 2H), 3.83 (dd, *J* = 12.9, 9.4 Hz, 2H), 3.03 (s, 18H), 2.79 (s, 18H), 2.33 – 2.28 (m, 2H), 2.00 (d, *J* = 7.9 Hz, 4H), 1.72 (d, *J* = 4.8 Hz, 4H).

^{13}C NMR (101 MHz, CD_3OD, 60 °C): δ 174.0, 153.4, 153.4, 143.2, 132.5, 130.3, 125.1, 124.3, 118.2, 83.4, 82.5, 80.8, 71.4, 60.3, 59.8, 56.1, 36.9, 28.8, 28.6, 10.5, 3.7.

HRMS (ESI-TOF): calculated for $C_{50}H_{66}N_4NaO_{12}$ $[M+Na]^+$: 937.4569, found: 937.4573.

TLC: R_f = 0.4 (4:1 hexane:acetone, $Ce_2(SO_4)_3$ in phosphomolybdic acid).

$[\alpha]^{21}_D$: –25.8 (c = 1.67, $CHCl_3$)

Note: Due to rotamerism at room temperature, NMR spectra used for assignment were taken at 60 ˚C in CD_3OD.

Compound 13

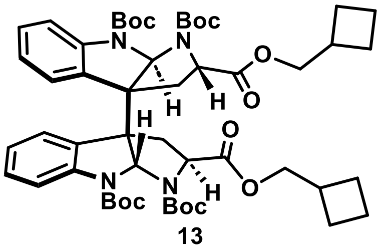

13

On a 0.2 mmol scale, **General Procedure B** was followed with bromo-cyclized compound **S4** (110 mg, 0.2 mmol, 1.0 eq.) and Bis(butylcyclopentadienyl)zirconium(IV) dichloride (8.0 mg, 20 µmol, 0.1 eq.) instead of Cp_2ZrCl_2. Purification by standard procedure afforded the title compound **13** (49.0 mg, 52%).

Physical State: white solid

^{1}H NMR (400 MHz, CD_3OD, 60 °C): The mixture of multiple rotamers. (See the spectrum below.)

^{13}C NMR (101 MHz, CD_3OD, 60 °C): The mixture of multiple rotamers. (See the spectrum below.)

HRMS (ESI-TOF): calculated for $C_{52}H_{70}N_4NaO_{12}$ $[M+Na]^+$: 965.4882, found: 965.4882.

TLC: R_f = 0.4 (4:1 hexane:acetone, $Ce_2(SO_4)_3$ in phosphomolybdic acid).

[α]^{21}$_{D}$: −12.9 (c = 3.2, $CHCl_3$)

Note: Due to rotamerism at room temperature, NMR spectrums were taken at 60 ˚C in CD_3OD. However, the presence of rotamers was not resolved.

Compound 14

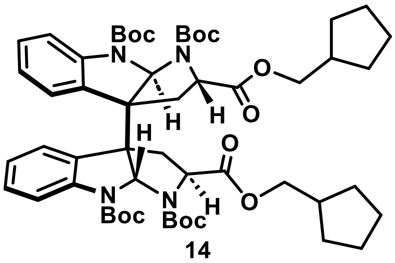

14

On a 0.2 mmol scale, **General Procedure B** was followed with bromo-cyclized compound **S5** (113 mg, 0.2 mmol, 1.0 eq.) and Bis(butylcyclopentadienyl)zirconium(IV) dichloride (8.0 mg, 20 µmol, 0.1 eq.) instead of Cp_2ZrCl_2. Purification by standard procedure afforded the title compound **14** (34.0 mg, 35%).

Physical State: white solid

^{1}H NMR (400 MHz, CD_3OD, 60 °C): The mixture of multiple rotamers. (See the spectrum below.)

^{13}C NMR (101 MHz, CD_3OD, 60 °C): δ The mixture of multiple rotamers. (See the spectrum below.)

HRMS (ESI-TOF): calculated for $C_{54}H_{74}N_4NaO_{12}$ $[M+Na]^+$: 993.5195, found: 993.5209.

TLC: R_f = 0.4 (4:1 hexane:acetone, $Ce_2(SO_4)_3$ in phosphomolybdic acid).

[α]^{21}$_{D}$: −21.5 (c = 2.2, $CHCl_3$)

Note: Due to rotamerism at room temperature, NMR spectra used for assignment were taken at 60 ˚C in CD_3OD. However, the presence of rotamers was not resolved.

Compound 15

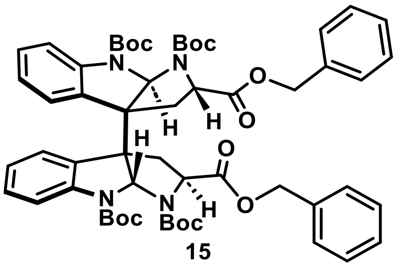

15

On a 0.2 mmol scale, **General Procedure B** was followed with bromo-cyclized compound **S6** (114 mg, 0.2 mmol, 1.0 eq.) and Bis(butylcyclopentadienyl)zirconium(IV) dichloride (8.0 mg, 20 µmol, 0.1 eq.) instead of Cp_2ZrCl_2. Purification by standard procedure afforded the title compound **15** (57.2 mg, 58%).

Physical State: white solid

^{1}H NMR (400 MHz, DMSO-*d6*, 70 °C): δ 7.37 (s, 14H), 7.24 – 7.12 (m, 2H), 6.89 (t, *J* = 7.5 Hz, 2H), 6.02 (s, 2H), 5.29 – 5.06 (m, 4H), 3.76 (t, *J* = 8.3 Hz, 2H), 2.32 (dd, *J* = 12.7, 9.2 Hz, 2H), 1.54 (s, 18H), 1.26 (s, 18H).

^{13}C NMR (101 MHz, DMSO-*d6*, 70 °C): δ 171.9, 151.4, 142.0, 135.9, 131.3, 129.7, 128.9, 128.6, 128.5, 128.5, 124.4, 123.3, 81.7, 81.0, 79.5, 67.0, 59.0, 58.8, 35.4, 28.4, 28.2.

HRMS (ESI-TOF): calculated for $C_{56}H_{66}N_4NaO_{12}$ $[M+Na]^+$: 1009.4569, found: 1009.4569.

TLC: R_f = 0.4 (4:1 hexane:acetone, $Ce_2(SO_4)_3$ in phosphomolybdic acid).

$[\alpha]^{20}_D$: –13.6 (c = 1.3, $CHCl_3$)

Note: Due to rotamerism at room temperature, NMR spectra used for assignment were taken at 70 ˚C in DMSO-*d6*.

Compound 16

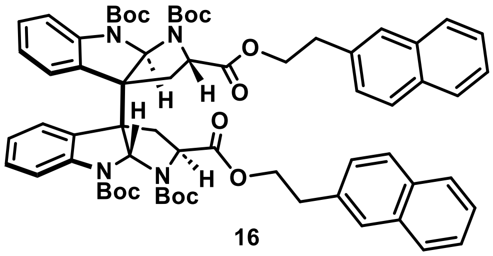

16

On a 0.2 mmol scale, **General Procedure B** was followed with bromo-cyclized compound **S7** (250 mg, 0.393 mmol, 1.0 eq.). Purification by standard procedure afforded the title compound **16** (84.0 mg, 38%).

Physical State: white solid

^{1}H NMR (400 MHz, CD_3OD, 60 °C): δ 7.80 – 7.72 (m, 6H), 7.65 (s, 2H), 7.47 – 7.30 (m, 8H), 7.13 (t, *J* = 7.8 Hz, 0H), 6.99 (d, *J* = 7.6 Hz, 2H), 6.88 (t, *J* = 7.5 Hz, 2H), 6.21 (brs, 2H), 4.38 (tq, *J* = 10.8, 6.7, 5.5 Hz, 4H), 3.70 (dd, *J* = 9.4, 7.0 Hz, 2H), 3.09 (t, *J* = 6.8 Hz, 4H), 2.41 (dd, *J* = 12.8, 7.0 Hz, 2H), 2.28 (dd, *J* = 12.8, 9.6 Hz, 2H), 1.58 (s, 18H), 1.29 (s, 18H).

^{13}C NMR (101 MHz, CD_3OD, 60 °C): δ 172.2, 151.9, 141.8, 135.2, 133.7, 132.5, 131.0, 129.0, 127.9, 127.3, 127.3, 127.1, 127.0, 125.8, 125.2, 123.7, 123.0, 82.0, 81.1, 79.3, 65.6, 59.0, 58.5, 35.4, 34.7, 27.4, 27.2.

HRMS (ESI-TOF): calculated for $C_{66}H_{74}N_4NaO_{12}$ $[M+Na]^+$: 1137.5195, found: 1137.5193.

TLC: R_f = 0.4 (4:1 hexane:acetone, $Ce_2(SO_4)_3$ in phosphomolybdic acid).

$[\alpha]^{25}_D$: –74.3 (c = 4.7, $CHCl_3$)

Note: Due to rotamerism at room temperature, NMR spectra used for assignment were taken at 60 ˚C in CD_3OD.

Compound 17

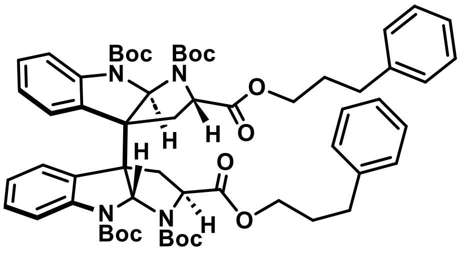

17

On a 0.2 mmol scale, **General Procedure B** was followed with bromo-cyclized compound **S8** (120 mg, 0.2 mmol, 1.0 eq.) and Bis(butylcyclopentadienyl)zirconium(IV) dichloride (8.0 mg, 20 µmol, 0.1 eq.) instead of Cp_2ZrCl_2. Purification by standard procedure afforded the title compound **17** (50.0 mg, 48%).

Physical State: white solid

^{1}H NMR (400 MHz, CD_3OD, 60 °C): δ 7.36 (d, *J* = 4.4 Hz, 2H), 7.25 (dd, *J* = 8.7, 3.6 Hz, 4H), 7.17 (m, 10H), 6.94 (t, *J* = 8.2, 4.2 Hz, 2H), 6.34 (s, 2H), 4.15 – 4.06 (m, 4H), 3.71 (d, *J* = 9.3 Hz, 2H), 2.68 (td, *J* = 7.6, 4.5 Hz, 4H), 2.55 (dd, *J* = 20.7, 7.7 Hz, 2H), 2.41 – 2.30 (m, 2H), 2.00 – 1.90 (m, 4H), 1.61 (s, 18H), 1.37 (s, 18H).

^{13}C NMR (101 MHz, CD_3OD, 60 °C): δ 161.9, 153.4, 143.1, 142.6, 130.5, 129.6, 129.5, 127.1, 125.0, 124.4, 83.4, 80.6, 66.2, 60.2, 36.7, 33.3, 31.2, 28.7, 28.4.

HRMS (ESI-TOF): calculated for $C_{60}H_{74}N_4NaO_{12}$ $[M+Na]^+$: 1065.5195, found 1065.5195.

TLC: R_f = 0.4 (4:1 hexane:acetone, $Ce_2(SO_4)_3$ in phosphomolybdic acid).

$[\alpha]^{21}_D$: −29.0 (c = 1.1, $CHCl_3$)

Note: Due to rotamerism at room temperature, NMR spectra used for assignment were taken at 60 ˚C in CD_3OD.

Compound 18

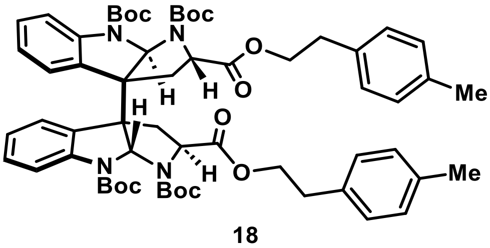

18

On a 0.2 mmol scale, **General Procedure B** was followed with bromo-cyclized compound **S9** (250 mg, 0.417 mmol, 1.0 eq.). Purification by standard procedure afforded the title compound **18** (92.0 mg, 42%).

Physical State: white solid

^{1}H NMR (400 MHz, CD_3OD, 60 °C): δ 7.36 (d, *J* = 8.0 Hz, 2H), 7.16 – 7.05 (m, 12H), 6.90 (t, *J* = 7.5 Hz, 2H), 6.29 – 6.23 (m, 2H), 4.38 – 4.15 (m, 4H), 3.73 (ddd, *J* = 9.3, 7.0, 1.7 Hz, 2H), 2.89 (td,

J = 7.0, 1.8 Hz, 4H), 2.50 (dd, *J* = 11.9, 7.0 Hz, 2H), 2.35 – 2.23 (m, 2H), 2.28 (s, 6H), 1.61 (s, 18H), 1.33 (s, 18H).

^{13}C NMR (101 MHz, CD_3OD, 60 °C): δ 173.5, 153.2, 143.1, 137.3, 135.8, 132.4, 130.4, 130.2, 130.2, 130.1, 129.9, 129.8, 124.9, 124.3, 118.2, 83.3, 82.5, 80.7, 67.1, 60.3, 36.9, 35.5, 30.6, 28.8, 28.7, 28.6, 28.5, 21.2, 21.1.

HRMS (ESI-TOF): calculated for $C_{60}H_{74}N_4NaO_{12}$ $[M+Na]^+$: 1065.5195, found: 11065.5197.

TLC: R_f = 0.4 (4:1 hexane:acetone, $Ce_2(SO_4)_3$ in phosphomolybdic acid).

$[\alpha]^{21}_D$: –74.1 (c = 5.6, $CHCl_3$)

Note: Due to rotamerism at room temperature, NMR spectra used for assignment were taken at 60 ˚C in CD_3OD.

Compound 19

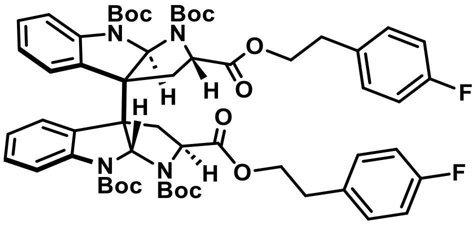

19

On a 0.2 mmol scale, **General Procedure B** was followed with bromo-cyclized compound **S10** (390 mg, 0.644 mmol, 1.0 eq.). Purification by standard procedure afforded the title compound **19** (223 mg, 33%).

Physical State: white solid

^{1}H NMR (400 MHz, CD_3OD, 60 °C, mixture of two rotamers, A:B = 1:1): δ 8.10 (d, *J* = 8.2 Hz, 1H), 7.49 (d, *J* = 7.8 Hz, 1H), 7.43 (s, 1H), 7.26 (m, 6H), 7.14 (dd, *J* = 8.3, 5.6 Hz, 2H), 7.08 (t, *J* = 7.5 Hz, 1H), 7.02 (t, *J* = 8.8 Hz, 2H), 6.95 (t, *J* = 8.8 Hz, 2H), 6.26 (d, *J* = 5.8 Hz, 1H), 4.36 – 4.18 (m, 4H), 3.96 (s, 1H), 3.81 (dd, *J* = 9.8, 6.9 Hz, 1H), 3.13 (dd, *J* = 14.6, 5.8 Hz, 1H), 2.93 (t, *J* = 6.7 Hz, 3H), 2.80 (dt, *J* = 9.9, 4.9 Hz, 2H), 2.55 (dd, *J* = 12.9, 7.0 Hz, 1H), 2.17 – 2.08 (m, 2H), 1.65 (s, 9H), 1.57 (s, 8H), 1.34 (s, 18H).

^{13}C NMR (101 MHz, CD_3OD, 60 °C, mixture of two rotamers, A:B = 1:1): δ 174.3, 173.6, 164.5, 162.1, 160.0, 154.1, 151.1, 143.2, 136.9, 135.2, 133.8, 131.7, 131.6, 131.6, 131.5, 129.3, 125.5, 125.4, 125.2, 125.0, 124.8, 123.7, 119.9, 118.6, 117.4, 116.3, 116.2, 116.2, 116.0, 115.9, 85.0, 83.5, 83.0, 82.2, 78.6, 66.8, 60.6, 46.1, 35.1, 35.0, 33.5, 28.7, 28.6, 28.5, 28.5.

^{19}F NMR (377 MHz, CD_3OD, 60 °C, mixture of two rotamers, A:B = 1:1): δ -118.9.

HRMS (ESI-TOF): calculated for $C_{58}H_{68}F_2N_4NaO_{12}$ $[M+Na]^+$: 1073.4694, found: 1073.4690.

TLC: R_f = 0.4 (4:1 hexane:acetone, $Ce_2(SO_4)_3$ in phosphomolybdic acid).

$[\alpha]^{20}_D$: –10.1 (c = 2.3, $CHCl_3$)

Note: Due to rotamerism at room temperature, NMR spectra used for assignment were taken at 60 ˚C in CD_3OD.

Compound 20

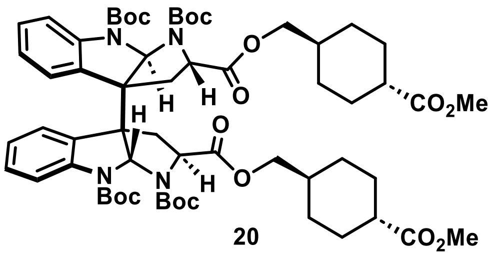

20

On a 0.2 mmol scale, **General Procedure B** was followed with bromo-cyclized compound **S11** (127 mg, 0.2 mmol, 1.0 eq.). Purification by standard procedure afforded the title compound **20** (37.8 mg, 34%).

Physical State: white solid
^{1}H NMR (400 MHz, CD_3OD, 60 °C): δ ^{1}H NMR (400 MHz, Chloroform-*d*) δ 7.35 (d, *J* = 8.2 Hz, 2H), 7.19 – 7.09 (m, 4H), 6.92 (t, *J* = 7.5 Hz, 2H), 6.32 (s, 2H), 4.03 – 3.87 (m, 4H), 3.79 (t, *J* = 8.3 Hz, 2H), 3.66 (s, 6H), 2.73 – 2.59 (m, 2H), 2.40 (dd, *J* = 12.8, 9.5 Hz, 2H), 2.30 (tt, *J* = 12.3, 3.7 Hz, 2H), 2.06 – 1.97 (m, 4H), 1.91 – 1.83 (m, 4H), 1.72 – 1.63 (m, 2H), 1.62 (s, 18H), 1.38 (s, 18H), 1.48 – 1.20 (m, 4H), 1.09 (qd, *J* = 13.0, 3.1 Hz, 4H).
^{13}C NMR (101 MHz, CD_3OD, 60 °C): δ 180.4, 176.3, 156.0, 145.7, 135.0, 132.9, 127.7, 126.9, 120.8, 86.0, 85.2, 83.3, 73.9, 62.9, 62.5, 54.5, 46.9, 40.3, 39.4, 32.4, 32.3, 32.0, 31.4, 31.2.
HRMS (ESI-TOF): calculated for $C_{60}H_{82}N_4NaO_{16}$ $[M+Na]^+$: 1137.5618, found: 1137.5620.
TLC: R_f = 0.3 (4:1 hexane:acetone, $Ce_2(SO_4)_3$ in phosphomolybdic acid).
$[\alpha]^{21}_D$: −7.8 (c = 8.4, $CHCl_3$)
Note: Due to rotamerism at room temperature, NMR spectra used for assignment were taken at 60 ˚C in CD_3OD.

Compound 21

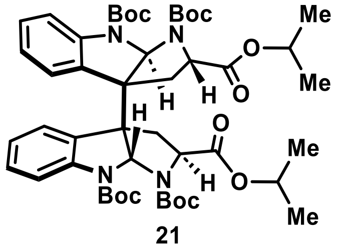

21

On a 0.2 mmol scale, **General Procedure B** was followed with bromo-cyclized compound **S12** (105 mg, 0.2 mmol, 1.0 eq.). Purification by standard procedure afforded the title compound **21** (32.1 mg, 36%).

Physical State: white solid
^{1}H NMR (400 MHz, CD_3OD, 60 °C):δ 7.32 (d, *J* = 8.1 Hz, 2H), 7.18 (d, *J* = 7.6 Hz, 2H), 7.10 (t, *J* = 7.8 Hz, 2H), 6.89 (t, *J* = 7.5 Hz, 2H), 6.33 (s, 2H), 5.00 (p, *J* = 6.2 Hz, 2H), 3.76 (dd, *J* = 9.3, 7.2

Hz, 2H), 2.71 (dd, J = 12.6, 6.4 Hz, 2H), 2.40 (dd, J = 12.8, 9.4 Hz, 2H), 1.63 (s, 18H), 1.39 (s, 18H), 1.29 (d, J = 6.1 Hz, 6H), 1.26 (d, J = 6.0 Hz, 6H).
^{13}C NMR (101 MHz, CD_3OD, 60 °C): δ 173.4, 153.3, 143.1, 132.6, 130.3, 124.9, 124.3, 118.1, 83.4, 82.5, 80.7, 70.6, 60.3, 59.8, 36.9, 28.8, 28.7, 28.6, 22.1, 22.0.
HRMS (ESI-TOF): calculated for $C_{48}H_{66}N_4NaO_{12}$ $[M+Na]^+$: 913.4569, found: 913.4578.
TLC: R_f = 0.4 (4:1 hexane:acetone, $Ce_2(SO_4)_3$ in phosphomolybdic acid).
$[\alpha]^{22}_D$: −164.1 (c = 3.1, $CHCl_3$)
Note: Due to rotamerism at room temperature, NMR spectra used for assignment were taken at 60 ˚C in CD_3OD.

Compound 22

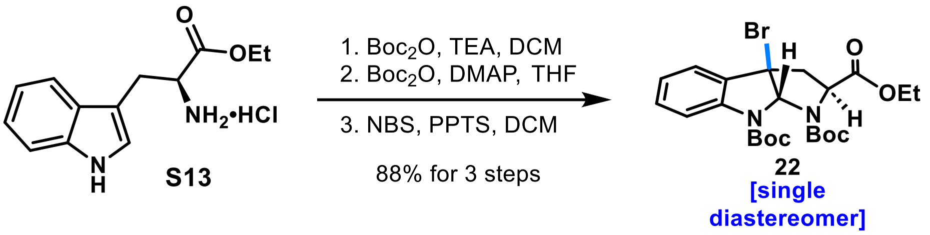

Step 1: L-Tryptophan ethyl ester hydrochloride **S13** (50 g, 186 mmol, 1.0 eq.), Boc_2O (42.7 mL, 186 mmol, 1.0 eq.), and triethylamine (38.8 mL, 279 mmol, 1.5 eq.) were dissolved in 500 mL DCM. The reaction mixture was stirred at room temperature for 2 h, then water was added into the reaction mixture, and extracted with DCM (300 mL x3). The combined organic layer was dried over Na_2SO_4 and concentrated under reduced pressure. The crude was used for the next step without purification.
Step 2: The crude, Boc_2O (42.7 mL, 186 mmol, 1.0 eq.), and DMAP (2.27 g, 18.6 mmol, 0.1 eq.) were dissolved in 625 mL THF. The reaction mixture was stirred at room temperature for 30 min. Then the water was added into the reaction mixture, and extracted with EtOAc (500 mL x3). The combined organic layer was dried over Na_2SO_4 and concentrated under reduced pressure. The crude was used for the next step without further purification.
Step 3[1]: The crude and pyridinium *p*-toluenesulfonate (46.7 g, 186 mmol, 1.0 eq.) were dissolved in 700 mL DCM in dark. *N*-Bromosuccinimide (33.1 g, 186 mmol, 1.0 eq.) was added into the reaction mixture portionwise (20 portions, 10 min/portion) and stirred at room temperature. After the addition of all portions, and all starting material consumed according to the TLC, saturated $Na_2S_2O_3$ solution was added into the reaction mixture, and extracted with DCM (500 mL x3). The combined organic layer was dried over Na_2SO_4 and concentrated under reduced pressure. The crude residue was purified by column chromatography (silica gel, 4:1 hexane:EtOAc) to afford 83.7 g (88% for 3 steps) of the title compound **23**.
Note: To obtain a single diastereomer **22**, the portionwise of NBS is essential.

Physical State: white solid
^{1}H NMR (600 MHz, $CDCl_3$): δ 7.77 – 7.39 (brs, 1H) 7.36 (d, J = 7.6 Hz, 1H), 7.31 (t, J = 7.9 Hz, 1H), 7.12 (t, J = 15.2 Hz, 1H), 6.39 (s, 1H), 4.21 – 4.16 (m, 2H), 3.90 – 3.84 (m, 1H), 3.21 (dd, J = 12.7, 6.4 Hz, 1H), 2.80 (t, J = 11.5 Hz, 1H), 1.58 (s, 9H) 1.40 (s, 9H), 1.27 (t, J = 7.2 Hz, 3H).

^{13}C NMR (151 MHz, CDCl$_3$): δ 171.0, 152.3, 141.6, 133.0, 130.7, 124.5, 123.4, 119.0, 118.4, 83.9, 82.3, 81.5, 61.5, 59.9, 59.7, 42.1, 28.4, 14.2.
HRMS (ESI-TOF): calculated for $C_{23}H_{31}BrN_2NaO_6$ $[M+Na]^+$: 533.1263, found: 533.1266.
TLC: R_f = 0.55 (4:1 hexane:acetone, $Ce_2(SO_4)_3$ in phosphomolybdic acid).
$[\alpha]^{25}_D$: −128.3 (c = 1.0, CHCl$_3$).

Compound 23

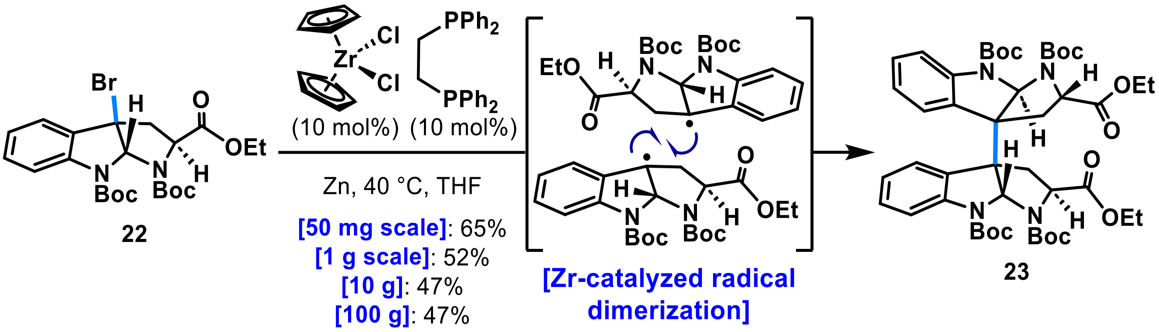

22 23

[100 g scale] A 2.0 L round-bottom flask was added with compound **22** (100 g, 196 mmol, 1.0 eq.), Cp_2ZrCl_2 (5.73 g, 19.6 mmol, 0.1 eq.), 1,2-Bis(diphenylphosphino)ethane (7.81 g, 19.6 mmol, 0.1 eq.), Zinc dust (25.6 g, 392 mmol, 2.0 eq.), purged with N_2 for three times. Then, 1.0 L THF was added slowly via the syringe. The reaction mixture was stirred at 40°C for 8 h, then the reaction mixture turned green and was filtered, concentrated under reduced pressure. The crude residue was purified by column chromatography (silica gel, 10:1 hexane:Acetone) to afford 40.0 g (47%) of the dimeric compound **23**.
[10 g scale] A 300 mL round-bottom flask was added with compound **22** (10 g, 19.6 mmol, 1.0 eq.), Cp_2ZrCl_2 (573 mg, 1.96 mmol, 0.1 eq.), 1,2-Bis(diphenylphosphino)ethane (781 mg, 1.96 mmol, 0.1 eq.), Zinc dust (2.56 g, 39.2 mmol, 2.0 eq.), purged with N_2 for three times. Then, 100 mL THF was added slowly via the syringe. The reaction mixture was stirred at 40°C for 12 h, then the reaction mixture turned green and was filtered and concentrated under reduced pressure. The crude residue was purified by column chromatography (silica gel, 10:1 hexane:Acetone) to afford 3.98 g (47%) of the dimeric compound **23**.
[1 g scale] Same procedures as above. 100 ml round-bottom flask, compound **22** (1.0 g, 1.96 mmol, 1.0 eq.), Cp_2ZrCl_2 (57.3 mg, 0.196 mmol, 0.1 eq.), 1,2-Bis(diphenylphosphino)ethane (78.1 mg, 0.196 mmol, 0.1 eq.), Zinc dust (256 mg, 3.92 mmol, 2.0 eq.) and 10 mL THF.
439 mg (52%) of the dimeric compound **23** was obtained.
[50 mg scale] Same procedures as above. 10 ml vial, compound **22** (50 mg, 0.1 mmol), Cp_2ZrCl_2 (2.9 mg, 0.01 mmol, 0.1 eq.), 1,2-Bis(diphenylphosphino)ethane (3.9 mg, 0.01 mmol, 0.1 eq.), Zinc dust (0.0256g, 0.2 mmol, 2.0 eq.) and 1 mL THF.
27.5 mg (65%) of the dimeric compound **23** was obtained.

Physical State: white amorphous
^{1}H NMR (600 MHz, CDCl$_3$): The mixture of rotamers was observed. (See spectrum below)

^{13}C NMR (151 MHz, CDCl$_3$): The mixture of rotamers was observed. (See spectrum below)
HRMS (ESI-TOF): calculated for $C_{46}H_{62}N_4NaO_{12}$ [M+Na]$^+$: 885.4262, found: 885.4271.
TLC: R$_f$ = 0.4 (3:1 hexane:Acetone, $Ce_2(SO_4)_3$ in phosphomolybdic acid).
[α]$^{25}_{D}$: −110.9 (c = 1.0, CHCl$_3$)
Note: 1H NMR and 13C NMR were obtained at room temperature because the product is unstable at high temperatures.

(–)-ditryptophenaline (38)

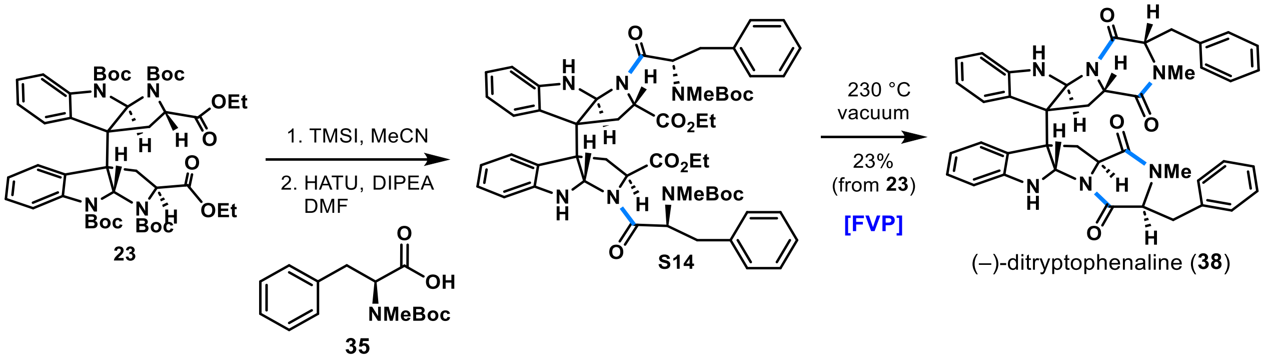

Step 1: To a stirred solution of the compound **23** (5.97 g, 6.91 mmol, 1.0 eq.) in MeCN (35 mL, 0.2 M) was added TMSI (7.9 mL, 55.4 mol, 8.0 eq.) dropwise at 0 °C for 15 min. The reaction mixture was warmed to room temperature and stirred for 30 min. The reaction was quenched by $NaHCO_3$ solution and extracted with DCM/MeOH = 10:1 (50 mL x5). The combined organic layer was dried over Na_2SO_4 and concentrated under reduced pressure to give the crude indoline. The crude mixture was used for the next step without further purification.
Step 2: To a solution of the crude indoline, DIPEA (12 mL, 69 mmol. 10 eq.) and *N*-(*tert*-butoxycarbonyl)-*N*-methyl-*L*-phenylalanine (10.5 g, 37.2 mmol, 5.4 eq.) in DMF (30 mL, 0.2 M), HATU (13.2 g, 34.6 mmol, 5.0 eq.) was added at room temperature. The resulting mixture was stirred for 16 h. Then, the reaction was slowly quenched by aqueous 0.5 N HCl. The mixture was extracted with EtOAc (50 mL x3). The combined organic layer was washed with brine and saturated aqueous $NaHCO_3$. Combined organic layers were dried over Na_2SO_4, and concentrated under reduced pressure. The crude mixture was used for the next step without further purification.
Step 3: The crude mixture in round bottom flask was heated at 230 °C under vacuum condition for 15 min. The crude residue was purified by column chromatography (silica gel, MeOH:EtOAc = 3:97) to afford 1.11 g (23% from compound **S3**) of the (–)-ditryptophenaline (**38**).

Physical State: brown solid
^{1}H NMR (400 MHz, CDCl$_3$): δ 7.54 (t, *J* = 7.0 Hz, 4H), 7.49 (t, *J* = 7.1 Hz, 2H), 7.13 (d, *J* = 6.7 Hz, 4H), 7.06 (t, *J* = 7.6 Hz, 2H), 6.96 (d, *J* = 7.6 Hz, 2H), 6.69 (t, *J* = 7.5 Hz, 2H), 6.54 (d, *J* = 7.8 Hz, 2H), 4.81 (s, 2H), 4.24 (s, 2H), 3.65 (dd, *J* = 11.4, 4.4 Hz, 2H), 3.51 (dd, *J* = 14.4, 3.2 Hz, 2H), 3.24 (dd, *J* = 14.3, 4.4 Hz, 2H), 3.01 (s, 6H), 2.01 (dd, *J* = 12.4, 4.9 Hz, 2H), 1.56 (t, *J* = 12.2 Hz, 2H).
^{13}C NMR (101 MHz, CDCl$_3$): δ 165.5, 164.1, 150.3, 134.6, 129.7, 129.5, 129.4, 128.0, 126.5, 125.8, 119.0, 109.7, 78.7, 63.2, 59.0, 58.6, 36.3, 36.0, 32.7.

HRMS (ESI-TOF): calculated for $C_{42}H_{41}N_6O_4$ $[M+H]^+$: 693.3189, found: 693.3187.
TLC: R_f = 0.3 (EtOAc only, $Ce_2(SO_4)_3$ in phosphomolybdic acid).
$[\alpha]^{25}_D$: –286 (c = 0.6, CH_2Cl_2).

(–)-dibrevianamide F (39)

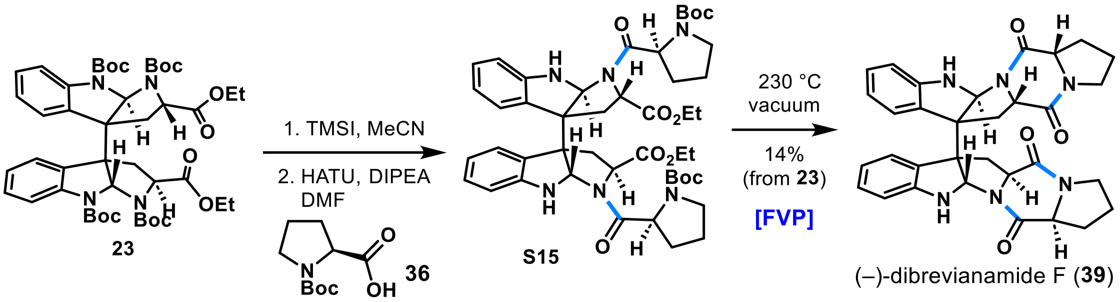

Step 1: To a stirred solution of the compound 23 (6.77 g, 7.85 mmol, 1.0 eq.) in MeCN (40 mL, 0.2 M) was added TMSI (8.9 mL, 63 mmol, 8.0 eq.) dropwise at 0 °C for 15 min. The reaction mixture was warmed to room temperature and stirred for 30 min. The reaction was quenched by $NaHCO_3$ solution and extracted with DCM/MeOH = 10:1 (50 mL x5). The combined organic layer was dried over Na_2SO_4 and concentrated under reduced pressure to give the crude indoline. The crude mixture was used for the next step without further purification.
Step 2: To a solution of the crude indoline, DIPEA (14 mL, 79 mmol. 10 eq.) and *N*-(*tert*-butoxycarbonyl)-*L*-proline (10.1 g, 47.1 mmol, 6.0 eq.) in DMF (40 mL, 0.2 M), HATU (14.9 g, 39.2 mmol, 5.0 eq.) was added at room temperature. The resulting mixture was stirred for 16 h. Then, the reaction was slowly quenched by aqueous 0.5 N HCl. The mixture was extracted with EtOAc (50 mL x3). The combined organic layer was washed with brine and saturated aqueous $NaHCO_3$. Combined organic layers were dried over Na_2SO_4 and concentrated under reduced pressure. The crude mixture was used for the next step without further purification.
Step 3: The crude mixture in round bottom flask was heated at 230 °C under vacuum condition for 10 min. The crude residue was purified by column chromatography (silica gel, MeOH:EtOAc = 3:97) to afford 615 mg (14% from compound **S3**) of the (–)-dibrevianamide F (**39**).

Physical State: brown solid
^{1}H NMR (400 MHz, $CDCl_3$): δ 7.25 (d, *J* = 7.6 Hz, 2H), 7.14 (td, *J* = 7.7, 1.2 Hz, 2H), 5.20 (s, 4H), 4.00 (ddd, *J* = 9.2, 7.0, 1.9 Hz, 2H), 3.93 (dd, *J* = 10.9, 6.3 Hz, 2H), 2.74 (dd, *J* = 13.0, 10.9 Hz, 2H), 2.61 (dd, *J* = 13.0, 6.3 Hz, 2H), 2.31 – 2.15 (m, 2H), 2.15 – 1.96 (m, 4H), 1.94 – 1.77 (m, 2H).
^{13}C NMR (101 MHz, $CDCl_3$): δ 166.9, 165.5, 150.0, 129.8, 127.0, 125.5, 119.4, 110.3, 78.3, 60.5, 60.2, 59.5, 45.2, 34.7, 27.7, 23.1.
HRMS (ESI-TOF): calculated for $C_{32}H_{33}N_6O_4$ $[M+H]^+$: 565.2563, found: 565.2560.
TLC: R_f = 0.5 (MeOH:EtOAc 1:19, $Ce_2(SO_4)_3$ in phosphomolybdic acid).
$[\alpha]^{25}_D$: –449 (c = 0.4, $CHCl_3$).

Compound S1[2]

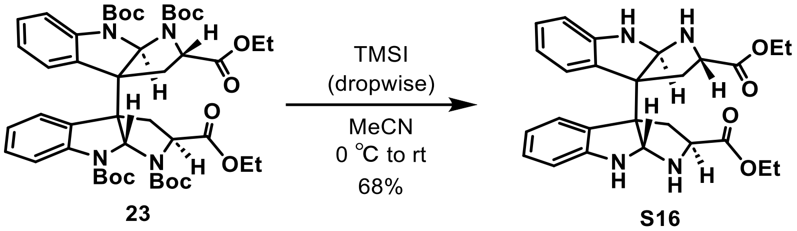

To a stirred solution of the compound **23** (150 g, 170 mmol, 1.0 eq.) in MeCN (1 L) was added TMSI (189 mL, 1.36 mol, 8.0 eq.) dropwise at 0 °C for 15 min. The reaction mixture was warmed to room temperature, stirred for 30 min. The reaction was quenched by $NaHCO_3$ solution and extracted with DCM/MeOH = 10:1 (500 mL x5). The combined organic layer was dried over Na_2SO_4 and concentrated under reduced pressure. The crude residue was purified by column chromatography (silica gel, EtOAc) to afford 54.8 g (68%) of the deprotected compound **S16**.

Physical State: light brown amorphous
^{1}H NMR (600 MHz, $CDCl_3$): δ ^{1}H NMR (600 MHz, $CDCl_3$) δ 7.21 (d, *J* = 7.4 Hz, 2H), 7.06 (t, *J* = 7.4 Hz, 2H), 6.73 (t, *J* = 7.2 Hz, 2H), 6.56 (d, *J* = 5.5 Hz, 2H), 4.75 (s, 2H), 4.36 – 4.06 (m, 6H), 3.60 – 3.49 (m, 2H), 2.45 – 2.34 (m, 4H), 1.21 (t, *J* = 7.1 Hz, 6H).
^{13}C NMR (151 MHz, $CDCl_3$): δ ^{13}C NMR (151 MHz, $CDCl_3$) δ 173.4, 151.2, 129.5, 129.2, 124.9, 118.7, 109.3, 81.0, 64.0, 61.3, 59.8, 42.3, 14.2.
HRMS (ESI-TOF): calculated for $C_{26}H_{30}N_4NaO_4$ $[M+Na]^+$: 485.2165, found: 485.2166.
TLC: R_f = 0.3 (EtOAc only, $Ce_2(SO_4)_3$ in phosphomolybdic acid).
$[\alpha]^{25}_D$: –39.9 (c = 1.0, $CHCl_3$)

Tetratryptomycin A (3) [3]

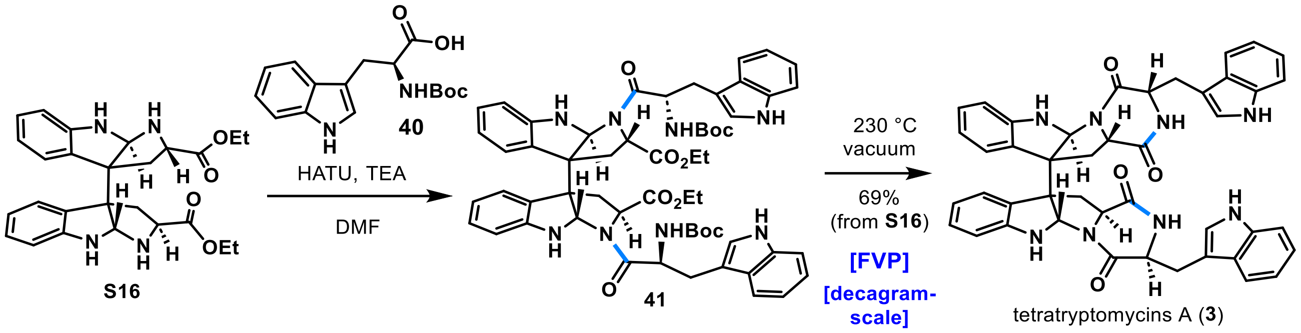

Step 1: To a solution of the compound **S16** (11.7 g, 25.3 mmol, 1.0 eq.), TEA (21.1 mL, 152 mmol. 6.0 eq.) and *N*-Boc-*L*-tryptophan (25.0 g, 82.1 mmol, 3.25 eq.) in DMF (130 mL), and HATU (31.3 g, 82.3 mmol, 3.25 eq.) was added at room temperature. The resulting mixture was stirred for 16 h. Then was slowly quenched by water. The mixture was extracted by DCM/MeOH = 10:1 (300 mL x3). The combined organic layer was washed with brine, dried over Na_2SO_4, and concentrated under reduced pressure. The crude mixture was used for the next step without further purification.

Step 2: The crude mixture in round bottom flask was heated at 230 °C under vacuum condition for 90 min. The crude residue was purified by column chromatography (silica gel, hex/EtOAc = 1:2 to EtOAc) to afford 13.0 g (69% for 2 steps) of the tetratryptomycin A (**3**).

Note: A total of 21 g of tetratryptomycin A (**3**) was prepared by the above procedure.

Physical State: brown solid

^{1}H NMR (600 MHz, *d*$_6$-DMSO): δ 10.69 (s, 2H), 7.72 (s, 2H), 7.54 (d, *J* = 8.0 Hz, 2H), 7.38 (d, *J* = 8.2 Hz, 2H), 7.18 (d, *J* = 7.6 Hz, 2H), 7.08 – 7.00 (m, 6H), 6.95 (t, *J* = 7.6 Hz, 2H), 6.70 (s, 2H), 6.64 (t, *J* = 7.5 Hz, 2H), 6.56 (d, *J* = 7.9 Hz, 2H), 5.09 (s, 2H), 4.32 (d, *J* = 5.8 Hz, 2H), 3.81 (dd, *J* = 11.2, 6.1 Hz, 2H), 3.24 (dd, *J* = 14.9, 4.8 Hz, 2H), 3.04 (dd, *J* = 15.2, 5.7 Hz, 2H), 2.33 (dd, *J* = 13.4, 6.1 Hz, 2H), 2.23 (t, *J* = 12.1 Hz, 2H).

^{13}C NMR (151 MHz, *d*$_6$-DMSO): δ 168.1, 165.5, 151.1, 136.0, 129.2, 127.5, 127.0, 124.7, 123.8, 121.0, 118.5, 118.3, 117.5, 111.4, 109.4, 108.8, 77.3, 58.7, 57.9, 55.1, 36.3, 25.5.

HRMS (ESI-TOF): calculated for $C_{44}H_{38}N_8NaO_4$ $[M+Na]^+$: 765.2914, found: 765.2915.

TLC: R_f = 0.20-0.30 (tailing) (EtOAc only, $Ce_2(SO_4)_3$ in phosphomolybdic acid).

$[\alpha]^{25}_D$: –35.1 (c = 1.0, $CHCl_3$)

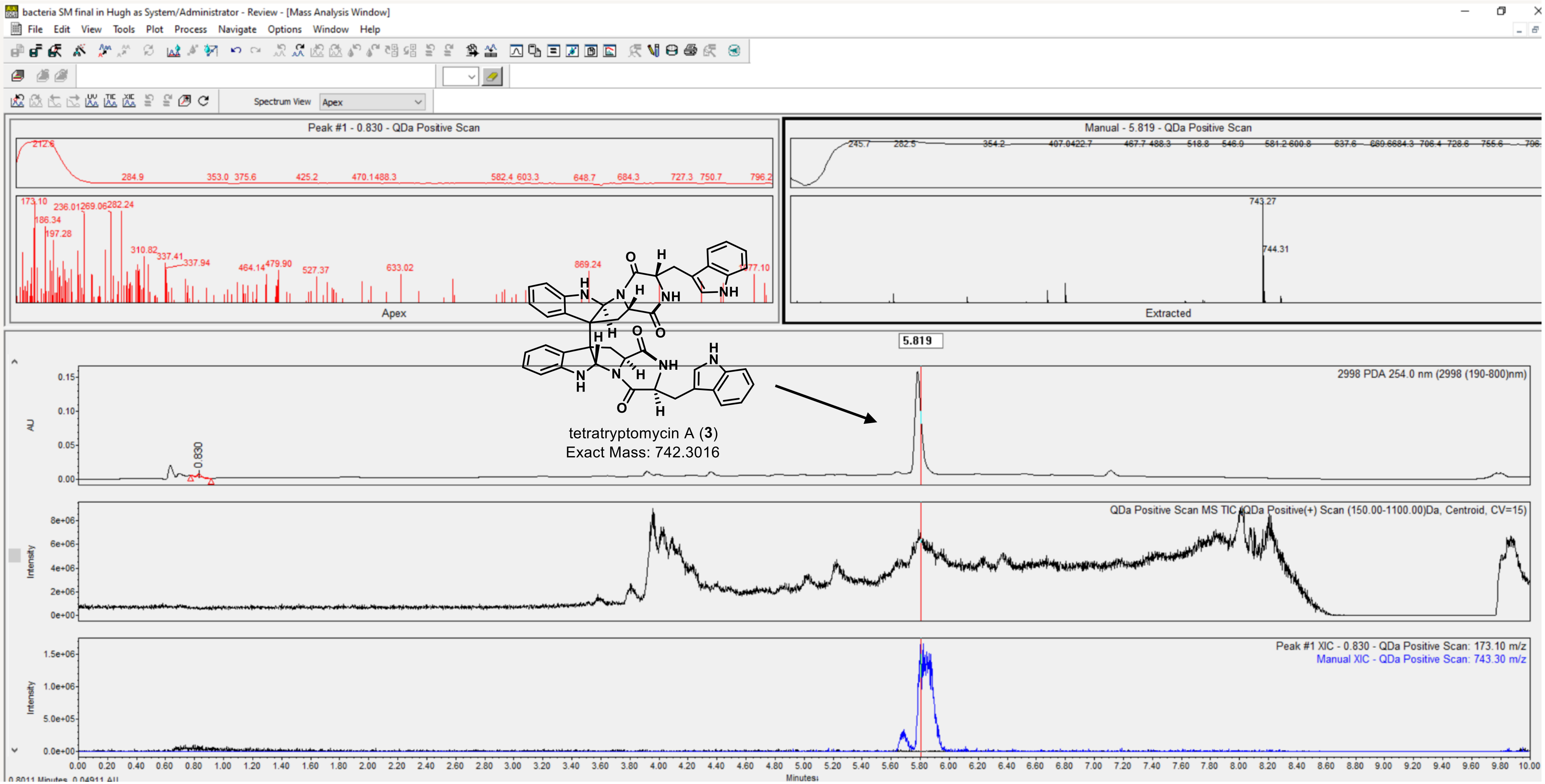

Figure S5. LC-MS chromatogram of tetratryptomycin A (**3**) (Exact Mass: 742.3016) after the manual column chromatography.

NMR Comparison of tetratryptomycin A (3)

Synthetic (This work)<br>^{1}H NMR (600 MHz, d_6-DMSO)	Reported[4]<br>^{1}H NMR (800 MHz, d_6-DMSO)
2.23 (t, *J* = 12.1 Hz, 2H)	2.24 (br, s, 2H)
2.33 (dd, *J* = 13.4, 6.1 Hz, 2H)	2.34 (br, s, 2H)
3.04 (dd, *J* = 15.2, 5.7 Hz, 2H)	3.03 (t, *J* = 8.0, 8.0 Hz, 2H)
3.24 (dd, *J* = 14.9, 4.8 Hz, 2H)	3.25 (dd, *J* = 15.0, 5.7 Hz, 2H)
3.81 (dd, *J* = 11.2, 6.1 Hz, 2H)	3.81 (dd, *J* = 8.0, 8.0 Hz, 2H)
4.32 (d, *J* = 5.8 Hz, 2H)	4.33 (d, *J* = 8.0 Hz, 2H)
5.09 (br, s, 2H)	5.07 (br, s, 2H)
6.56 (d, *J* = 7.9 Hz, 2H)	6.56 (d, *J* = 8.0 Hz, 2H)
6.64 (t, *J* = 7.5 Hz, 2H)	6.65 (d, *J* = 8.0 Hz, 2H)
6.70 (br, s, 2H)	6.69 (br, s, 2H)
6.95 (t, *J* = 7.6 Hz, 2H)	6.95 (t, *J* = 8.0 Hz, 2H)
7.08 – 7.00 (m, 6H)	7.03 – 7.06 (m, 6H)
7.18 (d, *J* = 7.6 Hz, 2H)	7.19 (d, *J* = 8.0 Hz, 2H)
7.38 (d, *J* = 8.2 Hz, 2H)	7.38 (d, *J* = 8.0 Hz, 2H)
7.54 (d, *J* = 8.0 Hz, 2H)	7.54 (d, *J* = 8.0 Hz, 2H)
7.72 (br, s, 2H)	7.68 (br, s, 2H)
10.69 (br, s, 2H)	10.69 (br, s, 2H)

**Synthetic (This work)**<br>**^{13}C NMR (151 MHz, d_6-DMSO)**	**Reported**[4b]<br>**^{13}C NMR (125 MHz, d_6-DMSO)**
25.5	25.4
36.3	36.2
55.1	55.0
57.9	57.8
58.7	58.7
77.3	77.2
108.8	108.7
109.4	109.3
111.4	111.4
117.5	117.4
118.3	118.2
118.5	118.5
120.9	120.9
123.8	123.7
124.7	124.6
127.0	127.0
127.5	127.5
129.2	129.1
136.0	136.0
151.1	151.0
165.5	165.4
168.1	168.1

Cyctetryptomycin A (1) and cyctetryptomycin B (2)

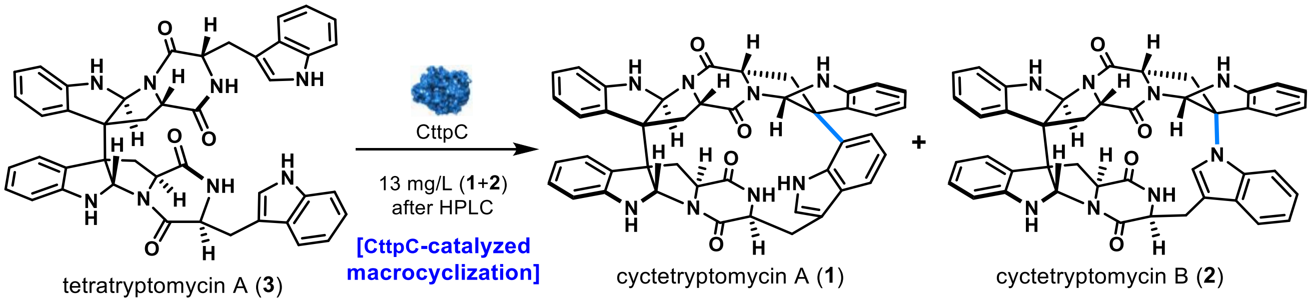

The *S. coelicolor* M1146 harboring pIZ10 were cultured into TSB in 28-30 °C. After 2 days, 1.5% precultures were taken from TSB culture medium and cultured in 50 ml AM6 medium (per liter: 20 g soluble starch, 10 g glucose, 5 g tryptone, 5 g yeast extract, 2 g $CaCO_3$, pH 7.2-7.5) for 3 days in 220 rpm, 30 °C. After 3 days, 3.54 g of tetratryptomycin A (**3**) were added into the 118 flasks (each flask: 30 mg of **3**) of culture medium (volume: each flask 50 ml x 118 flasks = total 5.9 L). The reaction mixture was stirred for 2-3 weeks (depends on season) for additional incubation. The reaction was monitored by LC-MS for each week. After 2-3 weeks, the secondary metabolites were extracted for three times by DCM/MeOH = 10:1 (2000 mL x3) and purified by short column chromatography (silica gel, EtOAc to DCM:MeOH =10:1) to obtain 1.6 g of crude mixture. Then, the crude mixture was purified by preparative HPLC for a couple of times to afford cyctetryptomycin A (**1**) (7.5 mg/L) and cyctetryptomycin B (**2**) (5.5 mg/L). A total of 13 mg/L of cyctetryptomycin A and B were obtained after the preparative HPLC for a couple of times.

Note: For more detail, please see the graphical guide for S. coelicolor M1146 expressing CttpC.

Cyctetryptomycin A (**1**)

Physical State: brown solid

^{1}H NMR (600 MHz, *d*$_6$-DMSO): δ 7.72 (d, *J* = 7.2 Hz, 1H), 7.46 (d, *J* = 6.5 Hz, 1H), 7.35 (d, *J* = 6.9 Hz, 1H), 7.18 (s, 1H), 7.14 (m, 1H), 7.07 (d, *J* = 7.1 Hz, 1H), 7.03 (d, *J* = 7.2 Hz, 1H), 6.98 (br, 1H), 6.82 (m, 1H), 6.68 (m, 4H), 6.28 (t, *J* = 6.6 Hz, 1H), 6.21 (m, 3H), 5.90 (s, 1H), 5.76 (s, 1H), 4.68 (t, *J* = 7.2 Hz, 1H), 4.40 (br, 1H), 4.00 (dd, *J* = 9.8, 4.3 Hz, 1H), 3.39 (m, 2H), 3.06 (d, *J* = 11.6 Hz, 1H), 2.82 (d, *J* = 7.5 Hz, 2H), 2.48 (d, *J* = 7.0 Hz, 1H), 1.98 (d, *J* = 7.4 Hz, 1H), 1.20 (m, 1H), 0.42 (d, *J* = 11.7 Hz, 1H).

^{13}C NMR (151 MHz, *d*$_6$-DMSO): δ 165.3, 163.0, 161.8, 161.6, 150.1, 149.7, 148.2, 131.7, 130.4, 129.3, 128.4, 127.9, 127.6, 127.6, 126.8, 124.6, 124.3, 123.0, 122.5, 122.0, 121.4, 118.8, 118.3, 117.5, 116.7, 113.2, 109.3, 108.8, 107.0, 80.3, 75.2, 75.0, 60.2, 59.1, 56.5, 56.4, 56.2, 55.3, 48.7, 41.6, 38.6, 28.4.

HRMS (ESI-TOF): calculated for $C_{44}H_{36}N_8O_4Na$ $[M+Na]^+$: 763.2757, found: 763.2761

TLC: R_f = 0.15-0.25 (tailing) (EtOAc only, $Ce_2(SO_4)_3$ in phosphomolybdic acid).

$[\alpha]^{25}_D$: –185.0 (c = 0.1, $CHCl_3$)

Cyctetryptomycin B (**2**)

Physical State: colorless solid

^{1}H NMR (600 MHz, *d*$_6$-DMSO): ^{1}H NMR (600 MHz, DMSO) δ 8.84 (s, 1H), 7.64 (d, *J* = 8.0 Hz, 1H), 7.22 (s, 1H), 7.19 (t, *J* = 8.3 Hz, 1H), 7.10 (d, *J* = 4.8 Hz, 1H), 7.07 (t, *J* = 7.2 Hz, 1H), 7.00 (t, *J* = 7.8 Hz, 1H), 6.89 (d, *J* = 8.4 Hz, 1H), 6.84 (t, *J* = 7.6 Hz, 2H), 6.78 (m, 3H), 6.67 (t, *J* = 7.5 Hz, 1H),

6.61 (d, J = 8.3 Hz, 1H), 6.45 (d, J = 4.3 Hz, 1H), 6.34 (ddd, J = 26.4, 13.3, 6.4 Hz, 4H), 6.30 (t, J = 7.4 Hz, 1H), 6.15 (d, J = 3.2 Hz, 1H), 4.72 (m, 1H), 4.69 (t, J = 9.2 Hz, 1H), 3.96 (m, 1H), 3.90 (dd, J = 11.7, 5.1 Hz, 1H), 3.63 (m, 1H), 3.14 (m, 2H), 2.91 (dd, J = 14.2, 8.3 Hz, 1H), 2.63 (dd, J = 11.4, 4.3 Hz, 1H), 2.47 (m, 1H), 1.85 (t, J = 11.9 Hz, 1H), 1.52 (t, J = 12.0 Hz, 1H).

^{13}C NMR (151 MHz, d_6-DMSO): δ 165.1, 163.3, 162.0, 161.4, 150.5, 150.0, 149.3, 135.0, 130.2, 129.6, 129.2, 128.9, 128.6, 128.0, 127.9, 127.0, 123.0, 122.4, 122.2, 121.9, 119.8, 119.2, 119.0, 118.8, 117.6, 117.5, 111.5, 110.9, 109.6, 107.9, 107.7, 81.7, 77.1, 75.5, 73.6, 61.4, 60.2, 55.8, 55.6, 55.2, 54.0, 45.0, 44.0, 42.5, 24.9.

HRMS (ESI-TOF): calculated for $C_{44}H_{36}N_8O_4Na$ $[M+Na]^+$: 763.2757, found: 763.2762

TLC: R_f = 0.15-0.25 (tailing) (EtOAc only, $Ce_2(SO_4)_3$ in phosphomolybdic acid).

$[\alpha]^{25}_D$: –121.6 (c = 0.1, $CHCl_3$)

*Note: Cyctetryptomycin B (**2**) is unstable under the acidic conditions.*

(1) First purification by preparative HPLC: For cyctetryptomycin A (**1**) and cyctetryptomycin B (**2**)

8 ml/min, 38%ACN+0.05%TFA

XBridge BEH C18 OBD Prep Column (100 Å, 5 µm, 19 i.d. x 250 mm; Waters Corporation)

(RT P1: 9.5 min; P2: 15.5 min)

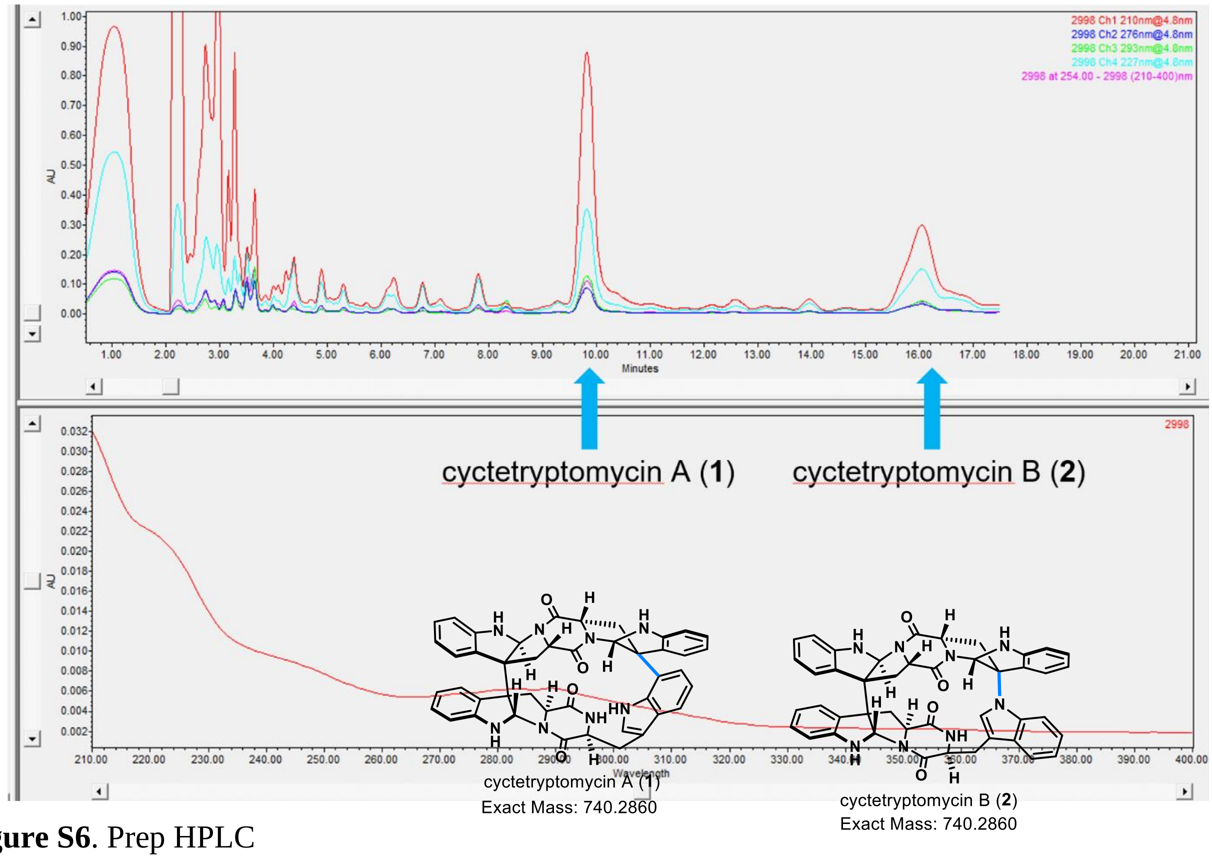

Figure S6. Prep HPLC

chromatogram of cyctetryptomycin A (**1**) (left) and cyctetryptomycin B (**2**) (right).

(2) Second purification by preparative HPLC: For cyctetryptomycin A (**1**)

8 ml/min, 38%ACN+0.05%TFA

XBridge BEH C18 OBD Prep Column (100 Å, 5 µm, 19 i.d. x 250 mm; Waters Corporation)

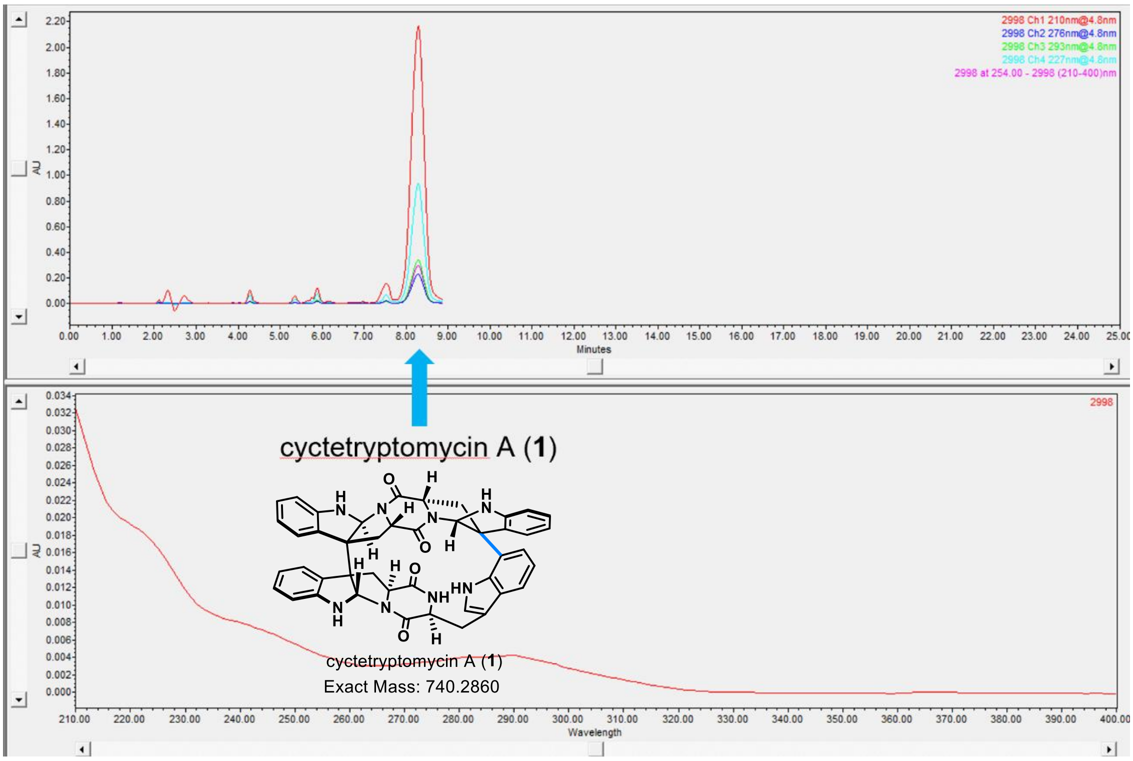

Figure S7. Prep HPLC chromatogram of cyctetryptomycin A (**1**) (Second purification)

(3) Third purification by preparative HPLC: For cyctetryptomycin B (**2**)

3 ml/min, 39%ACN+0.05%TFA

COSMOSIL 5C18-AR-II column (10 i.d. x 250 mm, Nacalai Tesque, Inc)

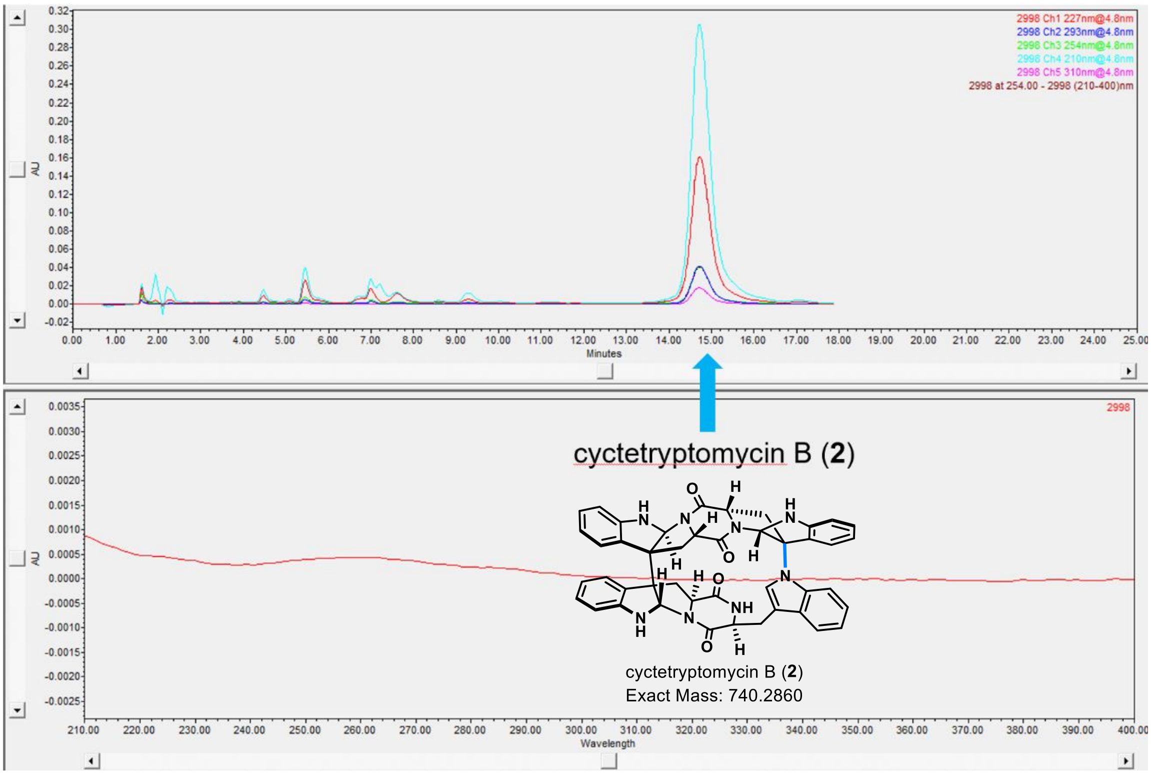

Figure S8. Prep HPLC chromatogram of cyctetryptomycin B (**2**) (Third purification).

(4) Fourth purification by preparative HPLC: For cyctetryptomycin B (**2**)

The purification condition is the following.

CHROMATOGRAPHY REPORT

Column	:	OJ-H
Column size	:	0.46 cm I.D. × 15 cm L
Injection	:	2ul
Mobile phase	:	CO2:ETOH(0.05%DEA)=60:40
Flow rate	:	2.5ml
Wave length	:	UV 220nm
Temperature	:	25℃
Sample solution	:	X mg/ml in ETOH
Sample structure	:	DP

< Chromatogram >

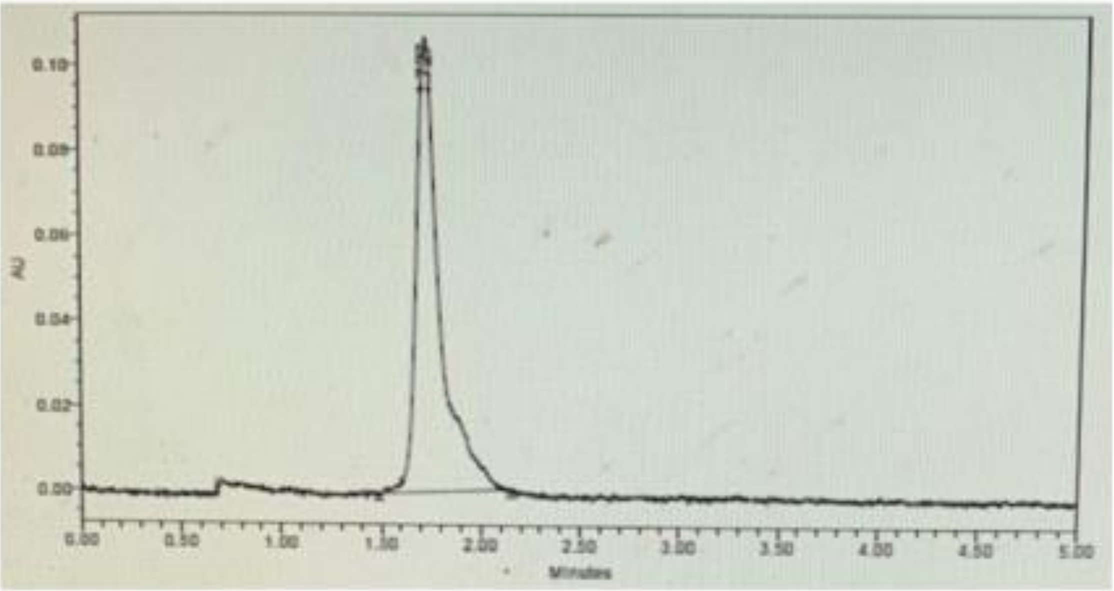

峰号	峰名	保留时间	含量
1	PEAK1	1. 727	100. 000
2	PEAK2	0. 000	0. 000

HRMS (ESI-TOF) of cyctetryptomycin A (1): calculated for $C_{44}H_{36}N_8O_4Na$ $[M+Na]^+$: 763.2757, found: 763.2761

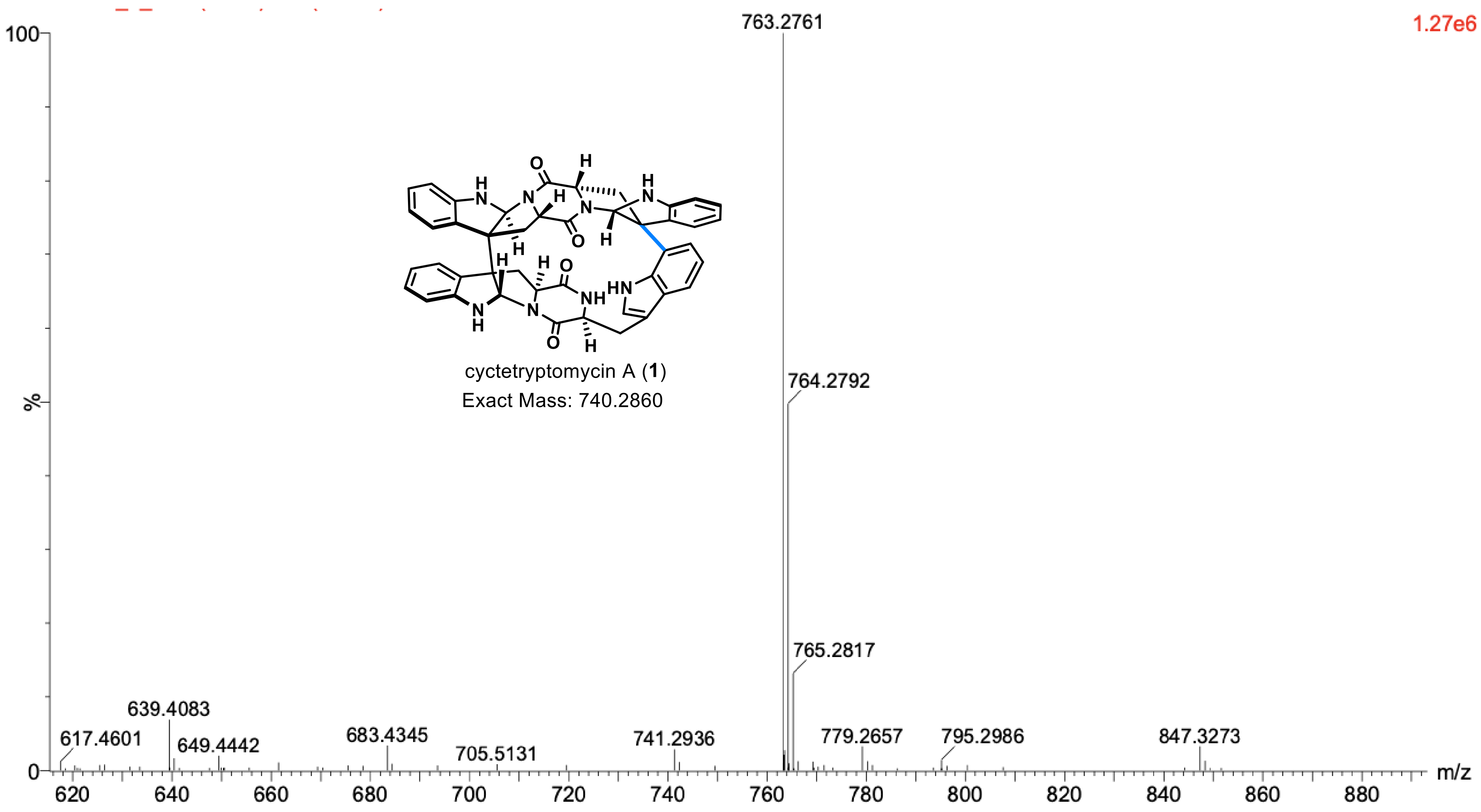

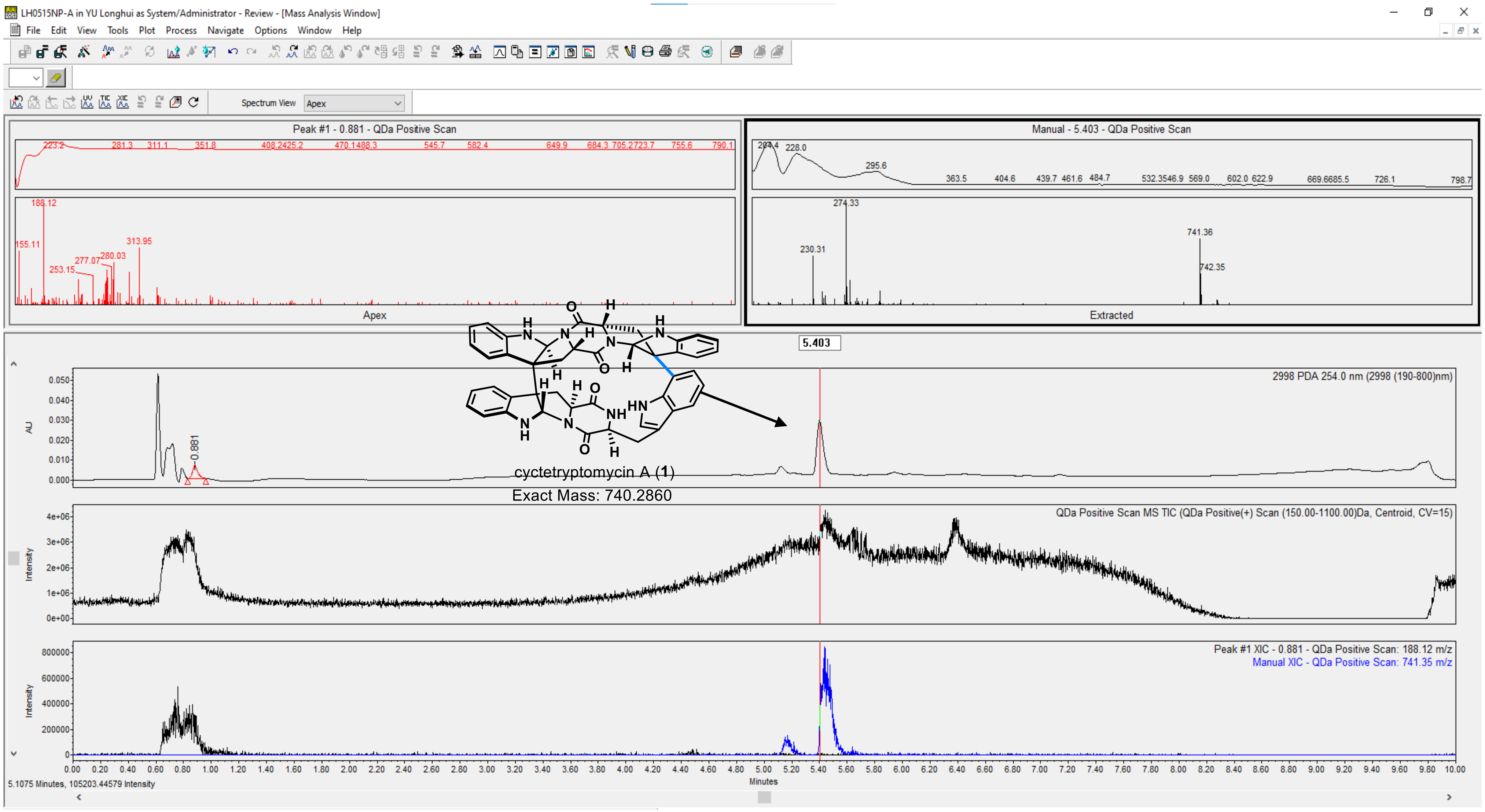

Figure S9. LC-MS chromatogram of cyctetryptomycin A (**1**) (Exact Mass: 740.2860) after the prep HPLC

HRMS (ESI-TOF) of Cyctetryptomycin B (2): calculated for $C_{44}H_{36}N_8O_4Na$ $[M+Na]^+$: 763.2757, found: 763.2762

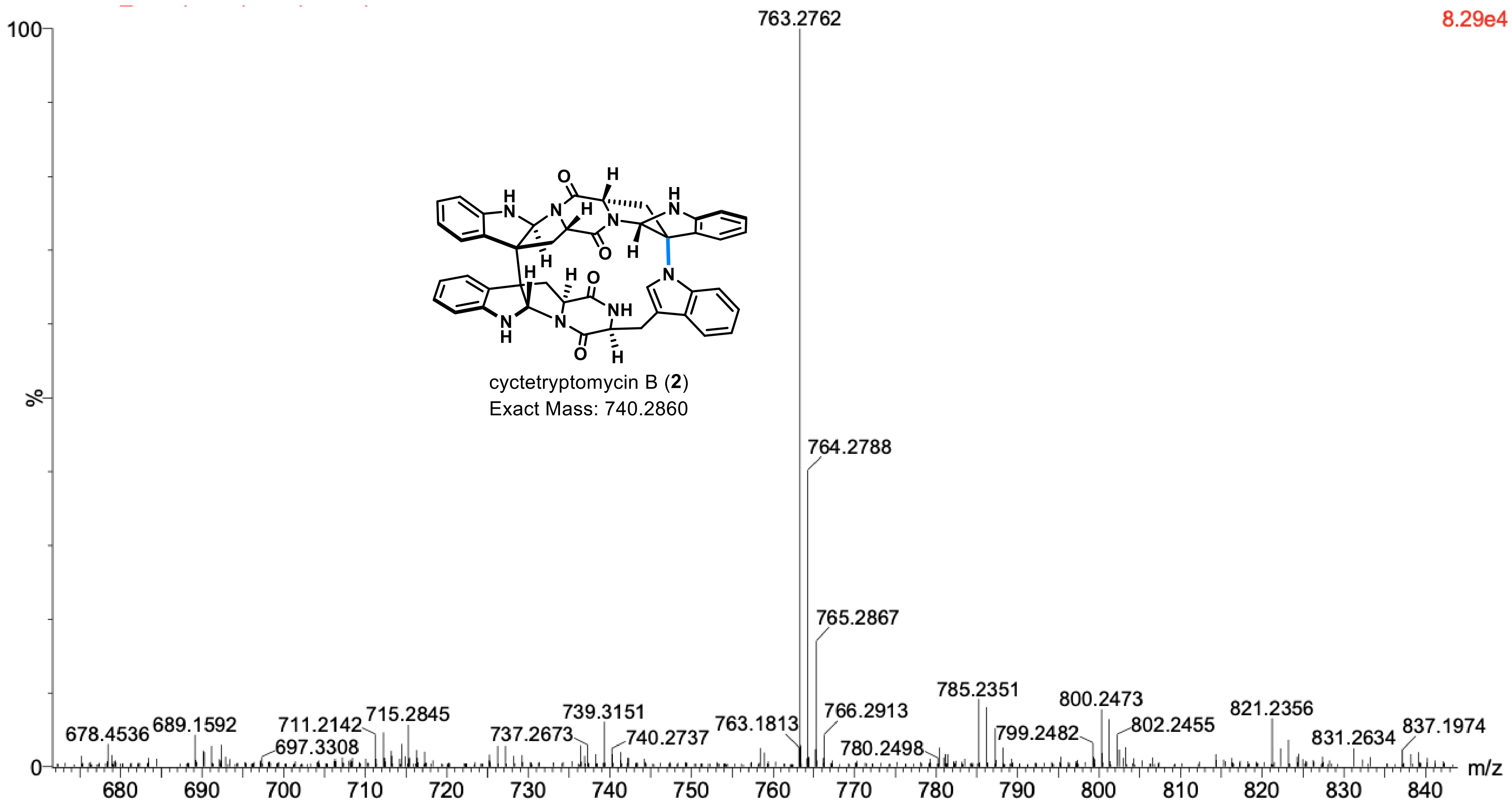

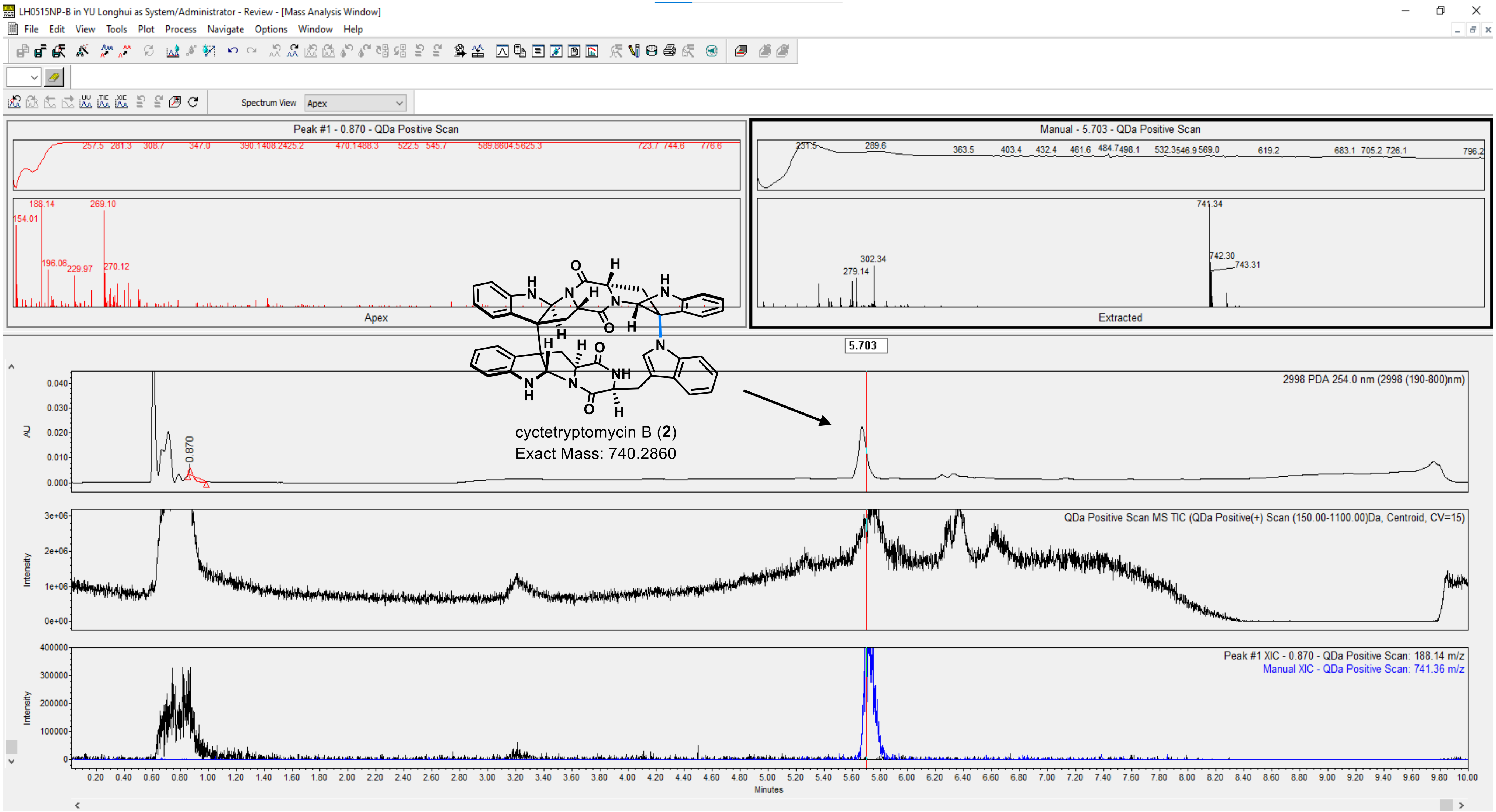

Figure S10. LC-MS chromatogram of cyctetryptomycin B (**2**) (Exact Mass: 740.2860) after the prep HPLC.

NMR Comparison of Cyctetryptomin A (1)

Synthetic (This work)<br>**^{1}H NMR (600 MHz, d_6-DMSO)**	Reported[4a]<br>**^{1}H NMR (800 MHz, d_6-DMSO)**
0.42 (d, *J* = 11.7 Hz, 1H).	0.40 (d, *J* = 12.0 Hz, 1H).
1.20 (m, 1H)	1.18 (m, 1H)
1.98 (d, *J* = 7.4 Hz, 1H)	1.98 (dd, *J* = 12.0, 4.6 Hz, 1H)
2.48 (d, *J* = 7.0 Hz, 1H)	2.46 (dd, *J* = 12.0, 4.6 Hz, 1H)
2.82 (d, *J* = 7.5 Hz, 2H)	2.82 (d, *J* = 8.0 Hz, 2H)
3.06 (d, *J* = 11.6 Hz, 1H)	3.05 (d, *J* = 14.1, 3.9 Hz, 1H)
3.39 (m, 2H)	3.38 (d, *J* = 4.6, 1.4 Hz, 2H)
4.00 (dd, *J* = 9.8, 4.3 Hz, 1H)	3.99 (d, *J* = 12.0, 4.6 Hz, 1H)
4.40 (br, 1H)	4.39 (m, 1H)
4.68 (t, *J* = 7.2 Hz, 1H),	4.68 (td, *J* = 8.1, 1.2 Hz, 1H)
5.76 (s, 1H)	5.76 (s, 1H)
5.90 (s, 1H)	5.90 (s, 1H),
6.21 (m, 3H)	6.21 (d, *J* = 7.9 Hz, 2H), 6.24 (m, 1H)
6.28 (t, *J* = 6.6 Hz, 1H)	6.28 (t, *J* = 7.9 Hz, 1H)
6.68 (m, 4H)	6.68 (m, 4H)
6.82 (m, 1H)	6.82 (d, *J* = 7.9 Hz, 1H)
6.98 (br, 1H)	6.97 (m, 1H)
7.03 (d, *J* = 7.2 Hz, 1H)	7.03 (d, *J* = 7.9 Hz, 1H)
7.07 (m, 1H)	7.06 (m, 1H)
7.14 (m, 1H)	7.14 (t, *J* = 7.9 Hz, 1H)
7.18 (s, 1H)	7.18 (s, 1H)
7.35 (d, *J* = 6.9 Hz, 1H)	7.36 (d, *J* = 7.9 Hz, 1H)
7.46 (d, *J* = 6.5 Hz, 1H)	7.45 (d, *J* = 7.9 Hz, 1H)
7.72 (d, *J* = 7.2 Hz, 1H)	7.71 (d, *J* = 7.9 Hz, 1H)

Synthetic (This work)<br>**^{13}C NMR (151 MHz, d_6-DMSO)**	Reported[4a]<br>**^{13}C NMR (200 MHz, d_6-DMSO)**
28.4	29.0
38.6	39.1
41.6	42.2
48.7	49.3
55.3	55.8
56.2	56.8
56.3	56.9
56.4	57.0

56.5	57.1
59.1	59.7
60.2	60.8
75.0	75.6
75.2	75.8
80.3	80.8
107.0	107.5
108.8	107.5
109.3	109.3
113.2	110.0
116.6	117.3
116.7	117.4
117.5	118.1
118.3	118.9
118.8	119.4
121.4	122.0
122.0	122.6
122.5	123.2
123.0	123.6
124.3	124.9
124.6	125.2
126.7	128.1
127.5	128.1
127.6	128.2
127.9	128.5
128.4	129.0
129.3	129.9
130.4	131.0
131.7	132.4
148.2	148.9
149.7	150.3
150.1	150.7
161.6	162.2
161.8	162.4
163.0	163.5
165.3	165.9

NMR Comparison of Cyctetryptomin B (2)

**Synthetic (This work)**<br>**^{1}H NMR (600 MHz, d_6-DMSO)**	**Reported[4a]**<br>**^{1}H NMR (800 MHz, d_6-DMSO)**
1.52 (t, *J* = 12.0 Hz, 1H)	1.51 (d, *J* = 12.0 Hz, 1H)
1.84 (t, *J* = 11.9 Hz, 1H)	1.83 (t, *J* = 12.0 Hz, 1H)
2.47 (m, 1H)	2.47 (dd, *J* = 12.0, 4.6 Hz, 1H)
2.63 (dd, *J* = 11.4, 4.3 Hz, 1H)	2.62 (dd, *J* = 12.0, 4.6 Hz, 1H)
2.91 (dd, *J* = 14.2, 8.3Hz**,** 1H)	2.90 (dd, *J* = 14.4, 8.4 Hz, 1H)
3.14 (m, 2H)	3.12 (m, 2H)
3.63 (m, 1H)	3.61 (m, 1H)
3.90 (dd, *J* = 11.7, 5.1 Hz, 1H)	3.89 (dd, *J* = 12.9, 5.0 Hz, 1H)
3.96 (m, 1H)	3.96 (m, 1H)
4.69 (t, *J* = 9.2 Hz, 1H)	4.68 (t, *J* = 9.4 Hz, 1H)
4.72 (m, 1H)	4.71 (m, 1H)
6.15 (d, *J* = 3.2 Hz, 1H)	6.14 (s, 1H)
6.30 (t, *J* = 7.4 Hz, 1H)	6.29 (t, *J* = 7.5 Hz, 1H)
6.35 (m, 4 H)	6.33 (m, 1H), 6.35 (s, 1H), 6.36 (m, 2H)
6.45 (d, *J* = 4.3 Hz, 1H)	6.45 (s, 1H)
6.61 (d, *J* = 8.3 Hz, 1H)	6.60 (d, *J* = 8.1 Hz, 1H)
6.67 (t, *J* = 7.5 Hz, 1H)	6.67 (t, *J* = 7.5 Hz, 1H)
6.77 (m, 3H)	6.75 (m, 1H), 6.77 (m, 2H)
6.84 (t, *J* = 7.6 Hz, 2H)	6.83 (d, *J* = 7.5 Hz, 1H), 6.84 (d, *J* = 7.5 Hz, 1H)
6.89 (d, *J* = 8.4 Hz, 1H)	6.89 (d, *J* = 7.5 Hz, 1H)
7.00 (t, *J* = 7.8 Hz, 1H)	7.00 (t, *J* = 8.1 Hz, 1H)
7.07 (t, *J* = 7.2 Hz, 1H)	7.06 (t, *J* = 8.1 Hz, 1H)
7.10 (d, *J* = 4.8 Hz, 1H)	7.18 (t, *J* = 7.5 Hz, 1H)
7.19 (t, *J* = 8.3 Hz, 1H)	7.18 (t, *J* = 7.5 Hz, 1H)
7.21 (s, 1H)	7.21 (s, 1H)
7.64 (d, *J* = 8.0 Hz, 1H)	7.64 (d, *J* = 8.1 Hz, 1H)
8.84 (s, 1H)	8.84 (s, 1H)

**sSynthetic (This work)**<br>**^{13}C NMR (151 MHz, d_6-DMSO)**	**Reported[4a]**<br>**^{13}C NMR (200 MHz, d_6-DMSO)**
24.9	25.3
42.5	42.7
44.0	44.3
45.0	45.3
54.0	54.4

55.2	55.6
55.6	55.9
55.8	56.1
60.2	60.0
61.4	61.9
73.6	74.0
75.5	75.8
77.1	77.5
81.7	82.1
107.7	108.0
107.8	108.2
109.6	110.0
110.9	111.3
111.5	111.8
117.5	117.8
117.6	117.9
118.8	119.2
119.0	119.4
119.2	119.6
119.8	120.2
121.9	122.3
122.2	122.6
122.4	122.7
123.0	123.4
127.9	128.2
128.0	128.3
128.6	129.0
128.9	129.2
129.1	129.5
129.6	129.9
130.2	130.6
135.0	135.4
149.2	149.6
150.0	150.4
150.5	150.9
161.4	161.8
162.0	162.4
163.2	163.6
165.1	165.5

List of gene information and bacterial hosts in this study

Name	Length (aa)	Accession number, source	Proposed function
CttpC	408	PTX68809.1 *Saccharopolyspora hirsuta*	cytochrome P450

** This gene information came from Malit, J. J. article.[5] which annotation by antiSMASH v5.1.2.*

Host	description	source
Saccharopolyspora hirsuta DSM 44795	Wild type strain	DSMZ
Streptomyces coelicolor M1146	Host strain for heterologous expression	[6]
**Plasmids**		
pPWW50A	*Streptomyces* expression vector, Ap^r	[7]
pIZ10	pPWW50A+cttpC expression plasmid, Ap^r	[4]

Protein sequence in this study

>PTX68809.1 cytochrome P450 [Saccharopolyspora hirsuta]
MPPGNDASRAATVLDGLPVPRGNAFGLPTEFARSRDRRPIYRMVYPDGHVGWLVTGYSAARAVLT
DHRFSAEMHRFRFPVPGPGSDPAAQQPGALHPGIFQAMDPPEHTRYRRLLAAKFTTRRMHQLEAKI
EQVTAEQLDQMQRQGPPADLVSTFAMPIPSQVIRHIVGAPDSDWGAFHRHVETMIATDVTFDDVA
AVMRAVPDFLRDLVLRKRRDPGDDVLSDLIATGELDDDELVGLCWLLLENGYTTTANMLALGTL
ALLVNPEQLAALRADPSLADKATEELLRYITIFQFGLFRAAREDVELAGHLIKAGDAVTVLQSTANR
DPAHFTDPDRLDLTRPATGHLSFGHGIHICLGQHLARAEMRIAHTALLRRFPSLHLAATPDELSFRTS
KVIYGVHRLPVSW

** This protein information download from NCBI (https://www.ncbi.nlm.nih.gov/).*

Docking analysis and workflow

The 3D structure of CttpC was predicted by SWISS-Model (http://swissmodel.expasy.org/) using the homology modelling method following by the protocol from SWISS-Model (https://swissmodel.expasy.org/docs/help). The docking analysis of Tetratryptomycin A and CttpC were predicted by software Schrödinger (https://www.schrodinger.com/) relying on the following workflow and the training from Schrödinger (https://www.schrodinger.com/learn/training/schrodinger-online-learning#materials-science-courses).

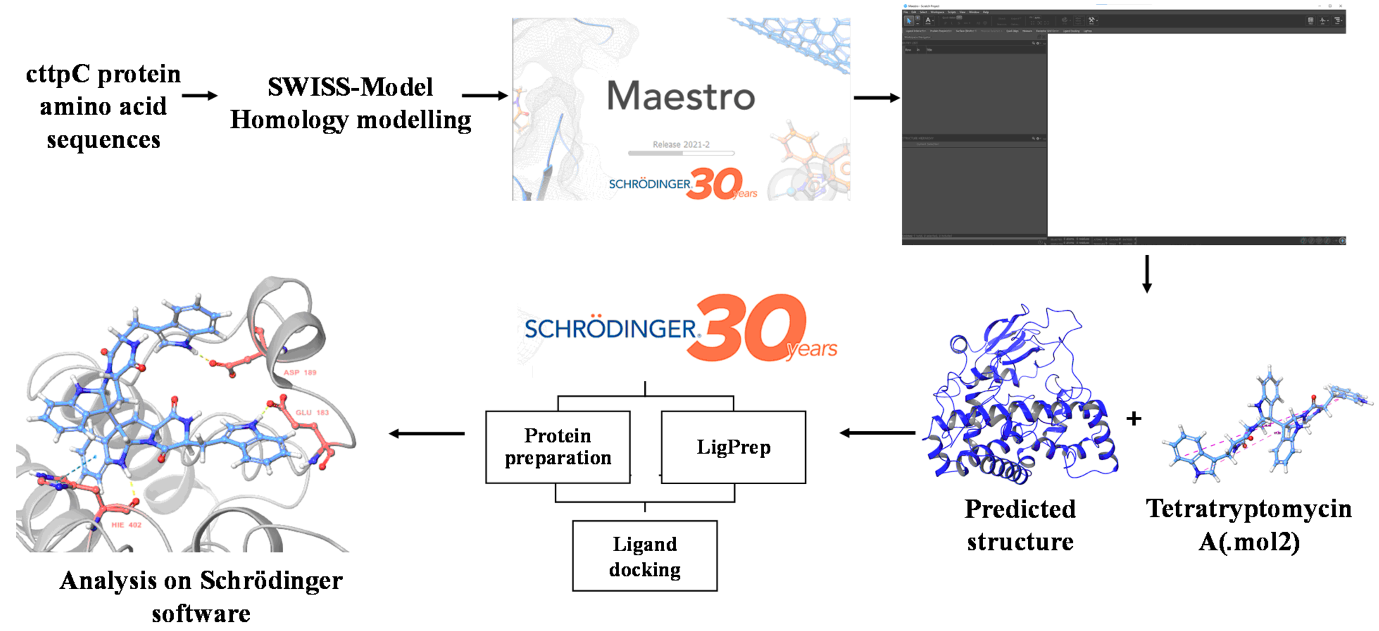

Figure S11. Overview for the process of the docking analysis

Preparation of 3D protein figure using SWISS-model

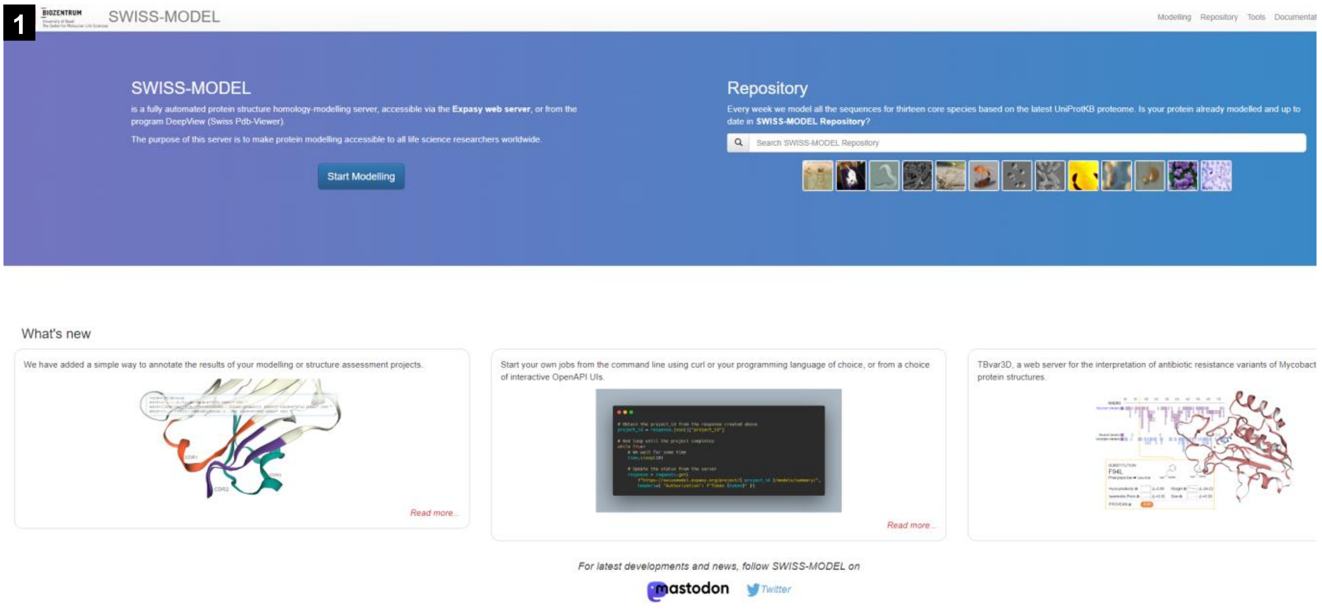

1: Use the SWISS-model website, click [Start Modeling].

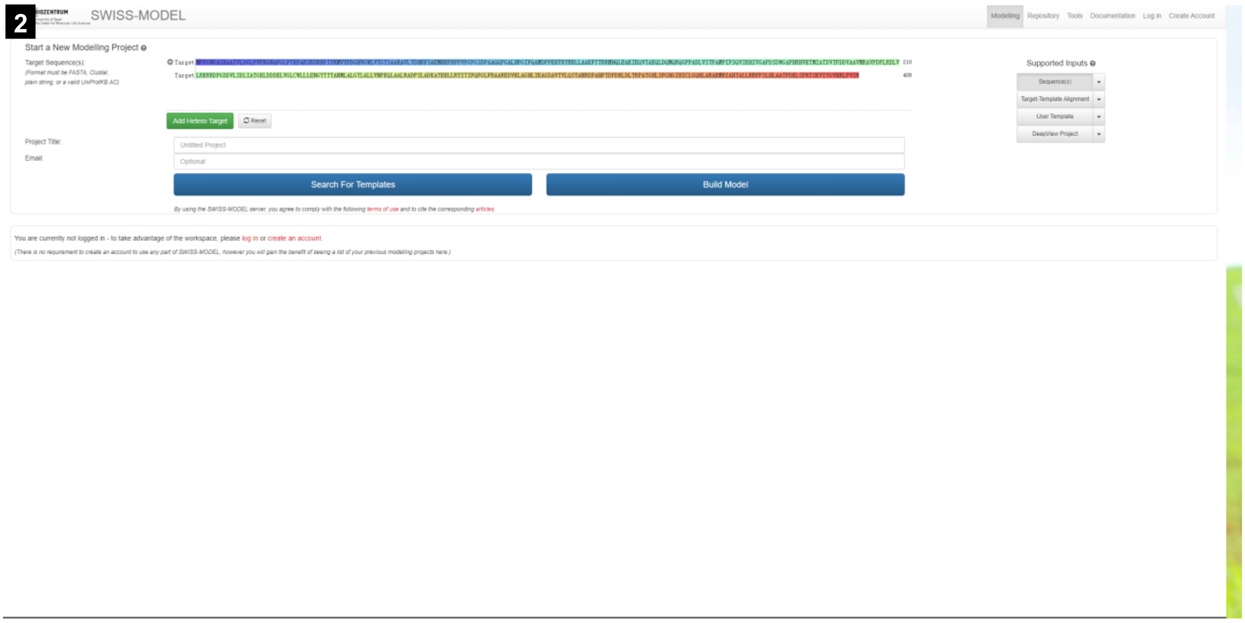

2: Input your amino sequence and click [Build Model]

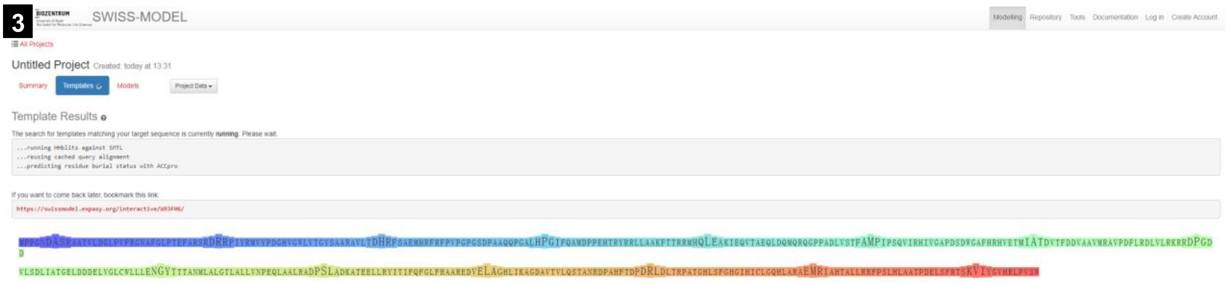

3: The website will search the templates for few minutes based on the amino sequence.

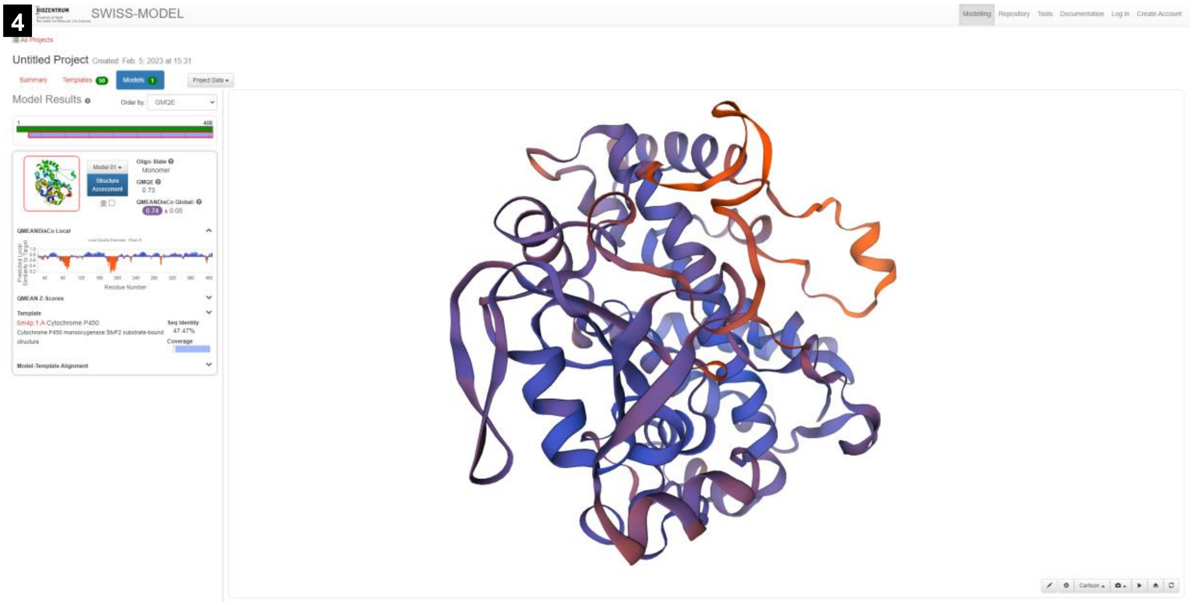

4: The 3D protein model is generated.

Preparation of docking figure using software Schrödinger

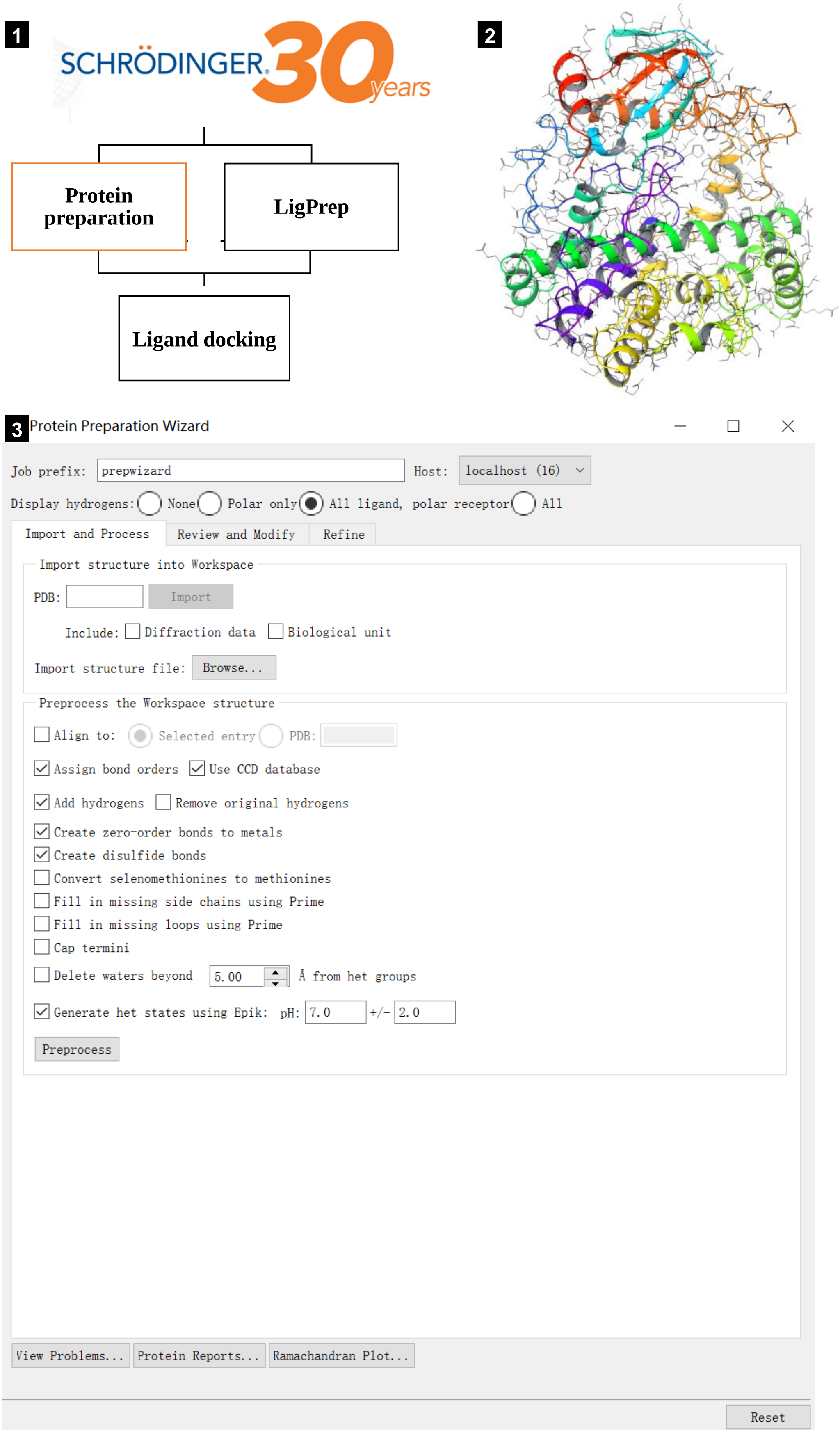

1: Protein preparation section on Schrödinger software.

2: Import the protein structure into Schrödinger.

3: Protein Preparation include preprocess the workspace structure, remove water, H-bond assignment and rest of set up.

4

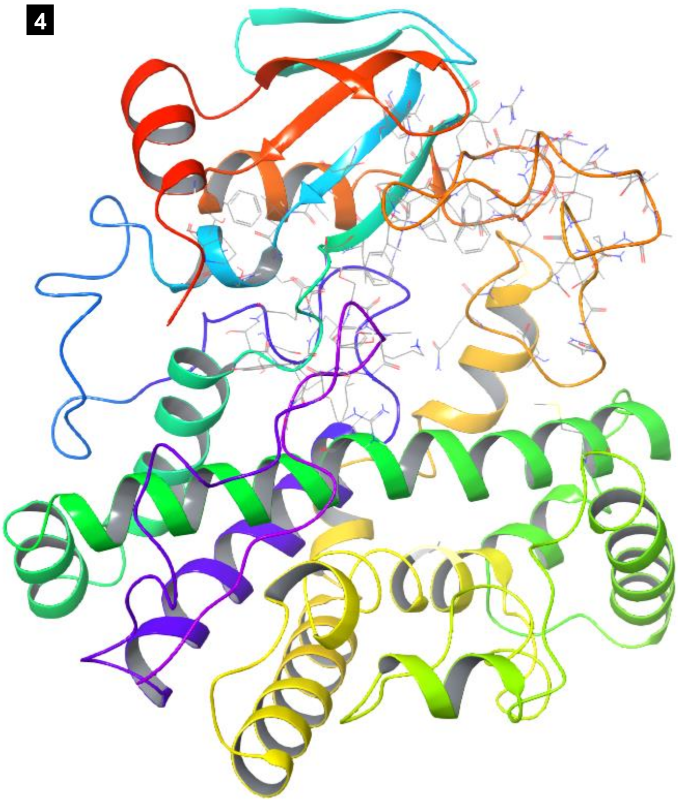

5

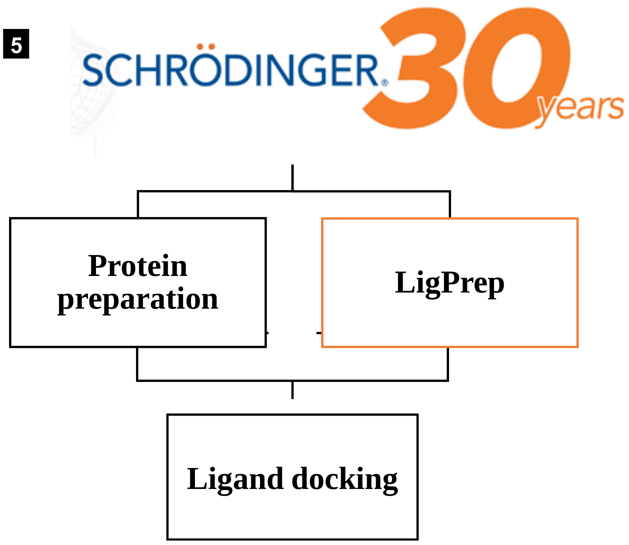

6

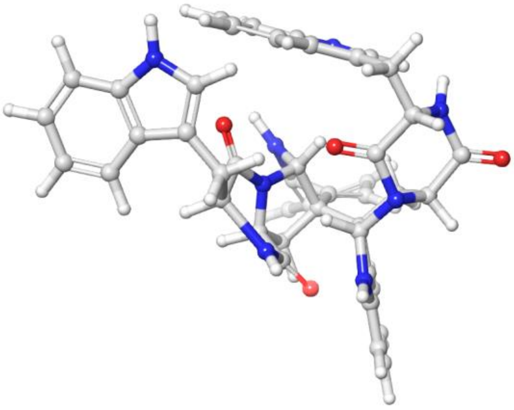

4: Store the result for further docking computation work.

5: Ligand (Staring material) preparation section on Schrödinger software.

6: Input the ligand 3D structure (.mol2) into Schrödinger.

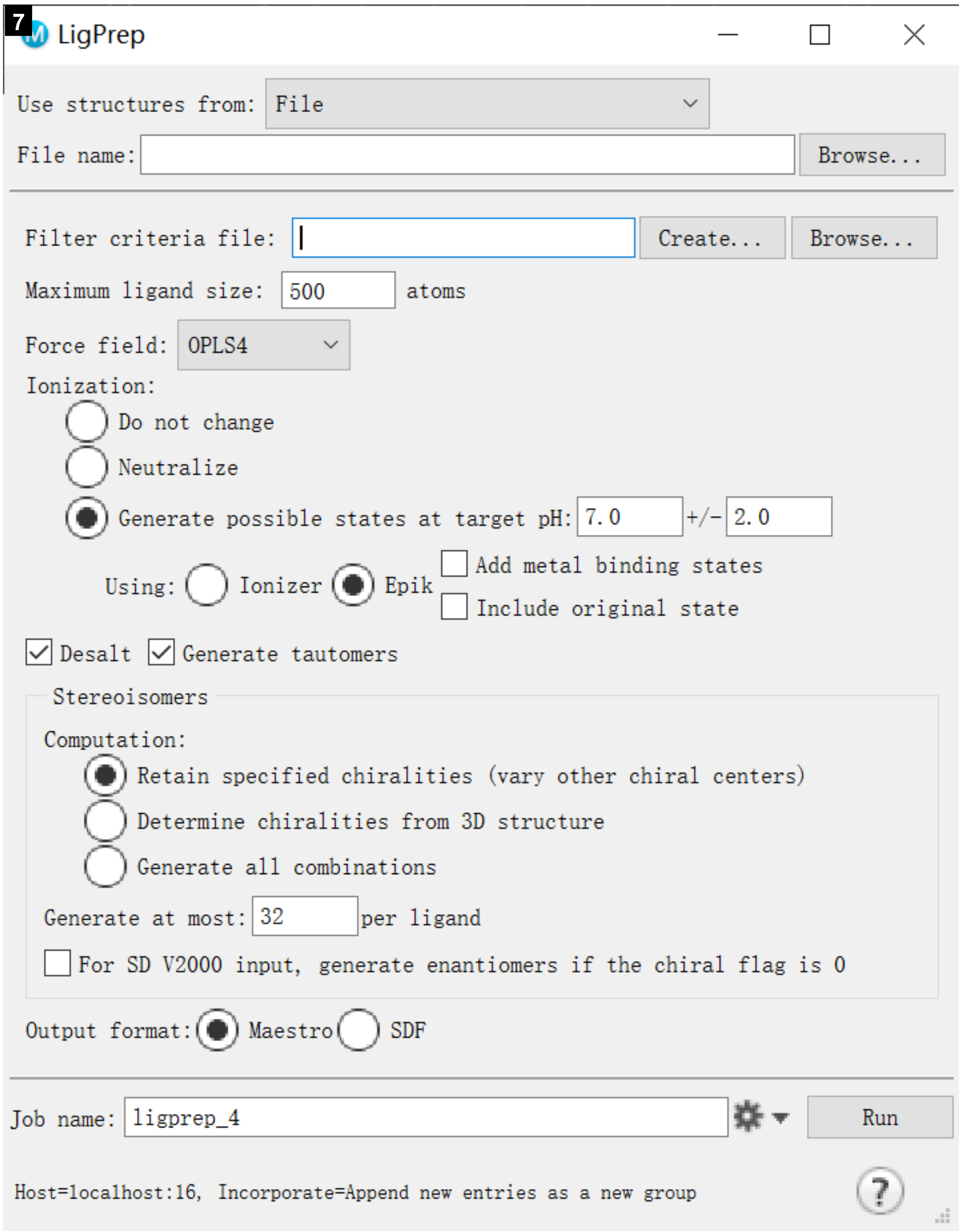

8

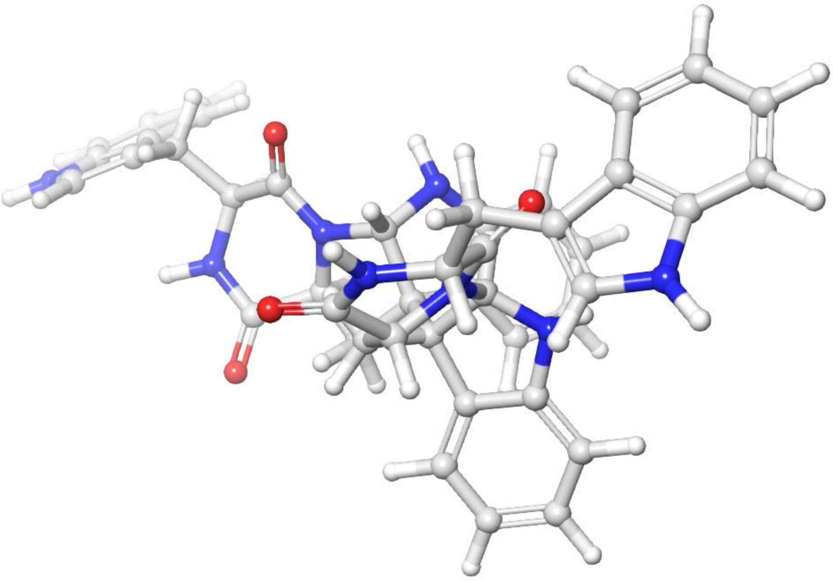

7: Change the setting of ligand for the generating more stable conformations.

8: 2Stable conformations were generated with corrected stereochemistry.

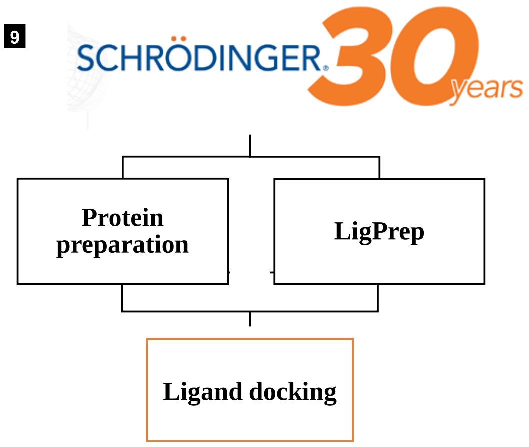

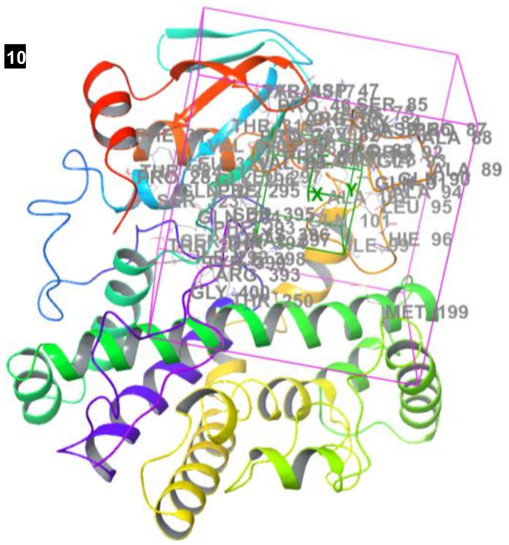

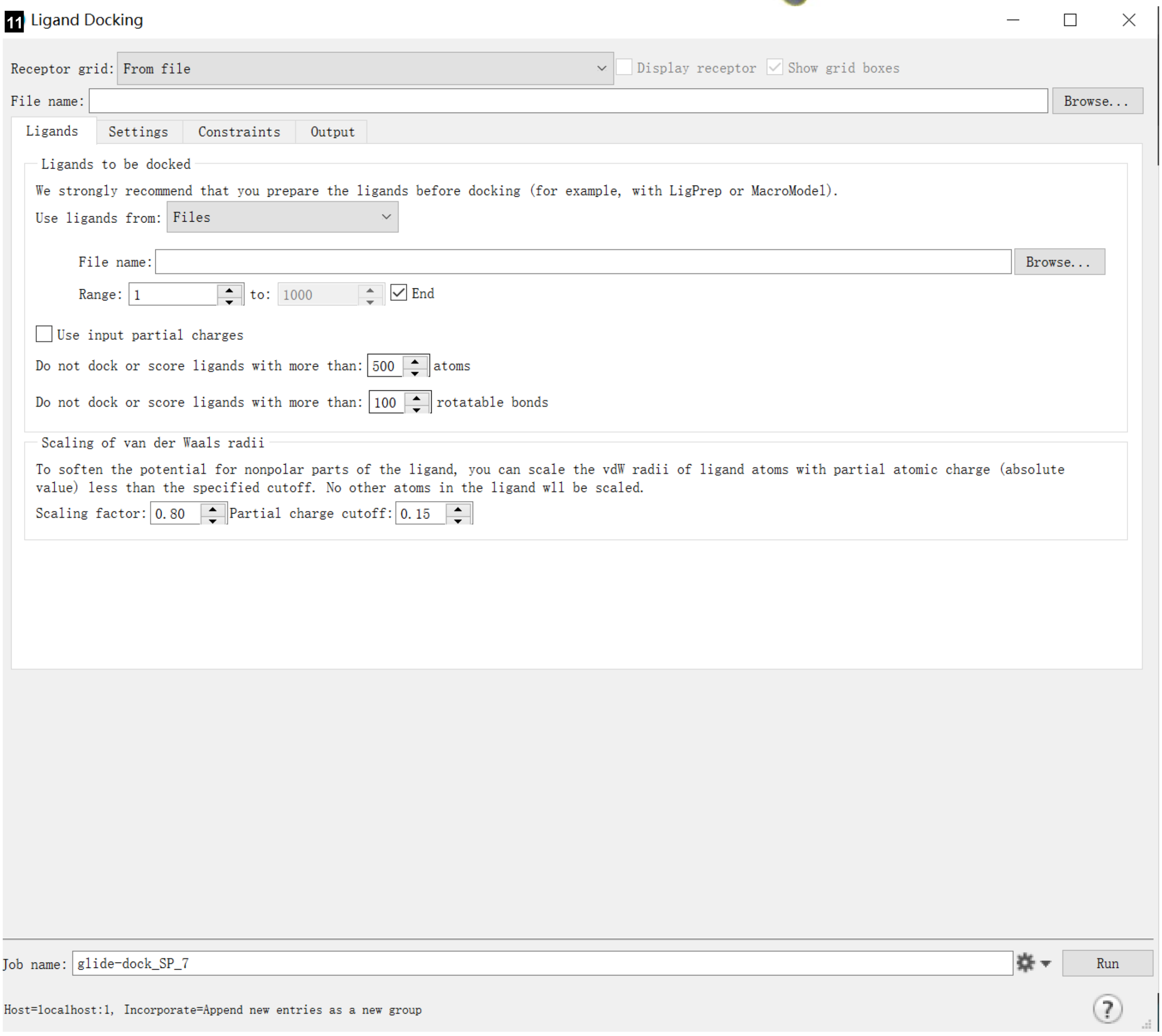

9: Ligand docking section on Schrödinger software.

10: Receptor grid generation using the structure generated from **4**.

11: Start the ligand docking with this setting to generate docking result.

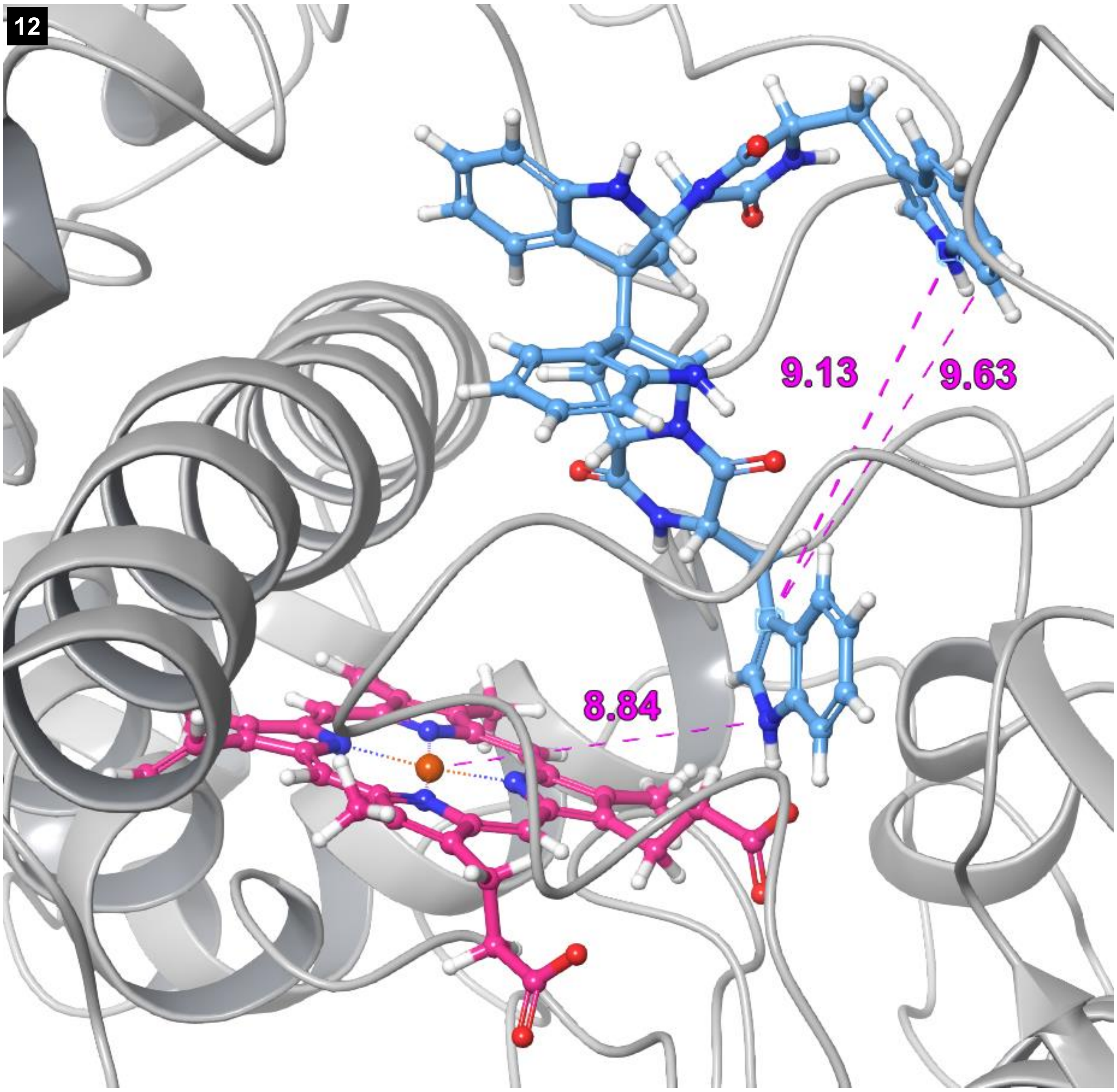

12: Check the result and find out the more reasonable result.

Comparison of Docking Results

Entry	Figure of docking & Docking score ΔG	Entry	Figure of docking & Docking score (ΔG)
**1**	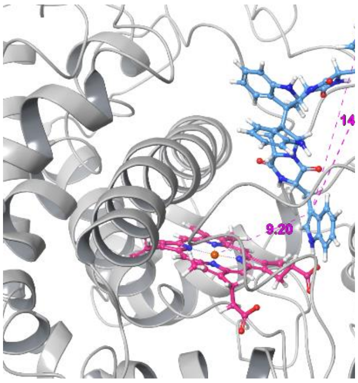 ΔG = -6.483	**2**	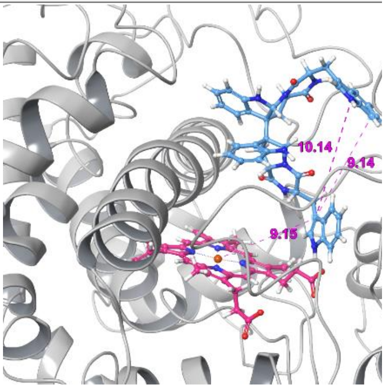 ΔG = -6.977
**3**	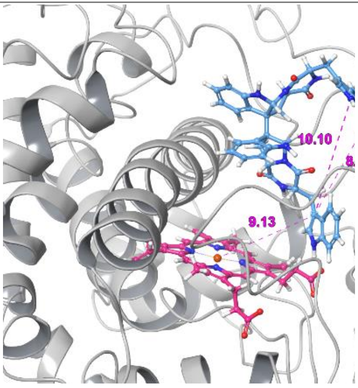 ΔG = -7.133	**4**	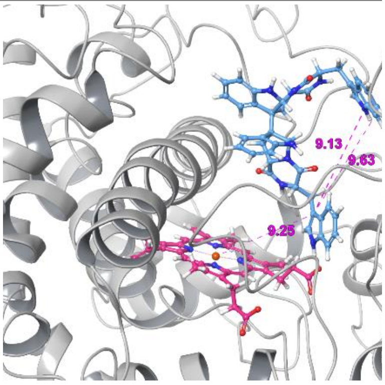 ΔG = -8.087
**5**	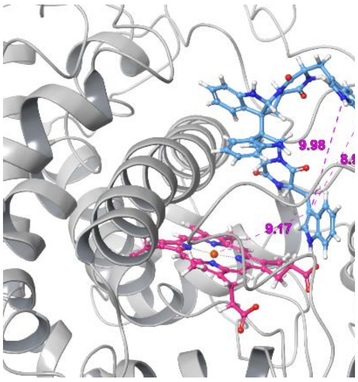 ΔG = -7.284	**6**	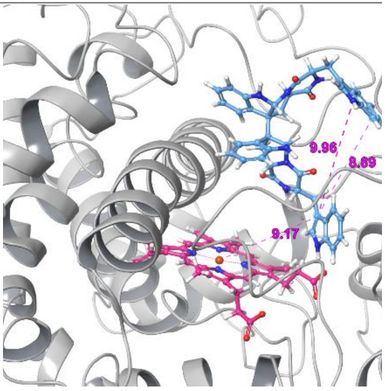 ΔG = -8.222
**7**	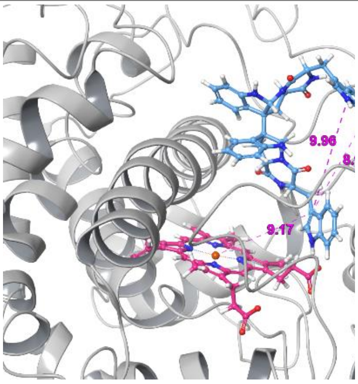 ΔG = -7.369	**8**	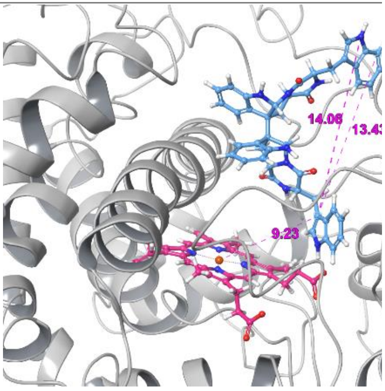 ΔG = -7.434

**9**	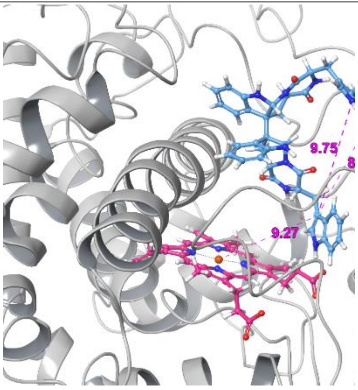<br>ΔG = -6.850	**10**	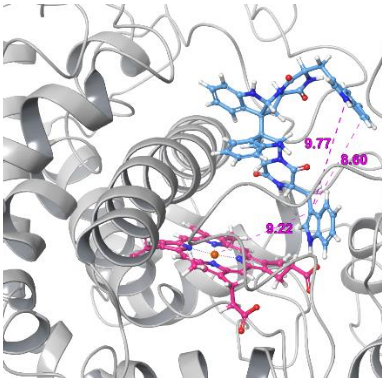<br>ΔG = -6.933
**11**	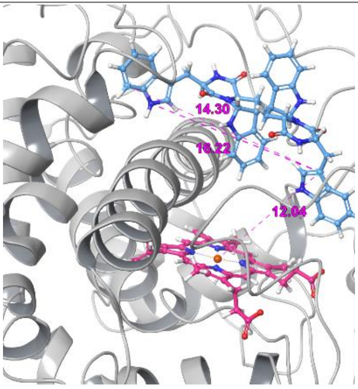<br>ΔG = -7.020	**12**	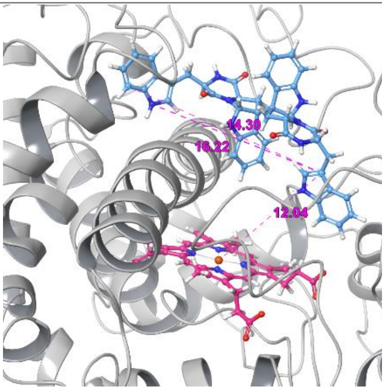<br>ΔG = -7.489
**13**	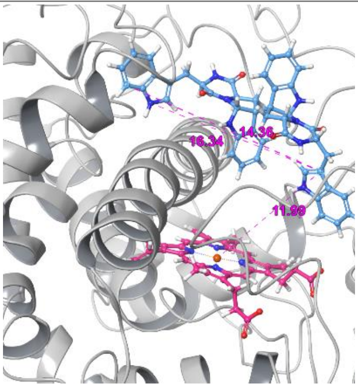<br>ΔG = -7.698	**14**	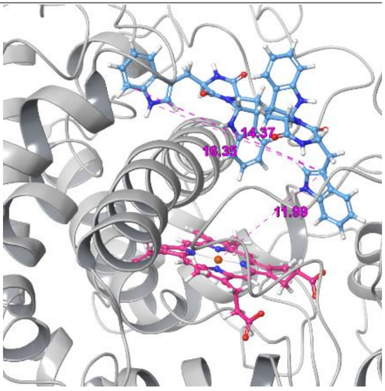<br>ΔG = -7.659
**15**	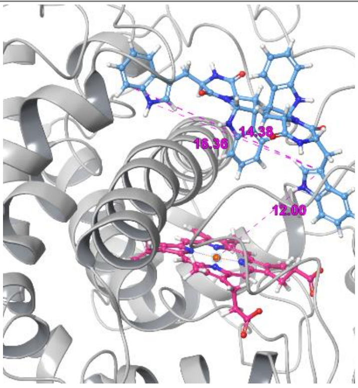<br>ΔG = -7.695	**16**	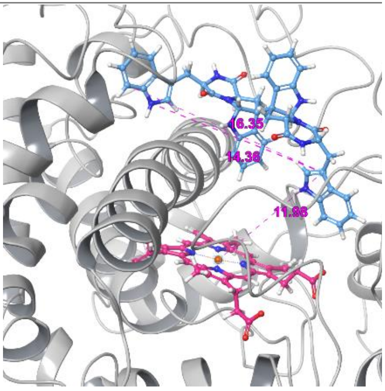<br>ΔG = -7.701

**17**	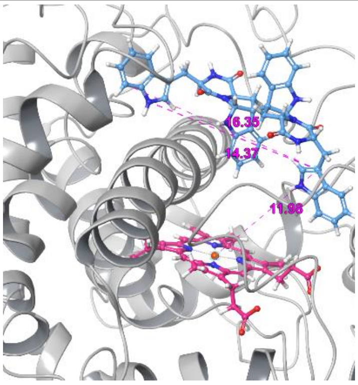<br>ΔG = -7.051	**18**	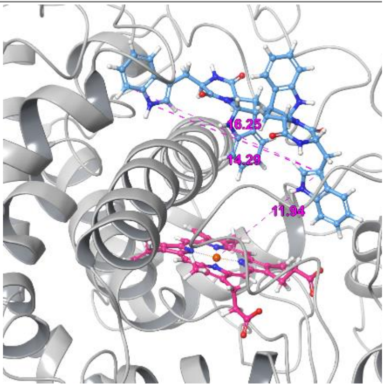<br>ΔG = -7.775
**19**	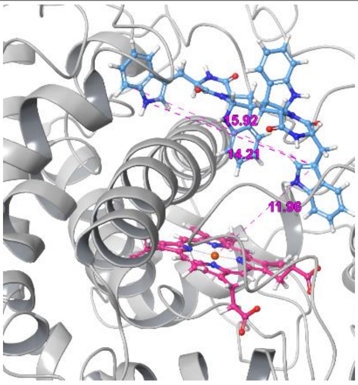<br>ΔG = -8.017	**20**	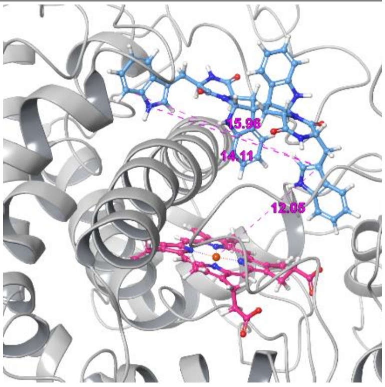<br>ΔG = -6.599
**21**	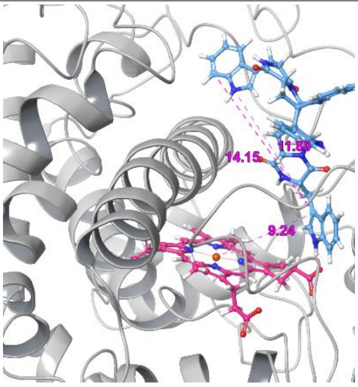<br>ΔG = -8.099	**22**	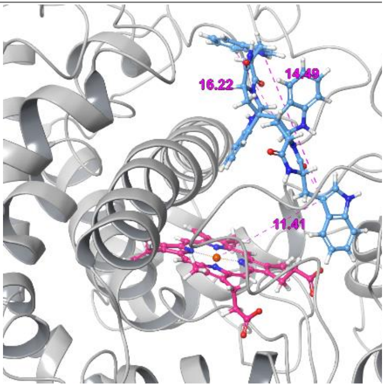<br>ΔG = -6.975

Computational Methods of DFT calculations (1)

All calculations were carried out using the Gaussian 16 program[7] Geometries of molecules and related radical species were optimized at various levels as indicated below. LANL2DZ [7] and DGDZVP [9] basis set were chosen for the application to the zirconium atom. All energetic calculations are on the basis of the ground-state structures, for either the neutral or radical fragments. The BDE were calculated by using the thermochemical scheme supplied by Gaussian as in the following equation: BDE (M–X) = [$D_f H$ (M) + $D_f H$ (X)] – D_f (M–X), in which M–X is the neutral molecule, and M and X are the corresponding radicals.

Table S1. Calculated BDE of Cp_2TiCl_2 at various levels

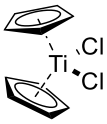

(U)B3LYP[S4]/DGDZVP	62.4
(U)B3LYP/LANL2DZ	54.7
(U)M062X[S5]/DGDZVP	56.6
(U)wB97XD[S6]/DGDZVP	63.6
(U)APFD[S7]/DGDZVP	68.4
**(U)MP2[S8]/DGDZVP**	**84.2**
Experimental data[S9]	93

Table S2. Comparison of BDE of Cp_2MX_2

	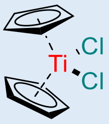	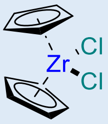	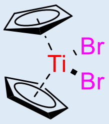	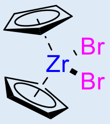
(U)B3LYP/DGDZVP	62.4 kcal/mol	89.0 kcal/mol	51.4 kcal/mol	78.1 kcal/mol
**(U)Mp2/DGDZVP**	**84.2** kcal/mol	**95.5** kcal/mol	**79.2** kcal/mol	**89.5** kcal/mol

Computational Methods of DFT calculations (2)

All geometry optimizations and frequency calculations for reported structures were performed using the B3LYP functional[11] with the def2-SVP basis set[12] using the Gaussian 16 (G16) program.[13] Dispersion interactions were included using Grimme's DFT-D3 correction.[14] The SMD solvent effects were incorporated into all calculations with tetrahydrofuran as the solvent.[15] This level is referred to as SMD(THF)/B3LYP-D3/def2-SVP. All stationary points have been verified, through vibrational analysis, to be minima (zero imaginary frequencies) or transition structures (one imaginary frequency). The character of the normal mode associated with the imaginary frequency has been analyzed to ensure it resembles the reaction under consideration. Energy changes were shown by the use of Gibbs free energies (T = 298.15 K and P = 1 atm). Optimized structures were illustrated using CYLview20.[16] Potential energies were refined by means of single point calculations using the M06 functional[17] with the def2-TZVPP basis set.[12] Dispersion interactions were included using Grimme's DFT-D3 correction.[14] This level is denoted SMD(THF)/M06/def2-TZVPP//SMD(THF)/B3LYP-D3/def2-SVP.

Quantitative analyses of the activation barriers associated with the transition states of the halogen atom transfer (XAT) were obtained by means of the activation strain model (ASM), which involves the decomposition of the electronic energy ΔE into the strain energy $\Delta E_{\mathrm{strain}}$ associated with the structural deformation of $Cp_2Zr(III)Cl$ and the alkyl bromide from their equilibrium geometry and the interaction energy ΔE_{int} between these deformed reactants [Eq. 1].[18] The $\Delta E_{\mathrm{strain}}$ is determined by the rigidity of the reactants and by the extent to which they must deform to achieve the geometry of the transition structure. The ΔE_{int} is usually stabilizing and is related to the electronic structure of the reactants and how they are mutually oriented over the course of the reaction.

$$\Delta E = \Delta E_{\mathrm{strain}} + \Delta E_{\mathrm{int}} \quad (1)$$

Computational Details

Table S3. Energies (electronic energies (*E*), enthalpies (*H*) and Gibbs free energies (G*)* in Hartrees) and imaginary frequencies (in *i* cm^{-1}) of all stationary points computed at SMD(THF)/B3LYP-D3/def2-SVP level of theory are provided. Electronic energies (E^{high} in Hartrees) at SMD(THF)/M06/def2-TZVPP//SMD(THF)/B3LYP-D3/def2-SVP level of theory are also provided.

structure	***E***	***H***	***G***	***E^high^***	***Imag. Freq.***
**MeBr**	-2613.765552	-2613.724746	-2613.752692	-2613.965665	-
**EtBr**	-2653.060158	-2652.989620	-2653.022134	-2653.266414	-
**iPrBr**	-2692.354571	-2692.254807	-2692.290394	-2692.567585	-
**tBuBr**	-2731.648167	-2731.519461	-2731.557584	-2731.869123	-
**TS1-Me**	-3507.901630	-3507.679445	-3507.739188	-3508.265237	320.4*i*
**TS1-Et**	-3547.198561	-3546.947172	-3547.011726	-3547.569296	275.0*i*
**TS1-iPr**	-3586.497000	-3586.215740	-3586.282012	-3586.874779	166.7*i*
**TS1-tBu**	-3625.793038	-3625.482154	-3625.549286	-3626.178859	102.1*i*
**Me radical**	-39.808374	-39.775147	-39.797997	-39.814926	-
**Et radical**	-79.105577	-79.042152	-79.071197	-79.119512	-
**iPr radical**	-118.402594	-118.309521	-118.342710	-118.423786	-
**tBu radical**	-157.699109	-157.576568	-157.613282	-157.727471	-
**Cp2ZrCl**	-894.136234	-893.955174	-894.006251	-894.306024	-
**Cp2ZrClBr**	-3468.107405	-3467.921573	-3467.976229	-3468.463965	-
**4**	-3989.743080	-3989.220239	-3989.316689	-3990.500969	-
**TS1**	-4883.898486	-4883.193178	-4883.315761	-4884.816440	89.0*i*
**Int1**	-1415.806573	-1415.288394	-1415.381784	-1416.374447	-

Cartesian Coordinates

MeBr

Br	0.00000000	0.00000000	0.42493400
C	0.00000000	0.00000000	-1.53879900
H	0.00000000	1.04242300	-1.87996500
H	-0.90276500	-0.52121200	-1.87996500
H	0.90276500	-0.52121200	-1.87996500

EtBr

Br	-0.80252300	-0.06666300	-0.00000100
C	1.03905000	0.68550400	0.00002200
C	2.09251400	-0.40177200	0.00003500
H	1.08823000	1.32032400	0.89491400
H	1.08811600	1.32016700	-0.89499900
H	3.09105100	0.07059200	-0.00035100
H	2.01565000	-1.04019800	-0.89416000
H	2.01588900	-1.04006000	0.89429100

***i*PrBr**

Br	1.02619800	0.00007900	-0.02511000
C	-0.94316900	0.00003200	0.41121000
C	-1.57054800	1.27326400	-0.12758900
H	-0.94341500	0.00010000	1.51011500
C	-1.56995900	-1.27358800	-0.12752000
H	-2.63686600	1.29289800	0.15957600
H	-1.51133100	1.31256200	-1.22724600
H	-1.08864000	2.17297600	0.28378800
H	-2.63640000	-1.29375900	0.15926800
H	-1.08763800	-2.17302400	0.28392300
H	-1.51057300	-1.31277300	-1.22716900

***t*BuBr**

Br	1.18447900	-0.00011700	0.00004400
C	-0.86254000	0.00008200	-0.00003000
C	-1.30125200	-0.60793600	1.32692400
C	-1.30106200	-0.84522300	-1.19005300
C	-1.30044200	1.45355600	-0.13701400
H	-2.40527800	-0.62290100	1.35891400
H	-0.94234800	-0.01589800	2.18240600
H	-0.94210700	-1.64238300	1.43729600

H	-2.40445500	1.48956400	-0.13996800
H	-0.94135500	1.89799800	-1.07760000
H	-0.94075700	2.06619900	0.70350500
H	-2.40510100	-0.86524400	-1.21952100
H	-0.94244300	-1.88220600	-1.10461600
H	-0.94115000	-0.42389900	-2.14092100

TS1-Me

Zr	-0.54395600	0.00285400	0.13518500
C	-0.20648400	-2.49996100	-0.07558500
C	-0.20285400	2.50565600	0.04799600
C	-0.65706400	-2.04853800	-1.35180300
C	-1.28283300	-2.37523300	0.84118900
C	-1.30992700	2.34448800	0.92253200
C	-0.61110300	2.10859900	-1.26047500
C	-2.00501200	-1.62935900	-1.20961400
C	-2.39392700	-1.83776800	0.14670300
C	-2.39720400	1.83950200	0.16691300
C	-1.96358300	1.68620000	-1.18151300
H	0.78975900	-2.87541400	0.15568600
H	0.78497900	2.87101000	0.32708100
H	-0.07763200	-2.04706300	-2.27415100
H	-1.24720100	-2.62054400	1.90176800
H	-1.31182100	2.54622900	1.99316100
H	-0.00253900	2.14745100	-2.16282500
H	-2.64035600	-1.23362500	-2.00152300
H	-3.36786900	-1.60359000	0.57824100
H	-3.38481900	1.58754500	0.55524800
H	-2.57121600	1.32444700	-2.01051600
C	3.75186200	-0.01418400	0.53644100
Cl	0.54371900	-0.08024500	2.39672900
Br	1.98275000	0.02088900	-0.87953800
H	4.27938600	0.91826900	0.30894700
H	4.28638000	-0.92789200	0.25553600
H	3.25051000	-0.04433200	1.50866800

TS1-Et

Zr	-0.68747900	0.09032100	0.10467600
C	-1.02556600	-2.41445900	0.12565400
C	0.23796500	2.38462000	-0.57042800

C	-1.45813100	-1.95505500	-1.15489400
C	-1.94867200	-1.94551200	1.09716400
C	-0.43259300	2.60364500	0.65605900
C	-0.73001100	2.01058900	-1.54980800
C	-2.63432300	-1.18345000	-0.95961700
C	-2.94480600	-1.19024900	0.43318200
C	-1.81751600	2.36031700	0.45632000
C	-2.00159000	2.01220000	-0.91424400
H	-0.14308900	-3.02139300	0.32549000
H	1.31101900	2.47168800	-0.73423100
H	-0.98742700	-2.17902800	-2.11130600
H	-1.87956400	-2.10889800	2.17169200
H	0.04035300	2.86968300	1.59977900
H	-0.53422400	1.79814100	-2.60095000
H	-3.21564400	-0.69531900	-1.74136600
H	-3.77901600	-0.67104700	0.90674800
H	-2.60010700	2.43977200	1.21167000
H	-2.95515100	1.78639800	-1.39069100
C	3.45665200	-0.70633800	0.39217700
Cl	0.43762300	-0.05900400	2.36047900
Br	1.67524600	-0.61968400	-1.01225600
C	4.05010200	0.66630500	0.45825700
H	4.05582500	-1.47817300	-0.10557400
H	2.94815900	-1.05772400	1.29628700
H	4.93048100	0.65726600	1.13117900
H	3.33189200	1.39578300	0.86514600
H	4.39340100	1.01632900	-0.52901600

TS1-^{i}Pr

Zr	-0.85848400	0.00039300	0.12038800
C	-0.51702900	-2.50088600	-0.10137900
C	-0.51602300	2.50214400	-0.18910400
C	-1.18873000	-2.06953600	-1.28542200
C	-1.41312200	-2.36425400	0.99006000
C	-1.33496900	2.39620800	0.96203500
C	-1.26268900	2.03497600	-1.31322000
C	-2.49023400	-1.64397400	-0.90791800
C	-2.62878300	-1.83190900	0.49902200
C	-2.57970500	1.84688500	0.57063700
C	-2.53908700	1.62675900	-0.83849400

H	0.50610600	-2.87020600	-0.04326400
H	0.50921200	2.86938700	-0.20917300
H	-0.78430100	-2.08625000	-2.29688700
H	-1.18633700	-2.58767600	2.03130800
H	-1.03756400	2.64670700	1.97886500
H	-0.92550700	2.02345400	-2.34934600
H	-3.25939700	-1.26548200	-1.58066500
H	-3.51131100	-1.59149600	1.09322600
H	-3.42107300	1.62871400	1.22951100
H	-3.35320800	1.23433500	-1.44712100
C	3.47242000	-0.01102800	-0.14117800
Cl	0.45671200	0.00774900	2.30214900
Br	1.52652000	0.00388100	-1.24653100
C	3.52715400	1.27306700	0.63963600
H	4.12701000	-0.01871500	-1.02305700
C	3.50461200	-1.29295200	0.64453800
H	4.49803900	1.32349500	1.17043900
H	2.72660400	1.31375900	1.39326700
H	3.45742300	2.15608500	-0.01382500
H	4.47743900	-1.36137000	1.16974400
H	3.41295900	-2.17697200	-0.00484800
H	2.70807500	-1.31406800	1.40309500

TS1-^{t}Bu

Zr	-1.08221400	-0.00010800	0.12617700
C	-0.73317000	-2.49227900	-0.01431900
C	-0.73346500	2.49228200	-0.01170700
C	-1.17259500	-2.07234100	-1.30708000
C	-1.82285500	-2.35667800	0.88726700
C	-1.82384800	2.35571900	0.88892100
C	-1.17183900	2.07343200	-1.30516900
C	-2.52528300	-1.65890400	-1.18729100
C	-2.92829400	-1.84129000	0.16805300
C	-2.92866800	1.84092500	0.16831100
C	-2.52457900	1.65973200	-1.18684200
H	0.26152700	-2.85913600	0.23673800
H	0.26102800	2.85890800	0.24049100
H	-0.58592100	-2.09505800	-2.22444300
H	-1.79852800	-2.57808800	1.95363100
H	-1.80044800	2.57621200	1.95550000

H	-0.58451000	2.09716200	-2.22208000
H	-3.15406900	-1.28305400	-1.99412500
H	-3.90813700	-1.60238100	0.58352900
H	-3.90883100	1.60153700	0.58276400
H	-3.15264000	1.28458000	-1.99456400
C	3.35151200	-0.00033200	0.14205800
Cl	0.04580100	-0.00114500	2.40189100
Br	1.46000700	0.00106200	-1.02200800
C	3.29187800	1.26946800	0.95898200
C	4.41112900	0.00059400	-0.93685800
C	3.29148000	-1.27173400	0.95644100
H	4.21625000	1.33719300	1.56463700
H	2.43208700	1.26557200	1.64398500
H	3.24445700	2.16444800	0.31959600
H	4.21582200	-1.34094800	1.56196900
H	3.24379400	-2.16542400	0.31527000
H	2.43167800	-1.26894100	1.64144500
H	5.40519200	-0.00003100	-0.44986200
H	4.35138800	0.89728700	-1.57312100
H	4.35112200	-0.89479500	-1.57492800

Me radical

C	0.00000000	0.00000000	0.00016900
H	0.00000000	1.09339300	-0.00033700
H	0.94690600	-0.54669700	-0.00033700
H	-0.94690600	-0.54669700	-0.00033700

Et radical

C	0.79455300	0.00001700	-0.02438800
C	-0.69297900	0.00000500	-0.00207900
H	1.35739800	0.93634800	0.05260500
H	1.35735600	-0.93635200	0.05261300
H	-1.09225100	-0.00055500	1.03672900
H	-1.11585000	-0.89463500	-0.49201700
H	-1.11609900	0.89506100	-0.49113000

^{i}Pr radical

C	0.00000200	0.53736200	-0.05701700
C	1.29616500	-0.19793700	0.00304800
C	-1.29616400	-0.19792800	0.00304900

H	-0.00003200	1.61873900	0.12508300
H	1.49170000	-0.60913100	1.01910300
H	2.15582500	0.44479700	-0.24985800
H	1.30409700	-1.06948600	-0.67902600
H	-1.49130900	-0.60985000	1.01889100
H	-1.30439900	-1.06904600	-0.67958500
H	-2.15589800	0.44499700	-0.24909200

^{t}Bu radical

C	0.00005200	-0.00007500	0.17247000
C	1.37578700	-0.55854800	-0.01597900
C	-0.20409500	1.47062700	-0.01594900
C	-1.17166300	-0.91208600	-0.01595800
H	1.62934100	-0.66127000	-1.09619100
H	1.47866400	-1.56634800	0.42336700
H	2.15192400	0.09233900	0.42357700
H	-0.24302400	1.74142700	-1.09614300
H	0.61772100	2.06353800	0.42259800
H	-1.15541000	1.81756400	0.42440600
H	-1.38770700	-1.07995500	-1.09615100
H	-2.09569300	-0.49701100	0.42353500
H	-0.99630800	-1.90979100	0.42349800

Cp_2ZrCl

Zr	-0.00143500	0.09936400	-0.20434700
C	2.50028800	0.07461500	-0.71707000
C	-2.49290700	-0.01226900	-0.79294300
C	2.00383400	-1.23377900	-0.98892100
C	2.40646200	0.31162300	0.67569100
C	-2.43457400	0.36647100	0.56888400
C	-1.97163200	-1.32946800	-0.92098100
C	1.59994300	-1.79767400	0.25627000
C	1.83982000	-0.83777300	1.28460000
C	-1.86826400	-0.70849000	1.30516300
C	-1.59518000	-1.76438200	0.38211100
H	2.87451100	0.78245300	-1.45857000
H	-2.85403700	0.61372000	-1.61107700
H	1.99040100	-1.73380800	-1.95763100
H	2.68122000	1.23559900	1.18344500
H	-2.73180800	1.33328700	0.97331000

H	-1.92074900	-1.91800400	-1.83741400
H	1.20001600	-2.80024100	0.40335500
H	1.64607300	-0.97129500	2.34944200
H	-1.70812300	-0.73599800	2.38337600
H	-1.19019900	-2.74266800	0.63745100
Cl	0.00843200	2.62053900	0.04655100

$Cp_2ZrClBr$

Zr	0.27014200	0.00761300	0.07088700
C	-0.20229300	2.47666500	-0.46441900
C	-0.12540000	-2.48978700	-0.37167400
C	0.40827700	1.91318700	-1.62034400
C	0.75198700	2.47992800	0.57831600
C	0.89894700	-2.43386200	0.60065300
C	0.38299100	-1.93653300	-1.58325100
C	1.75052600	1.59003100	-1.29048600
C	1.95835600	1.91323000	0.07815800
C	2.04469000	-1.84216400	0.00198900
C	1.73234100	-1.56179600	-1.35621000
H	-1.23127300	2.82342800	-0.39205600
H	-1.13195500	-2.87300400	-0.21601900
H	-0.06896300	1.76125500	-2.58937500
H	0.58147700	2.82516200	1.59623100
H	0.81510500	-2.75487900	1.63718000
H	-0.16388100	-1.83549400	-2.52092600
H	2.49194000	1.16865900	-1.96675600
H	2.88188200	1.76962700	0.63978600
H	2.99757700	-1.64556600	0.49535700
H	2.40936100	-1.13306000	-2.09275400
Br	-2.35537700	-0.03254600	-0.22601400
Cl	0.26168900	0.00441500	2.53223100

4

C	1.55094000	1.46387400	0.16591900
C	2.82971000	1.37371000	0.99802400
N	0.81844600	0.19929500	0.17938300
O	2.93317100	0.75161300	2.02768400
O	3.79611500	2.12656700	0.46598300
H	1.81337300	1.76245800	-0.85978400
Br	-2.22380100	2.47278800	1.70726300

C	1.38774500	-0.99555800	-0.17936100
C	5.03278400	2.18368000	1.19114400
C	0.55780900	2.45654400	0.80602200
C	-0.79392300	1.85987200	0.43767600
H	0.68811500	3.48090200	0.43461100
H	0.67901700	2.45273800	1.89855400
C	-0.60645900	0.32098500	0.50864100
N	-1.46971700	-0.19809500	-0.54798900
C	-1.66911100	0.81261100	-1.50942000
C	-1.26186000	2.05104500	-0.97757400
H	-0.85052800	-0.14370800	1.46853600
C	-2.17366900	0.72174600	-2.80985900
C	-1.34484500	3.21708600	-1.73399900
C	-1.84528300	3.13774400	-3.04017400
C	-2.25700800	1.90239900	-3.56105400
C	-2.11873500	-1.42510900	-0.53053500
H	-2.48767500	-0.23852100	-3.21410500
H	-2.64726800	1.85165200	-4.58161500
H	-1.03400000	4.17519800	-1.30902300
H	-1.91561100	4.03976000	-3.65348500
H	5.70337200	2.82440300	0.60389800
H	5.46787400	1.17806800	1.29912600
H	4.87747300	2.61360500	2.19329400
O	0.82760300	-2.07235200	-0.09812600
O	-2.70360200	-1.87466200	-1.49641300
O	-2.03892100	-1.98007900	0.67725800
O	2.63138500	-0.77918900	-0.64074300
C	-2.42937400	-3.37527700	0.93194000
C	3.58724800	-1.86666600	-0.89071700
C	4.85647700	-1.11183300	-1.28243100
C	3.08948200	-2.73772900	-2.04476100
C	3.80157500	-2.66419700	0.39668800
C	-3.94702100	-3.50864500	0.79911900
C	-1.98134500	-3.58510000	2.37710200
C	-1.67487400	-4.31418300	-0.01109000
H	-4.26826200	-3.32755600	-0.23538100
H	-4.45608400	-2.78830400	1.45965000
H	-4.25592300	-4.52441300	1.09334100
H	-2.01273800	-4.19518700	-1.04923600
H	-1.85134400	-5.35674800	0.29753700

H	-0.59734100	-4.10205300	0.03397700
H	-0.89323400	-3.44202900	2.46424100
H	-2.23178800	-4.60488100	2.70776500
H	-2.48217400	-2.86758800	3.04627400
H	5.66297000	-1.82347900	-1.51670900
H	5.19138900	-0.46146100	-0.45979200
H	4.67467800	-0.48328100	-2.16821600
H	2.16889100	-3.26957000	-1.77137200
H	3.86253700	-3.47717200	-2.30768600
H	2.88900700	-2.11761400	-2.93321000
H	2.89367700	-3.21247600	0.68192800
H	4.07802400	-1.98713000	1.22006200
H	4.61794000	-3.38877100	0.24993000

TS1

Zr	-3.65404500	-0.16221500	-0.44977100
C	-4.24190000	-2.40201600	0.60984700
C	-2.42170100	1.72148800	-1.61484800
C	-4.52453600	-2.51334000	-0.78388600
C	-5.16683000	-1.48918600	1.17485400
C	-3.53231200	2.35917200	-1.00300600
C	-2.91780800	0.86922500	-2.64426800
C	-5.60821100	-1.64667100	-1.07547500
C	-6.00935800	-1.01627600	0.13953600
C	-4.71261300	1.89124700	-1.62942400
C	-4.33357300	0.95674800	-2.63764100
H	-3.44764500	-2.91881100	1.14735300
H	-1.37803700	1.86409000	-1.33671200
H	-4.00876600	-3.15880200	-1.49371900
H	-5.19453700	-1.17474100	2.21663900
H	-3.48283400	3.05462800	-0.16698100
H	-2.31598000	0.26116900	-3.31812700
H	-6.07246200	-1.50688000	-2.05129000
H	-6.81389000	-0.28794100	0.24846200
H	-5.73330900	2.17445500	-1.36972200
H	-5.01117700	0.42860000	-3.30796300
H	7.12787400	1.35945100	-1.81001400
Cl	-2.87948600	0.73236700	1.73728300
C	2.84909100	-1.90993900	-0.07609200
C	3.78622800	-2.28812900	-1.21898000

N	2.62329300	-0.46700600	-0.02446000
O	3.81979900	-1.73519300	-2.29292900
O	4.51084700	-3.36603100	-0.90161000
H	3.26720600	-2.28625500	0.86942300
Br	-1.34757600	-1.48138300	-0.89902000
C	3.63800700	0.44967200	-0.01270500
C	5.36841500	-3.88419500	-1.92759000
C	1.43284800	-2.49102000	-0.33256600
C	0.53208900	-1.43652300	0.26622300
H	1.30392500	-3.48128200	0.12313300
H	1.26568900	-2.57688500	-1.41792900
C	1.20369900	-0.07982900	0.02129700
N	0.85448400	0.71540600	1.19961900
C	0.44368800	-0.15107300	2.23504200
C	0.24502100	-1.44612500	1.70208600
H	0.90837600	0.44974300	-0.89106900
C	0.22372300	0.11704600	3.58487000
C	-0.19713700	-2.48665000	2.52609500
C	-0.43193100	-2.22415800	3.88130100
C	-0.22245100	-0.93677800	4.39838600
C	0.66800700	2.08983100	1.21505700
H	0.38151100	1.11818400	3.98092300
H	-0.40771200	-0.74461300	5.45913400
H	-0.36199100	-3.48458000	2.11059700
H	-0.77771300	-3.02553200	4.54002900
H	5.86941300	-4.76024500	-1.49531300
H	6.11478400	-3.13298400	-2.22936400
H	4.78378300	-4.17959900	-2.81312400
O	3.47702800	1.65225100	-0.11356900
O	0.52158900	2.72819700	2.23824400
O	0.63936300	2.58156500	-0.02796800
O	4.81769100	-0.18022600	0.14813900
C	0.76499600	4.02120900	-0.30568500
C	6.10505000	0.47152600	-0.12119100
C	7.10208500	-0.67409400	0.04804600
C	6.35829700	1.57303600	0.90890100
C	6.11865600	0.99756400	-1.55789900
C	-0.52070800	4.73191400	0.11846600
C	0.94228800	4.05157100	-1.82282500
C	2.00164000	4.59247100	0.39142500

H	-0.67531700	4.64272100	1.20220600
H	-1.39107700	4.30271000	-0.40086800
H	-0.45618800	5.79994100	-0.14435200
H	1.87061300	4.62254000	1.48115200
H	2.17650000	5.61877600	0.03155000
H	2.87943500	3.97417600	0.15778300
H	1.84483900	3.49052000	-2.10959600
H	1.04302000	5.09069600	-2.17243400
H	0.07368800	3.59894000	-2.32642800
H	8.12844600	-0.31169500	-0.11670800
H	6.89441100	-1.47933500	-0.67331000
H	7.03769300	-1.09546600	1.06353200
H	5.63633000	2.39227100	0.79735400
H	7.37588500	1.97435600	0.77742400
H	6.27674900	1.16765700	1.93023100
H	5.40813700	1.82569300	-1.68482600
H	5.84701100	0.19151400	-2.25701000

Int1

C	-1.56604300	1.61018100	-0.43616300
C	-2.82760000	1.14818000	-1.15873400
N	-0.65273600	0.48604300	-0.20616600
O	-2.86386100	0.26248300	-1.98003800
O	-3.87813400	1.89744900	-0.80879300
H	-1.85520700	2.08223700	0.51354000
H	-3.97304800	-3.38739000	0.82194100
C	-1.03421200	-0.64154900	0.46648100
C	-5.11900200	1.60540700	-1.46590900
C	-0.74451000	2.60967500	-1.31863900
C	0.64949700	2.16702900	-1.03922900
H	-0.94424000	3.65340700	-1.04271500
H	-1.00676500	2.47043600	-2.38122800
C	0.66666700	0.66860400	-0.84765600
N	1.83128500	0.43503100	0.00882300
C	2.34834400	1.66949400	0.45238500
C	1.63296900	2.73060600	-0.19240500
H	0.74021800	0.05019900	-1.75343900
C	3.36584400	1.93590300	1.36431600
C	1.94892600	4.07316000	0.11289200
C	2.97410900	4.33676200	1.02054500

C	3.67582000	3.28156200	1.63252400
C	2.54048700	-0.76390800	0.06873000
H	3.89360600	1.12164100	1.85683900
H	4.47525000	3.50745600	2.34376700
H	1.39820700	4.88583600	-0.36790800
H	3.23446000	5.37086400	1.26247900
H	-5.85738100	2.30487600	-1.05253500
H	-5.42984200	0.56748800	-1.26868600
H	-5.02859600	1.74841300	-2.55431600
O	-0.35176300	-1.64172900	0.58986000
O	3.43239500	-0.96055000	0.87020400
O	2.13771700	-1.59934100	-0.88411400
O	-2.26378600	-0.45626200	0.98824200
C	2.54948300	-3.01095700	-0.92646800
C	-3.06707700	-1.55526000	1.53496700
C	-4.39569200	-0.87018400	1.85296300
C	-2.41249700	-2.09849700	2.80587300
C	-3.25380100	-2.63332800	0.46538900
C	4.03198900	-3.08840700	-1.29359600
C	1.67099100	-3.57667200	-2.04053000
C	2.24240200	-3.69864900	0.40522700
H	4.65470700	-2.64742400	-0.50333900
H	4.22422300	-2.55241400	-2.23706200
H	4.32579300	-4.14151700	-1.42922800
H	2.89132000	-3.32562500	1.20861300
H	2.40692200	-4.78248600	0.29714500
H	1.19520100	-3.51886800	0.68486500
H	0.60787700	-3.46513000	-1.77799500
H	1.89010600	-4.64483900	-2.19234300
H	1.85424000	-3.04444400	-2.98749600
H	-5.10362600	-1.59462200	2.28414300
H	-4.84034800	-0.44487500	0.93971700
H	-4.24588200	-0.05309000	2.57607900
H	-1.45019300	-2.57654400	2.58110600
H	-3.07760300	-2.84042200	3.27592200
H	-2.24178600	-1.28293800	3.52692300
H	-2.30446800	-3.13580200	0.23596800
H	-3.64607700	-2.18325400	-0.46001600

Compound S3 ^{1}H NMR (600 MHz, CDCl$_3$)

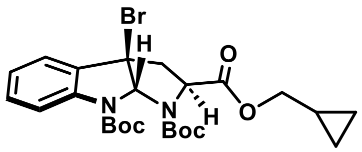

S3

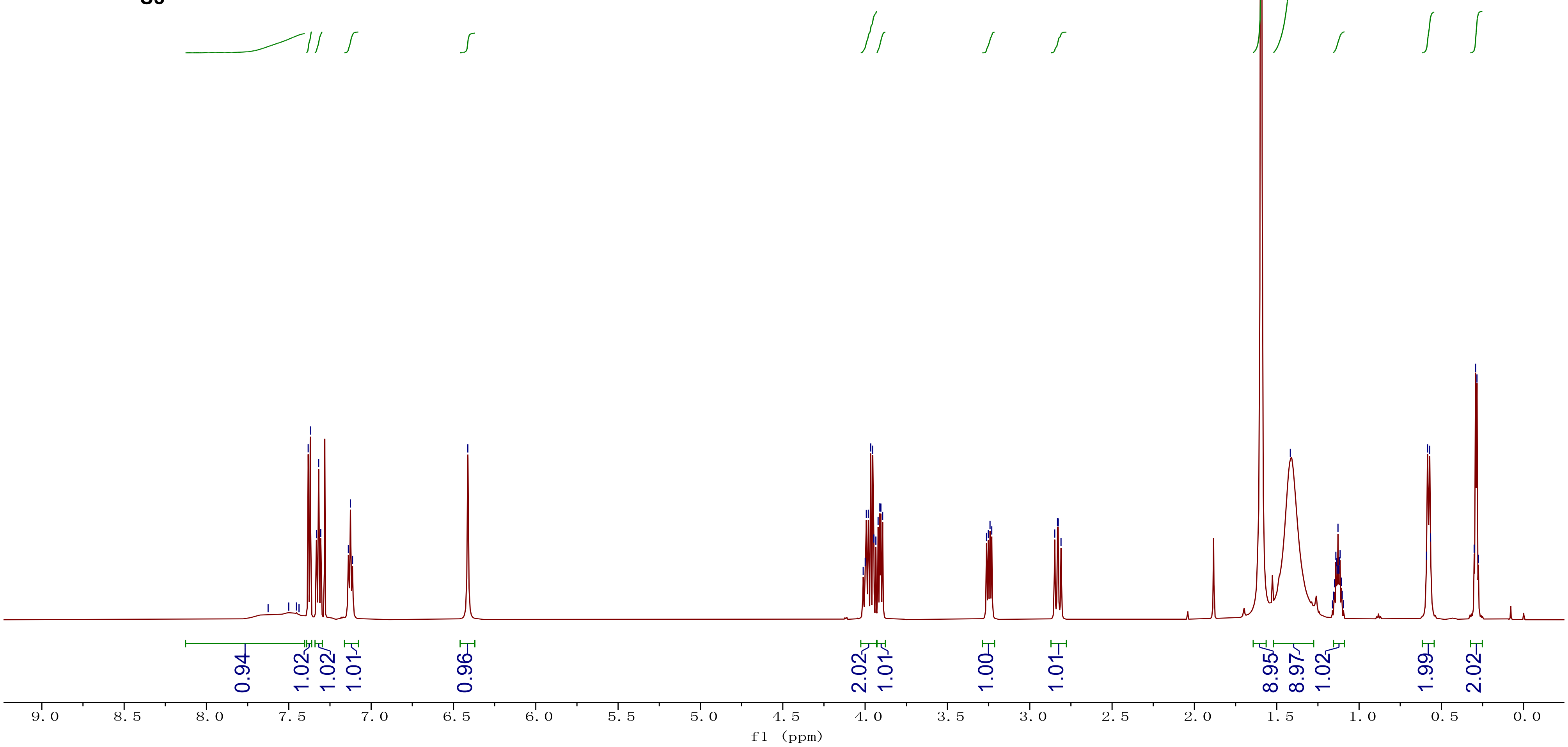

Compound S3 ^{13}C NMR (151 MHz, CDCl$_3$)

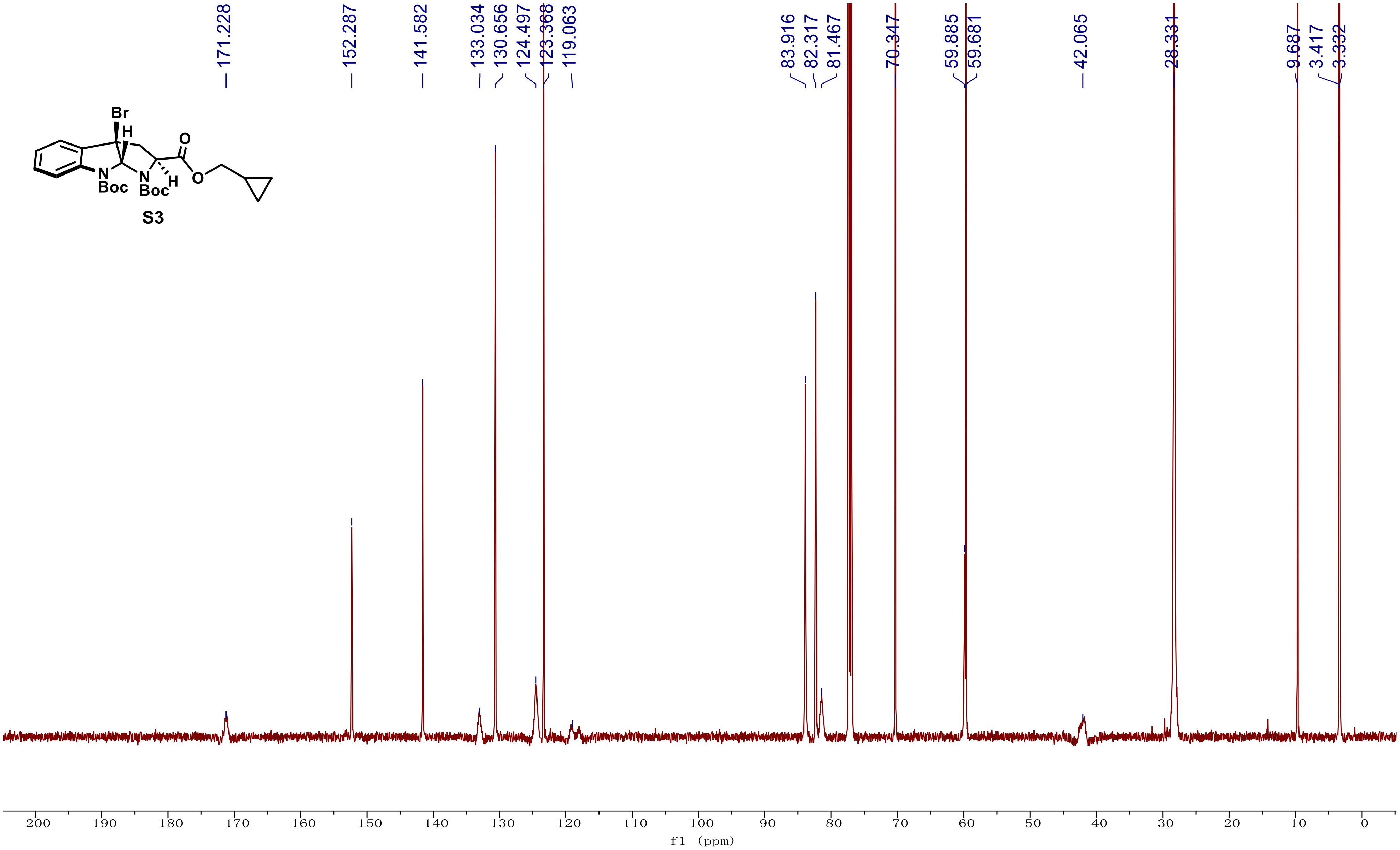

Compound S4 ^{1}H NMR (600 MHz, $CDCl_3$)

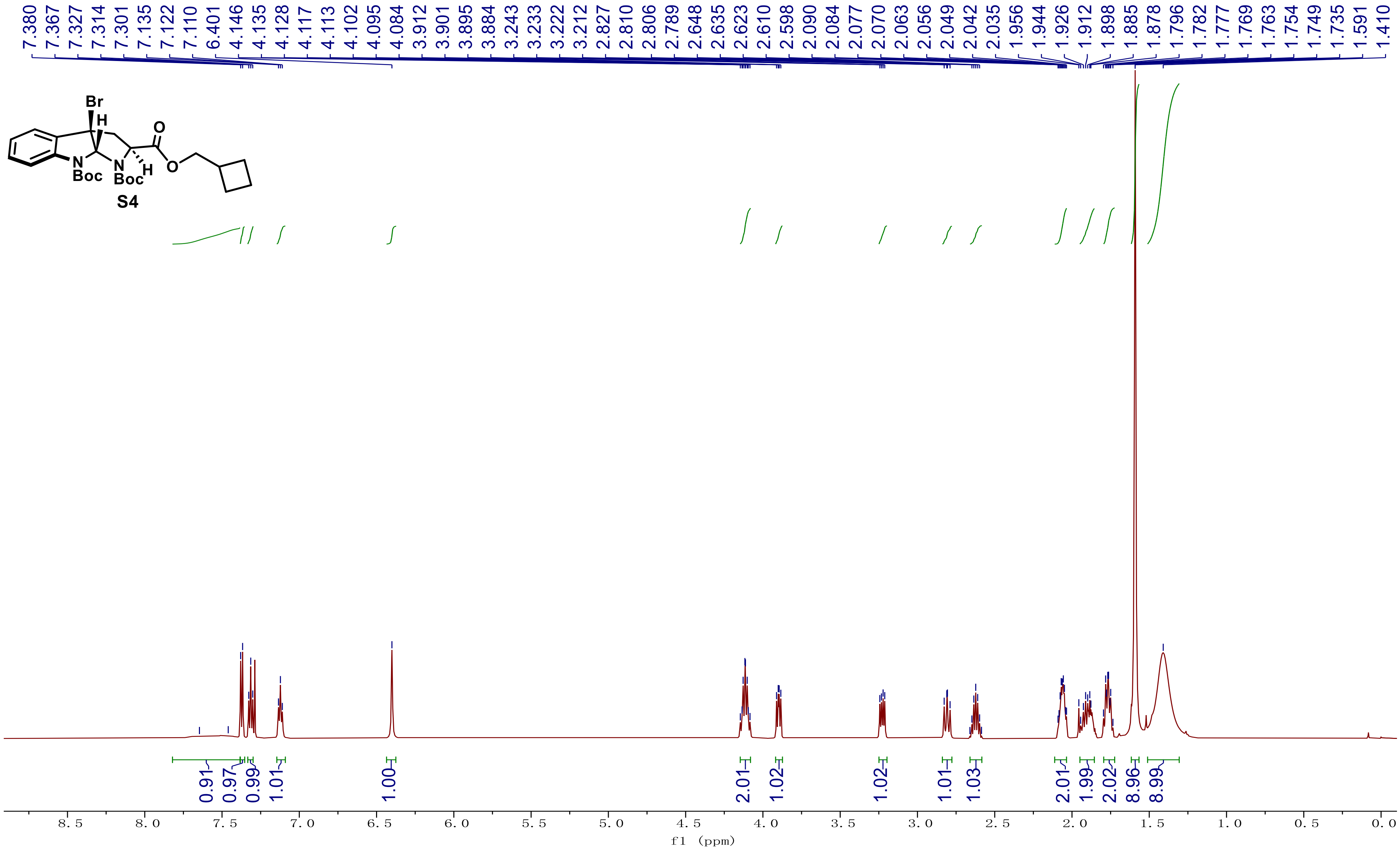

Compound S4 ^{13}C NMR (151 MHz, CDCl$_3$)

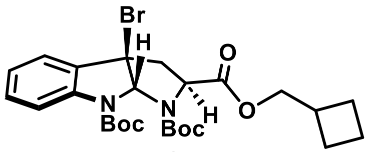

S4

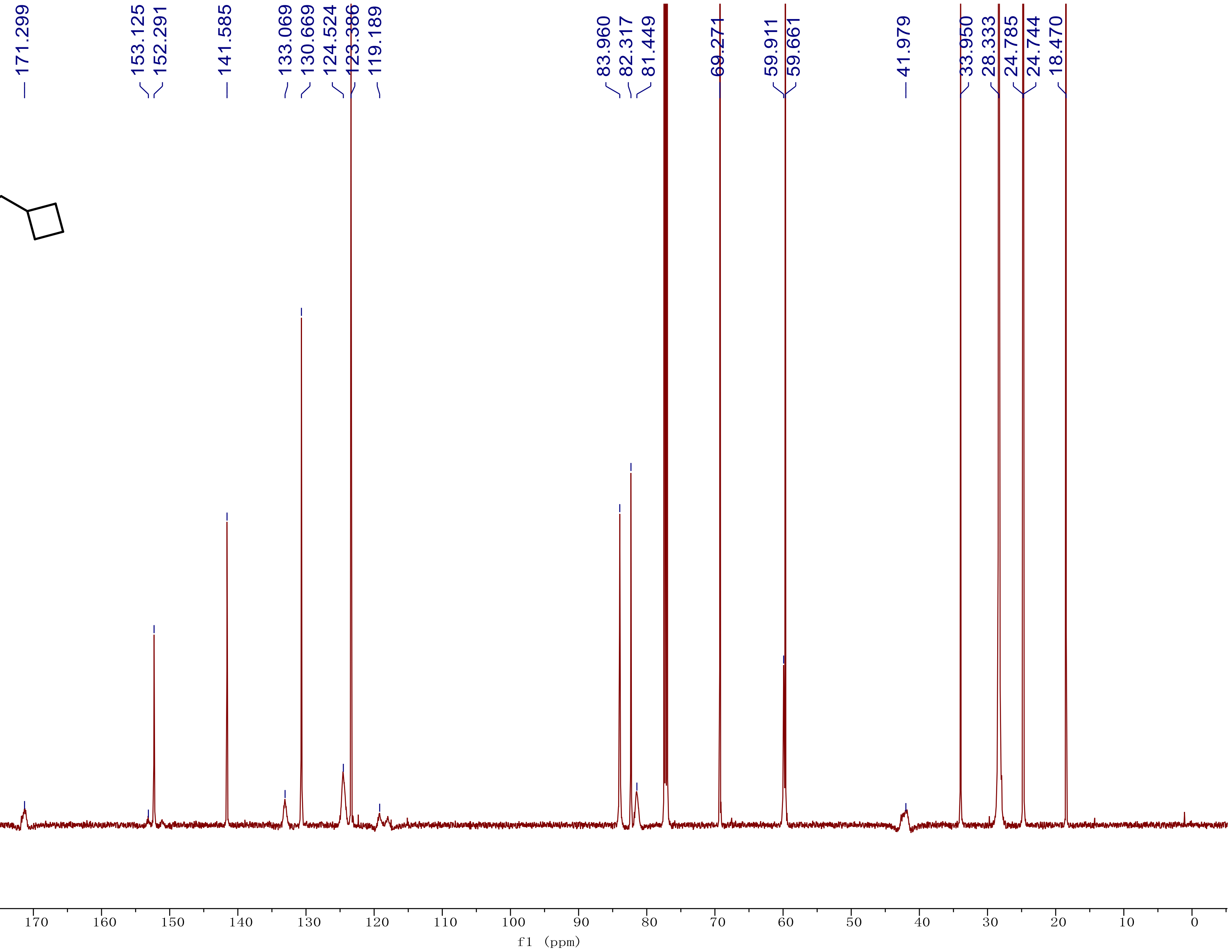

Compound S5 ¹H NMR (600 MHz, $CDCl_3$)

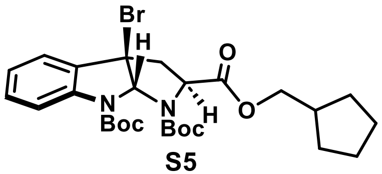

S5

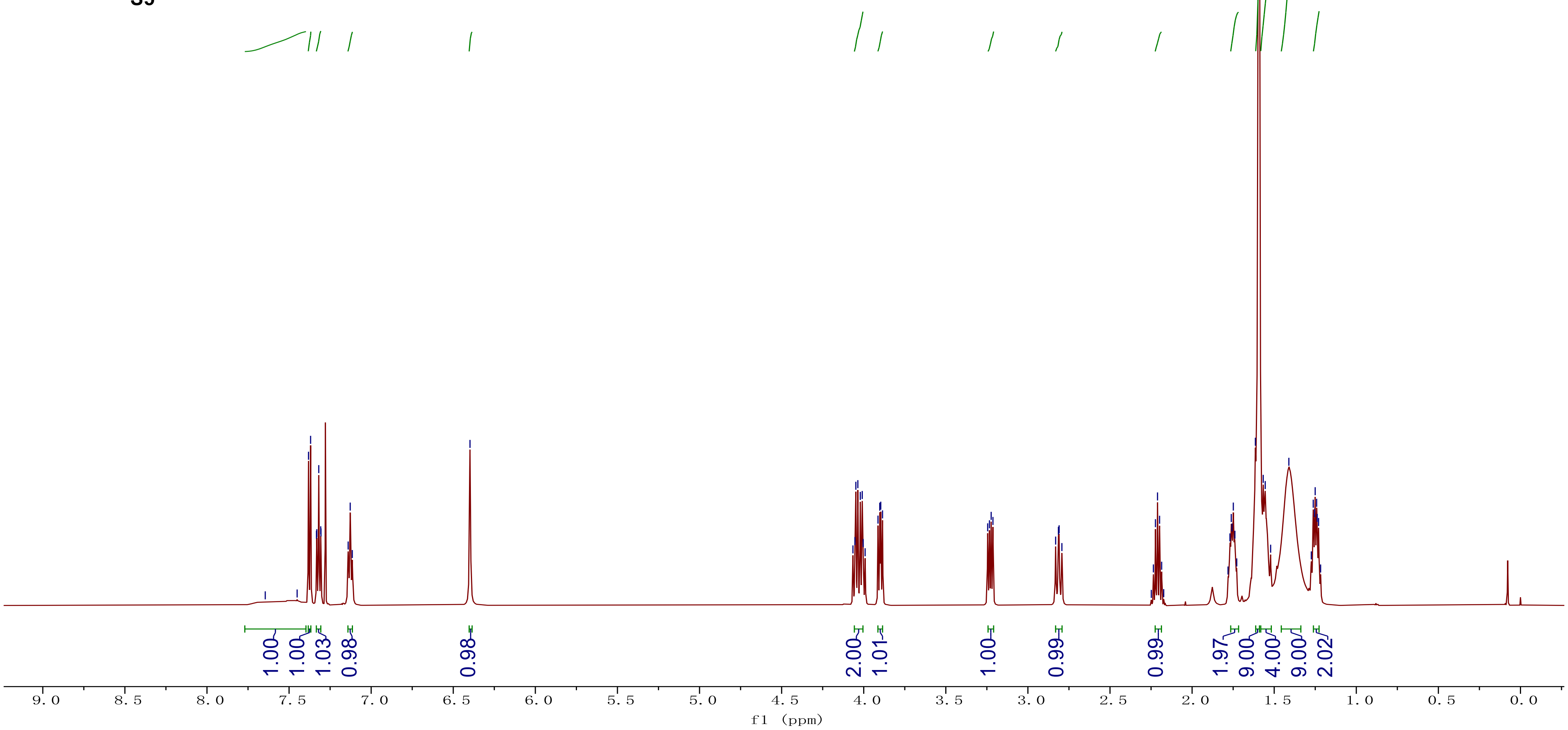

Compound S5 ^{13}C NMR (151 MHz, $CDCl_3$)

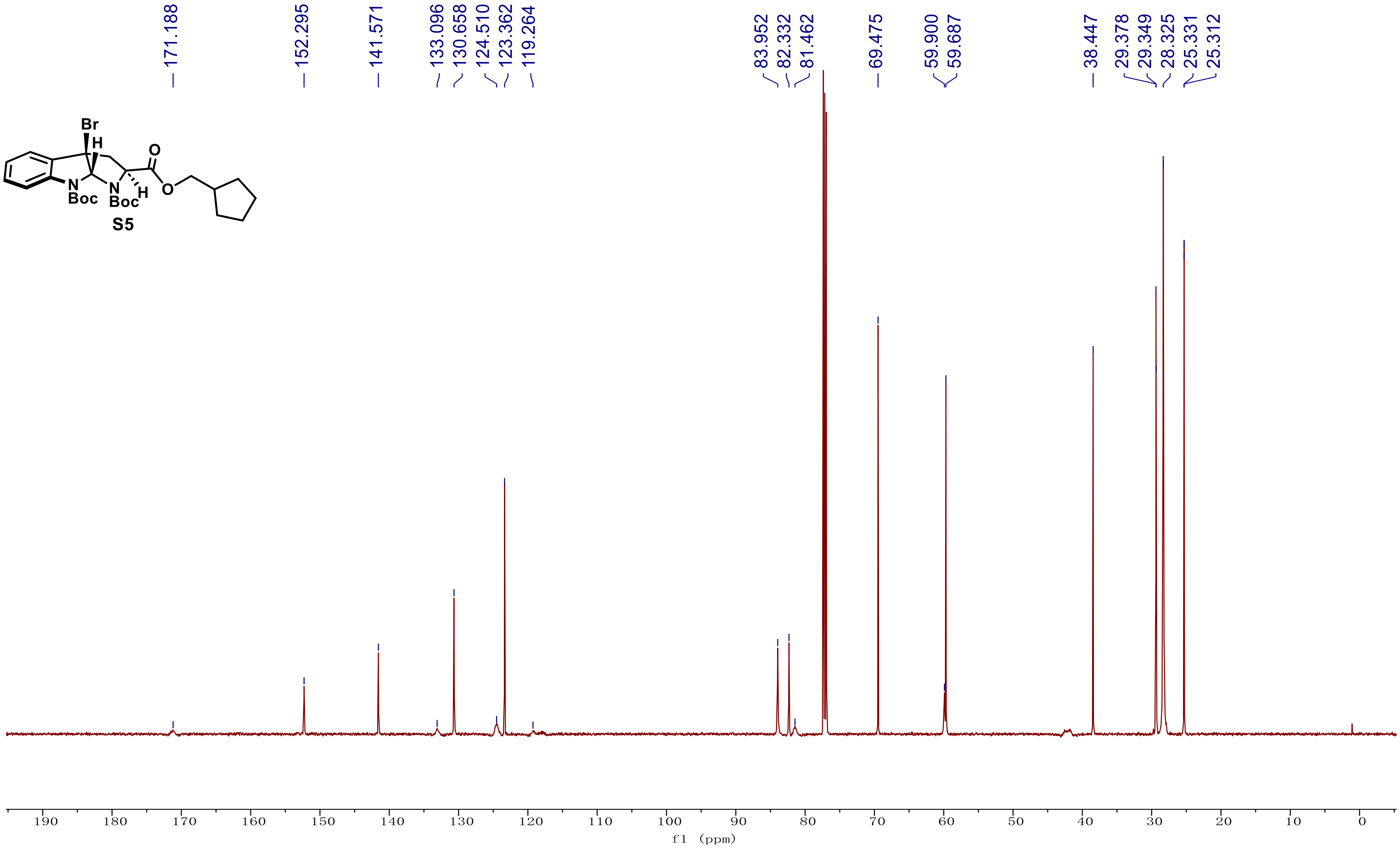

Compound S6 ^{1}H NMR (600 MHz, $CDCl_3$)

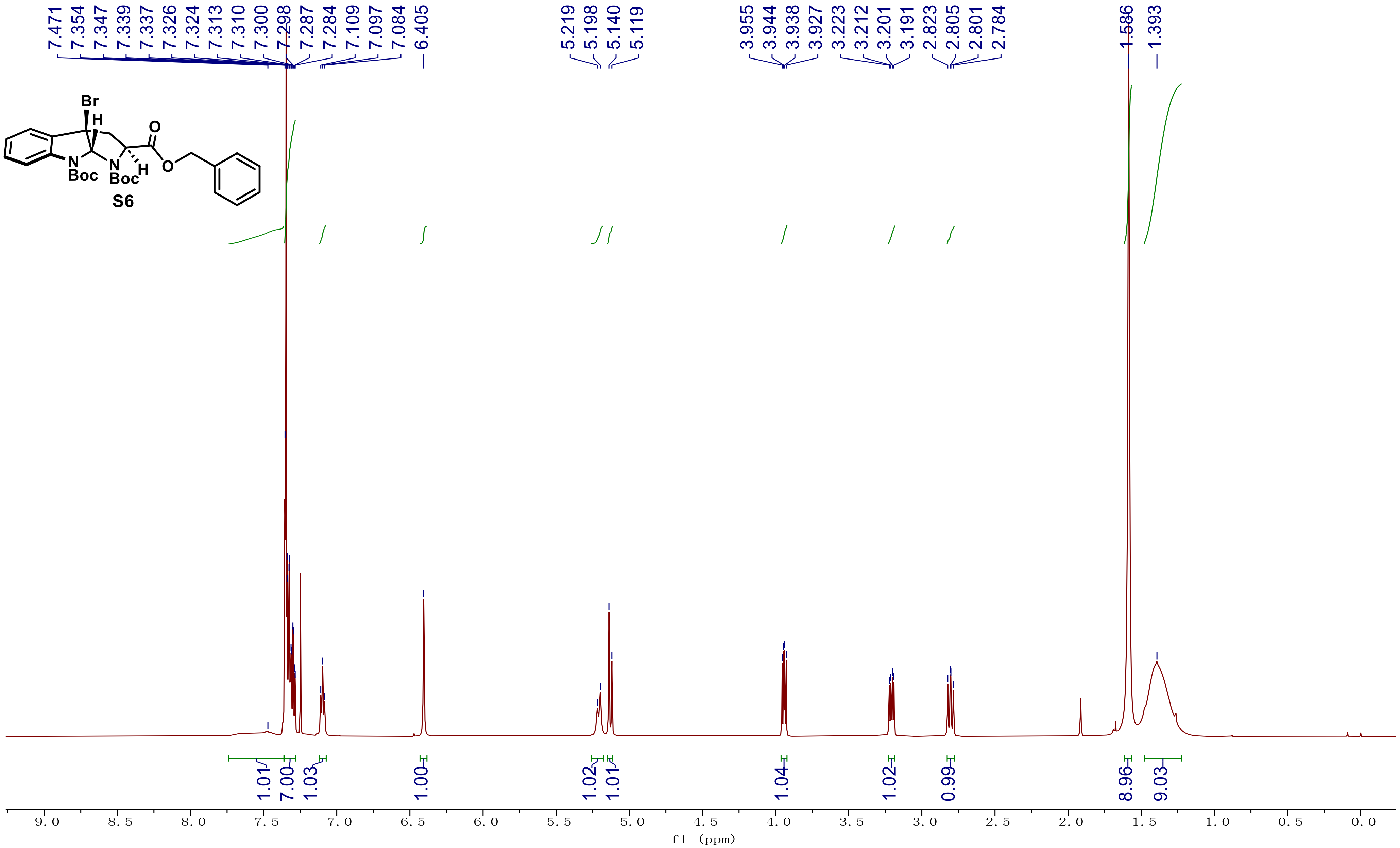

Compound S6 ^{13}C NMR (151 MHz, $CDCl_3$)

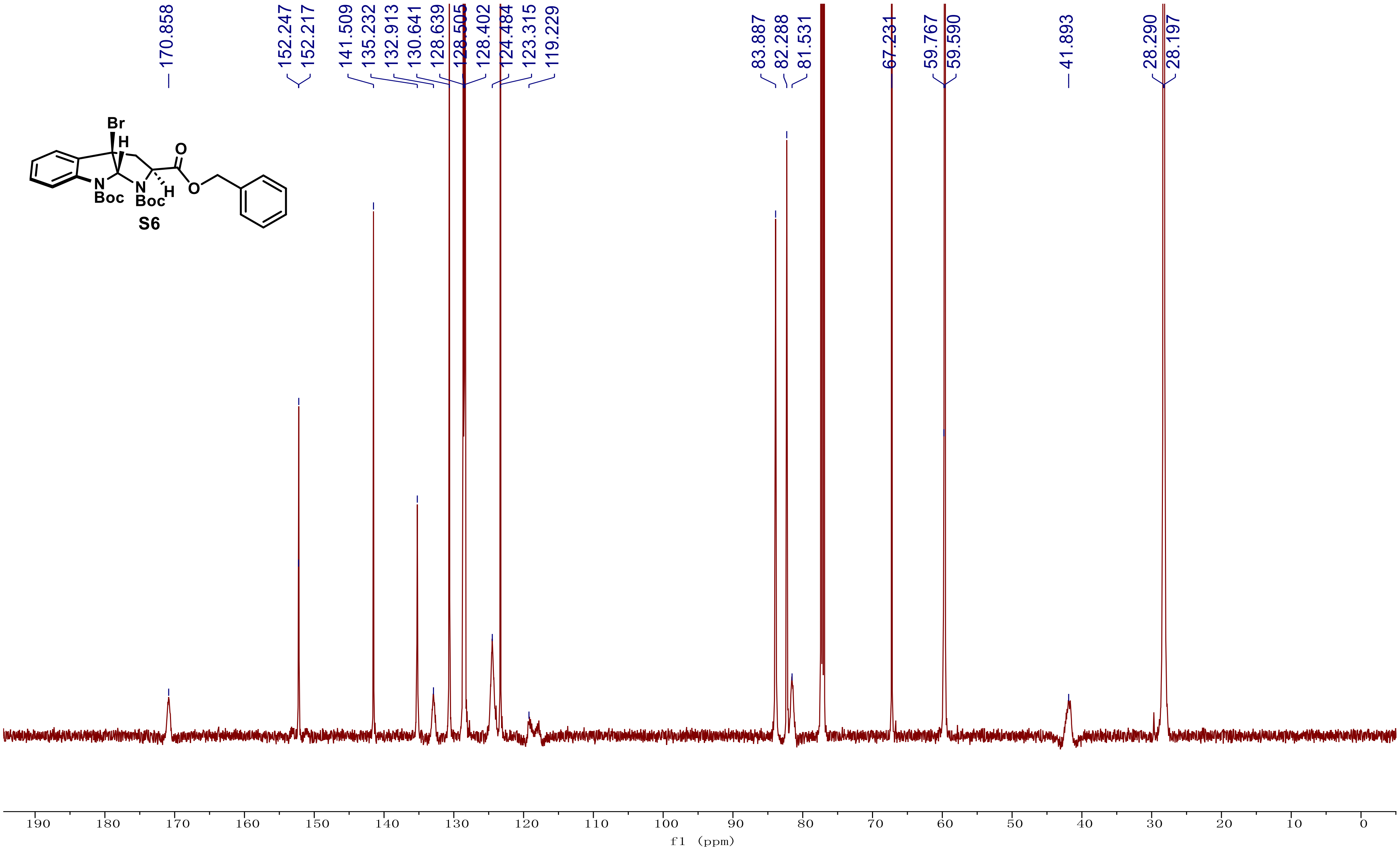

S6

Compound S7 ^{1}H NMR (600 MHz, $CDCl_3$)

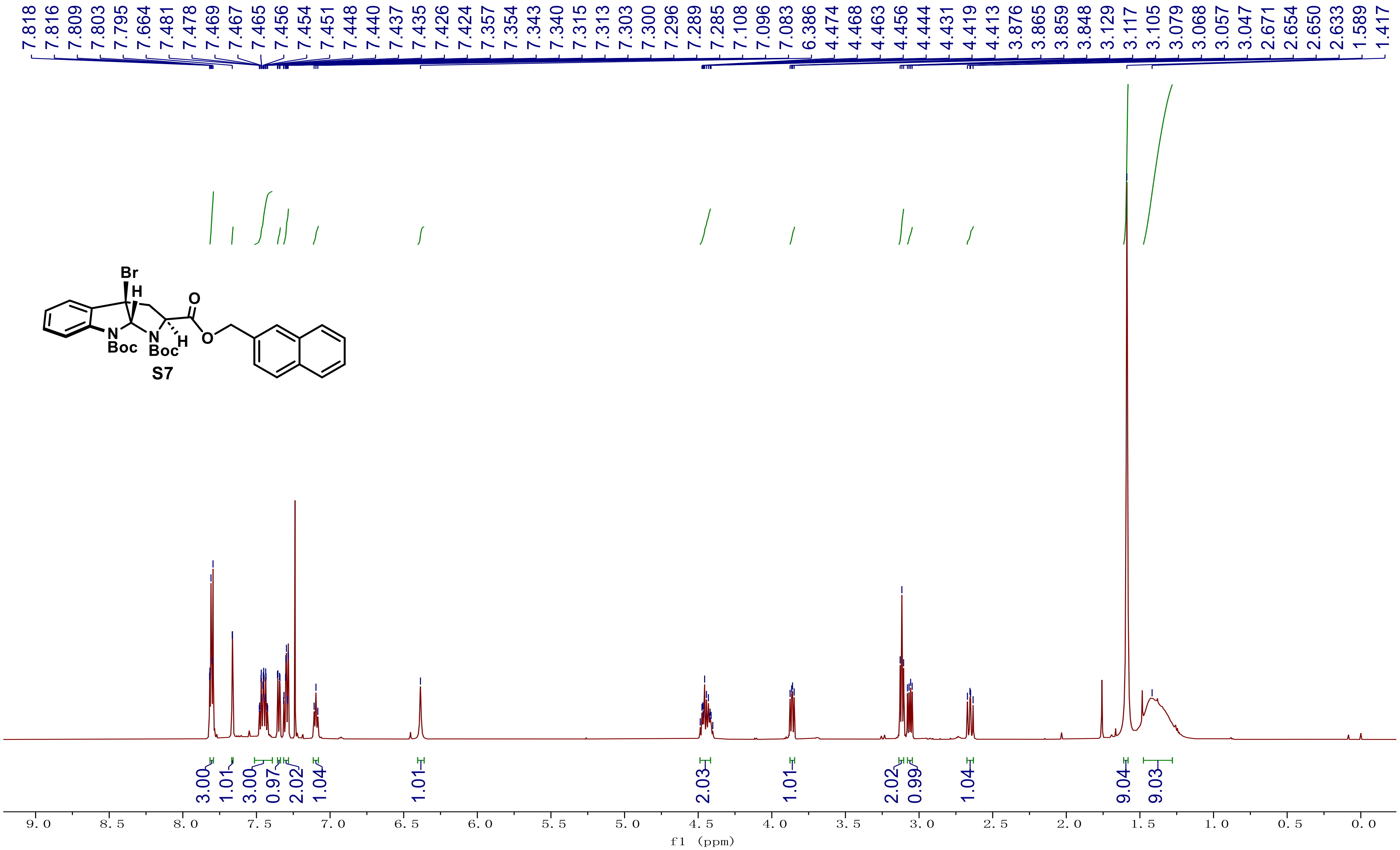

Compound S7 ^{13}C NMR (151 MHz, $CDCl_3$)

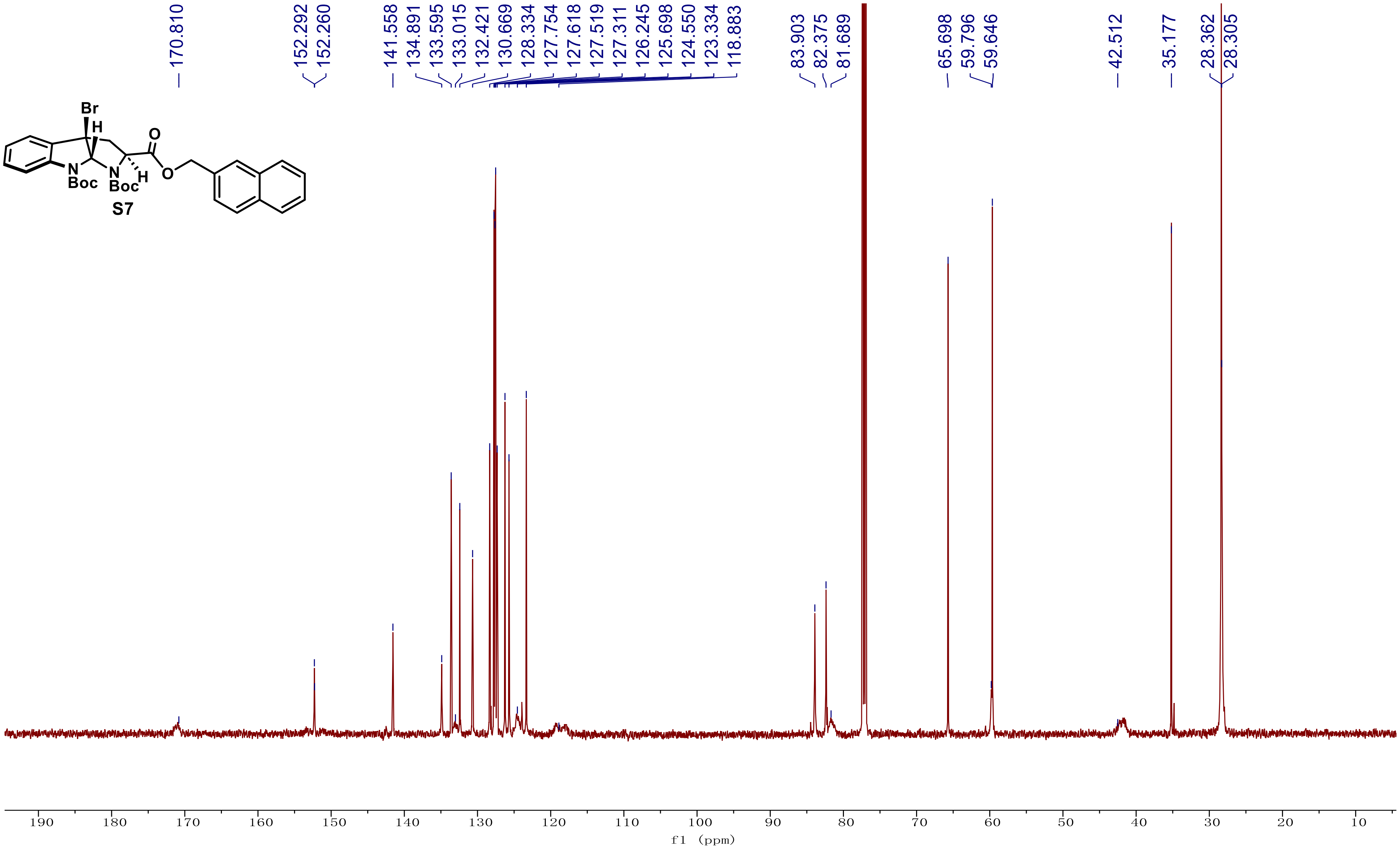

Compound S8 ^{1}H NMR (600 MHz, $CDCl_3$)

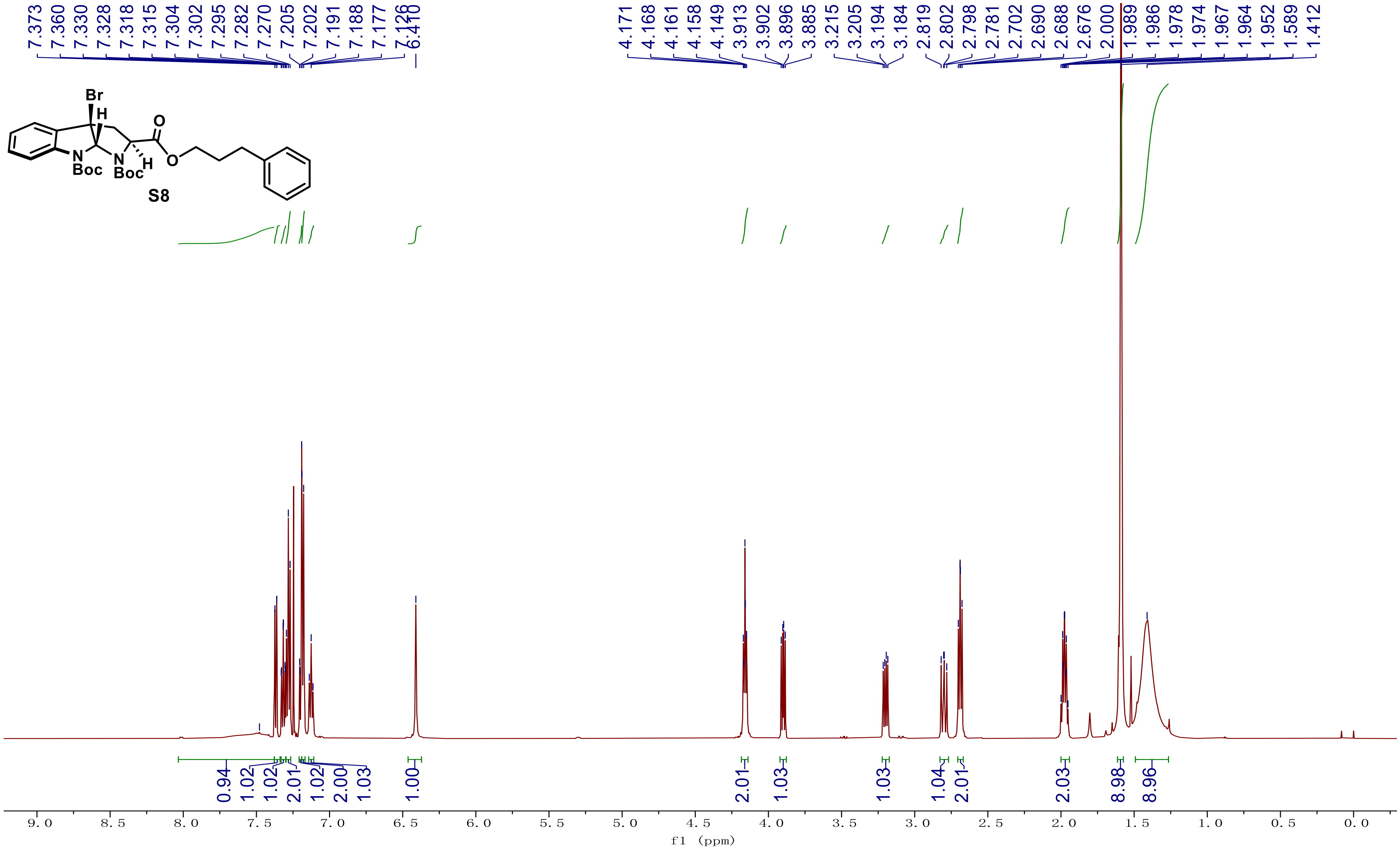

Compound S8 ^{13}C NMR (151 MHz, $CDCl_3$)

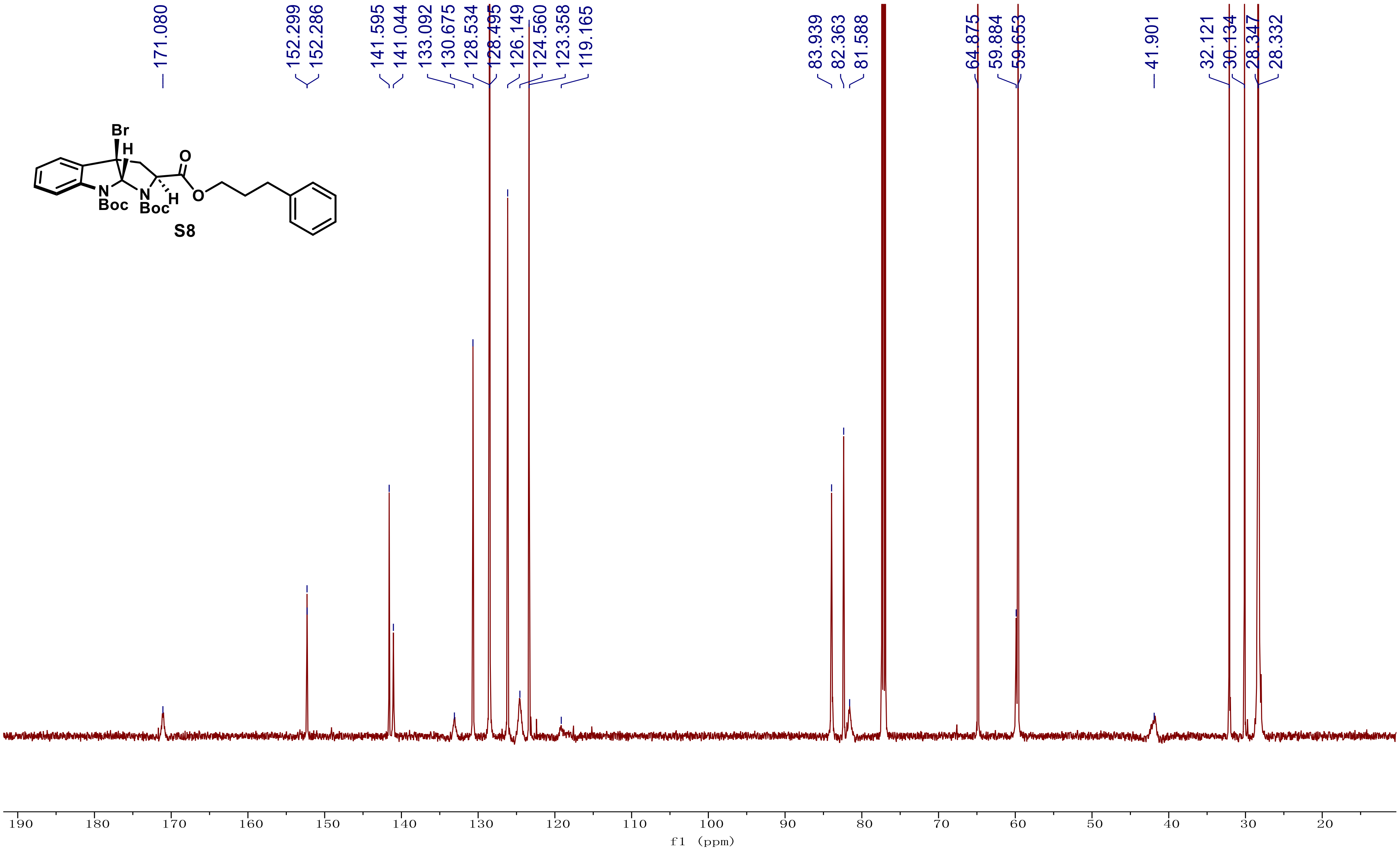

Compound S9 ^{1}H NMR (400 MHz, $CDCl_3$)

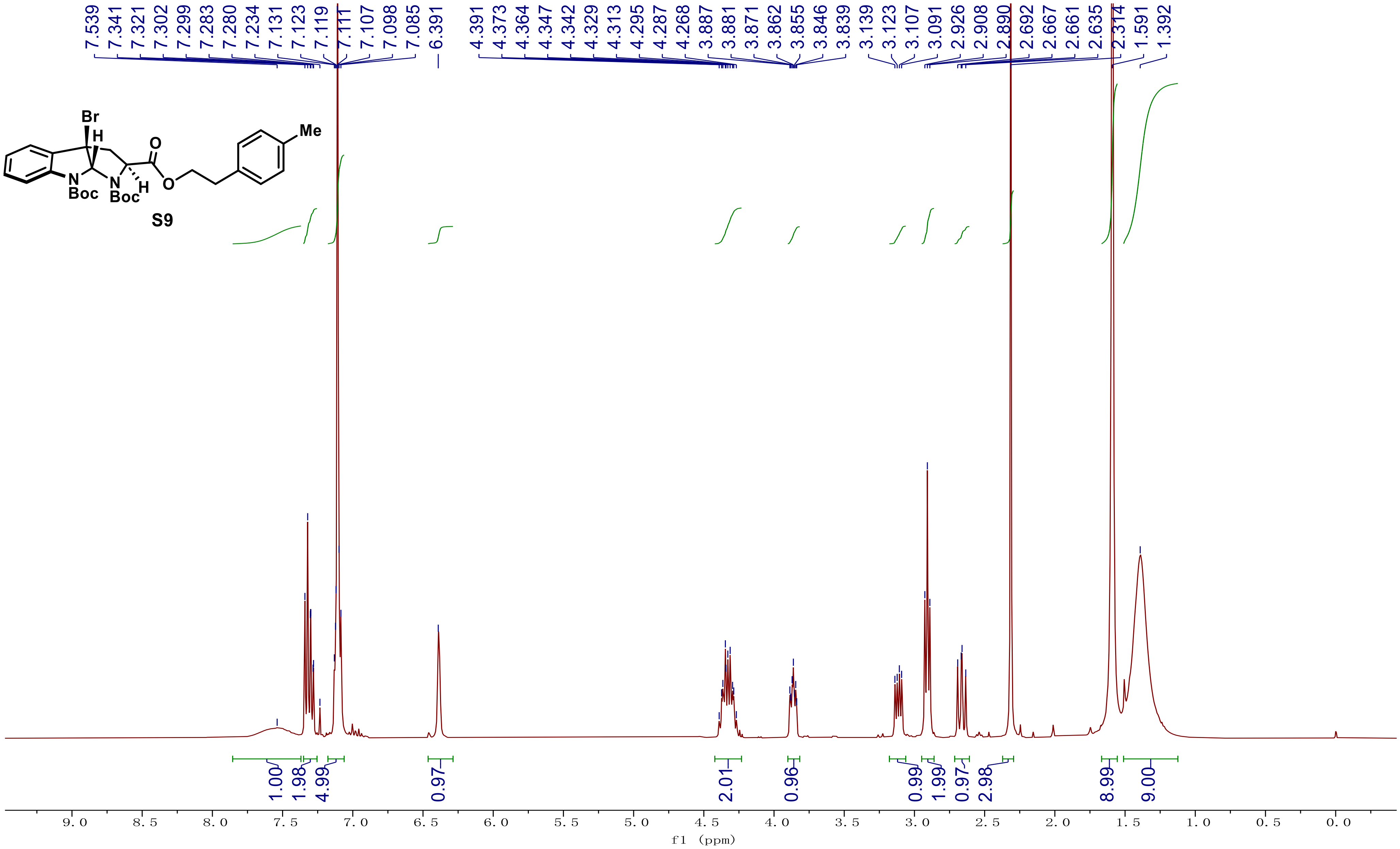

Compound S9 ^{13}C NMR (101 MHz, $CDCl_3$)

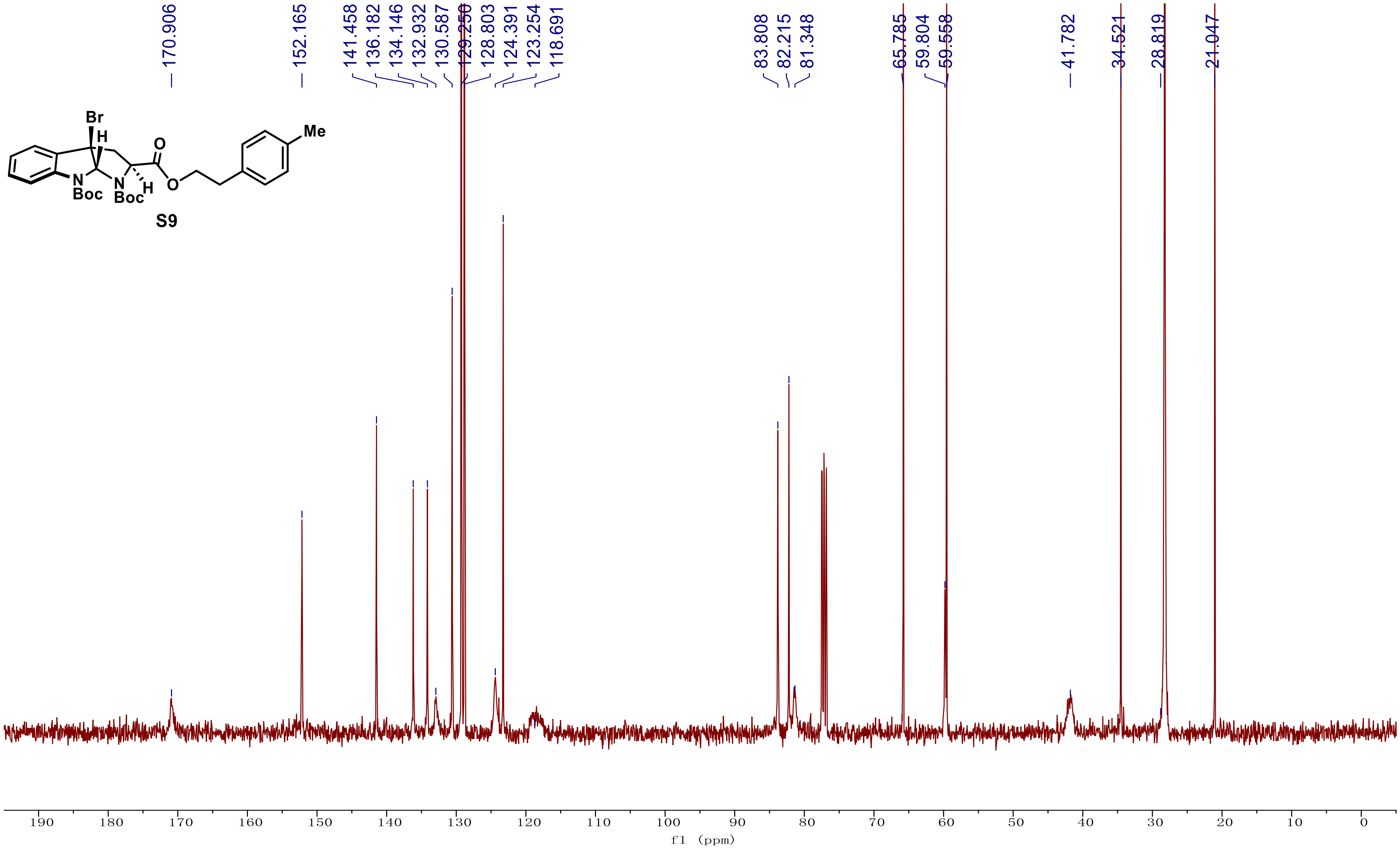

Compound S10 ^{1}H NMR (600 MHz, $CDCl_3$)

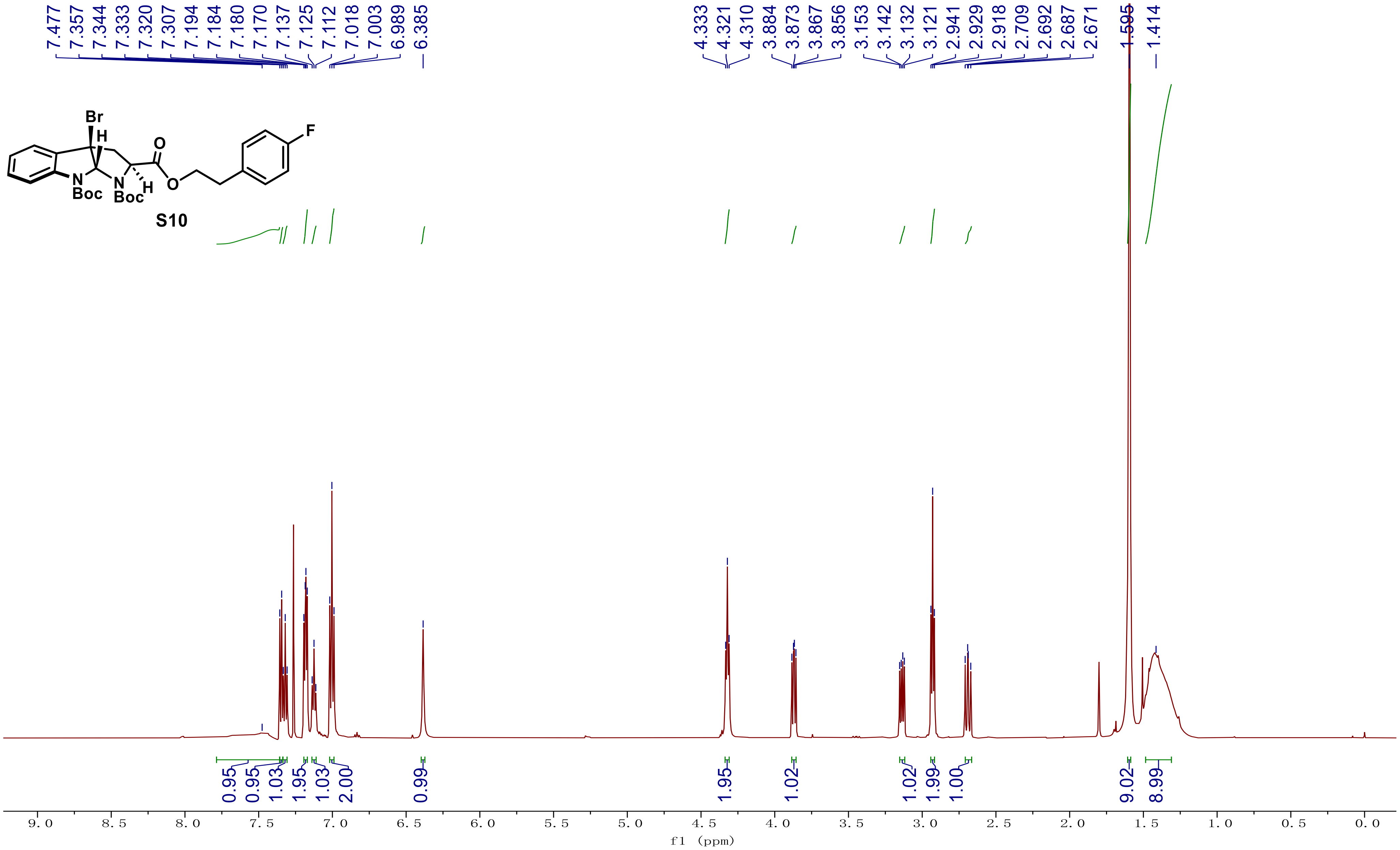

Compound A12 ^{13}C NMR (151 MHz, $CDCl_3$)

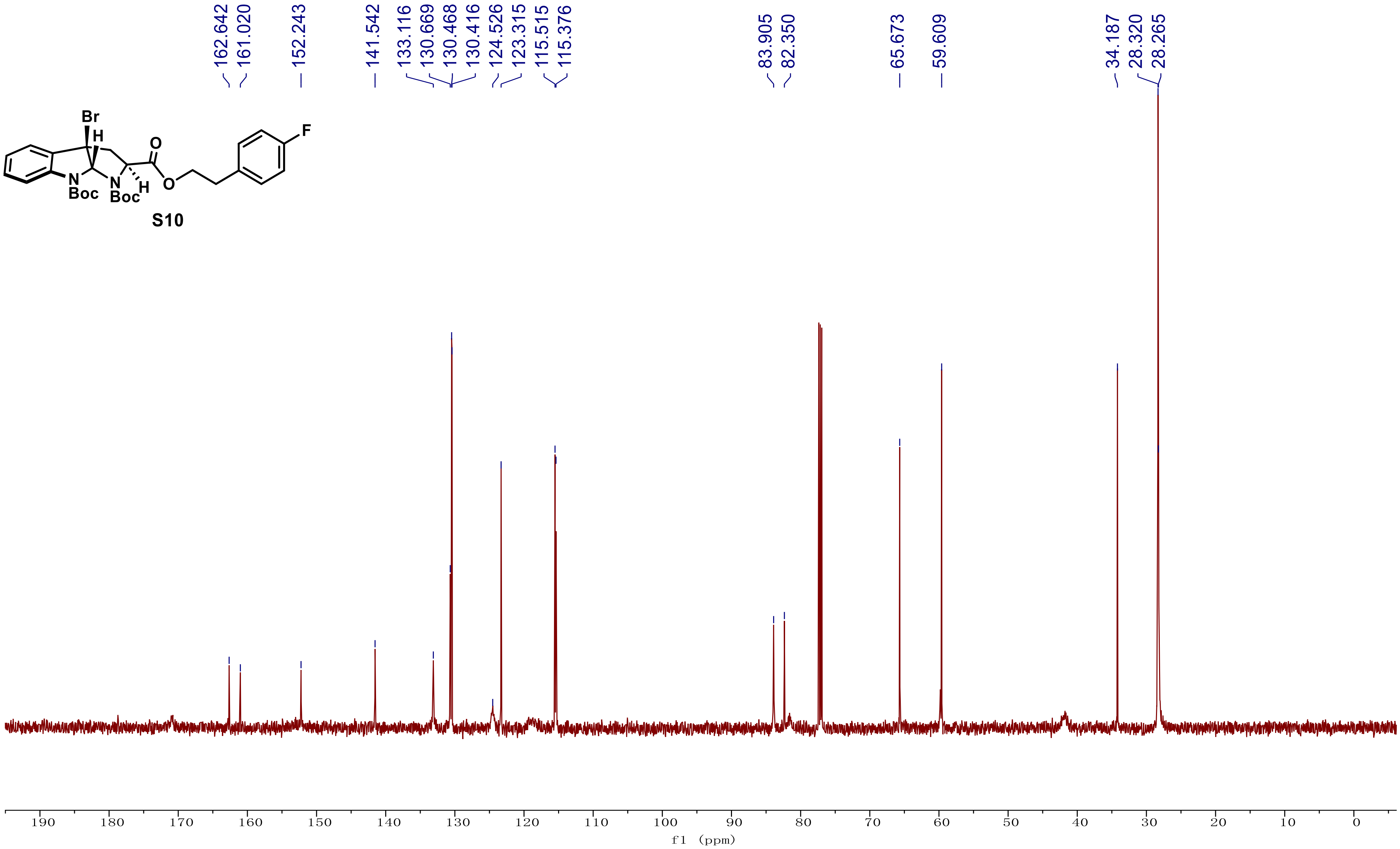

Compound S10 ^{19}F NMR (565 MHz, $CDCl_3$)

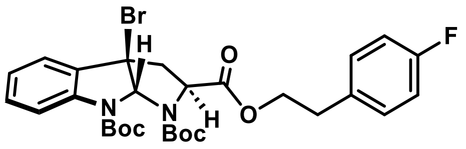

S10

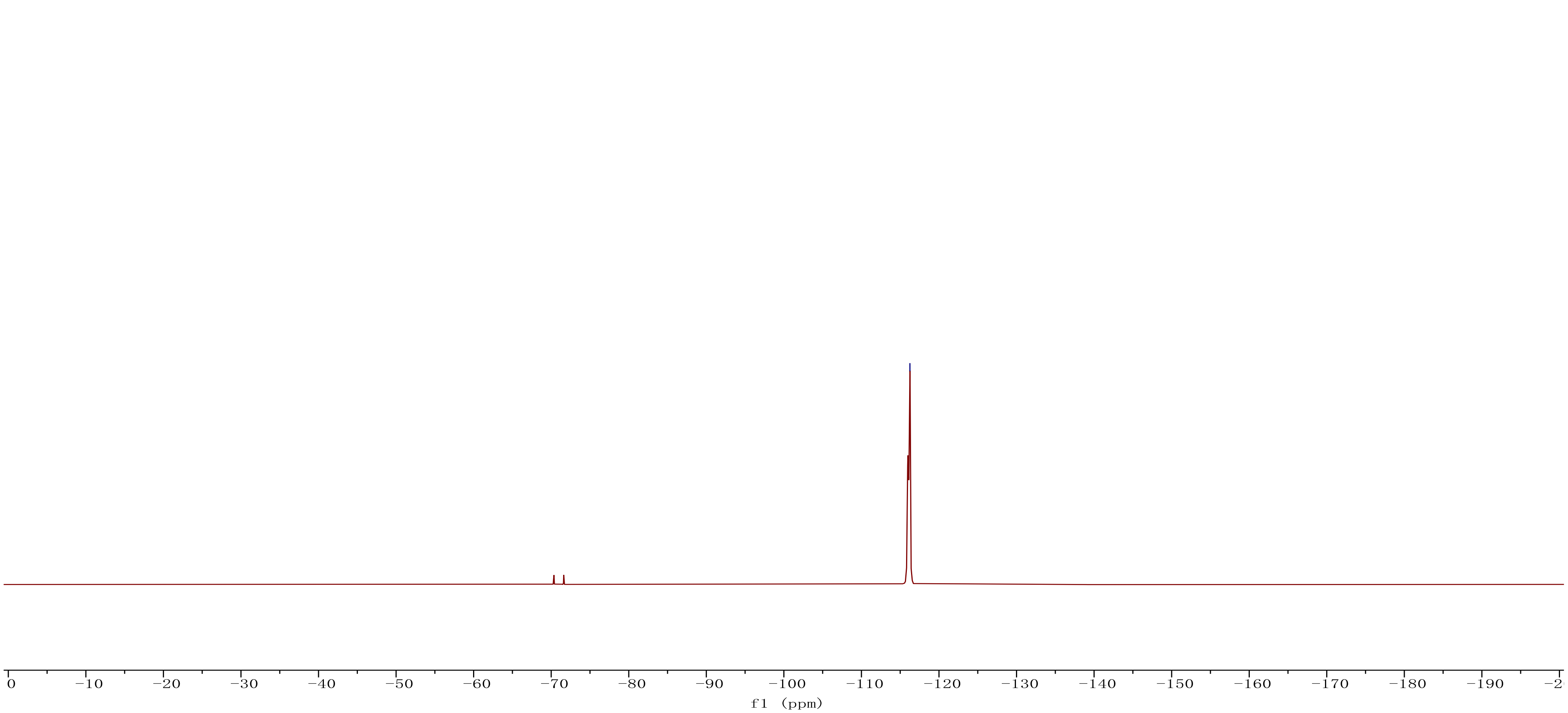

Compound S11 ^{1}H NMR (600 MHz, $CDCl_3$)

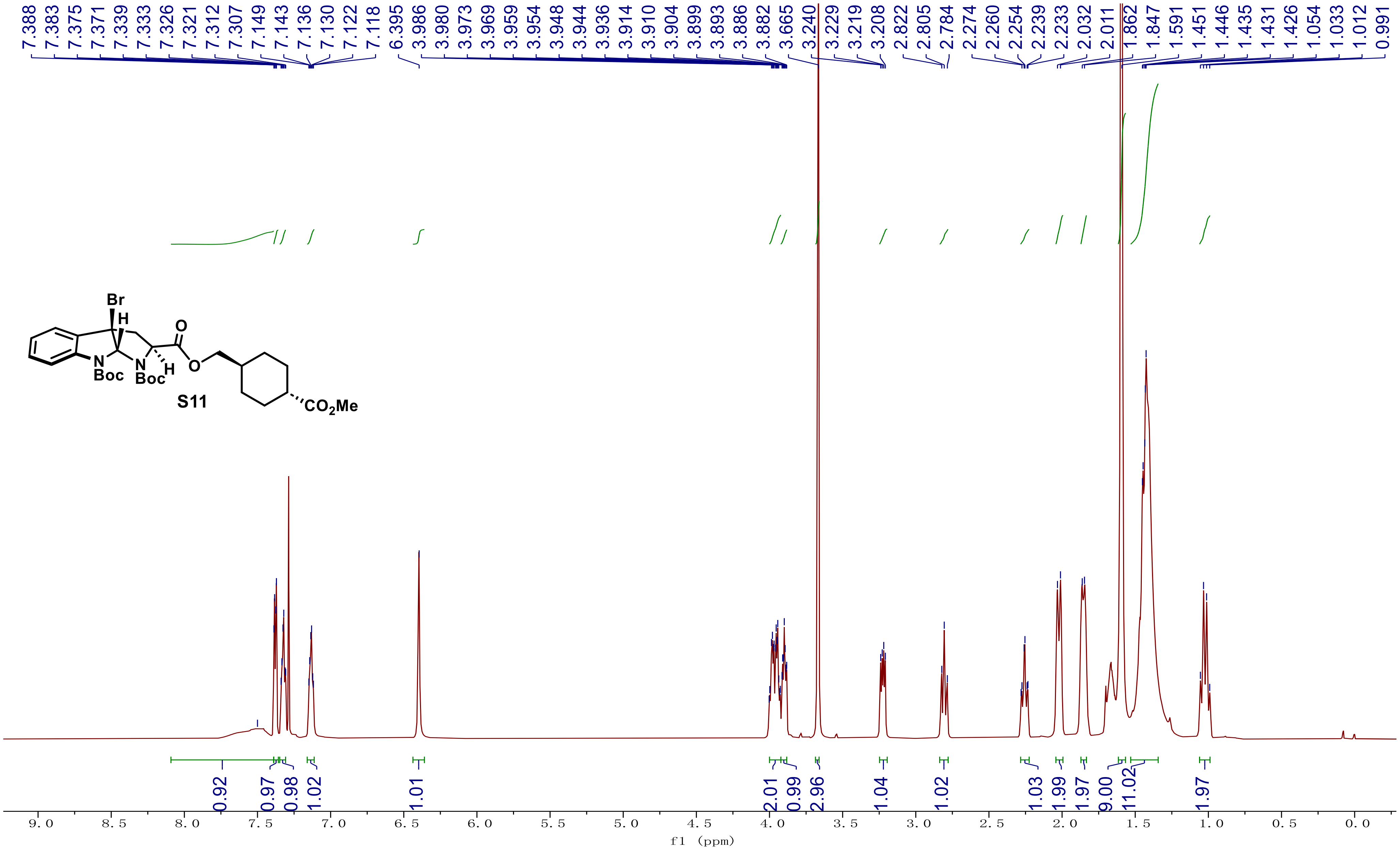

Compound S11 ^{13}C NMR (151 MHz, $CDCl_3$)

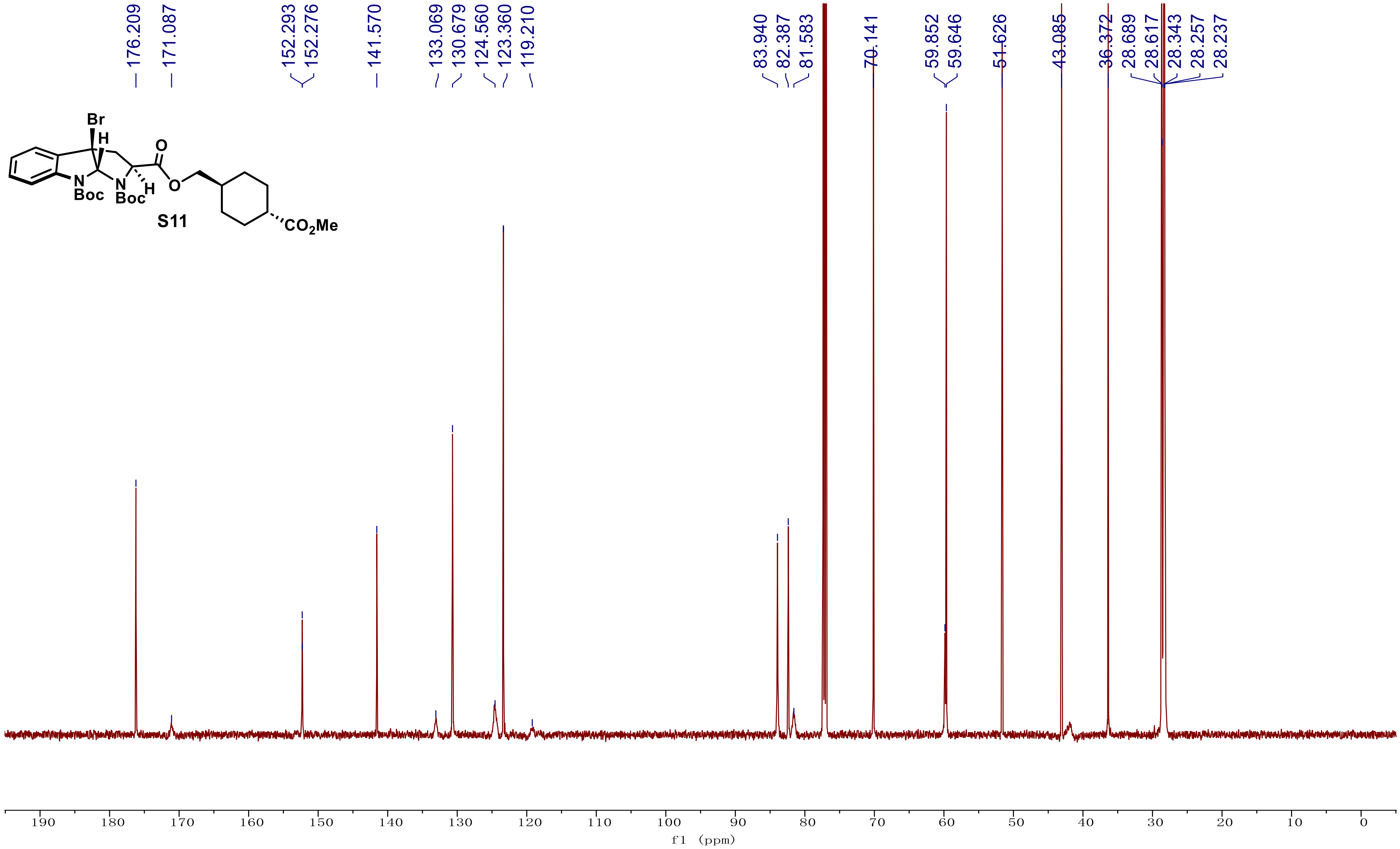

Compound S12 ^{1}H NMR (600 MHz, $CDCl_3$)

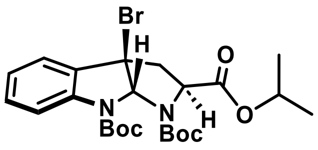

S12

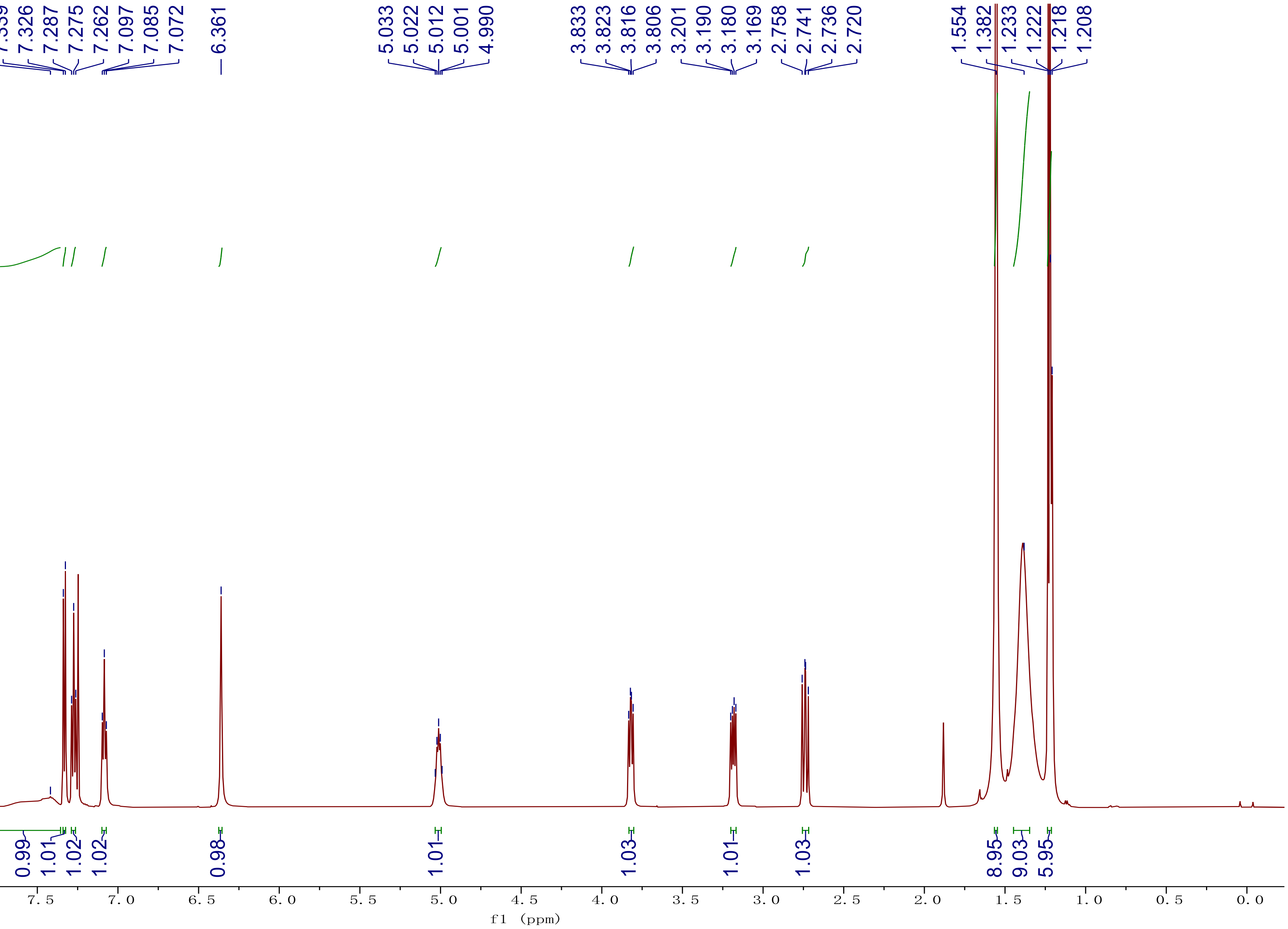

Compound S12 ^{13}C NMR (151 MHz, CDCl$_3$)

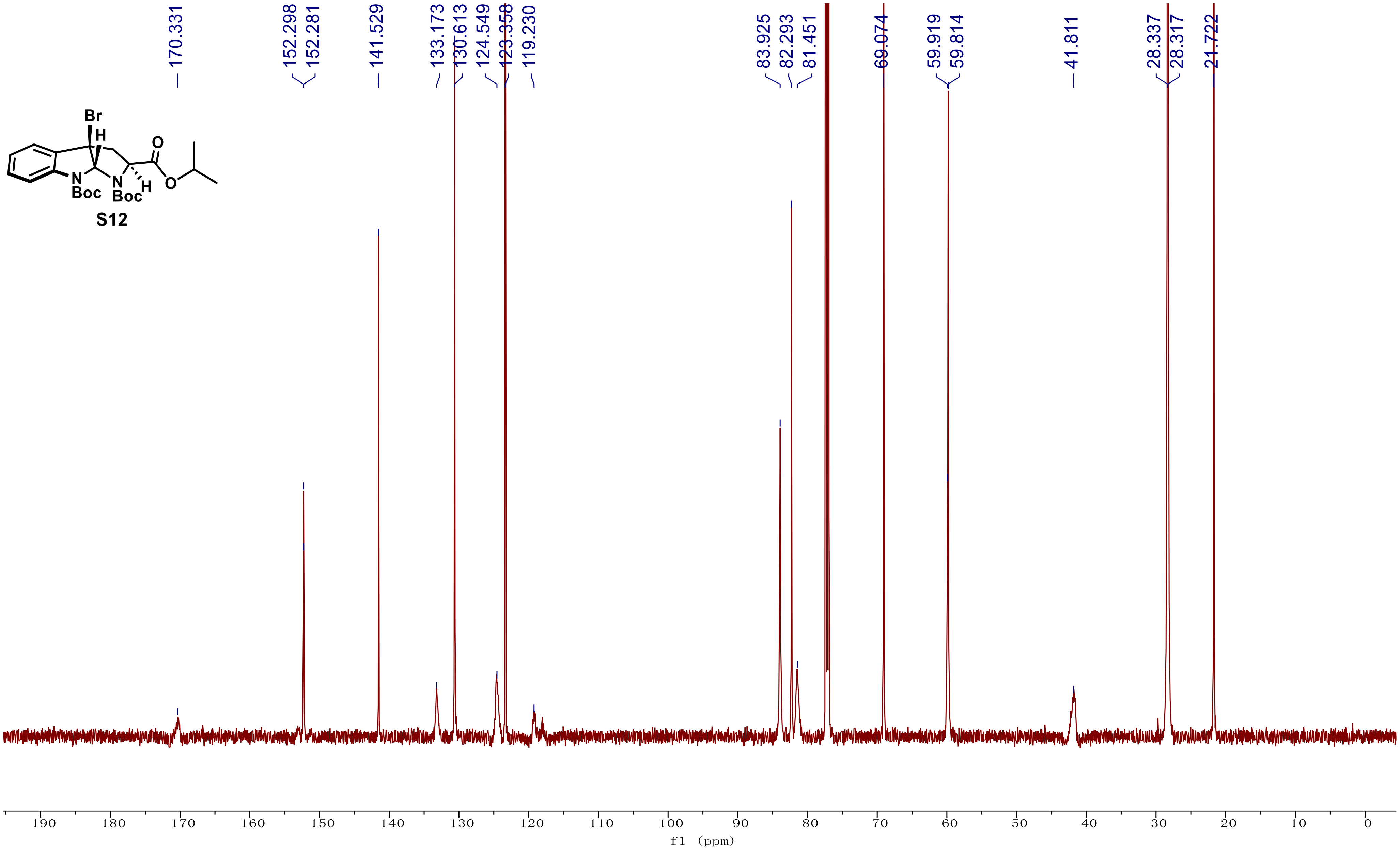

Compound 12 ^{1}H NMR (400 MHz, CD_3OD, 60 °C)

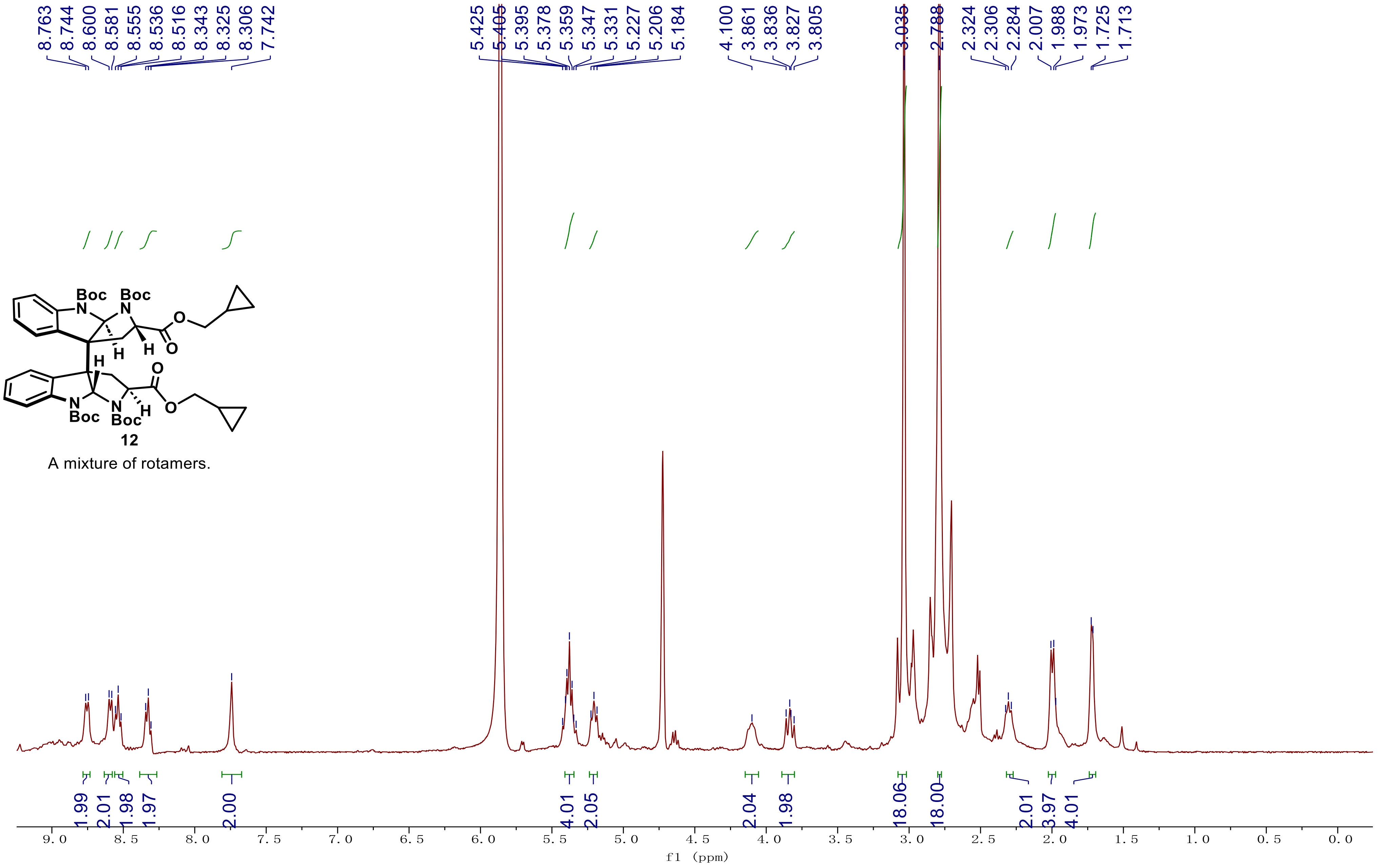

12

A mixture of rotamers.

Compound 12 ^{13}C NMR (101 MHz, CD_3OD, 60 °C)

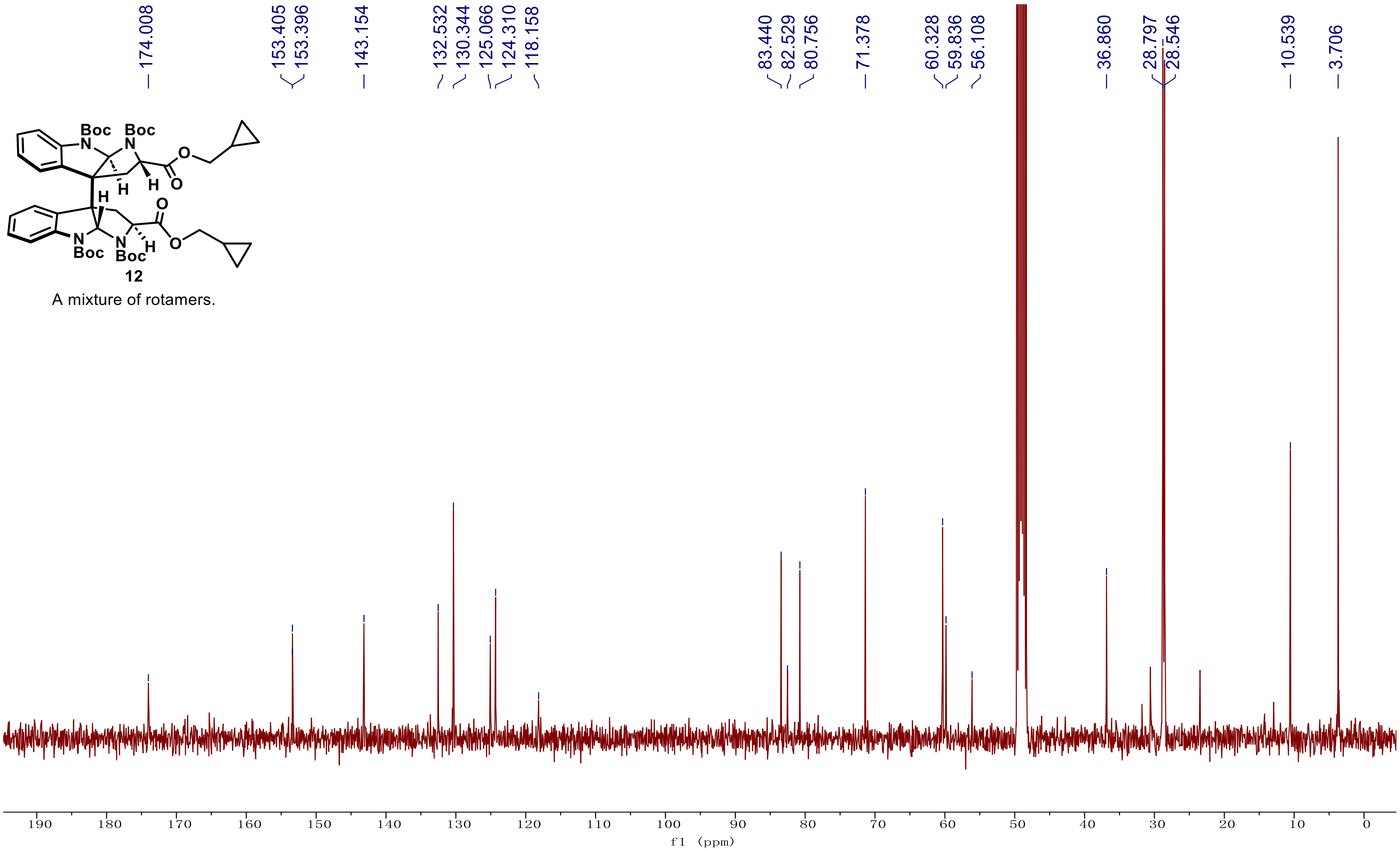

Compound 13 ^{1}H NMR (400 MHz, CD_3OD, 60 °C)

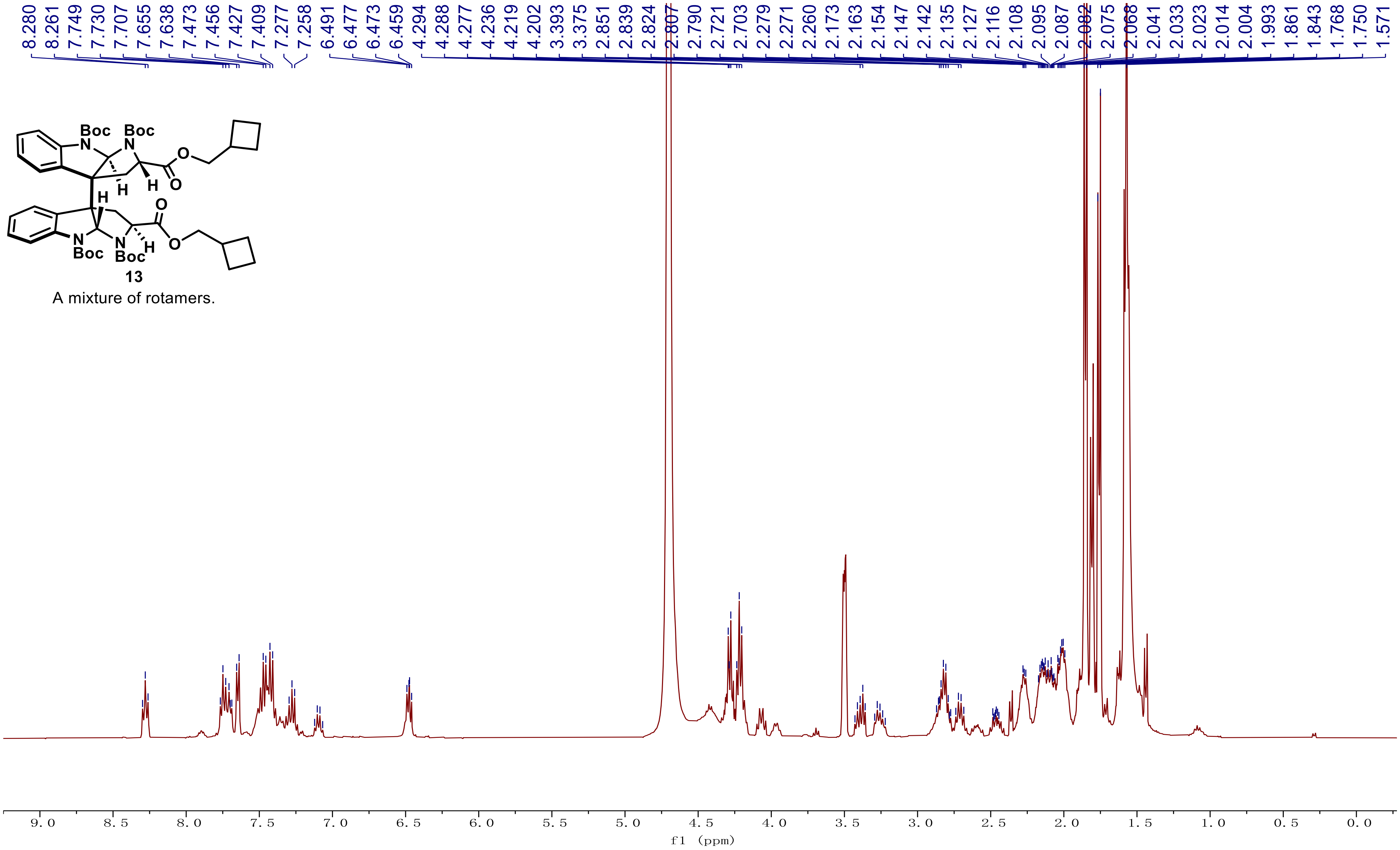

13

A mixture of rotamers.

Compound B3 ^{13}C NMR (101 MHz, CD_3OD, 60 °C)

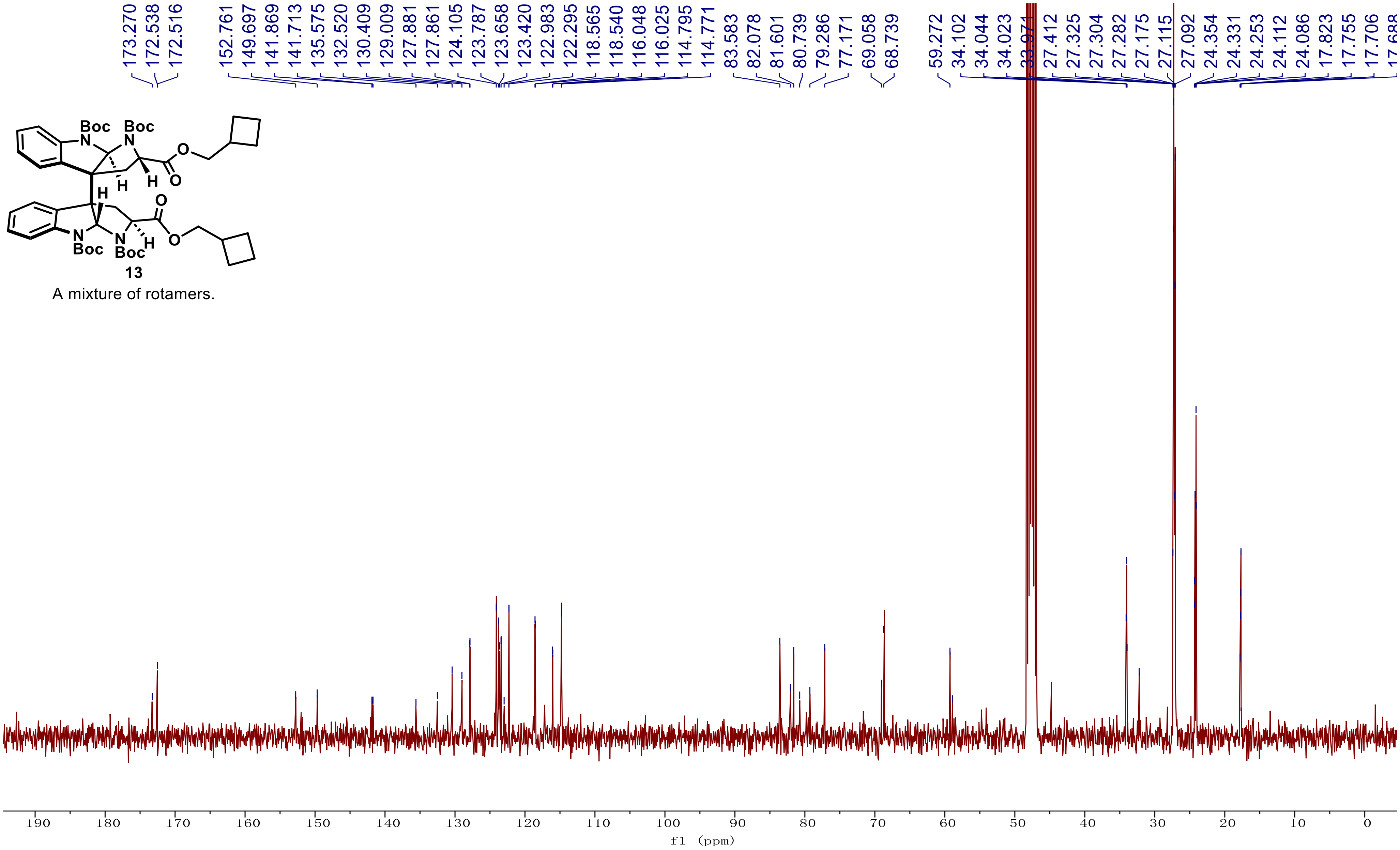

Compound 14 ^{1}H NMR (400 MHz, CD_3OD, 60 °C)

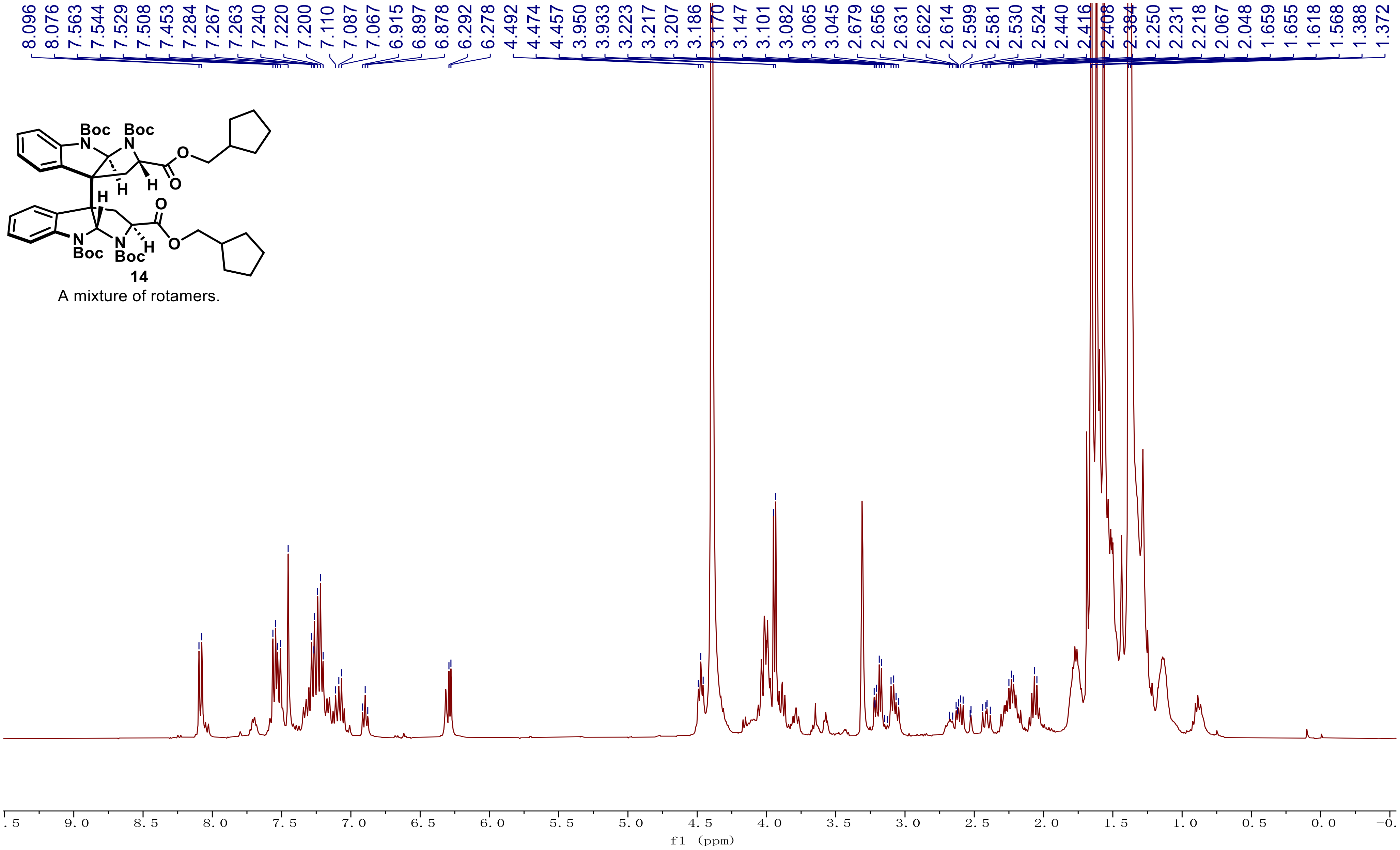

Compound 14 ^{13}C NMR (101 MHz, CD_3OD, 60 °C)

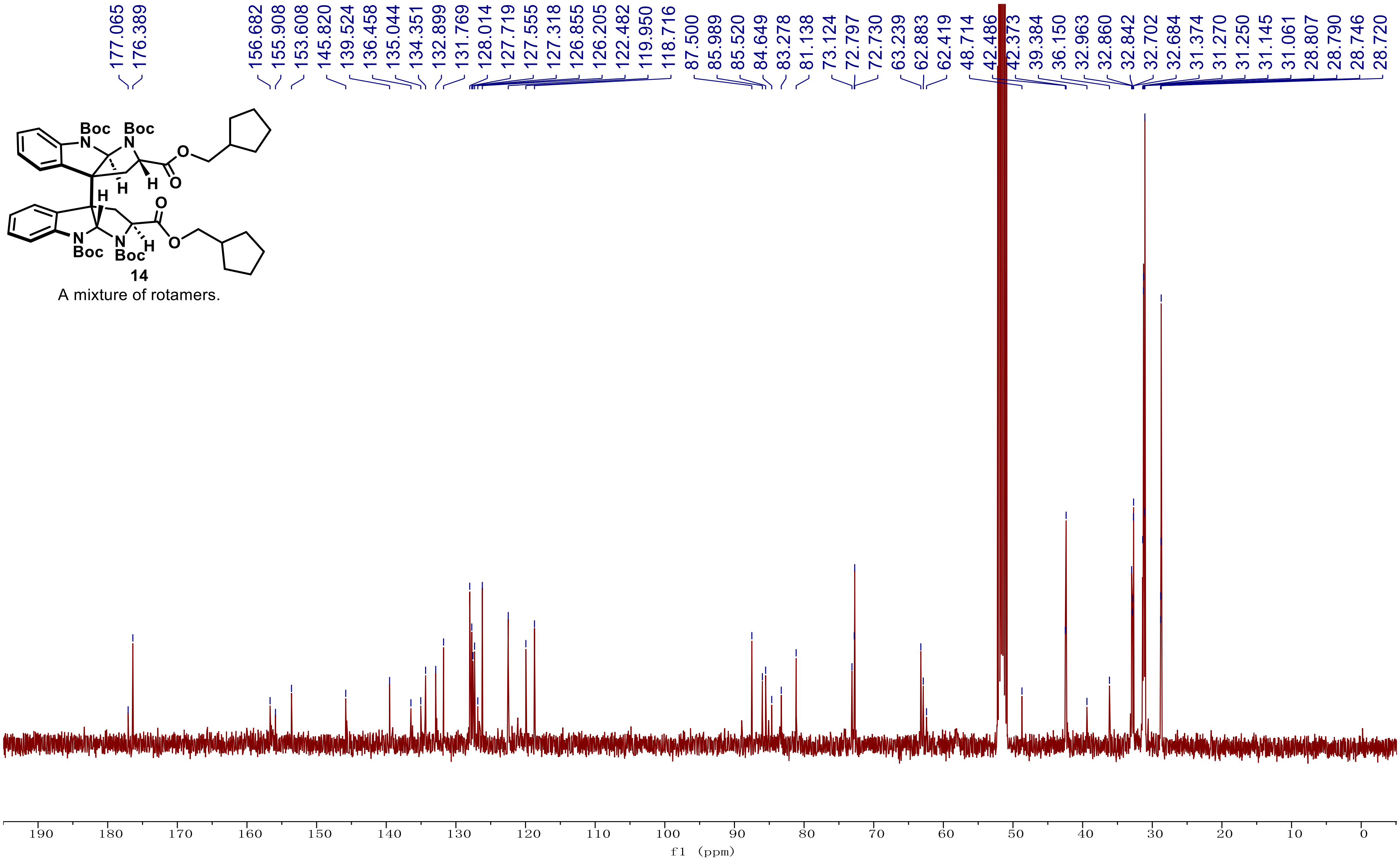

Compound 15 ^{1}H NMR (400 MHz, DMSO-*d6*, 70 °C)

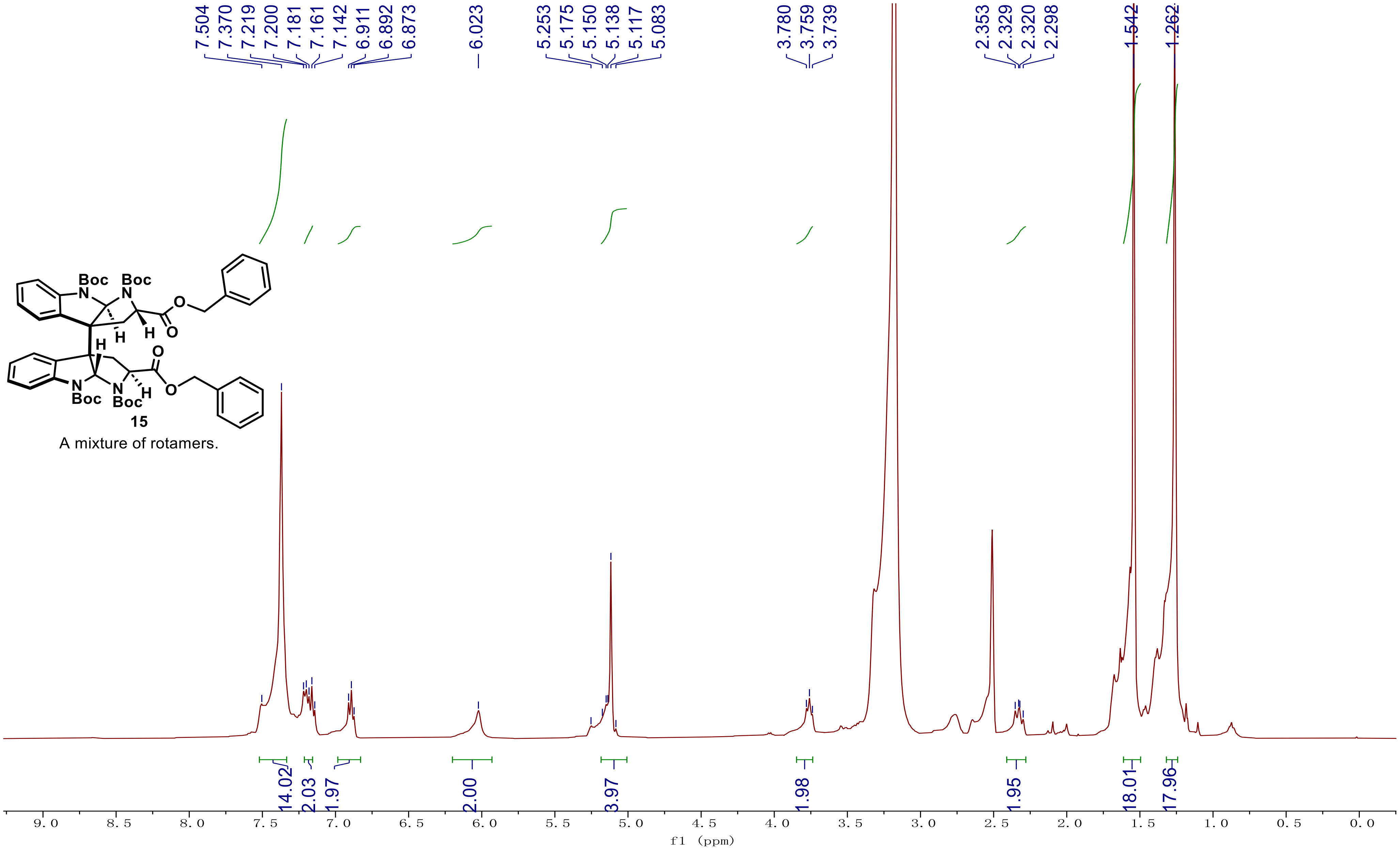

Compound 15 ^{13}C NMR (101 MHz, DMSO-*d6*, 70 °C)

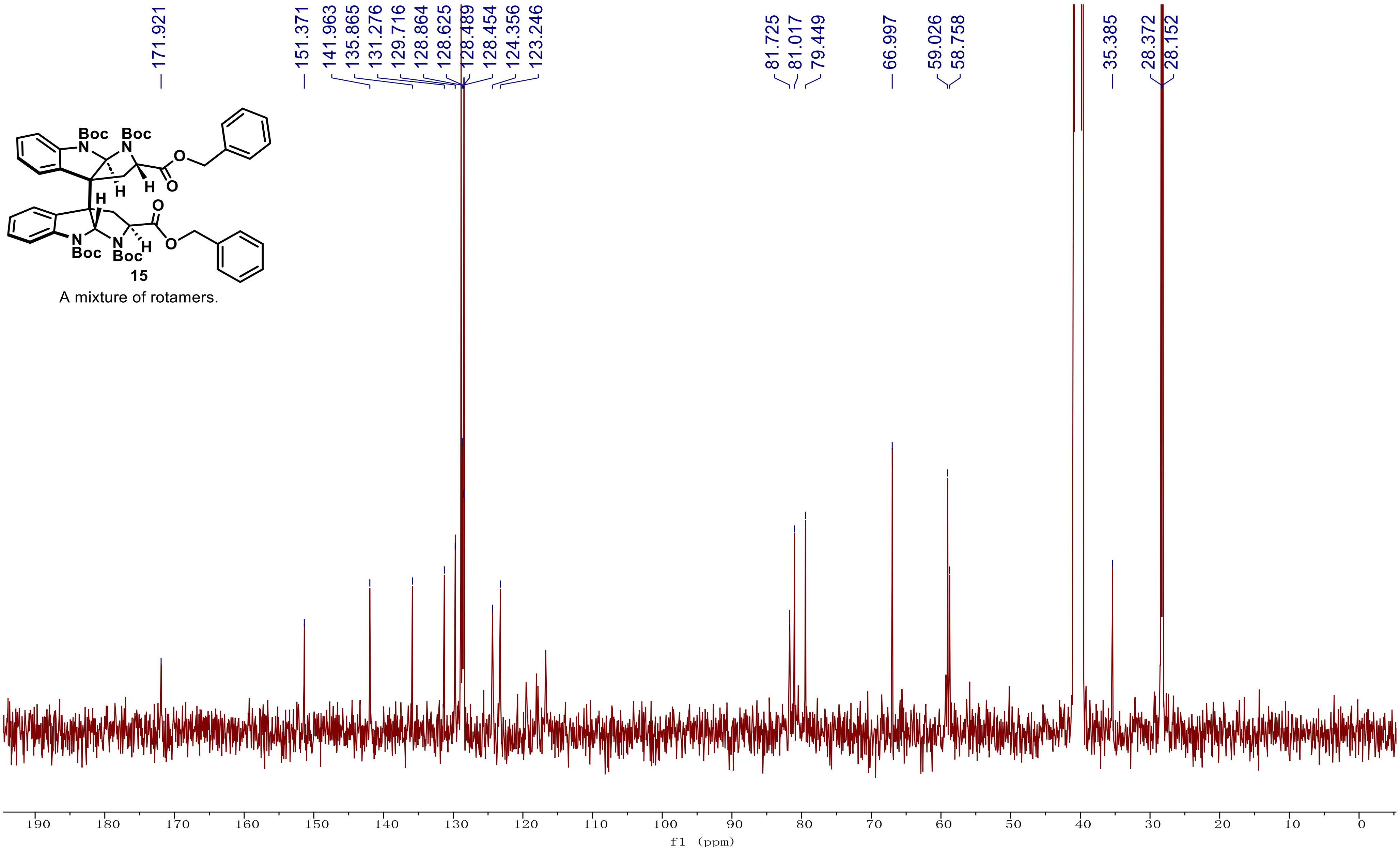

15

A mixture of rotamers.

Compound 16 ^{1}H NMR (400 MHz, CD_3OD, 60 °C)

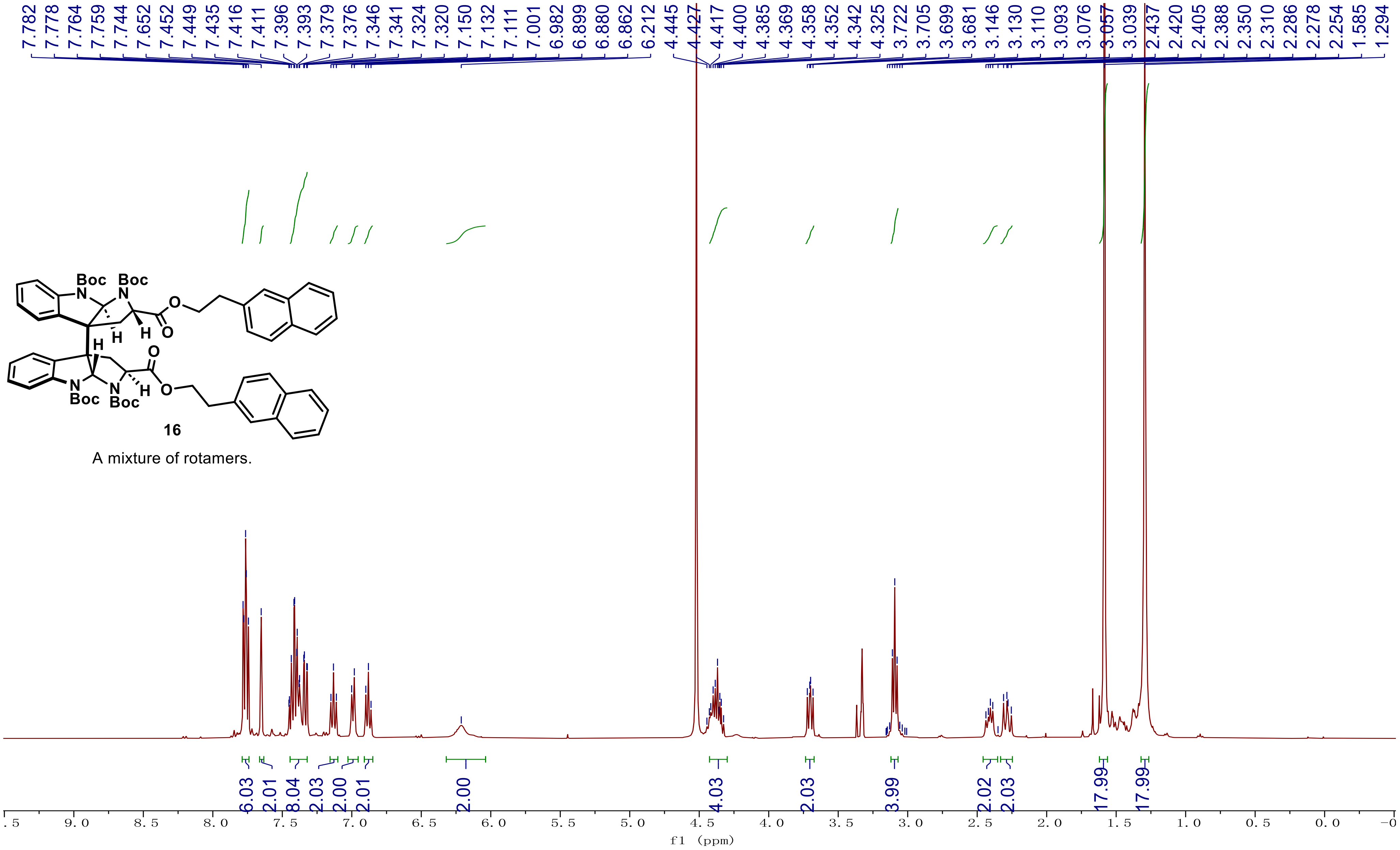

Compound 16 ^{13}C NMR (101 MHz, CD_3OD, 60 °C)

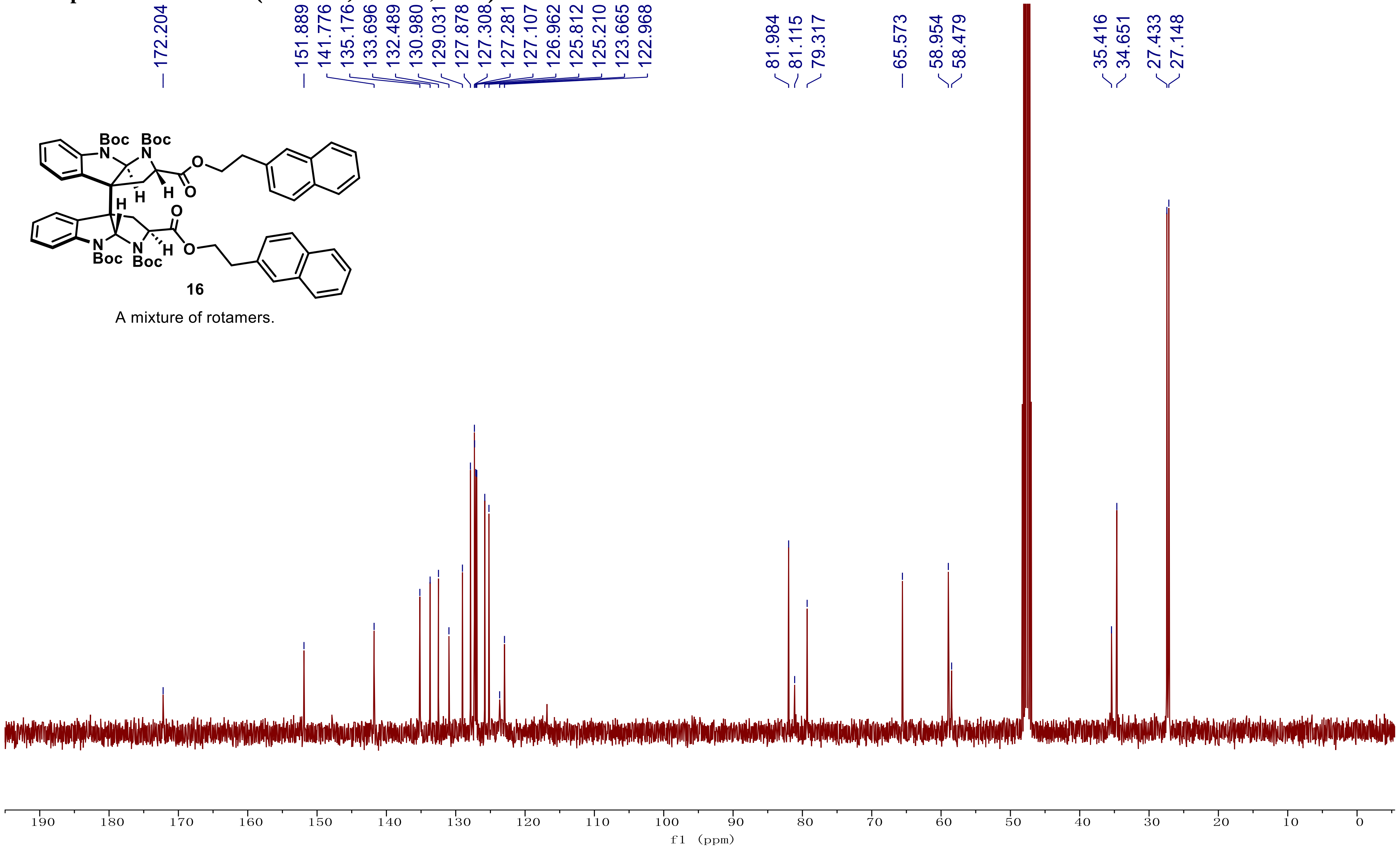

Compound 17 ^{1}H NMR (400 MHz, CD_3OD, 60 °C)

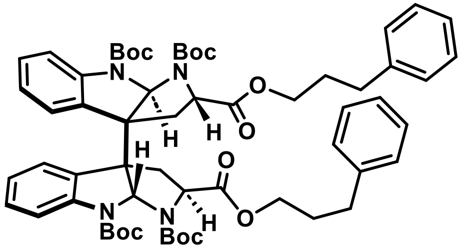

17

A mixture of rotamers.

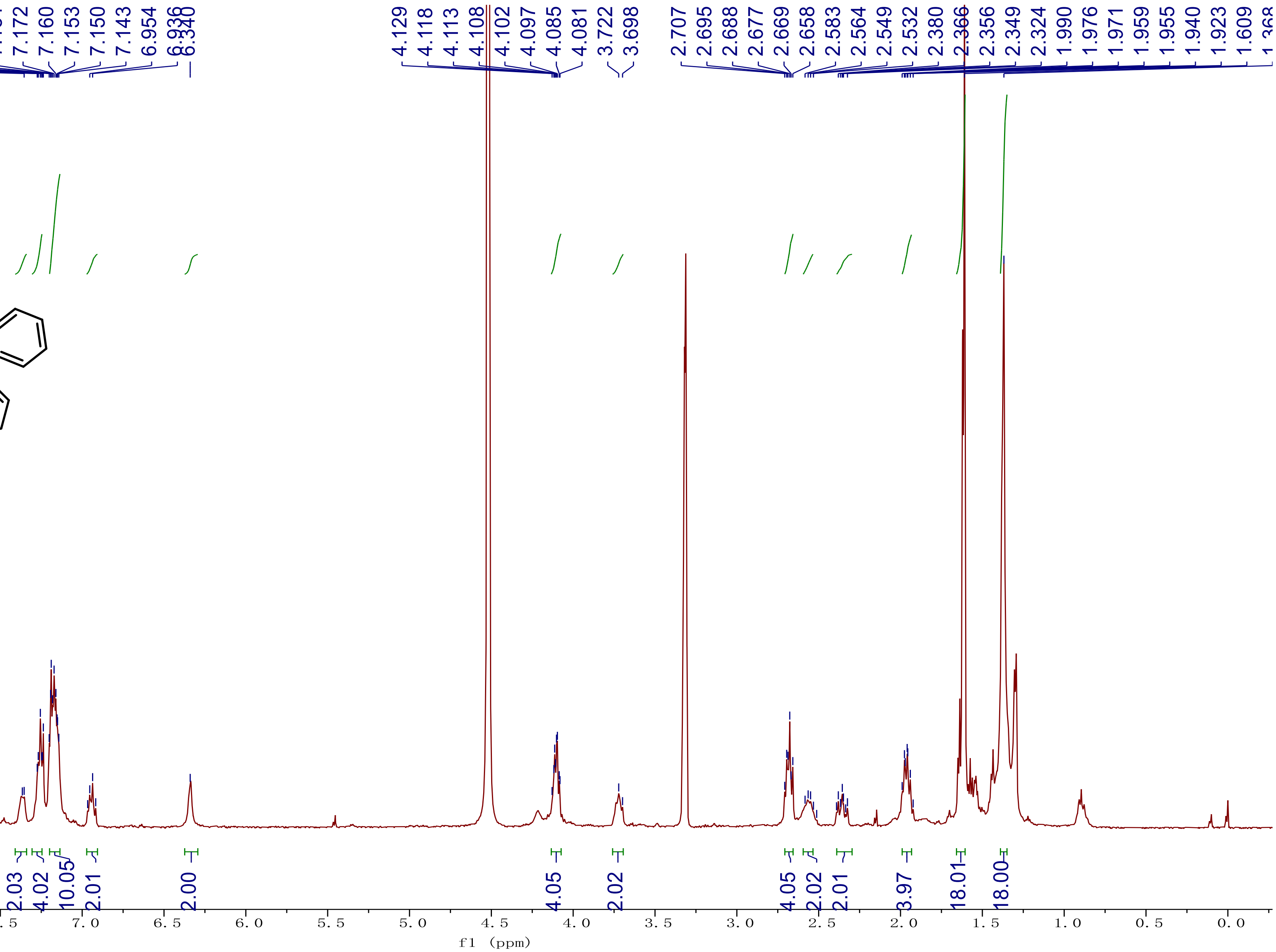

Compound 17 ^{13}C NMR (101 MHz, CD_3OD, 60 °C)

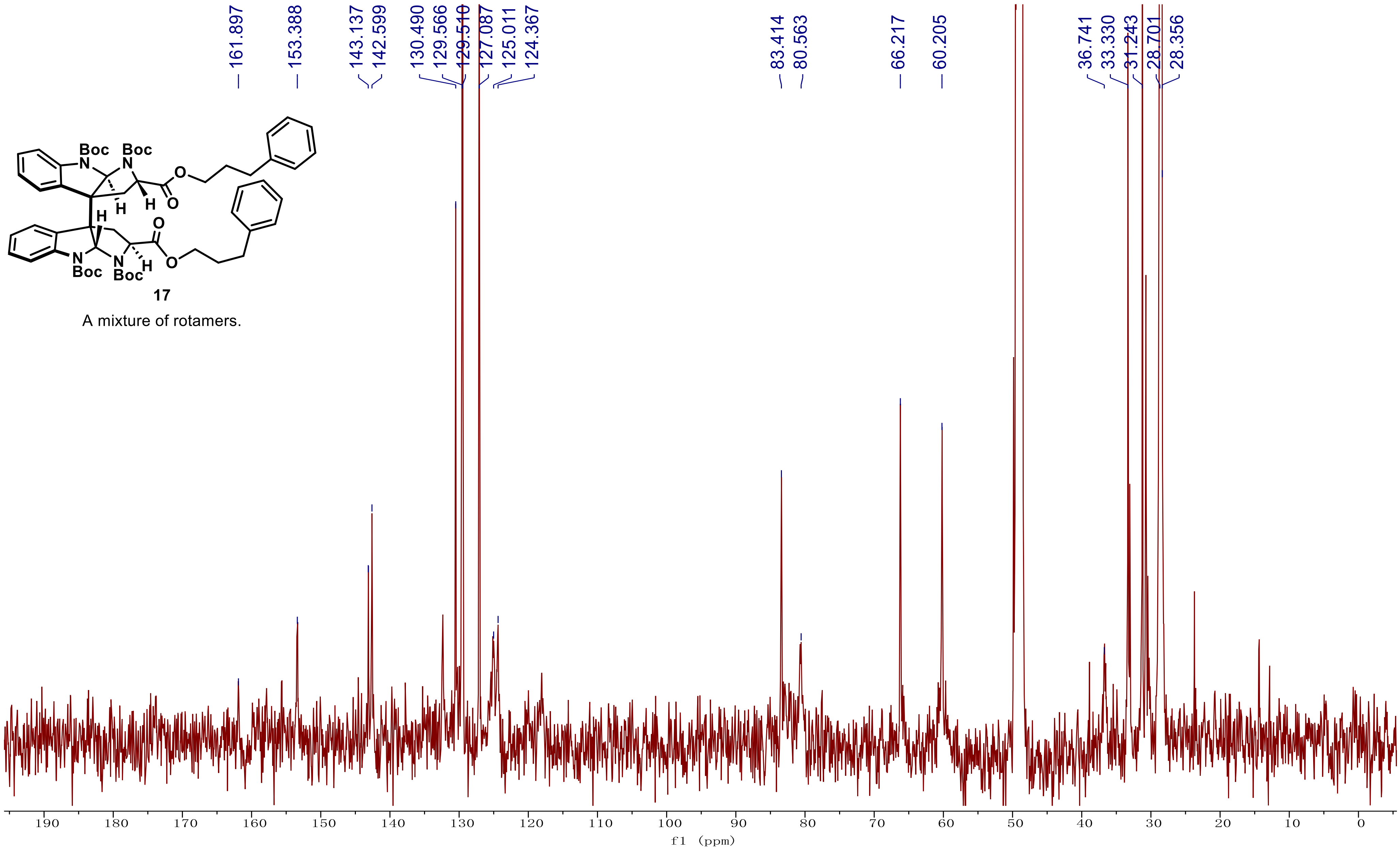

17

A mixture of rotamers.

Compound 18 ^{1}H NMR (400 MHz, CD_3OD, 60 °C)

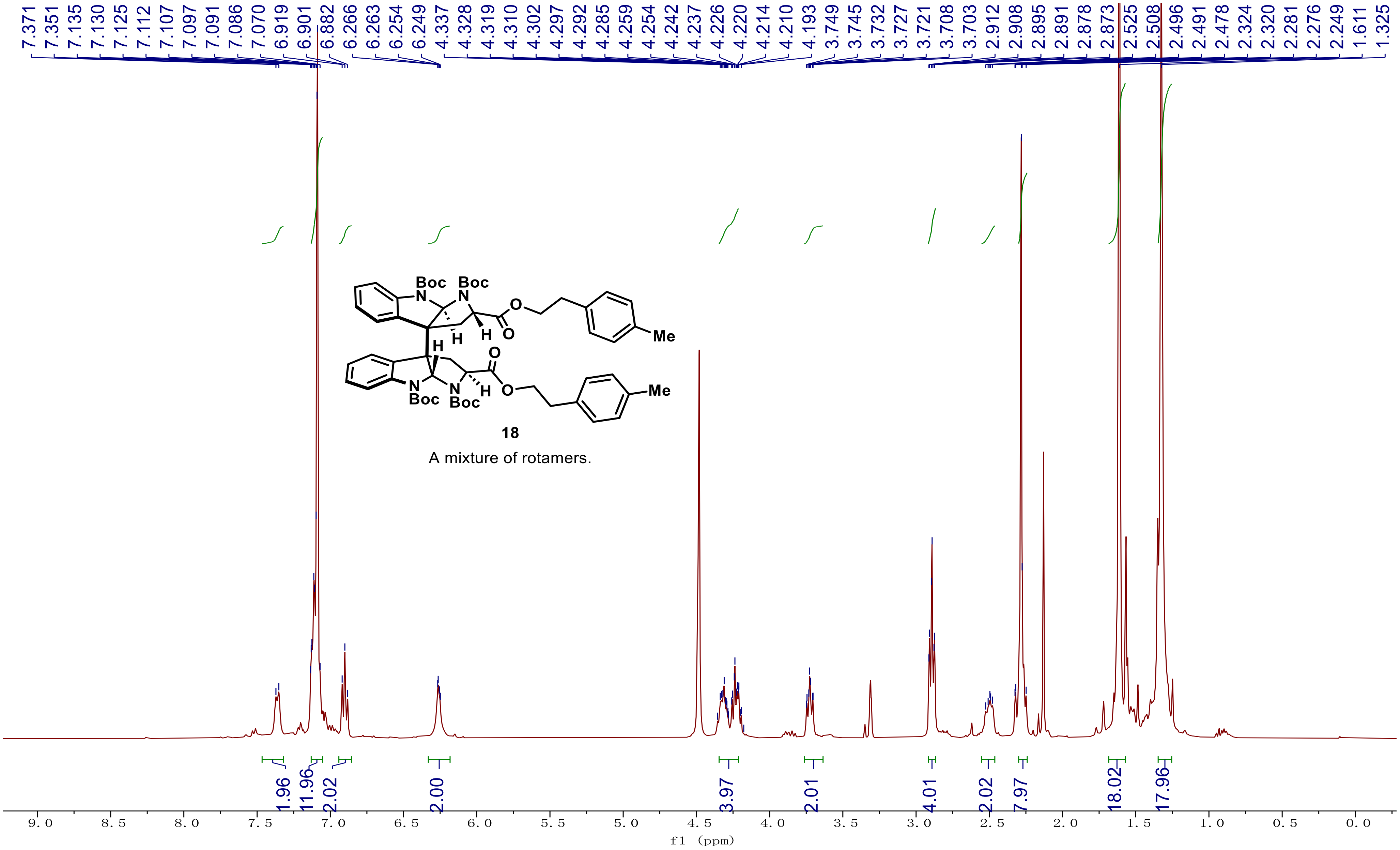

Compound 18 ^{13}C NMR (101 MHz, CD_3OD, 60 °C)

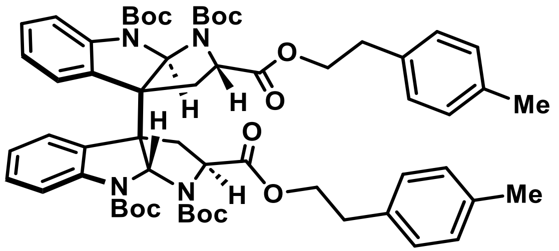

18

A mixture of rotamers.

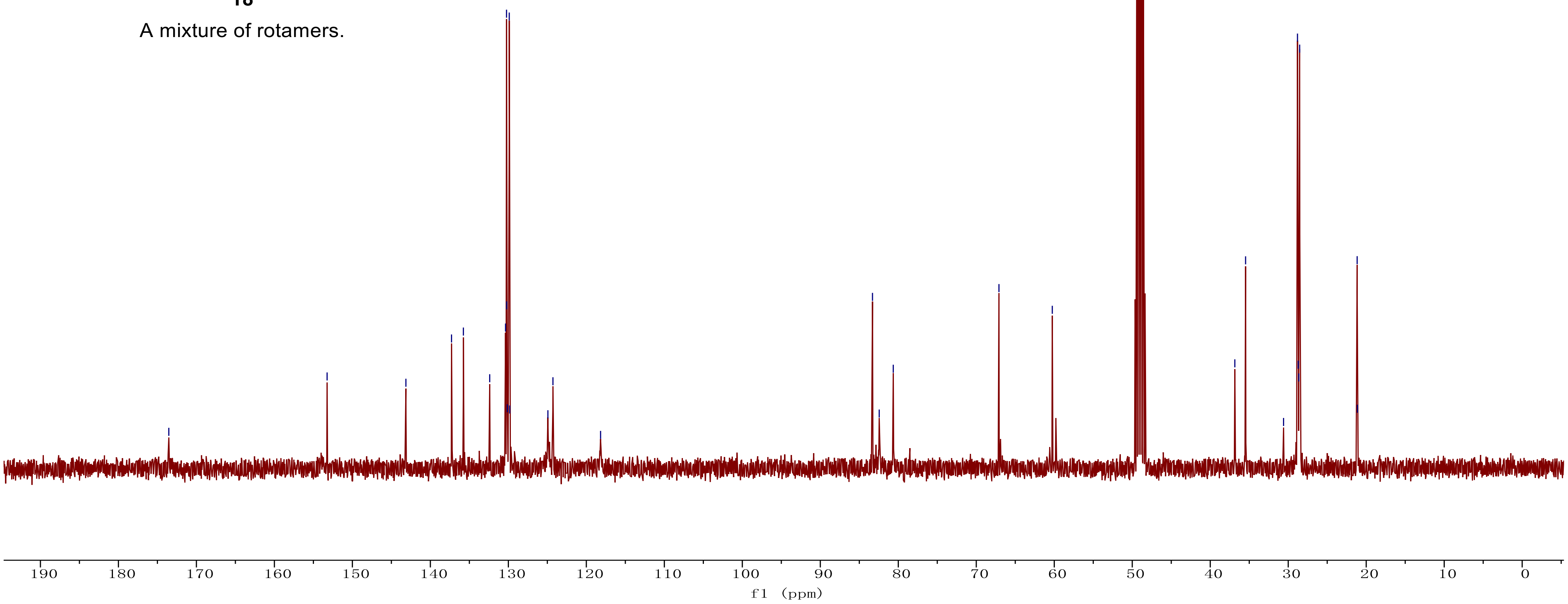

Compound 19 ^{1}H NMR (400 MHz, CD_3OD, 60 °C)

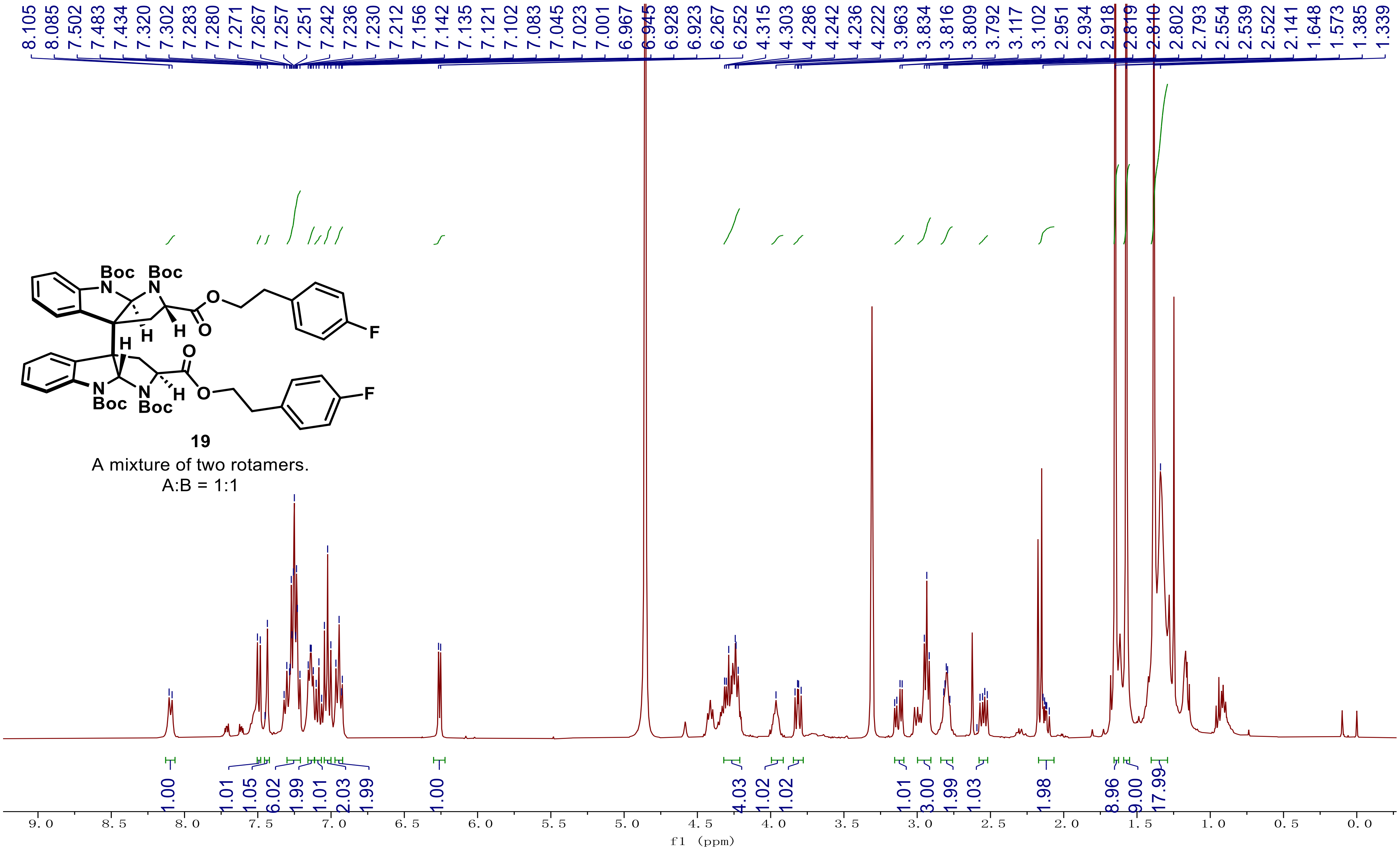

Compound B12 ^{13}C NMR (101 MHz, CD_3OD, 60 °C)

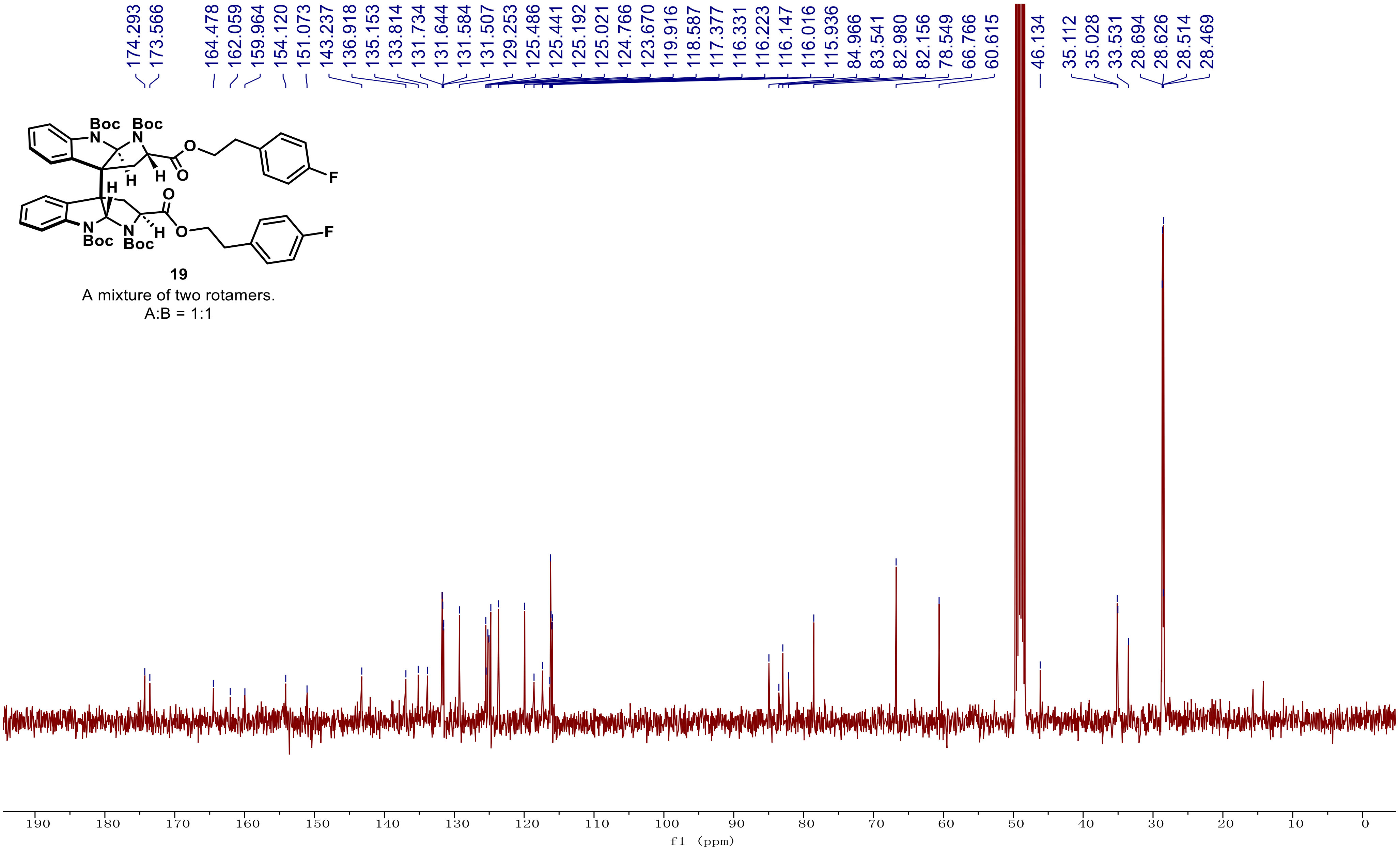

19

A mixture of two rotamers.

A:B = 1:1

Compound B12 ^{19}F NMR (377 MHz, CD_3OD, 60 °C)

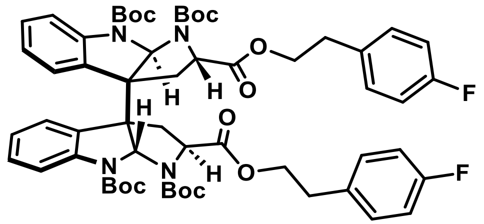

19

A mixture of two rotamers.

A:B = 1:1

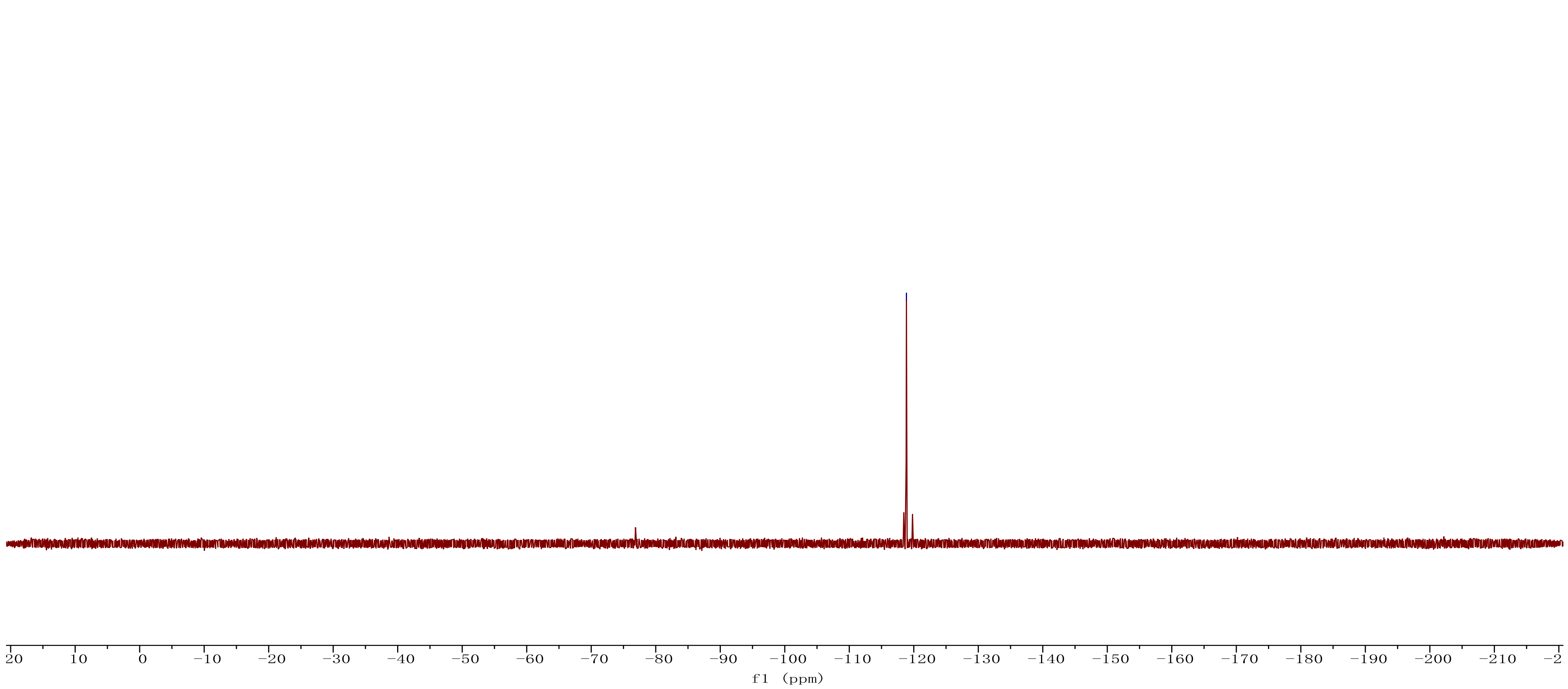

Compound 20 ^{1}H NMR (400 MHz, CD_3OD, 60 °C)

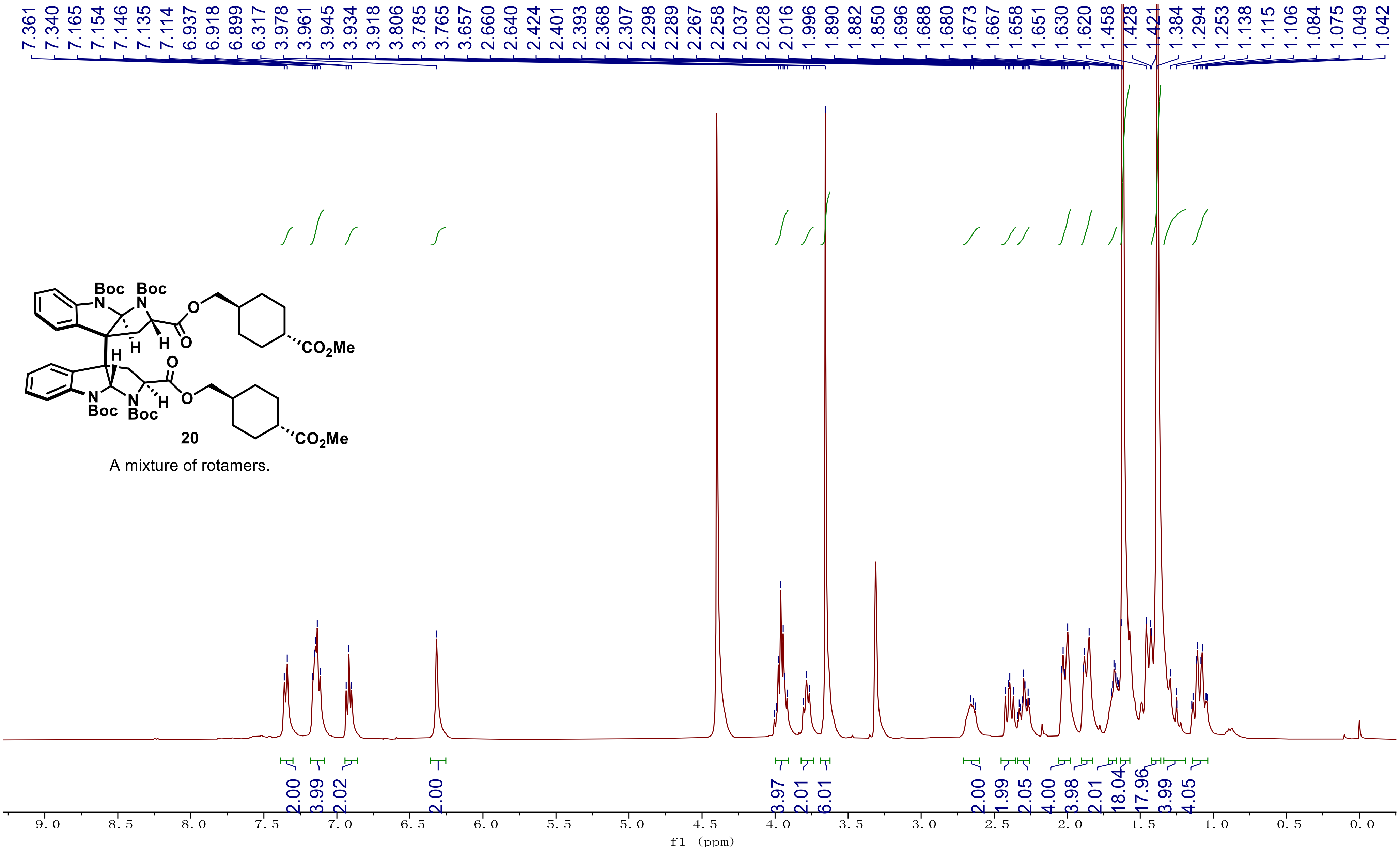

Compound B10 ^{13}C NMR (101 MHz, CD$_3$OD, 60 °C)

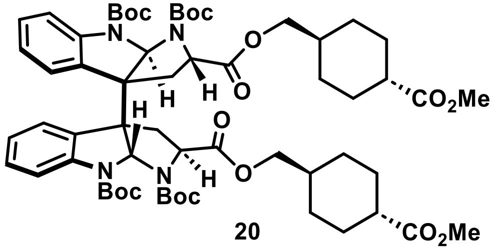

20

A mixture of rotamers.

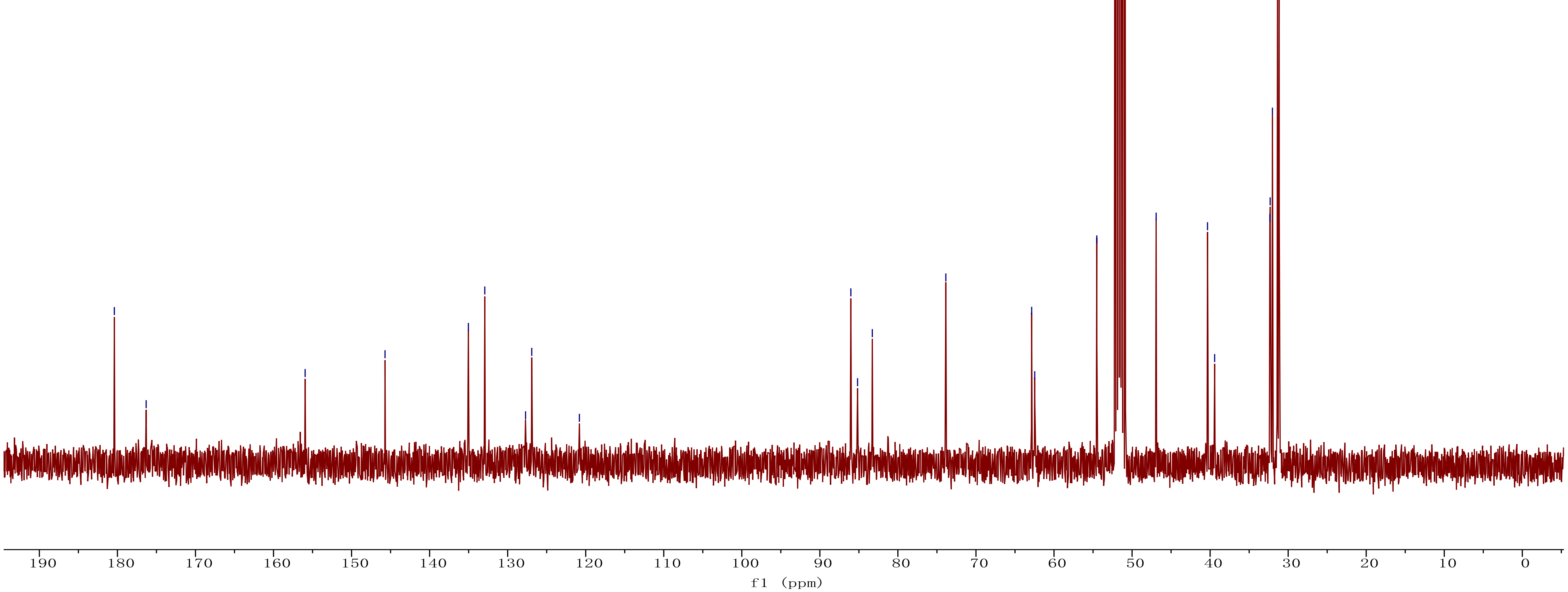

Compound 21 ^{1}H NMR (400 MHz, CD_3OD, 60 °C)

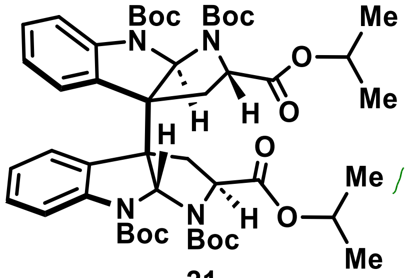

21
A mixture of rotamers.

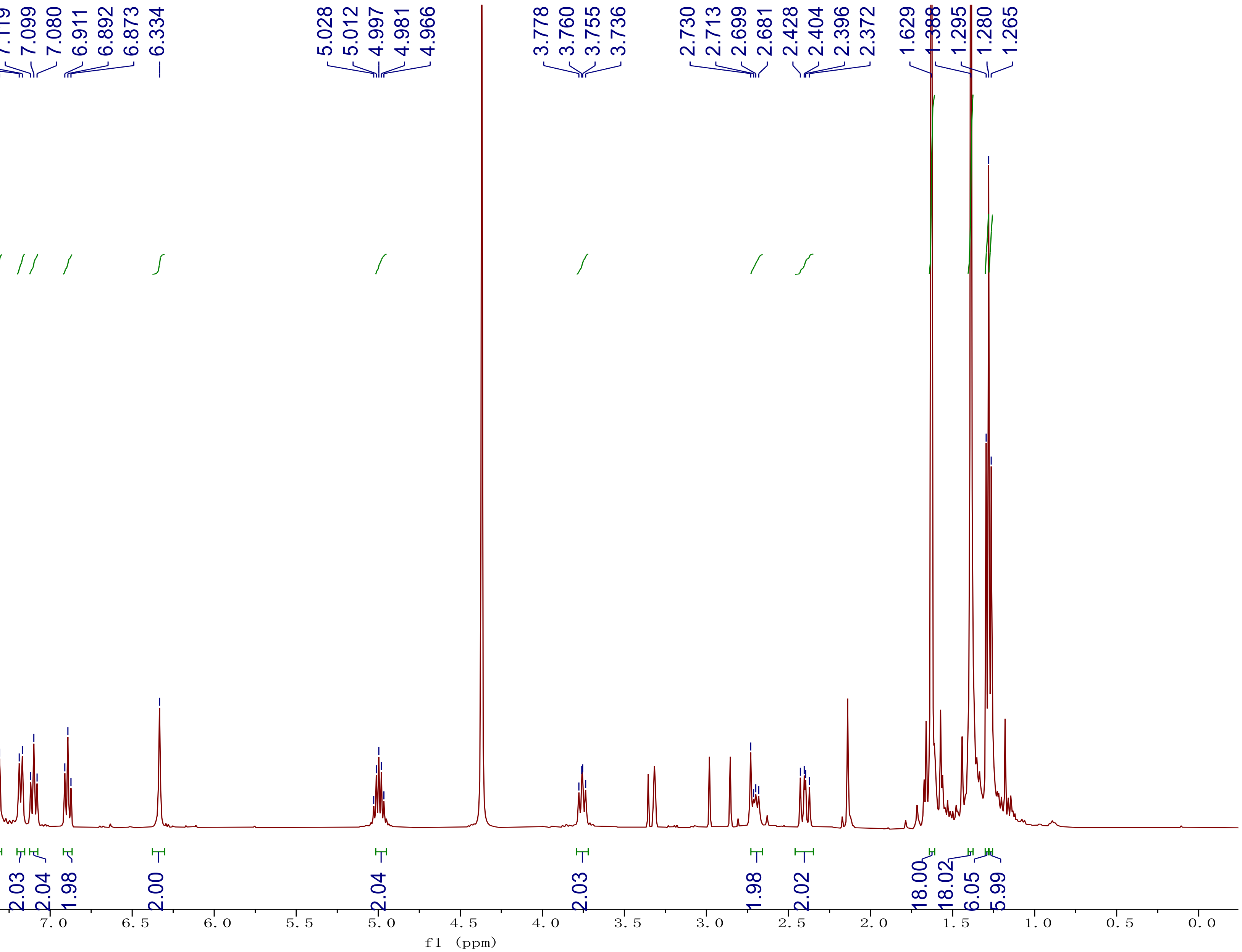

Compound 21 ^{13}C NMR (101 MHz, CD_3OD, 60 °C)

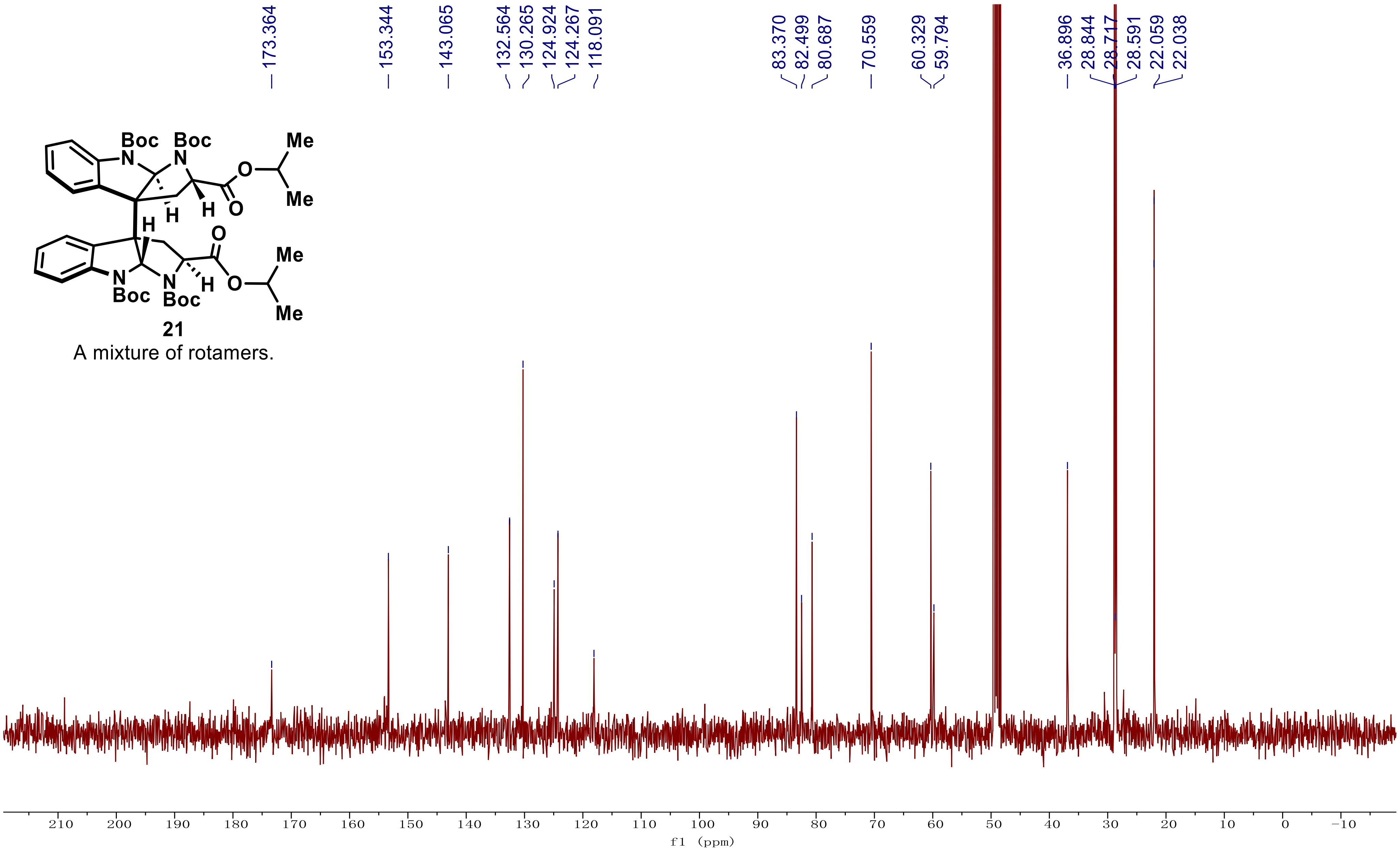

Compound 22 ^{1}H NMR (600 MHz, $CDCl_3$)

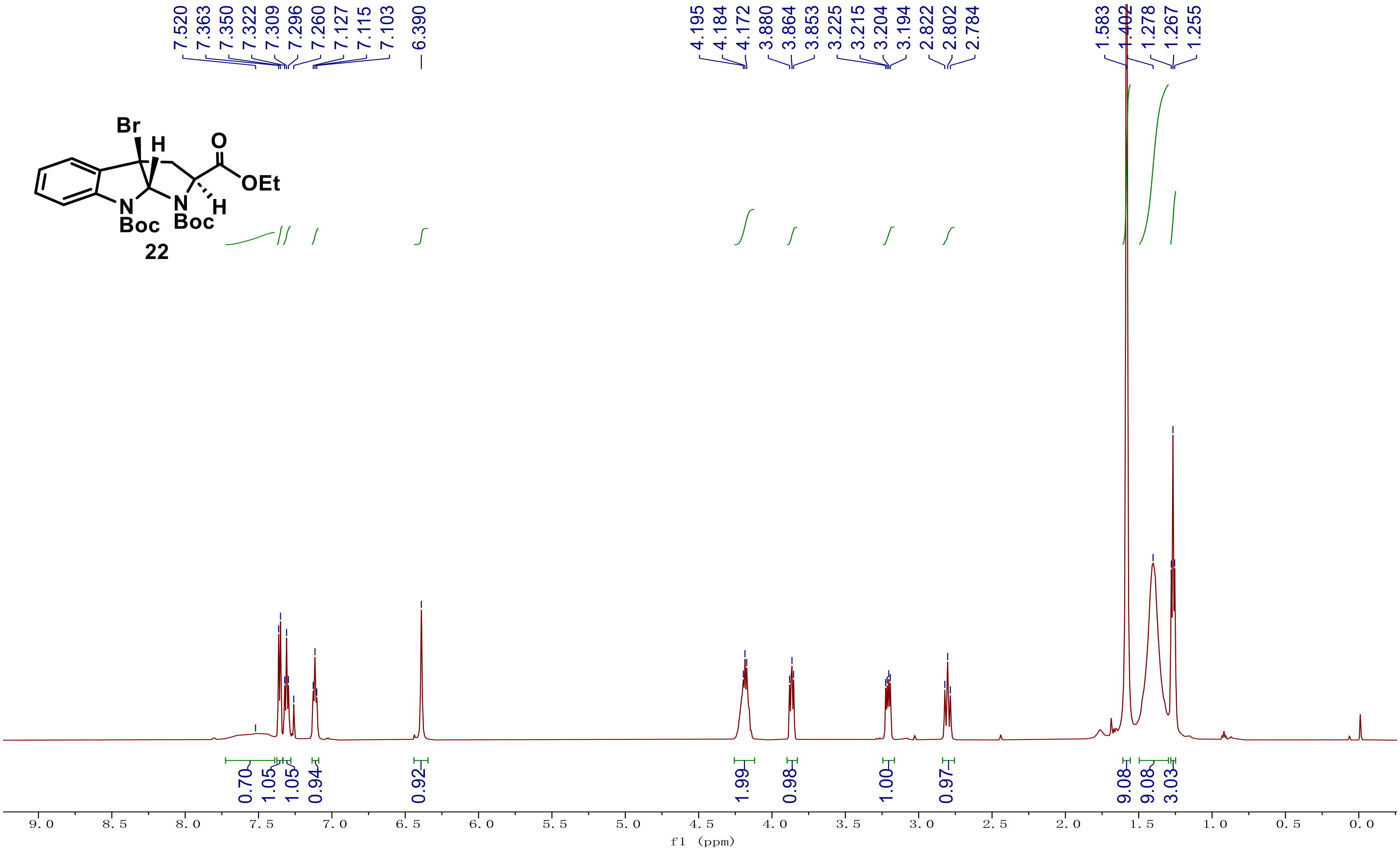

Compound 22 ^{13}C NMR (151 MHz, CDCl$_3$)

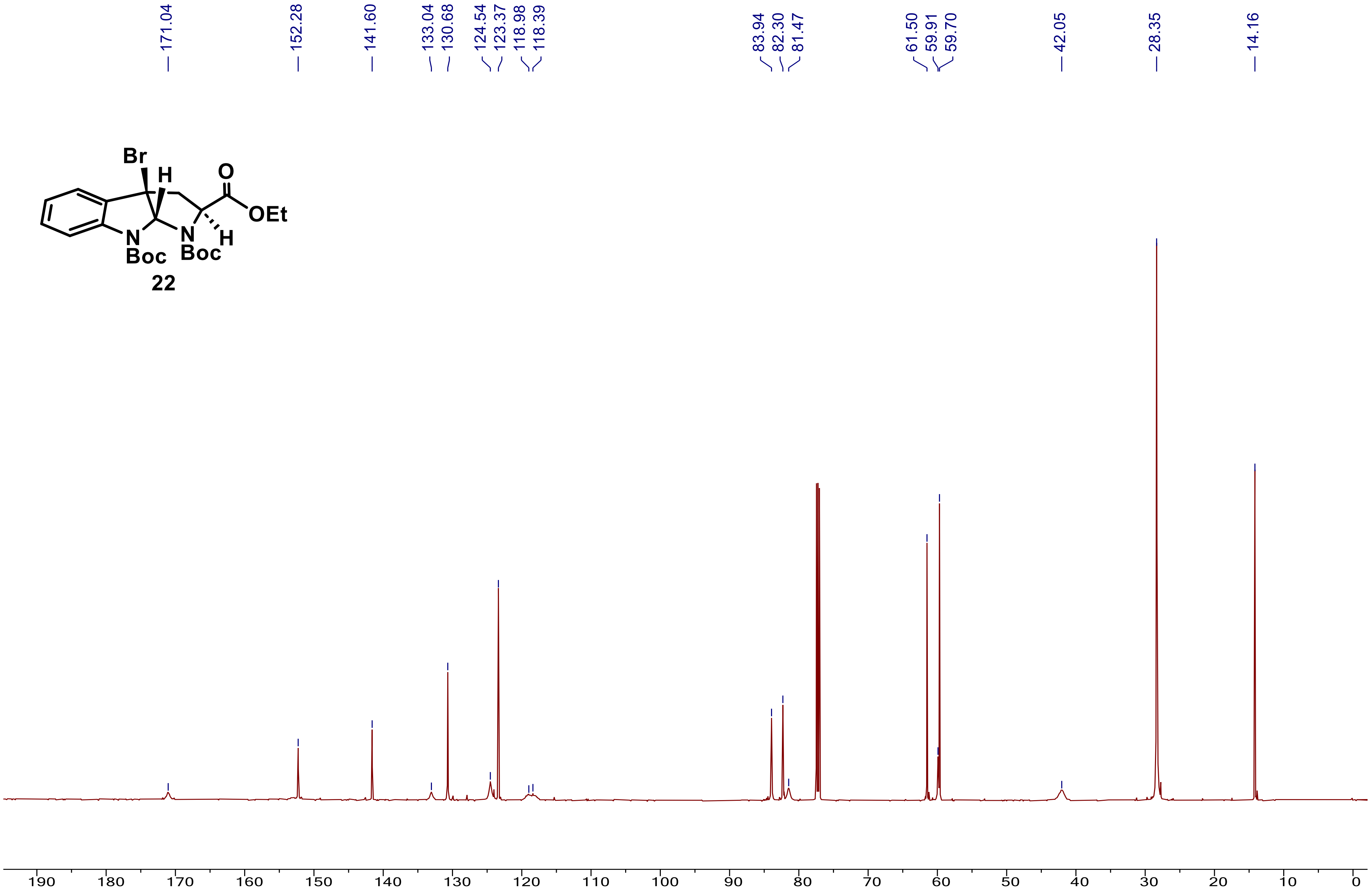

Compound 23 ^{1}H NMR (600 MHz, $CDCl_3$)

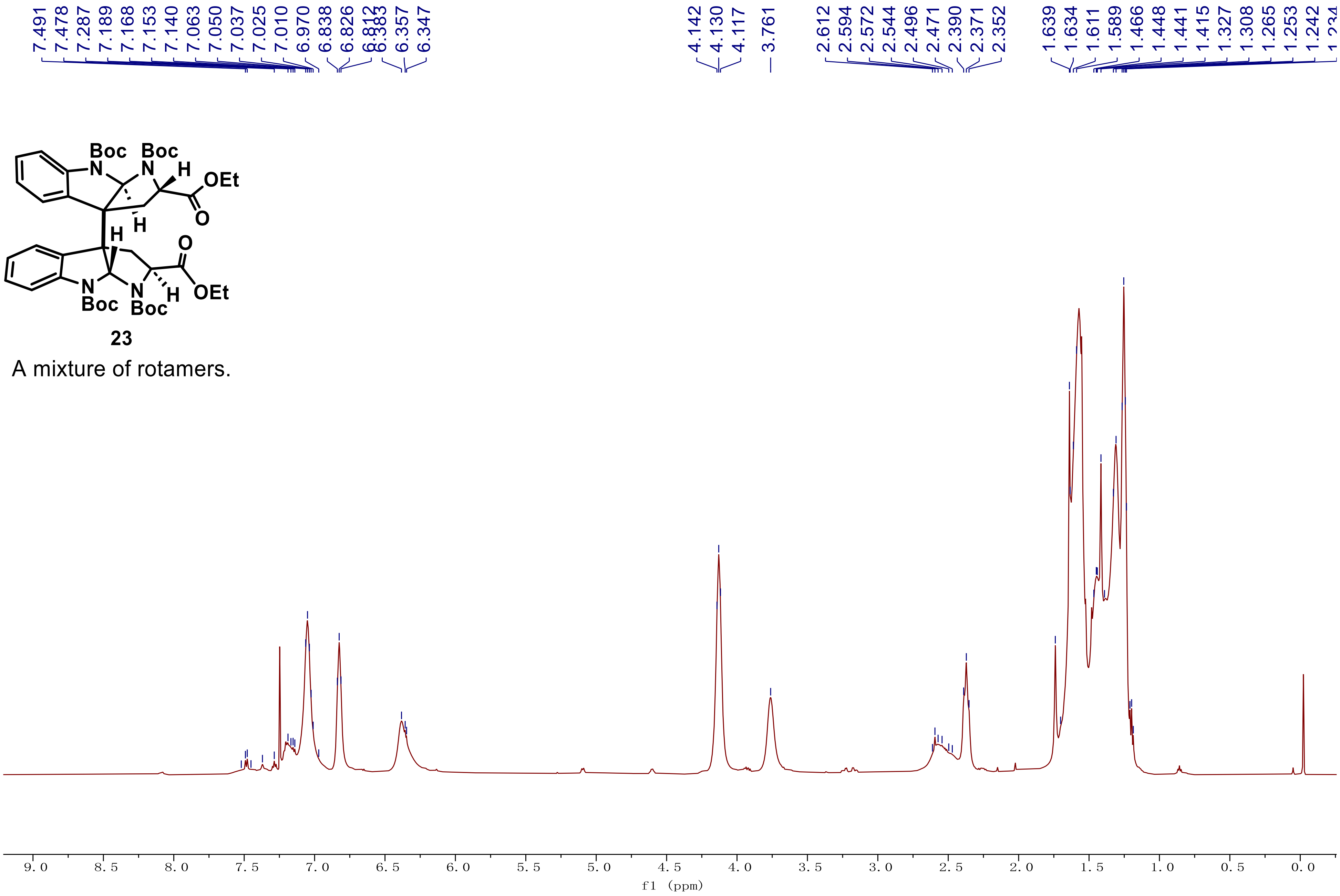

23

A mixture of rotamers.

Compound 23 ^{13}C NMR (151 MHz, CDCl$_3$)

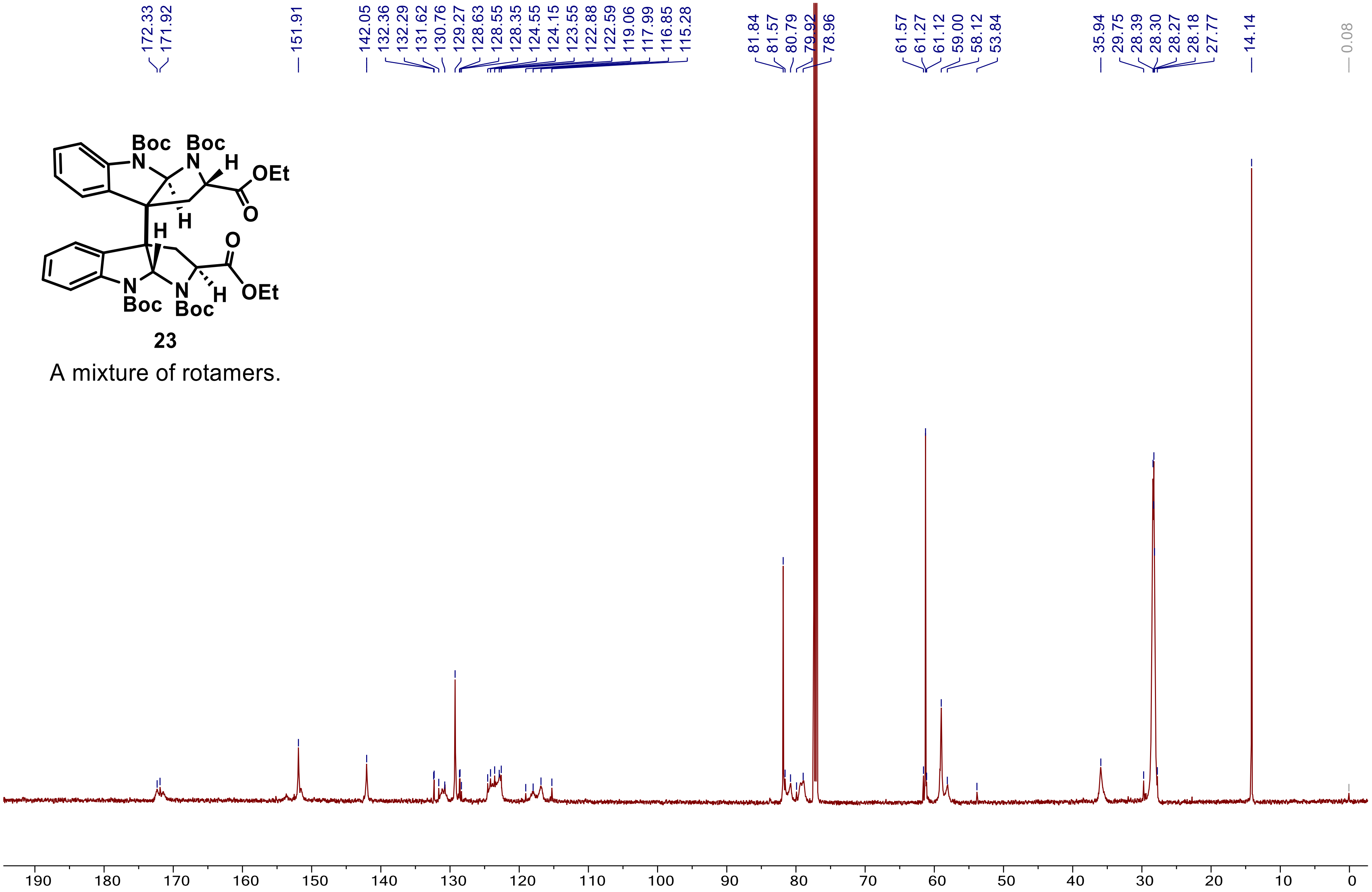

23

A mixture of rotamers.

(–)-ditryptophenaline (38) ¹H NMR (400 MHz, CDCl₃)

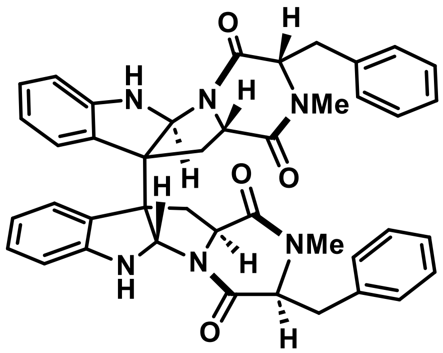

(–)-ditryptophenaline (**38**)

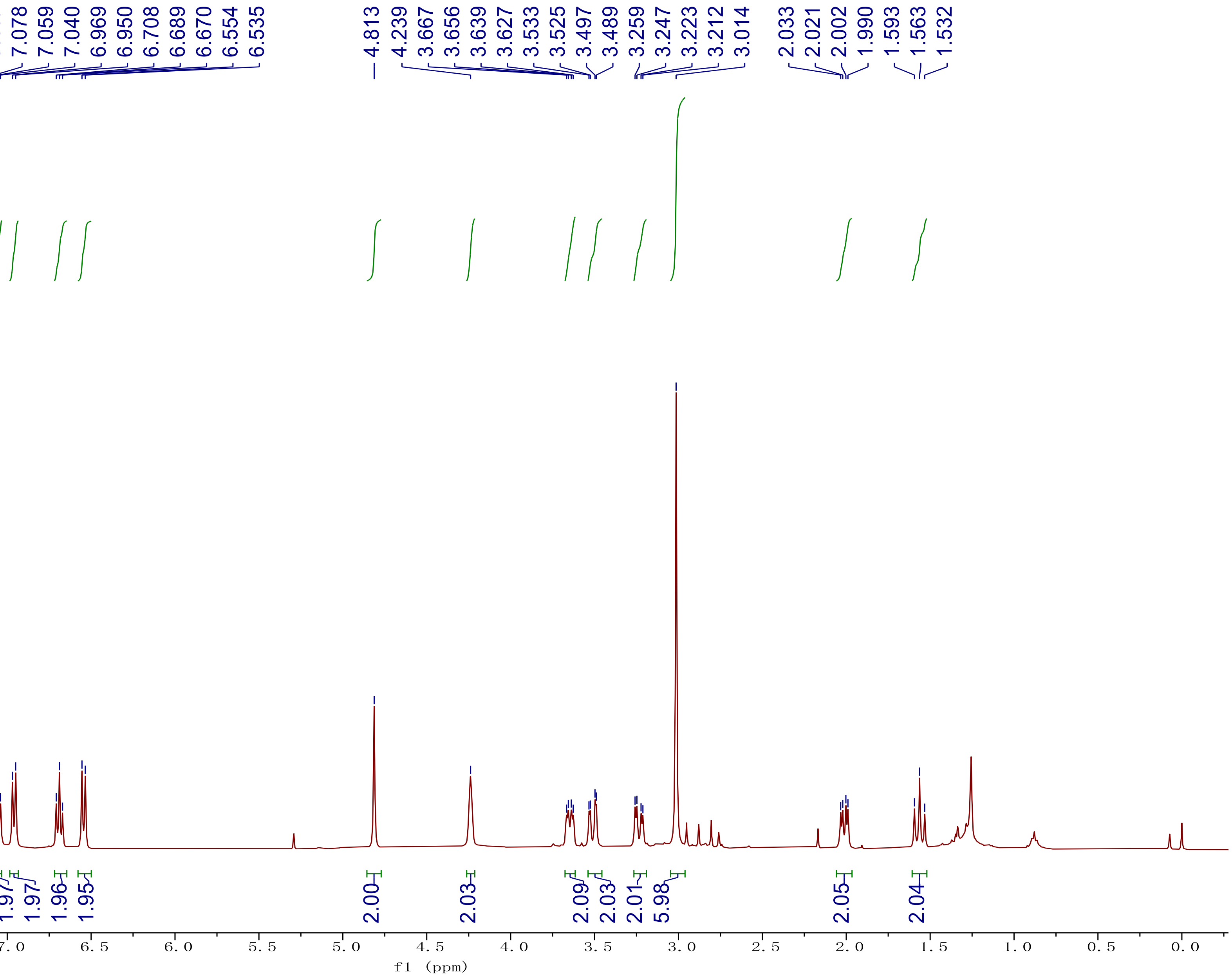

¹H NMR Comparison (–)-ditryptophenaline (38)[3]

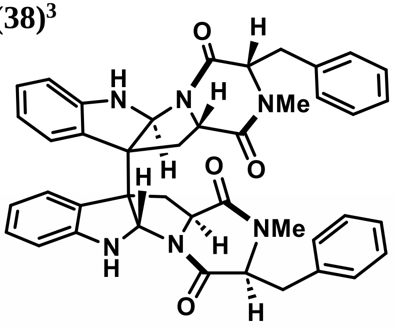

(–)-ditryptophenaline (**38**)

Reported[3]
in CDCl3 (500 MHz)

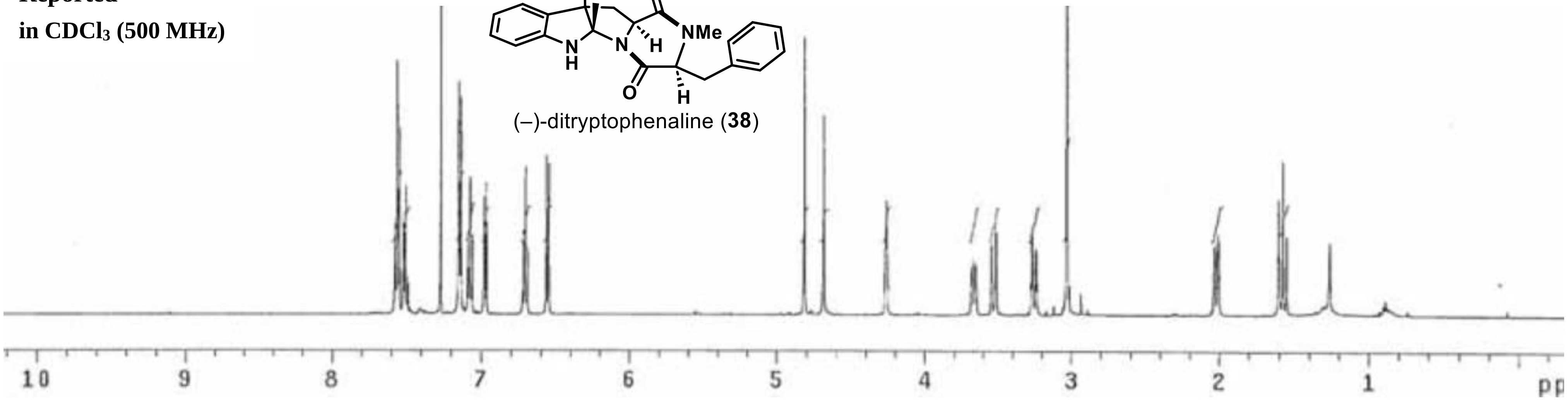

Synthetic (This work)
in CDCl3 (400 MHz)

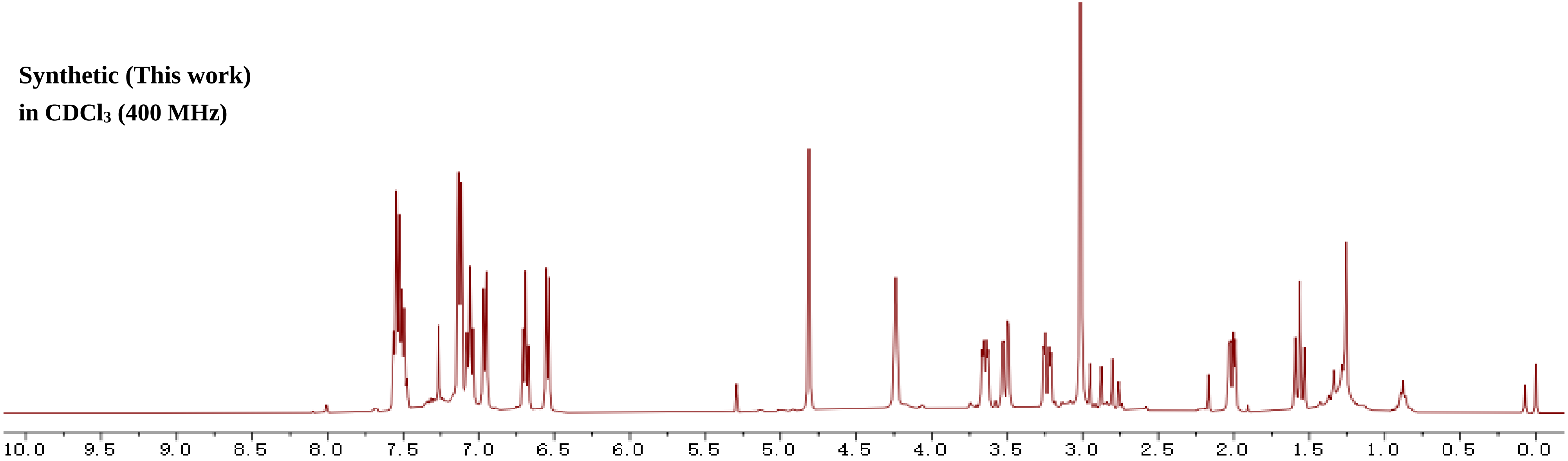

(–)-ditryptophenaline (38) ^{13}C NMR (101 MHz, CDCl$_3$)

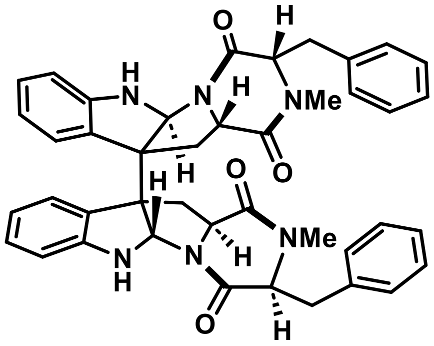

(–)-ditryptophenaline (**38**)

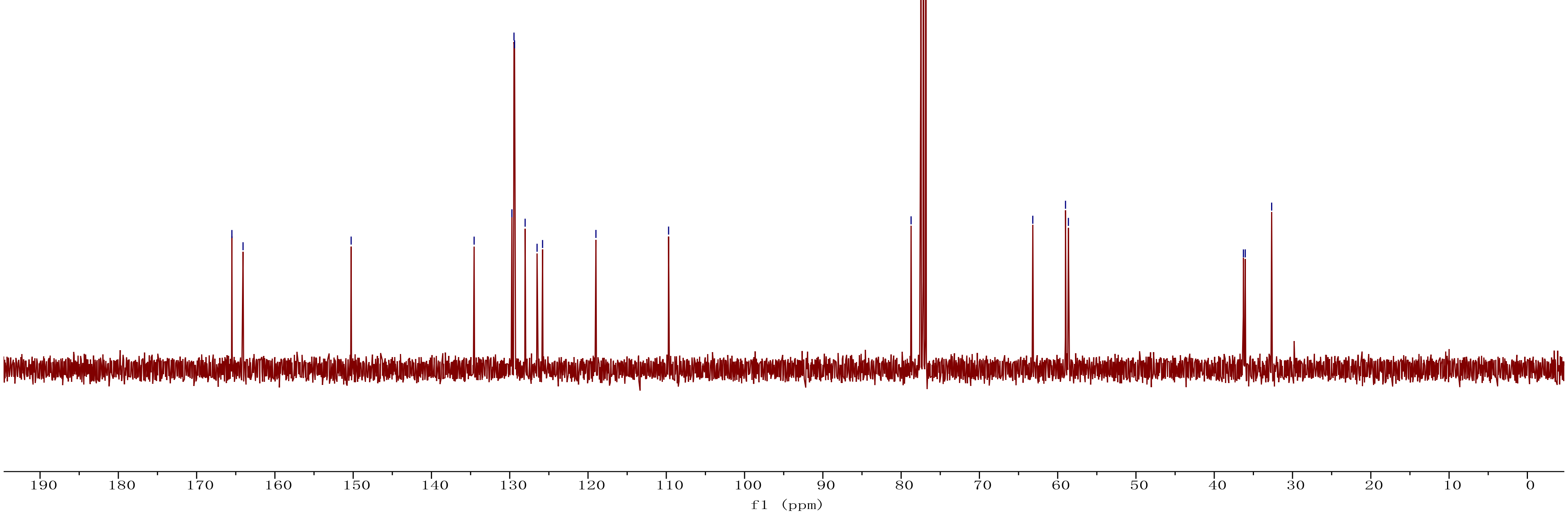

(–)-dibrevianamide F (39) ^{1}H NMR (400 MHz, $CDCl_3$)

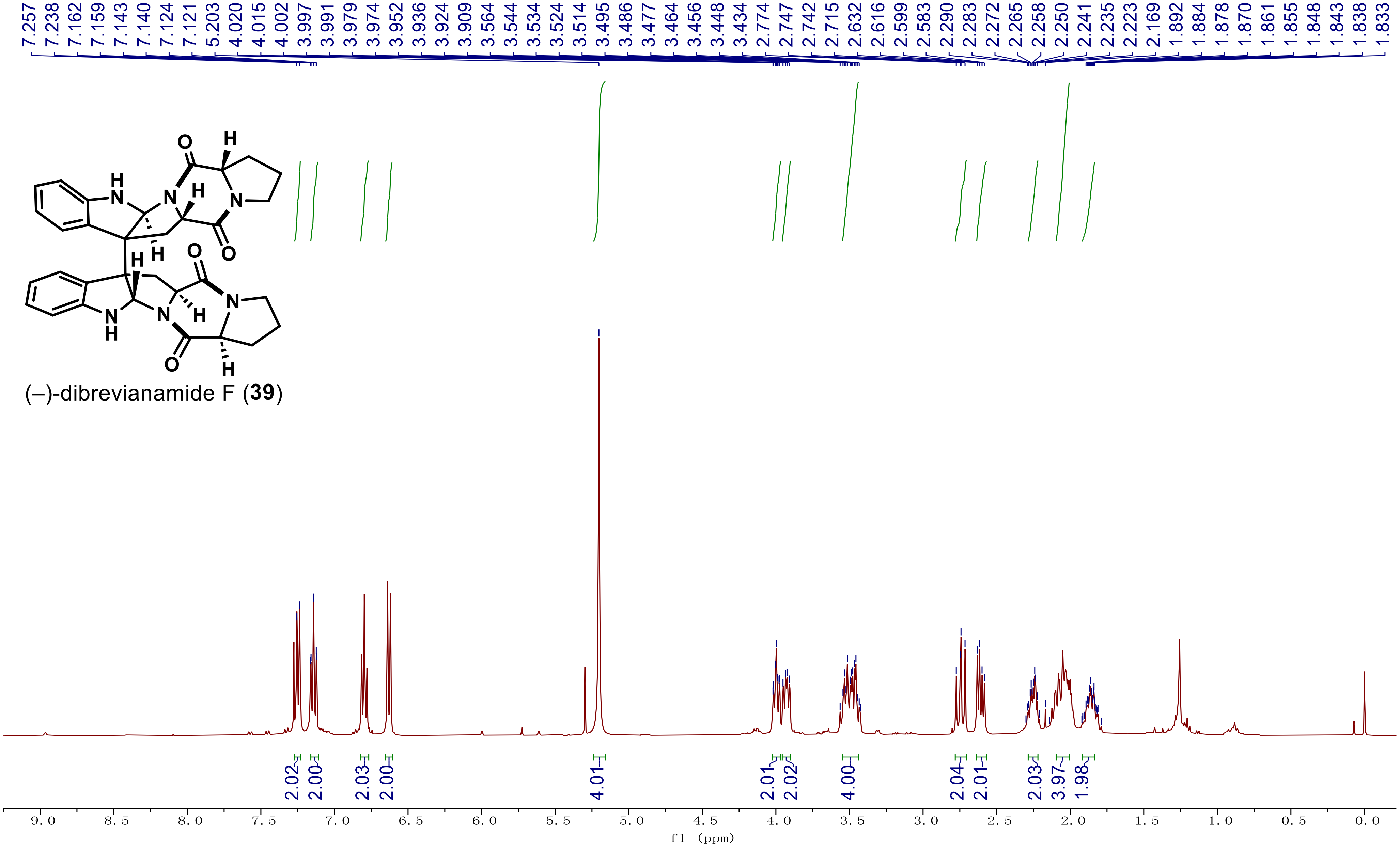

(–)-dibrevianamide F (**39**)

[1] 1H NMR Comparison (–)-dibrevianamide F (39)[10]

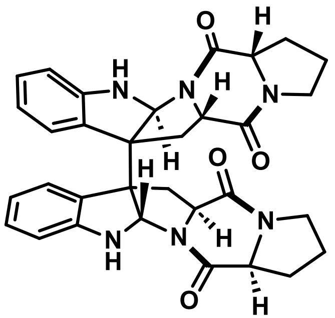

(–)-dibrevianamide F (**39**)

Reported[10]
in $CDCl_3$ (500 MHz)

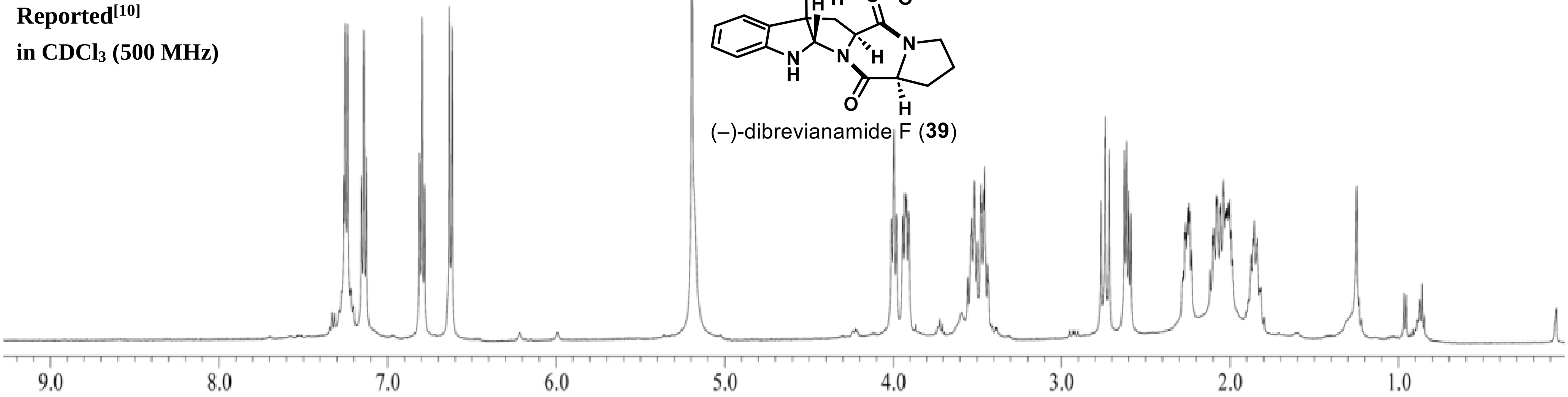

Synthetic (This work)
in $CDCl_3$ (400 MHz)

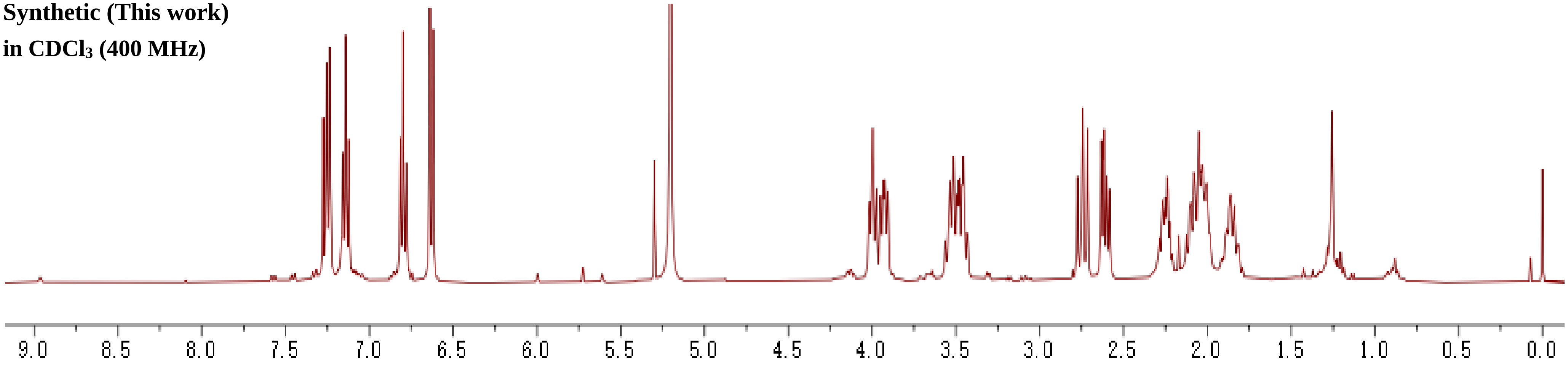

(–)-dibrevianamide F (39) ^{13}C NMR (101 MHz, CDCl$_3$)

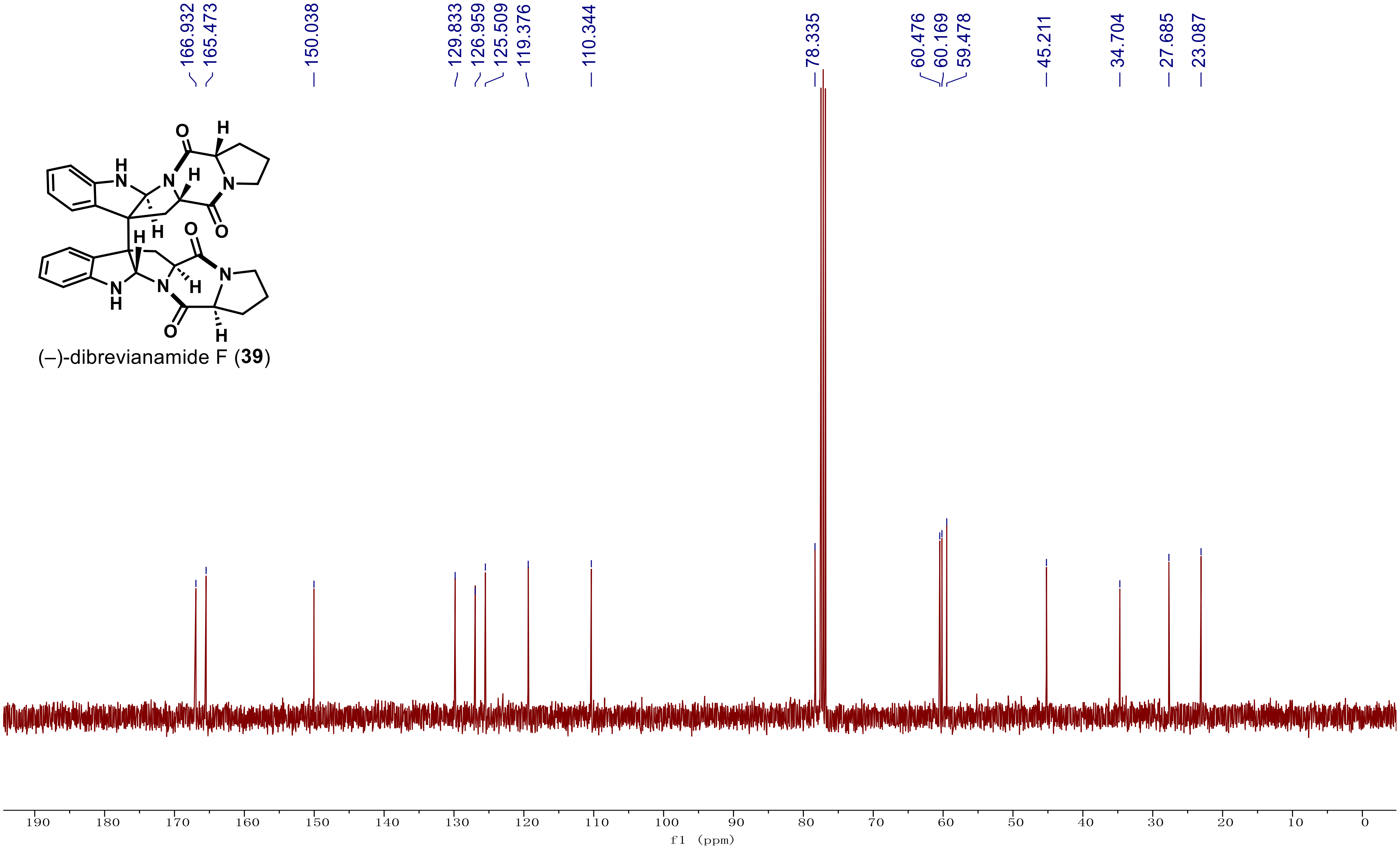

(–)-dibrevianamide F (**39**)

Compound S16 ^{1}H NMR (600 MHz, CDCl$_3$)

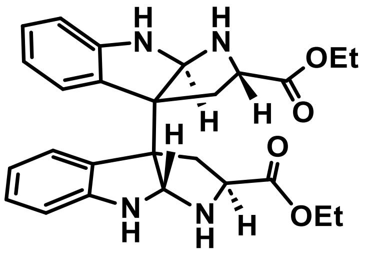

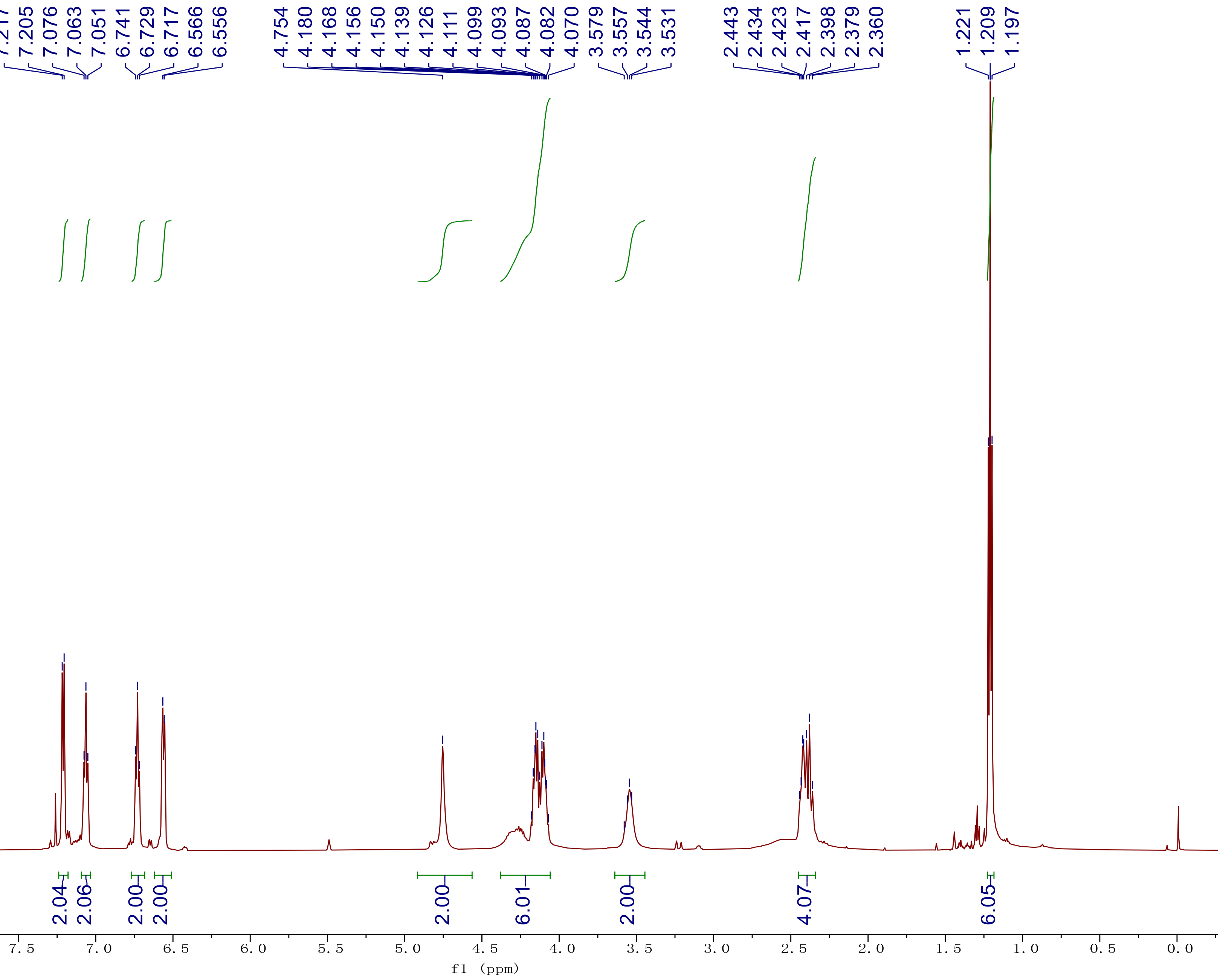

Compound S16 ^{13}C NMR (151 MHz, $CDCl_3$)

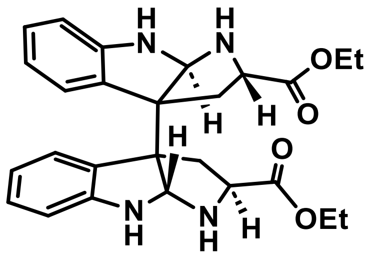

S16

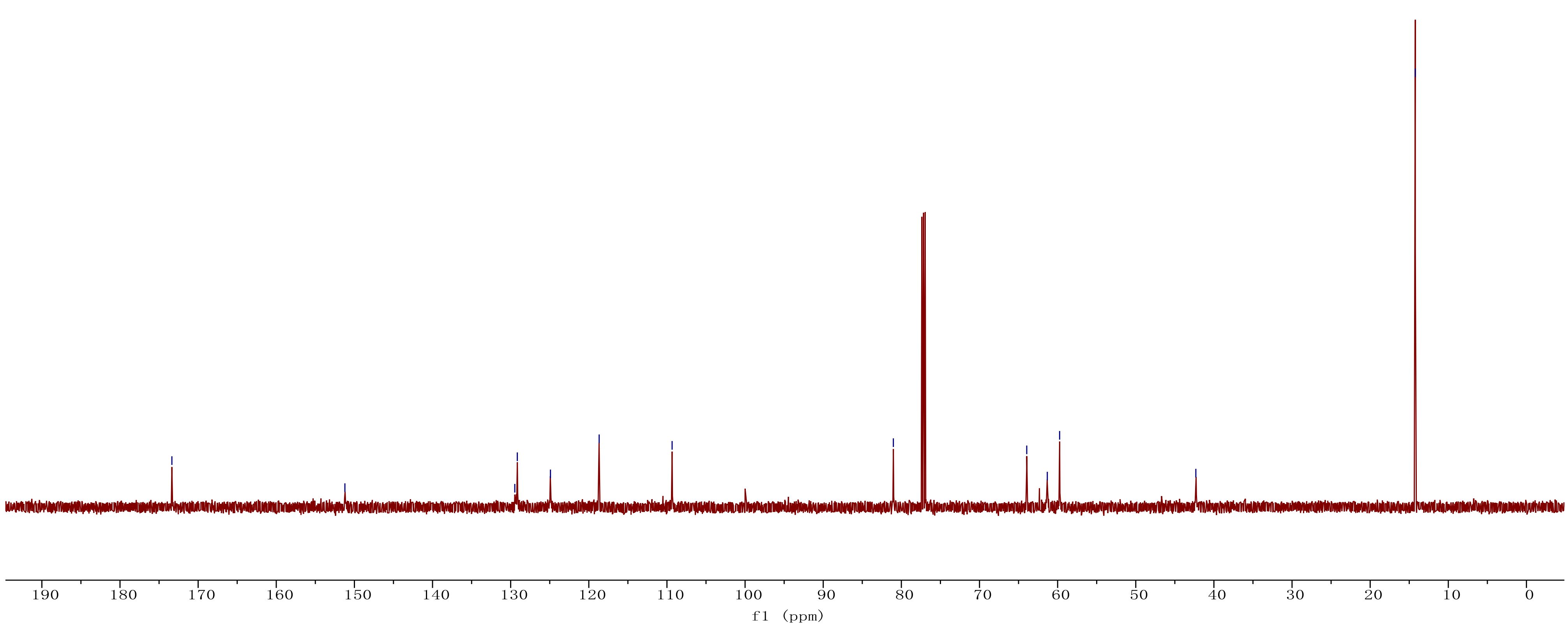

Tetratryptomycin A (3) ^{1}H NMR (600 MHz, *d6*-DMSO)

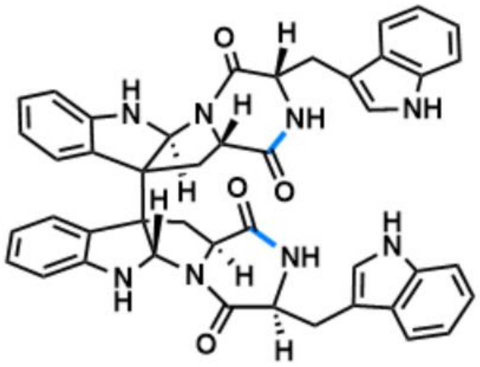

tetratryptomycins A (**3**)

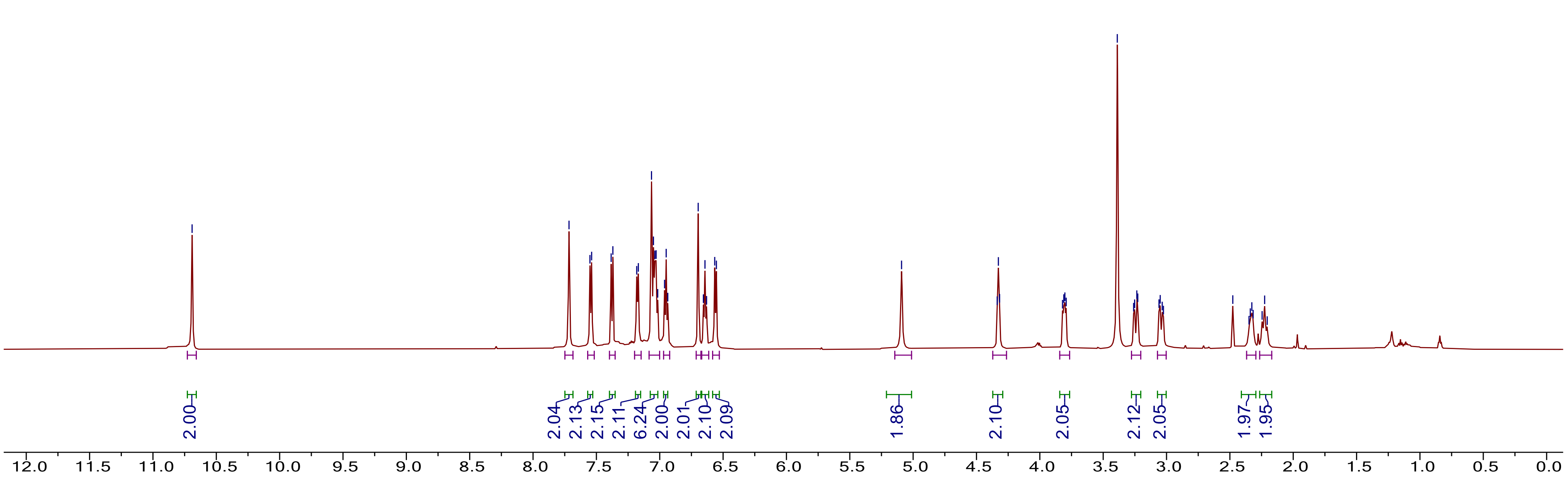

Tetratryptomycin A (3) ^{13}C NMR (151 MHz, *d6*-DMSO)

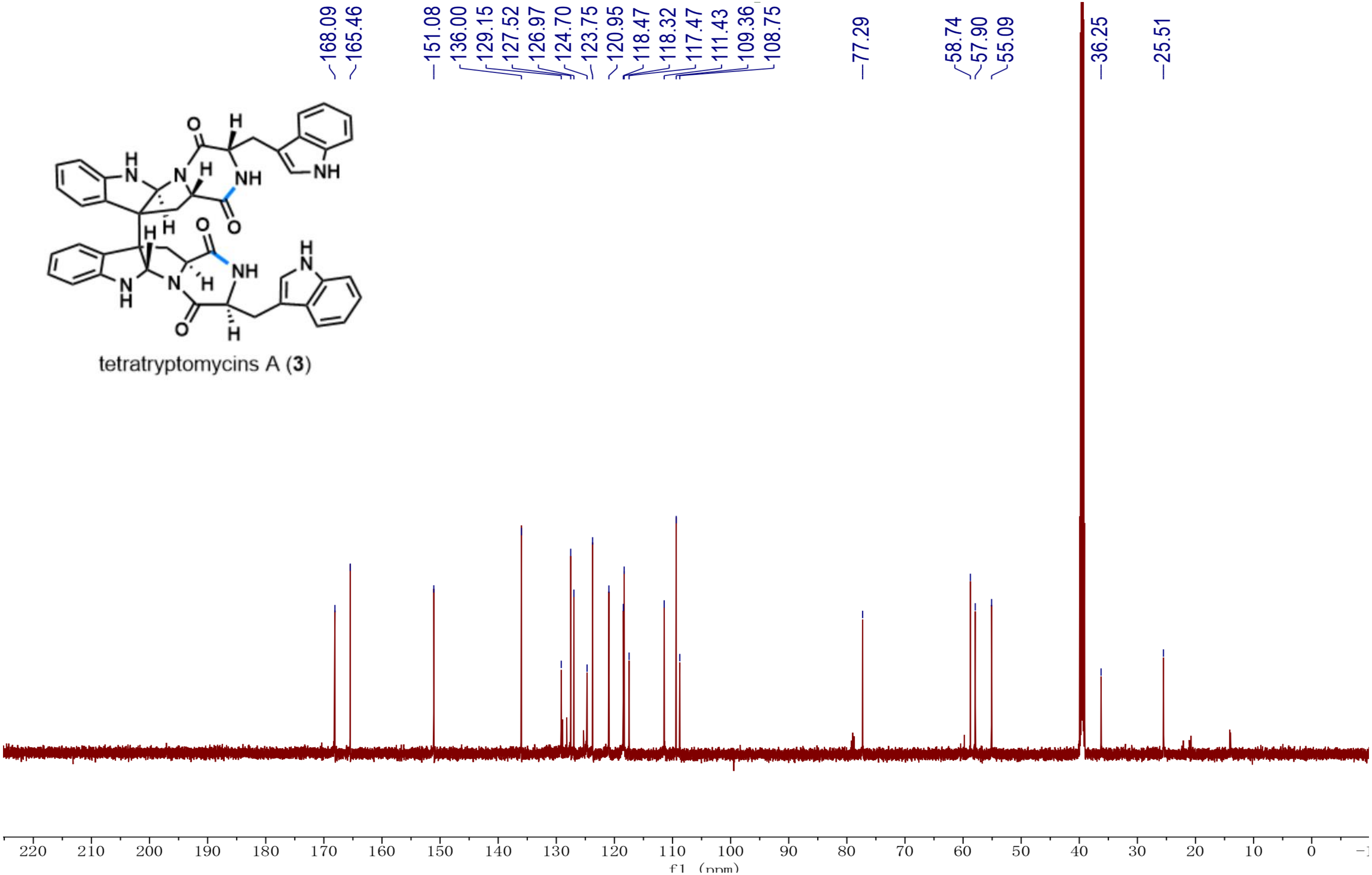

^{1}H NMR Comparison of tetratryptomycin A (3)[4a]

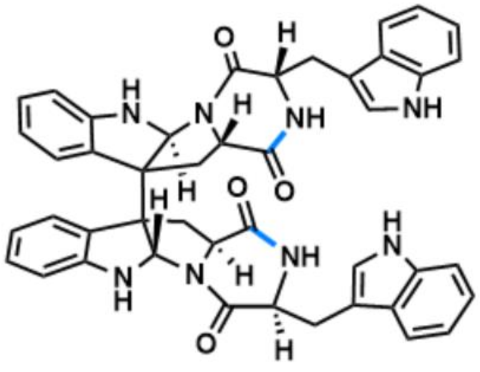

tetratryptomycins A (**3**)

Reported (Qian, P.-Y. *et al.*, *Org. Lett.* **2021**, *23*, 6601.)
in *d6*-DMSO (800 MHz)

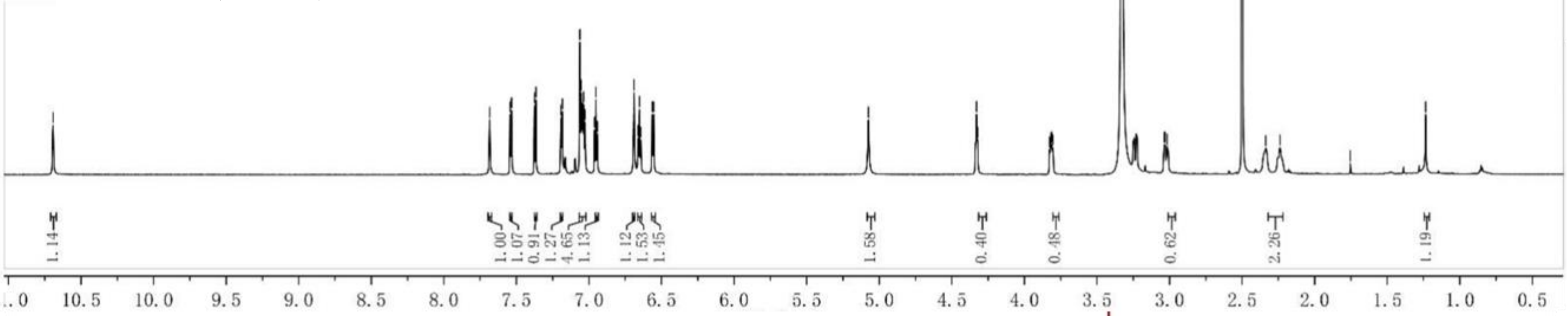

Synthetic (This work)
in *d6*-DMSO (600 MHz)

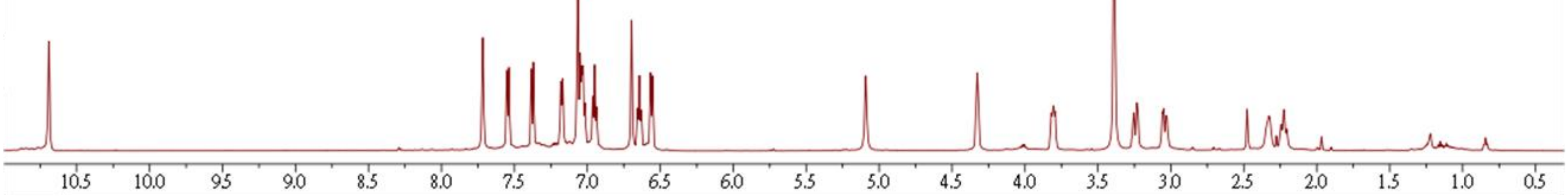

Cyctetryptomycin A (1) ^{1}H NMR (600 MHz, *d6*-DMSO)

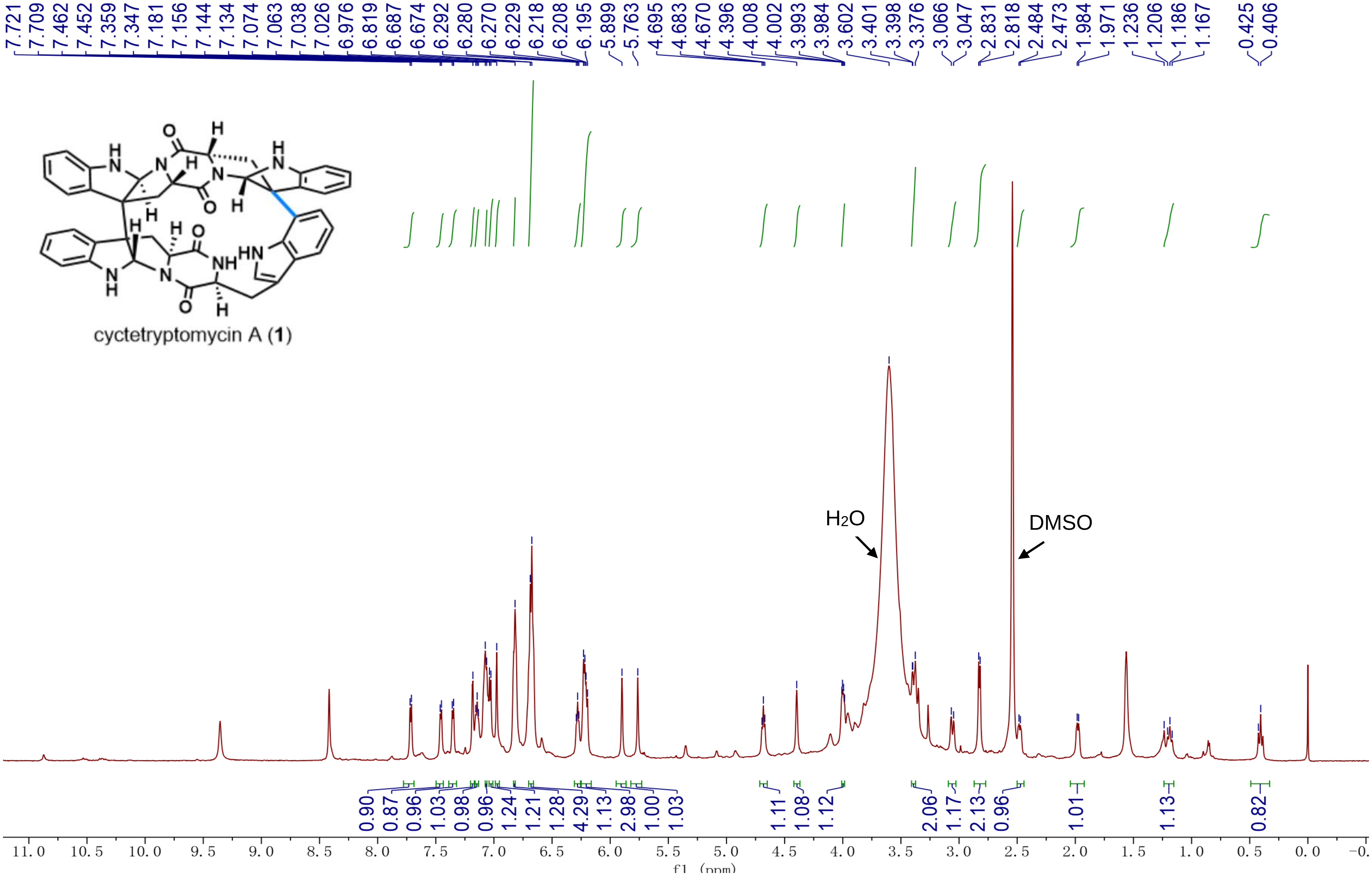

Cyctetryptomycin A (1) ^{13}C NMR (151 MHz, *d6*-DMSO)

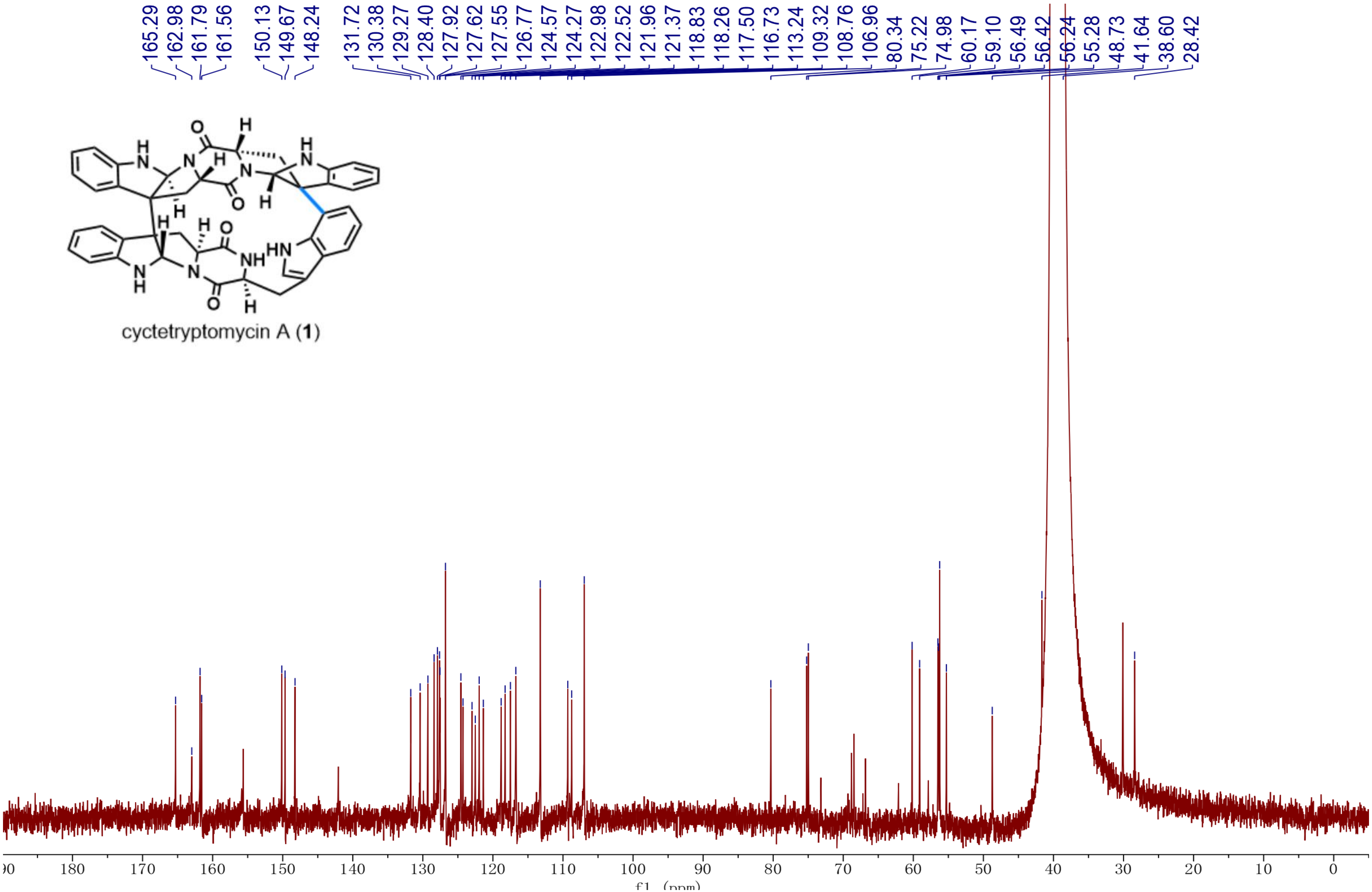

^{1}H NMR Comparison of Cyctetryptomycin A (1)[4a]

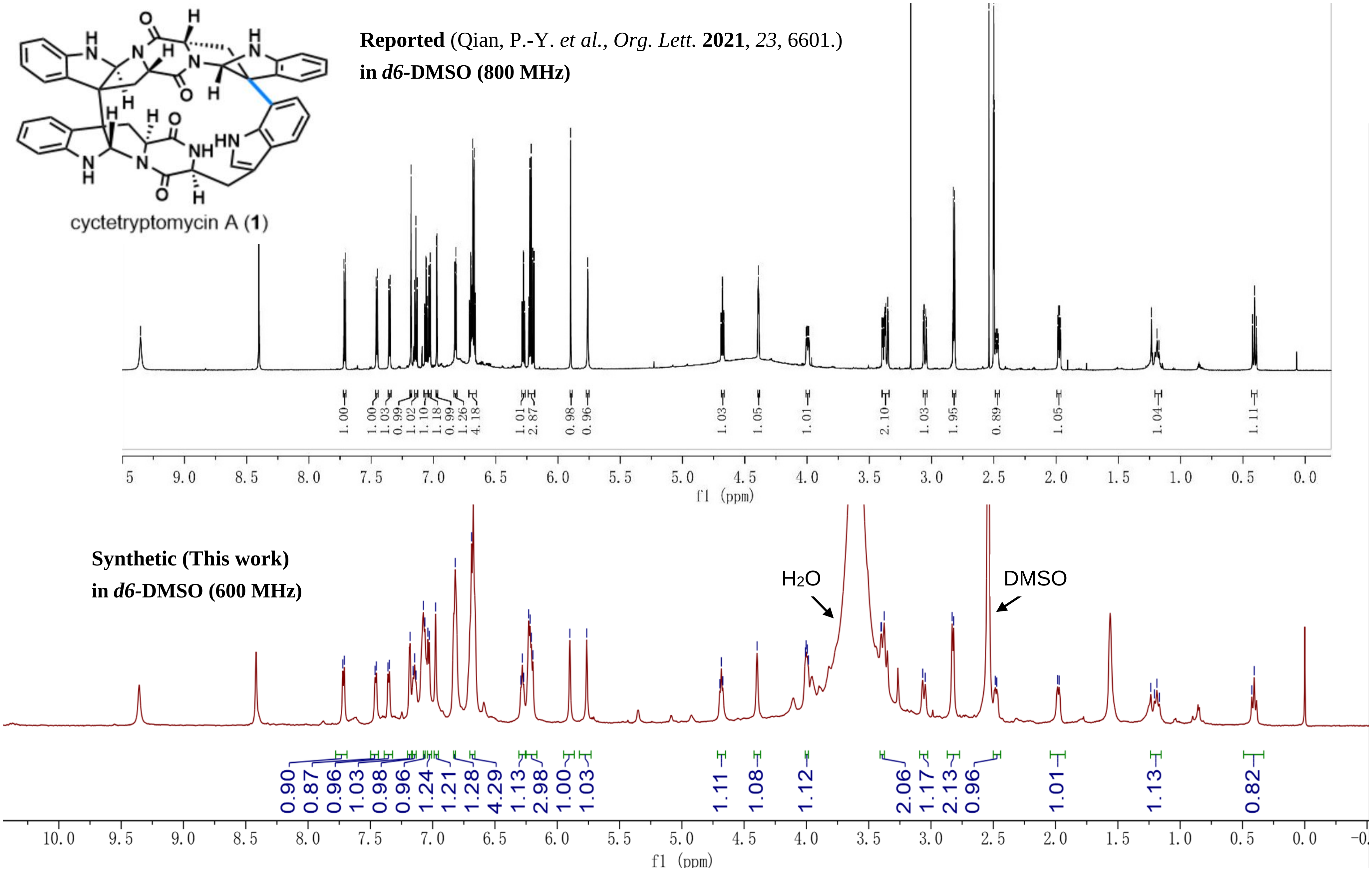

Cyctetryptomycin B (2) ^{1}H NMR (600 MHz, *d6*-DMSO)

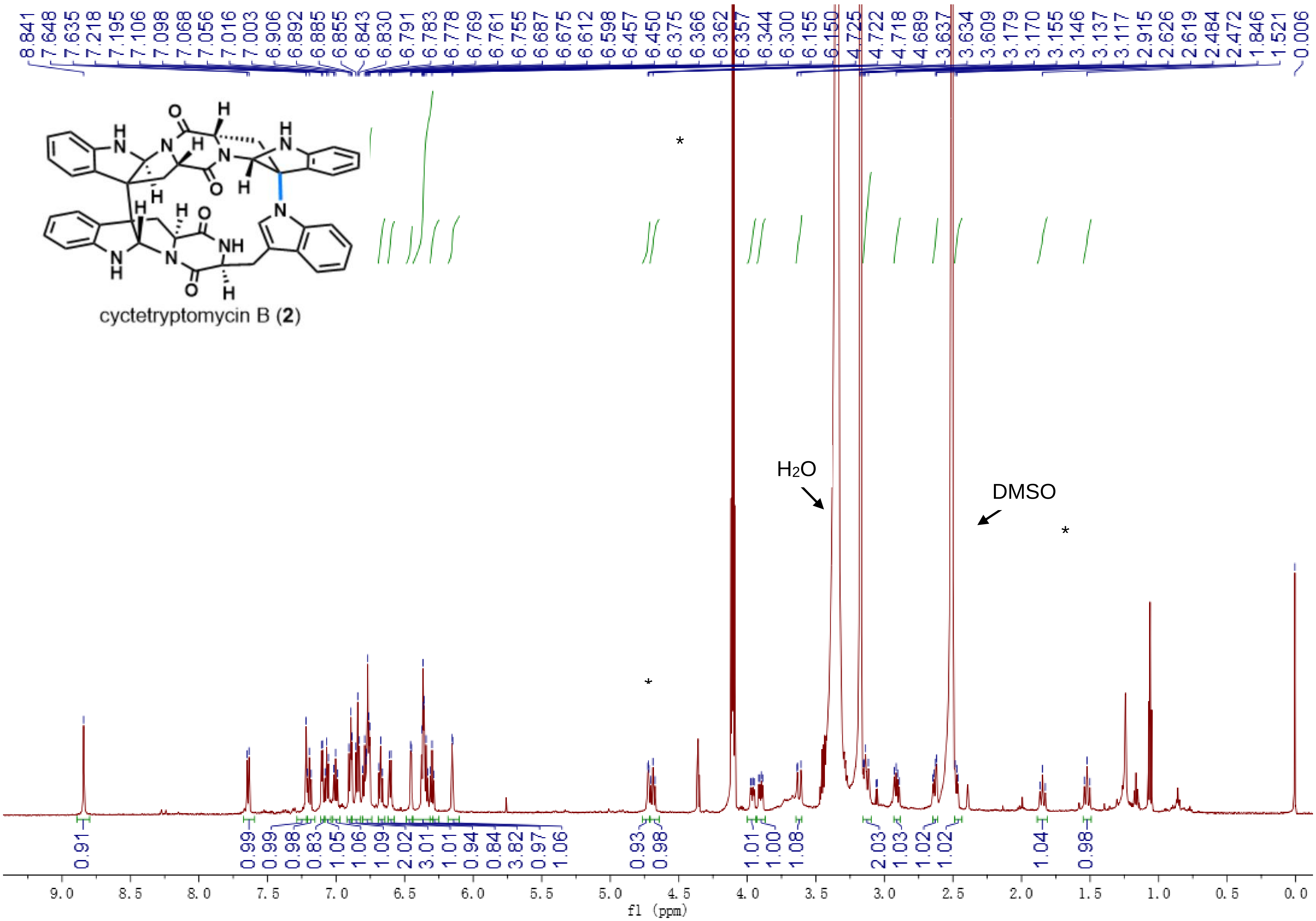

Cyctetryptomycin B (2) ^{13}C NMR (151 MHz, *d6*-DMSO)

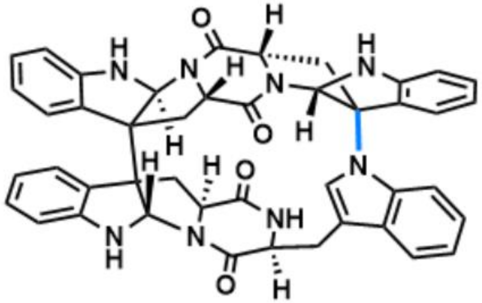

cyctetryptomycin B (**2**)

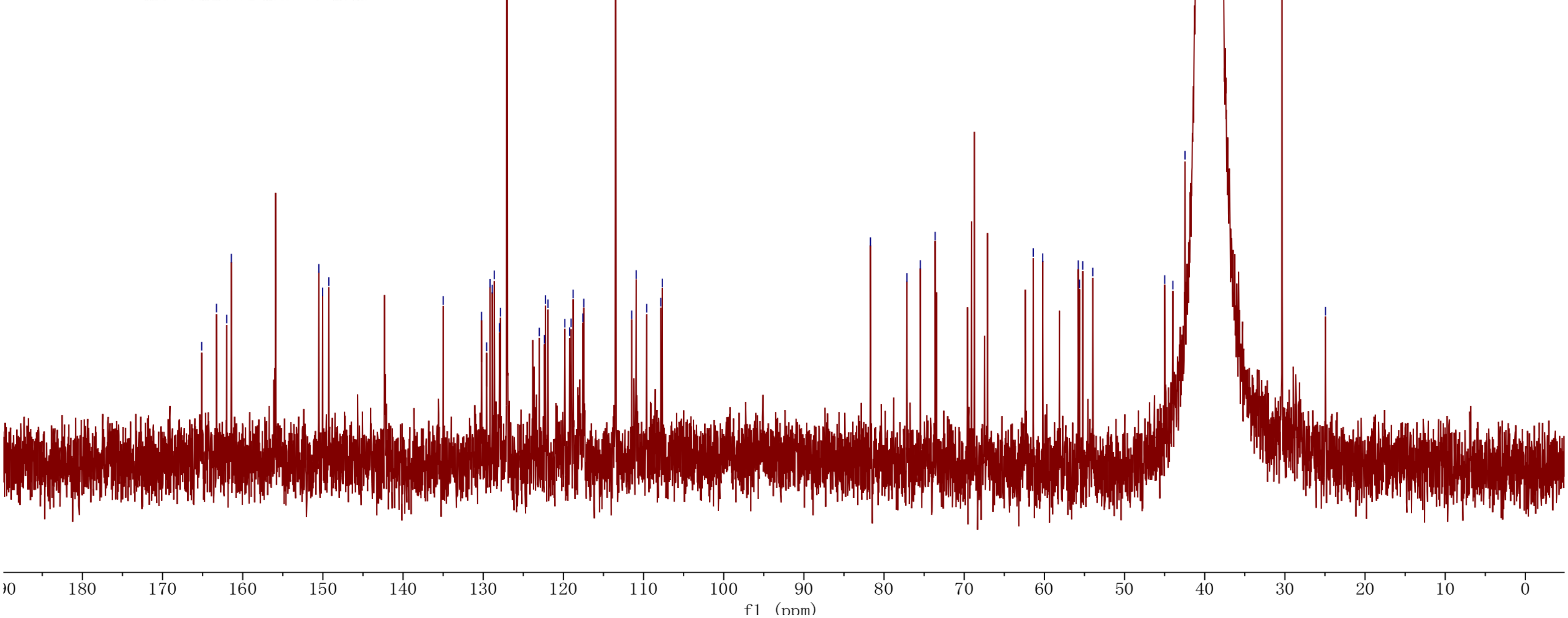

^{1}H NMR Comparison of Cyctetryptomycin B (2)[4a]

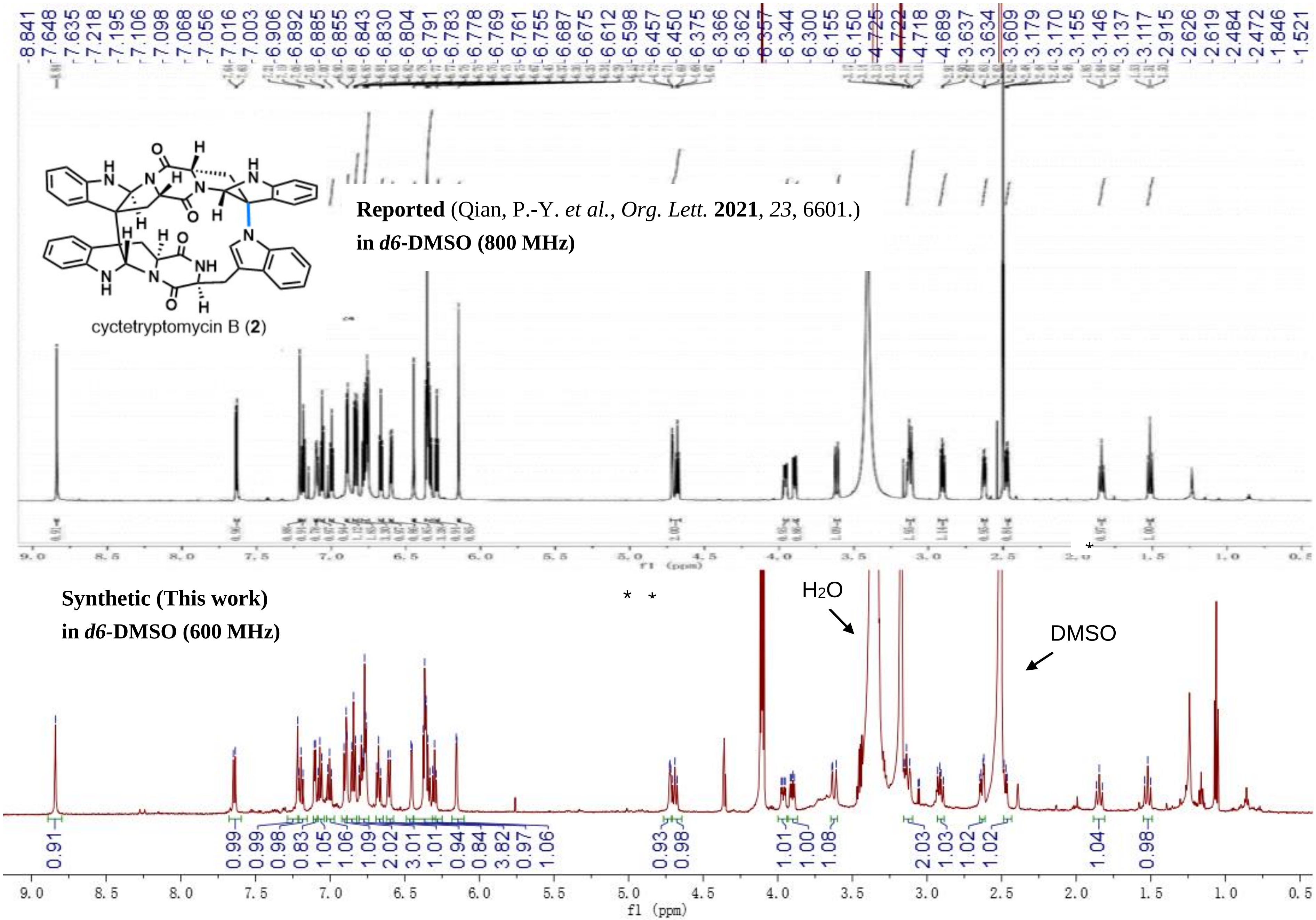

References

1. López, C. S.; Pérez-Balado, C.; Rodríguez-Graña, P.; de Lera, A. R. Mechanistic Insights into the Stereocontrolled Synthesis of Hexahydropyrrolo[2,3-b]Indoles by Electrophilic Activation of Tryptophan Derivatives. *Org. Lett.* **2008**, *10*, 77.
2. Peng, Y.; Luo, L.; Zhang, J.-J.; Ling, W.-J.; Shao, Y.-L.; Wang, Y.-W. Unified Synthesis of (–)-Folicanthine and (–)-Ditryptophenaline Enabled by a Room Temperature Nickel-Mediated Reductive Dimerization. *Synthesis.* **2014**, *46*, 1908.
3. Tadano, S.; Mukaeda, Y.; Ishikawa, H. Bio-Inspired Dimerization Reaction of Tryptophan Derivatives in Aqueous Acidic Media: Three-Step Syntheses of (+)-WIN 64821, (-)-Ditryptophenaline, and (+)-Naseseazine B. *Angew. Chem. Int. Ed.* **2013**, *52*, 7990.
4. (a) Malit, J. J. L.; Liu, W.; Cheng, A.; Saha, S.; Liu, L.-L.; Qian, P.-Y. Global Genome Mining Reveals a Cytochrome P450-Catalyzed Cyclization of Crownlike Cyclodipeptides with Neuroprotective Activity. *Org. Lett.* **2021**, *23*, 6601. (b) Areal, A.; Domínguez, M.; Vendrig, P.; Alvarez, S.; Álvarez, R.; de Lera, Á. R. *J. Nat. Prod.* **2021**, *84*, 1725.
5. B. Gust, G. L. Challis, K. Fowler, T. Kieser, K. F. *Chater, Proc. Natl. Acad. Sci*. **2003**, *100*, 1541.
6. Y. Zhu, P. Fu, Q. Lin, G. Zhang, H. Zhang, S. Li, J. Ju, W. Zhu, C. Zhang, *Org. Lett*. **2012**, *14*, 2666.
7. Gaussian 16, Revision C.01, Frisch, M. J.; Trucks, G. W.; Schlegel, H. B.; Scuseria, G. E.; Robb, M. A.; Cheeseman, J. R.; Scalmani, G.; Barone, V.; Petersson, G. A.; Nakatsuji, H.; Li, X.; Caricato, M.; Marenich, A. V.; Bloino, J.; Janesko, B. G.; Gomperts, R.; Mennucci, B.; Hratchian, H. P.; Ortiz, J. V.; Izmaylov, A. F.; Sonnenberg, J. L.; Williams-Young, D.; Ding, F.; Lipparini, F.; Egidi, F.; Goings, J.; Peng, B.; Petrone, A.; Henderson, T.; Ranasinghe, D.; Zakrzewski, V. G.; Gao, J.; Rega, N.; Zheng, G.; Liang, W.; Hada, M.; Ehara, M.; Toyota, K.; Fukuda, R.; Hasegawa, J.; Ishida, M.; Nakajima, T.; Honda, Y.; Kitao, O.; Nakai, H.; Vreven, T.; Throssell, K.; Montgomery, J. A., Jr.; Peralta, J. E.; Ogliaro, F.; Bearpark, M. J.; Heyd, J. J.; Brothers, E. N.; Kudin, K. N.; Staroverov, V. N.; Keith, T. A.; Kobayashi, R.; Normand, J.; Raghavachari, K.; Rendell, A. P.; Burant, J. C.; Iyengar, S. S.; Tomasi, J.; Cossi, M.; Millam, J. M.; Klene, M.; Adamo, C.; Cammi, R.; Ochterski, J. W.; Martin, R. L.; Morokuma, K.; Farkas, O.; Foresman, J. B.; Fox, D. J. Gaussian, Inc., Wallingford CT, 2016.
8. Chiodo, S.; Russoa, N.; Sicilia, E. *J. Chem. Phys*. **2006**, *125*, 104107.
9. (a) Godbout, N.; Salahub, D. R.; Andzelm, J.; Wimmer, E. *Can. J. Chem*. **1992**, *70*, 560. (b) Sosa, C.; Andzelm, J.; Elkin, B. C.; Wimmer, E.; Dobbs, K. D.; Dixon, D. A. *J. Phys. Chem.* **1992**, *96*, 6630.
10. Saruwatari, T.; Yagishita, F.; Mino, T.; Noguchi, H.; Hotta, K.; Watanabe, K. Cytochrome P450 as Dimerization Catalyst in Diketopiperazine Alkaloid Biosynthesis. *Chem. Bio. Chem.* **2014**, *15*, 656–659.
11. Becke, A. D. Density-functional thermochemistry. III. The role of exact exchange. *J. Chem. Phys.* **1993**, *98*, 5648−5652. (b) Lee, C.; Yang, W.; Parr, R. G. Development of the Colle-Salvetti correlation-energy formula into a functional of the electron density. *Phys. Rev. B* **1988**, *37*, 785−789.

12. (a) Weigend, F.; Ahlrichs, R. Balanced basis sets of split valence, triple zeta valence and quadruple zeta valence quality for H to Rn: Design and assessment of accuracy. *Phys. Chem. Chem. Phys.* **2005**, *7*, 3297-3305. (b) Weigend, F. Accurate Coulomb-fitting basis sets for H to Rn. *Phys. Chem. Chem. Phys.* **2006**, *8*, 1057-1065.
13. Gaussian 16, Revision C.02, M. J. Frisch, G. W. Trucks, H. B. Schlegel, G. E. Scuseria, M. A. Robb, J. R. Cheeseman, G. Scalmani, V. Barone, G. A. Petersson, H. Nakatsuji, X. Li, M. Caricato, A. V. Marenich, J. Bloino, B. G. Janesko, R. Gomperts, B. Mennucci, H. P. Hratchian, J. V. Ortiz, A. F. Izmaylov, J. L. Sonnenberg, D. Williams-Young, F. Ding, F. Lipparini, F. Egidi, J. Goings, B. Peng, A. Petrone, T. Henderson, D. Ranasinghe, V. G. Zakrzewski, J. Gao, N. Rega, G. Zheng, W. Liang, M. Hada, M. Ehara, K. Toyota, R. Fukuda, J. Hasegawa, M. Ishida, T. Nakajima, Y. Honda, O. Kitao, H. Nakai, T. Vreven, K. Throssell, J. A. Montgomery, Jr., J. E. Peralta, F. Ogliaro, M. J. Bearpark, J. J. Heyd, E. N. Brothers, K. N. Kudin, V. N. Staroverov, T. A. Keith, R. Kobayashi, J. Normand, K. Raghavachari, A. P. Rendell, J. C. Burant, S. S. Iyengar, J. Tomasi, M. Cossi, J. M. Millam, M. Klene, C. Adamo, R. Cammi, J. W. Ochterski, R. L. Martin, K. Morokuma, O. Farkas, J. B. Foresman, and D. J. Fox, Gaussian, Inc., Wallingford CT, 2016.
14. Grimme, S.; Antony, J.; Ehrlich, S.; Krieg, H. A consistent and accurate ab initio parametrization of density functional dispersion correction (DFT-D) for the 94 elements H-Pu. *J. Chem. Phys.* **2010**, *132*, 154104.
15. Marenich, A. V.; Cramer, C. J.; Truhlar, D. G. Universal Solvation Model Based on Solute Electron Density and on a Continuum Model of the Solvent Defined by the Bulk Dielectric Constant and Atomic Surface Tensions. *J. Phys. Chem. B* **2009**, *113*, 6378–6396.
16. CYLview20; C. Y. Legault, Unversité de Sherbrooke: Sherbrooke, **2020** (http://www.cylview.org).
17. Zhao, Y.; Truhlar, D.G. The M06 suite of density functionals for main group thermochemistry, thermochemical kinetics, noncovalent interactions, excited states, and transition elements: two new functionals and systematic testing of four M06-class functionals and 12 other functionals. *Theor. Chem. Acc.*, **2008**, *120*, 215-241.
18. (a) Vermeeren, P.; van der Lubbe, S. C. C.; Fonseca Guerra, C.; Bickelhaupt, F. M.; Hamlin, T. A. Understanding chemical reactivity using the activation strain model. *Nature Protoc,* **2020**, *15*, 649– 667. (b) Bickelhaupt, F. M.; Houk, K. N. Analyzing Reaction Rates with the Distortion/Interaction Activation Strain Model. *Angew. Chem. Int. Ed.* **2017**, *56*, 10070–10086. (c) Wolters, L. P.; Bickelhaupt, F. M. The activation strain model and molecular orbital theory. *WIRES Comput. Mol. Sci.* **2015**, *5*, 324–343. (d) Fernández, I.; Bickelhaupt, F. M. The activation strain model and molecular orbital theory: understanding and designing chemical reactions. *Chem. Soc. Rev.* **2014**, *43*, 4953–4967. (e) van Zeist, W.-J.; Bickelhaupt, F. M. The activation strain model of chemical reactivity. *Org. Biomol. Chem.* **2010**, *8*, 3118–3127. (f) Vermeeren, P.; Hamlin, T. A.; Bickelhaupt, F. M. Chemical reactivity from an activation strain perspective. *Chem. Comm.* **2021**, *57*, 5880–5896.
